# Supplementary material for: Exploring the experiences of people who had a stroke and therapists who managed people with stroke during the COVID-19 pandemic: An exploratory qualitative study
Source: PLoS One. 2023 Feb 28;18(2):e0282325. doi: 10.1371/journal.pone.0282325 (PMC9974115; doi:10.1371/journal.pone.0282325)
Supplement: S1 File — (PDF) [file pone.0282325.s001.pdf]

[FEMALE RESPONDENT]
[Other comments:]

Okay, now you can say.

I agree to be recorded.

Okay, and your name, please.

[Curr 0:00:08.0].

Thank you again for taking part in our study, and for agreeing for us to record this interview. I already got your consent, thank you very much for that. Just to let you know, we're going to ask you some questions about your experience with stroke and the rehabilitation after stroke during the pandemic time. After we finish our questions, we'll be doing to assessment to see how you are doing with your activities and with your life after stroke. Just to remind you, there is no right or wrong answer for our question, just feel free to share whatever information or experience you want to share with us, that would be appreciated. Just to let you know, we won't use your name at any of our paper or our reports that will come out of this study. So everything will be unnamed if you share any information with us. Last thing, you can stop at any time. You might ask for a break, or if you want to just stop the interview, it is your right to do so. Just let me know if you want to do anything. Yes, this is all. We're going to start. Just give you a space if you have any question or you want to ask anything before we start.

No, it's fine.

Thank you, if you can please at the beginning tell me about experience of having stroke, and especially during the pandemic time. How was the first moment when you get your stroke, and admitted to the hospital?

So I had my stroke on June 6th last year. I was fit and well beforehand, working. I used to go to the gym every day. I had my stroke, and I was taken to XXX Hospital. I was allowed visitors. I was allowed two nominated visitors. They could only stay for an hour each time, and then I went to a rehabilitation centre for three months after a month in hospital, and I was only allowed one nominated visitor there. It was really difficult, because I've got four children, and my partner. It was very difficult to choose who I could have, and they were quite inflexible.

Sorry to hear this. If you can tell us when the stroke onset started, like when you started having some symptoms of stroke, and how was that happened, and then you moved to the hospital and what they did for you.

So I just stood up, I went to the kitchen, and I just felt weakness. That was all I had. I'd felt well the whole day beforehand. It was in the evening, I just felt weakness down on the side of my body, and I fell to the floor, and then my son called an ambulance.

Then they took you to the hospital right away?

Yes, I had to wait about half an hour.

Were they, right away, told you that you have had a stroke?

No, they thought I had a migraine, and they said it's very unlikely that I've had a stroke, because I don't have high blood pressure, I've got no underlying illnesses. At that time, my face wasn't droopy or anything like that. They said it could be a hemiplegic migraine, but they'll do an MRI anyway. When they did the MRI, they said, yes, I've had a stroke.

Can you tell us, please, after that, how was your journey? You were admitted to the hospital, to the acute [over speaking 0:04:29.5].

Yes, I was admitted straightaway to the acute stroke ward. I stayed there for three days, and I moved to the other stroke ward, which is less intensive, and I waited there until I could get a rehabilitation place.

How long did you stay there?

A month.

Can you tell us what sort of care did they deliver to you?

I had physio, but it wasn't every day because there's lots of staff absences and things like that, so it wasn't very consistent.

During that time, if you can remember, can you tell us how did the stroke affected your body, your activities, your movement?

Oh, okay. I was paralysed down my left side for a few days, completely paralysed, then I started getting movement back in my leg, a little bit in my arm. I couldn't walk.

How about your speech?

My speech was a little bit affected, but not too much.

How about your mood and memory?

My memory was okay; my mood was up and down, because I think I was still in shock.

Hopefully now you are feeling better than you had before. If you can tell us please, what do you think were the main challenges that you experienced as a result of the pandemic? During that time when you were at hospital, and the pandemic was going on, so can you tell us what a challenge you felt during that time because of the COVID thing?

It was just visiting, the visiting restrictions, so I wasn't allowed to go outside the hospital, to the park or anything like that, and I was only allowed certain visitors, so that was challenging.

How about the services, or the care they did for you in the hospital? Do you think it was okay, was enough for you?

I don't think it was enough for me, but I don't think that had to do with the pandemic, it's just the NHS.

It was regular care for everybody?

Yes.

how did the stroke affect you in term of your mobility and walking?

I had to learn how to walk again, that took me about a month, and I used the quad stick, but now I'm able to walk with a walking stick.

Okay, so you walk independently just with a little help with stick?

I have an AFO in my shoe and a stick, and I can walk.

Did they train you for your walking at the hospital, or after leaving the hospital?

Both in the hospital, and then in the rehabilitation centre.

Can you tell me a little bit, when you moved from hospital, what was the case? Did they deliver programme for you at home, or they sent you to a rehabilitation centre?

I went to a rehabilitation centre for six weeks.

Okay, and you started right away, after you left from hospital?

Yes, after a month in hospital. I stayed there for six weeks, and I had 45 minutes of physio in the morning, and then 45 minutes of physio in the afternoon.

Every day?

Every day.

Okay, and you were travelling from your…?

No, I stayed there.

Oh, you stayed there, for six weeks?

Yes.

How was it?

It was okay, but I didn't think it was enough, really.

Do you think that maybe because of the COVID and the restriction on staff?

No. I don't think it was to do with COVID, I just think it's the - there wasn't many staff there.

Yes, so maybe because of the caseload on the staff.

Yes.

They cannot do more for you?

Yes.

All right. let us talk about another point. If you'll please tell me about the care provided by the NHS or other community services after your stroke? Through your journey, if you tell me…

Okay.

Yes.

I don't think it's been great. It wasn't tailored to me, I didn't think. In the rehabilitation, I was with all elderly people, so I don't think they really took into consideration my age, like my needs, my family.

I've got children, and I was different than the elderly people who were there. I don't think they took that into consideration. Then when I left and came home, I was discharged to neuro rehabilitation physio team, and they've been very inconsistent. I've only seen them about three times, three or four times, and they discharged me. So most of my rehabilitation's been myself, looking on YouTube and stuff like that.

We will come to this point; we'll ask you if they provided anything - or online training, or something. We will come to this point later. Now if you can tell me please, what do you think was the impact of the COVID-19 on your care and rehabilitation service?

I don't think it impacted on my care, it was more to do with my visitation, so it affected my emotional health because I didn't see a few of my children for two months.

That was very long.

Yes, it's a long time.

I would imagine as a parent, this is very challenging. Yes, it's a very hard time. Sorry to hear this.

I know that it's hard for them because they worried. They don't see you [over speaking 0:11:14.9]…

Well, it's hard for everybody, I think. For you and for them as well.

Yes.

I'm glad that it was done, and now hopefully you live with them, and you don't have to leave them again, hopefully. can you please tell me about things or people that you feel were helpful to manage your situation at that time?

I think it was just being in contact with friends. I have one friend in particular who I was in contact with a lot on video call, so she provided me a lot of emotional support, and I think that was the most important thing.

How about maybe family, or maybe staff in the hospital, or NHS staff?

I don't think staff. It was mostly family and friends.

Let us move, to another point. We want to see what were the needs. Can you tell me, please, about your needs during the period, in the term of healthcare and rehabilitation; what you felt, at that time, you needed most?

I felt I needed a clear plan of physio, physiotherapy plan, and emotional support as well.

Can you tell us if any of this need were not fulfilled at that time?

The emotional support, because when I got discharged from the rehabilitation centre, they said they'd call me and follow up, but they didn't.

Was it from your side, or they didn't follow up with you?

No, they didn't follow up.

Sorry for that. Talking from personal perspective, what was your main goal for having rehabilitation after stroke?

Just becoming independent, and walking, and getting used to my arm, making my arm functional.

Was there anything else, maybe?

Yes, going back to work.

Do you want to add anything else on? Maybe that was your main goals at that time. now let us move to the management, and how things went at that time. Did the healthcare professional teach you any strategies to self-manage your condition at home after discharging from hospital, or something?

Yes, they came and did an assessment, and they gave me a…

At home?

Yes, to look at what I needed, and then gave me a stair rail so I can go upstairs to my bedroom.

Can you, a little bit, tell us what was happening at that time? What did they teach you to do at that time?

They didn't teach me; they just gave me the physical… A stair rail and a bath stool. They didn't really teach me.

Yes, I was wondering if they talked about anything self-management, like how to maybe self-manage your condition or how to carry out some of training at home, or doing some exercise.

Yes, they came and showed me some exercises to do. I met neurophysio [sic] once in a gym; showed me what I could do in a gym, and then a couple of times at home.

Okay, was that something you did buy for yourself and paid for it or was [over speaking 0:15:18.6]…?

No, it was NHS.

Okay, so that's great. If you think before you left…

No, I wouldn't say it was great, because I only saw them a few times. Since August to now, I've seen them four times.

Oh, I see, yes, so you'll like the follow up with them, so they just maybe met you once or twice, and that's it?

Yes, it's not enough. I didn't - I saw my physio hasn't been enough since I was discharged.

How did you feel before you were discharged from the hospital? Did they teach you enough, or told you enough, what to do at home, and how to self-manage?

Yes, they told me enough what to do at home, but obviously as I'm progressing and getting better, I need to extend my physio programme and do new exercises, and I haven't really been shown that.

Yes, usually patients, they will need to be seen every two or three weeks to follow up with some plans, maybe update the level of exercise or something, to move maybe up level`. about from the self-management strategies that professional maybe mentioned to you, or told you to do at home, can you please tell me what else have you been doing to manage your situation on your own?

I belong to some stroke groups online, where everybody gives each other advice, and then I also look on YouTube, and I follow an American physiotherapist, and she has lots of exercises. Yes, that's what I do.

Who is organising this group? Is it part of association, or…?

Yes, one of them's Different Strokes, Stroke Association, and then there's one I belong to in America, and there's a few people I follow on Instagram, on social media, and we're in touch. They're in America as well, but we help each other out.

Would these people provide individual maybe care plans, or just general everbody?

No, it's a group.

Okay, so they don't assist your individual needs or [over speaking 0:17:39.2], or something?

No.

They just provide generic exercise for everybody to maybe follow?

Yes.

Were any specific efforts taken to improve your mobility, maybe? Did you focus on mobility? Did you have a chance to focus on mobility exercise?

Yes, but like I said, I've got different exercises from YouTube.

More specifically, if you can tell me please, what are you doing either on your own or with other groups, in terms of self-management? How to maybe exercise, or maybe timing or you training, or any kind of - doing something for yourself?

I just do it half-an-hour in the morning, half-an-hour at lunchtime, and half-an-hour before I go to bed, I do my own exercise programme.

How did you find that? Did you find it helpful, useful?

It was helpful.

Is it a thing you feel like to carry on and to manage?

Yes, because I'm motivated to get better, so yes.

Okay, I like that. Some motivation is a really important thing for people, yes, after stroke. I know a lot of patients I have met, they are okay with their physical level and activities, but because they are lacking motivation, so they get depressed and they cannot carry on any training programme or something, they've got terrible, any sort of training. Thank you, for this. Do you want to add anything about self-management or motivation?

Yes, I was just going to say about motivation, so I have different goals. Obviously I wanted to get my hands functional. I'm left-handed, and it's my left side, and my daughter's having a baby in April, so I'm using that as a motivation that I need to be able to look after the baby, so I need to - if I'm feeling tired, I don't want to exercise. It's like, 'No, you need to exercise.' [Over speaking 0:19:48.7].

So maybe this excitement encourages you more to maybe motivate yourself?

Yes.

Okay, well, good look with your goals. The last part of this interview, we want to ask you about plans for future care. When the healthcare services go back after the COVID, and hopefully the COVID is almost over, so when the healthcare go back to normal after the pandemic restriction, what do you think the priorities for your rehabilitation should be?

I think it should be to work my upper limb, and obviously improve my mobility, my walking, and emotional support if I need emotional support.

If you go back, think about the time you were an inpatient, and think about maybe challenges or maybe things that can be done better than what they did to you, so if you can remember anything can be done better?

I think it's about recognising that not every patient is the same. So if you're 85 and I'm in my 40s, it's different, I've got different needs. About modifying your care, individualising it for each patient. We're not all the same.

My last question, how can we ensure services are tailored for people affected by stroke in future waves of the pandemic? If you have, let us say, a pandemic - hopefully we won't have any, but if we have - because I remember you saying maybe they didn't allow you to visit your family, or family to visit you, and this sort of challenges. What do you think can be done better to ensure services, and to help people who get…?

Make families take lateral flow tests before they come and see you, rather than saying, 'You can't see anybody, only one person.'

How about people who might get isolated because they are tested positive, if some patients or people with stroke, they maybe tested positive, can you think of any way of delivering the rehabilitation programmes to them?

Via video.

What else can you think? Especially when they move home.

Just by doing it by video. Especially group support, I think, group exercise.

All right. Thank you very much, for your time. I'm done with my questions. I just want to give you the time to speak about anything, if you feel we have missed anything about your experience with stroke rehabilitation during the pandemic. Please feel free to add anything before we move to the assessment.

No, that's okay.

You feel we cover all the shared experience? All right, thank you very much. Now what happen, I'm going to stop the recording. Thank you very much.

[END OF TRANSCRIPT]

AS: We start now.

Participant: My name is Participant Clues and I've agreed for you to record this meeting.

Researcher: So again, as Ahmad just said, we want to hear your experience and thoughts about how you manage and dealing with life after stroke so…

Participant: Can I just interface, there's a, it's covering your face, there's a thing come up this meeting is being recorded so, either 'leave the meeting or got it'

Researcher: Hit the got it button to get rid of it.

Participant: Got it. Okay.

Researcher: You can see me okay. So yeah we want to hear your thoughts about how you're dealing with stroke and life at this point of time and the ultimate goal is to understand your needs and challenges that you may experience. This meeting will take about one hour. Remember, there is no right or wrong answers, we will simply want to hear your opinions and then you can stop at any time, if you want to or, if you want to have a break in between just let me know, is it alright?

Participant: Yes, that's fine.

Researcher: Okay. Thank you again, and so we will start. So the first question is, can you tell me about your experience of having your stroke.

Participant: When you say my experience, my experience from the minute it's happened?

Researcher: Yes, how did that happen.

Participant: It was, um, I was in bed, I'd gone to bed on the Saturday night perfectly, all right fine, everything was fine. I woke up about five o'clock Sunday morning, I felt fine. I have a TV in my room, I switched the TV on to listen to the news. I was fine, I watched the news for about an hour. I doze back off to sleep. When I woke up the second time, it won't work me out, it was pins and needles in my leg, in my calf with my leg. Cramp, I've got crump in my leg, in my muscle, at the back of my calve, so I swung my legs out of bed, to be able to stamp my foot on the floor to get rid of the cramp. And as I'm trying to lift my leg to stump my foot, it didn't seem right. I couldn't do that now, I thought oh something's not right, I felt okay in myself but then I thought, oh I'll go to the toilet. How I got to the toilet I don't know, I can't remember, but I, I obviously walked to the toilet, so I sat on the toilet, and I, before I got out of it, I got my phone in my hand because I thought something isn't right, I must ring my daughter. That's on the toilet with the phone in my hand, press the button for my daughter's number and I'm sitting on the toilet, i'm saying to my daughter, 'Laura something's not right, I'm having a really funny turn' and she said mom what's wrong, what's wrong. And then I fell off the toilet onto the floor. And while I did that I dropped my phone in the sink, it's next to the toilet but thank God, I was on loudspeaker so she could still hear me and I'm saying, I am on the floor! And by this time now I can't move, and i'm saying Laura I can't move, I can't move! I think i'm having a stroke!

Researcher: And she called the ambulance for you.

Participant: Yes, i'm away. She rang the police, she rang the ambulance because they have to smash the door down to get at me. Nobody could get in the highest because I've got a double lock on my door.

Researcher: Okay, and did you had any cardiovascular disease before? Is it the first…

Participant: Yes, a few weeks before I had a very high, I had a very funny, my heart was beating too fast. And I could feel it pumping pumping pumping. My friend was here, and I said oh gosh my heart's pumping too fast! So she said, let's take your blood pressure, which she did, and my heart rate was, she said, your heart rate is way way too fast, you've got to ring 111 which I did. I told them what was happening, and they sent the paramedics and they came and they said i've got something fibrillation particularly. They took me into the hospital into the HOSPITAL. And they took me in the emergency department and they said, my heart rate was too too high and I think they gave me an injection. And then they left me there, and then after a while they came back and I thought I was going to be admitted, but they didn't admit me, they just said, oh your heart rate's gone back to its normal rhythm. And that was that.

Researcher: And you got discharged, was it one week before the stroke?

Participant: I can't remember how long, it was very long.

Researcher: Okay, and after you fell down on the floor in the toilet, you went to HOSPITAL again, was it HOSPITAL?

Participant: Yes.

Researcher: And was it diagnosed as an ischemic stroke, or was it a blood bursting within brain?

Participant: No it was a blood clot on my brain. I had a CT scan in the machine, the CT. And they said i've got a blood clot on the brain.

Researcher: Okay, and so that you should be admitted at HOSPITAL at that time, I suppose. And how long was the stay at HOSPITAL?.

Participant: Three days, four days. And then they sent me to Moseley Hall rehabilitation.

Researcher: So three days in acute stroke, was it?

Participant: Yes, it was when COVID was there, they've got no staff, and I was treated terrible awful. It was they left in wet sheets, they didn't feed me. I've got one girl only been on in the job for three days and she was trying to move me and she was pulling me by my arm to move me and a nurse came along, the qualified, and said don't do that, you let her arm out of a socket.

Researcher: yeah

Participant: Because she didn't know how to handle me. Anyway, I rang my daughter and complained and my daughter wrote to the hospital a terrible, a big letter of complaint, because I was treated so so bad.

Researcher: So the care is not great, do you think it's because of the pandemic?

Participant: Yes, I believe so.

Researcher: Talking about care, was that anything else that you spotted other than the transferring technique and the feeding and the wet sheets, what’s the other thing?

Participant: I had no, I had no care whatsoever, I mean no one did anything for me in the HOSPITAL. I was left. They even forgot to feed me. I didn't get any food. They just sort of forgot about and of course I was paralyzed I couldn't do anything, the bags of my family left me when I was admitted, they put out of reach, they were on the floor miles away from me, I couldn't get up them because I was paralyzed. The buzzer was on top of the unit, I couldn't get that so I couldn't buzzer anybody. I was just left.

Researcher: Was there anyone around like nurses or any people?

Participant: There was only this young girl who had been in the job three days, she tried her best, but she couldn't do anything for me. Anyway. I ran my daughter, told her that no food, so she rang the ward and spoke to the sister, and she said you best go and see my mother and the sister came and she said, ‘Why have you…’ she shouted at me ‘Why have you rung your daughter?’ I said because I want some help. And she was most annoyed. Anyway, they’ve changed my sheets then. That the day after, then they sent me to… Oh there's a doctor did come, then, and he had a word with me and he said we're transferring you to Moseley Hall, and I said okay. And then they transferred me there and then everything was fine.

Researcher: So do you think that this shortage of stuff is because of COVID?

Participant: I think it was because of COVID. I don't think they could take care of me, though, there wasn't enough stuff.

Researcher: And while you were in HOSPITAL at the acute ward, can you tell me how the stroke has affected your daily activities in terms of mobility?

Participant: I couldn't move. My left side was completely paralyzed. I could only move my right arm. And you can imagine, I was in a terrible state having just I mean mentally as well, I didn't know what was happening to me.

Participant: Just an awful, in an awful position. It was horrendous.

Researcher: So the stroke impacted your left side of the body and how about cognitively and speech?

Participant: My speech was affected but not badly. My daughter tells me when she took me in the ambulance and when she took me to the HOSPITAL she told me that my speech, I didn't know, my speech was affected and my swallowing was affected. Because I couldn't have anything by mouth, for when I got to Moseley Hall. They only had soft foods, I could only have soft foods, because I couldn't swallow. That lasted a couple of days, and then I could swallow.

Researcher: Okay, so it takes a while. So regarding the mobility side like was there any therapists like physiotherapists coming you?

Participant: Not at first. Yes, I did. After I think, a few days, I was there, they were trying to assessing me, and then they started to give me physiotherapy then.

Researcher: It is in the HOSPITAL?

Participant: No, no, no, I had no nothing in the HOSPITAL. Nothing. I was in Moseley Hall when I got to Moseley. I have no set of therapy in the HOSPITAL.

Researcher: So during the time that you didn't get any therapy in HOSPITAL, just talking about that three days, did you do anything yourself to self-manage?

Participant: No. I couldn’t do anything.

Researcher: yeah that seems like not a very pleasant stay.

Participant: Well, they put me on a bed pad because I wanted to go to the toilet. And they left me on the bed pad and because I could move, the bad pad tipped up and tipped all over the bed. And they didn't come back for a long, long, long time. That’s why my bed was soaking wet. I mean, it was just horrendous and they left me in those sheets. Because I couldn't get anybody to come to me, gradually gradually I lift, I was pulling with my right hand the sheet to get these off the bed.

And then, so I was lying then just on the mattress, managed to get the sheet from under me um and that's all I managed to.

Researcher: Participant did you remember, which month or the dates that you had your stroke?

Participant: Yes, the 13th of June.

Researcher: Okay, so it's already a while during COVID.

Participant: Yes.

Researcher: We had touched your stay in HOSPITAL and that's go onto the stage when you in Moseley Hall. During that time did your physical ability starts to regain because of the physiotherapy?

Participant: No, I still couldn't walk. I was in there for seven weeks, I think, six weeks. I still couldn't walk I was in a wheelchair. And then, it was about two weeks, maybe three weeks before I was discharged they started to take me up to the gym.

Researcher: The gym. Okay.

Participant: Yeah to give me exercises. And then they gave me a walker…

Researcher: A frame.

Participant: To try and use a frame. And I wasn't very good on that, I couldn't walk.

Researcher: And during that time, actually after straight away you transfer to Moseley Hall, after how many weeks or how many days that you got in touch with the physiotherapists?

Participant: Oh, it was quite a while, I'd say it was about um, well over 10 days.

Researcher: Okay. And what therapy that you got like physical therapy, occupational therapy, speech?

Participant: It was physical, they were giving me exercises to try and get me to walk.

Researcher: mm hmm so they are physios.

Participant: Basically yeah.

Researcher: Okay, do you go any else like occupational who works on your hand?

Participant: yeah they, the last two weeks before I was discharged, they had me and they gave me cognitive tests to see what I could remember and see you know that my brain was still working. I did well on that. So they did that, and then they took me up to the gym and had me make a canister, make myself a cup of coffee.

Researcher: Good. So regarding the physical therapy that you got at the first of the stay, were the physios all the way present to do your exercises or did they advise you on some exercises?

Participant: No they were present.

Researcher: They were present, all the way.

Participant: Yes, yes.

Researcher: How often did they visit you?

Participant: Only once a day.

Researcher: Okay, and how long was the section usually last?

Participant: Oh, half an hour?

Researcher: Okay, but then last time I saw you got very independent, obviously.

Participant: When they discharged me, I couldn't walk only on a walker, I couldn't use my arm at all, you could see it was like that.

Researcher: Like fisted.

Participant: yeah. You couldn't open it, could not open my hand, couldn't move my arm, it was like that.

Researcher: Like very rigid, high tone. Yeah.

Participant: So I have to move and put my hand on the walker to walk, and when I was discharged they have to sent two carers in. Well, they weren't carers really, they were just there to see that I was safe. I still have to do it myself. I had a perching stool in the bathroom and I have to wash myself. They just were there to see that I didn't fall. So I started to wash myself and then made myself some toasts. They would butter it, I done the toast and then they would go. They were only here about 15 minutes.

Researcher: So to regain your independence, did you get any community physio or any services?

Participant: I had to wait 12 weeks, they came to assess me and they said, the waiting list was 12 weeks before I had physio.

Researcher: Okay, so they are the physios from the NHS community team.

Participant: Yes, yes, they were from Mosley Hall.

Researcher: So what else support that you get in the community then, any else other than physios?

Participant: No, none whatsoever.

Researcher: Okay so um after they came in, the physios, how long did it take you to be independent back to your normal life?

Participant: I was already independent before they came in! What I did, I exercised myself, and I was determined to get downstairs. So I was doing this with my hand to get it open so I was able to hold grip. And so i'm going to get down the stairs. I've got to get out because I was trapped in this flat, I couldn't go anywhere. So I started then, there were the seven steps out to go to the first landing, so I went down crab style, held on to the rail, and went crab style down the first seven steps and back up. And I did that for about 10 days up and down, up and down twice a day, maybe three times a day. And then once I've mastered that set I did the next seven and then the next seven. By the time physios came 12 weeks later, I was already doing the stairs and getting down to the bottom.

Researcher: Cool it's very good that you can self-manage, but at the same time, I mean the services should get to you much earlier than that.

Participant: So, but if I wouldn't have done that I wouldn't be walking now. If I just have to sit here and I have no treatment for 12 weeks three months, now that would have been finished, that would have been it, had been crippled for life.

Researcher: So what do you think were the main challenges that you experienced as a result of the pandemic in terms of assessing the services?

Participant: Yes, it was the fact that they couldn't come. They haven't got the staff. They are all under stress, I know that. There was a waiting list for everything, they send carers in after the initial few weeks of just monitoring me. I had to have private carers come in and then I had to pay for that and they were absolutely hopeless, they did oddly anything, they were rubbish. That after four weeks I said I don't want you anymore, and it costed me 700 pounds which was disgusting.

Researcher: So short of staff is one of the big points. Any other thing that you think it's all because of the pandemic that's why you get a long waiting period?

Participant: Well, this is what I think. It must have been I can't see, I mean. I can't understand why I didn't get any treatment earlier. All I can put it down to and I'm being very forgiving is the pandemic that they couldn't you know, they just haven't got the resources. But it was just unfortunate for me, but maybe fortunate for me that I was self-motivated and I was doing it myself when they came, they could not believe what I could do.

Researcher: Did you ever think of hiring or going to private physios?

Participant: yeah, but it was 50 pound a day. An old age pensioner, I'm on a pension, I live alone, I've got one income that is my pension. I have to top that up with pension credit, how can I find 50 pound a day for private? I can't. It took every penny, my 700 pound to pay the carers that came in, just to get me washed and they wouldn't, all they did was supervised me, watching myself. All that time they were coming I never had my feet washed once.

Researcher: um okay so talking about something that's more positive, do you think that there is anything or any people that you feel were helpful to manage your situation, including family and friends?

Participant: It was my family, my daughter. She did everything she could, she is a nurse, she encouraged me to do, to be motivated, and at one time my mental health was down. I kind of lost the will to live in lots of ways because I was here 24/7 on my own. My daughter couldn't be here and only on a day off. My mental house was dreadful, I said to my daughter, I'd rather be dead than be in the situation. But she said Mom, get a grip, you can't be, you're a strong woman, you've got to fight. And I thought to myself and I said no I'm not going to be beaten, i'm gonna beat this, i'm going to show everybody that I can do this on my own and that's what I did, and I got my mental capacity right and I was doing all this physical exercise as well.

Researcher: Good mentality! So your daughter can get to you after you got discharged from Moseley Hall, but had she ever got a chance to visit you during your stay in the hospital?

Participant: No, you're only allowed one visit a week at that time because of the pandemic.

Researcher: And do you think that impact your mental health?

Participant: Yes, definitely yes. Because everything, when I was incapacitated, when I couldn't move, everything I wanted I had to ask for. And the nursing staff there were very, very good, I can't complain about them, but they were really up against it because they have to do everything for me, whereas if you got visitors, they would take over a lot of the care. You know if he could have let my daughter come in, or my granddaughter or my friends, they could have done a lot for me while they were visiting. But as it was, everything I wanted, every time I wanted to go to the toilet, I had to call the nurse.

Researcher: So touching about your needs then, in terms of the needs for mobility say walking, rehabilitation, can you tell me what are your needs during that period of time and did your needs been fulfilled?

Participant: Everything, it was just everything. My needs were multiple. I couldn't cut anything up I, I could only use one and I couldn't cut my food. I couldn't get up and go to the toilet.

Researcher: Washing and dressing.

Participant: I couldn't do that, I couldn't dress myself, I couldn't wash myself. They used to come for me each day and take me to the bathroom and either wash me down or put me in the shower and shower me, I couldn't shower myself.

Researcher: Do you think your needs have been fulfilled during that period of time?

Participant: Only at the very last apart. I'd say for the last three weeks of my stay there. For the first three weeks all my needs were catered for. They tried their best, I'm not complaining about that, they couldn't help that, they've got far far too much to do, because they were looking at for a whole ward and we're all in the same face, they couldn't have visitors, there were sicker of people than me. There were people that were at a bleed on the brain. I was lucky in a way i'd only got a

clot, but there were people that were really in the distressed way and couldn't communicate or anything so really I felt bad for these nursing staff because they were, and I was trying my best not to call them unless it was absolutely necessary.

Researcher: Okay, so to name a few, say three to four, what was your main goal for rehabilitation during that time?

Participant: When I was in the hospital?

Researcher: Yeah during the hospital and Moseley Hall.

Participant: Just to get out to there.

Researcher: Okay, so you regain your independence, I would say.

Participant: No, I didn't gain my independence until I got home.

Researcher: OK, so the primary goal is just to go back home and to get out.

Participant: yeah yeah and to get back to my normal self yeah.

Ahmad Sahely: What was the goals after you move to your home?

Participant: To get out of this building, to get out of this flat.

Ahmad Sahely: yeah okay good.

Participant: That was the main motivator.

Ahmad Sahely: Maybe the main thing was about mobility in the community.

Participant: Yes, to get out and get the life that I normally live. See my friends, go to the theater, go out for a meal, go to the library, shopping.

Ahmad Sahely: Participant you have been doing a very interesting chat with us. I was enjoying, listening, and I feel sometimes sorry for the way that you have treated in the hospital. Maybe because of the COVID, even though I feel there shouldn't be any excuses for not caring for patients while they are in the hospital but anyway i'm glad that you have recovered too much to reach this achievement where you are now and yeah I think you have done a great job, especially with management and having those for you to reach and to keep moving and not to lie down for any challenges.

Participant: My daughter wrote to the hospital, a sharp letter to the chief executive of the hospital and they wrote a five page letter back, which I can show you when you come.

Ahmad Sahely: yeah okay.

Participant: I've still got the letter.

Ahmad Sahely: yeah this is interesting, well hopefully we can have some time to see that. Yes Jessica, you can carry on with your question, thank you again for this.

Researcher: yeah so we really fascinated by your determinations to self-manage your situation. Actually did any healthcare professionals teach you any strategies to self-manage in Moseley Hall when you got discharged?

Participant: Not really no, as I say, they gave me a little bit of, there was some, about six stairs, mobile stairs, and one day they said one day we'll try to get you up the stairs because they knew I got 27 of my own stairs. I was very pleased about that, that will be good, they did it once, that's all, one day once. They had me go up and come down, and I never ever did it again. And I thought that was disgusting because they knew that I'd got to get my independence, to get out of this building. They knew I had 27 steps to go, and yet they didn't give me physio to walk, to get me to go and walk down the stairs.

Researcher: And there were basically no advice to you during the gap of that 12 weeks that you were waiting.

Participant: No, none whatsoever, no one rang me, they came and assess me one day, and then they told me, we're sorry we won't be able to send physio, you were, I know you're on a waiting list and it's only 12 to 13 weeks. Well I cried when they left, how can I wait 12 weeks for physio.

Researcher: Yeah i'm so sorry to hear about that really.

Participant: And that's when my mental health, so I started then thinking, I might as well be dead. I may as well not recover because I can't just sit here and be like this.

Researcher: Very glad that you have your daughter to support you.

Participant: yeah yeah.

Researcher: And do you get your community physio now, at this point?

Participant: Yes, they've been very good, since I started to come, i've got no complaints about that. They're not only allowed so many weeks and now they're finished with me now.

Researcher: Oh so they're not coming in.

Participant: Tomorrow is gonna be the last one.

Researcher: How many sections, or how many months, did they come to your house?

Participant: Oh i'd have to check up I can't remember. But they know how many weeks they are allowed to get in touch with them. Well when he comes tomorrow i'll ask him.

Researcher: Okay, and how often do they come, once a week?

Participant: yeah sometimes they have been twice a week, but mainly it was once a week. Occasionally, they are twice a week, but they were good, they were very good because they could see I was doing the exercises when they're not there. They gave me certain exercises and i'd follow those and then, when they come back again, so we can tell that you've been doing your exercises.

Researcher: yeah you had been improving very, very much.

Participant: yeah yeah

Researcher: Okay, so um do you think that the exercises that you are doing, like the ones that opening your hands, the ones that you're doing your stairs on your own, was that useful for you?

Participant: Especially the walking because that's you know I think I walk very well. My arm and hand wasn't as good. I think I need a lot more work done on my arms and my hand to be able to manage, to be back to where I was.

Researcher: And did anyone ever set goals like involve goal setting sections with you?
Say community physio or Moseley Hall physio, anyone?

Participant: No.

Researcher: not really okay. I think it's very good that you have been self-managing really and you had your own goals to be determined, to be independent and get out of your home and be back to the community. So talking about future care, then say when the health services starts to reopen after COVID, what do you think are your priorities for your rehabilitation?

Participant: Well me personally. I think i'm doing well. I think I don't know what else they can do for me now. You know, maybe just own mind… I think it's up to me now to just motivate myself to improve.

Researcher: yeah you're very independent on your own on the program.

Participant: yeah yeah.

Researcher: Okay, so do you think that, i'm say thinking about if you, touchwood, if you have your stroke during in another pandemic, what could be done better by the services?

Participant: Oh, they could have done so much better. From initially taking me into hospital when I was in the HOSPITAL, they could have 1000% better. in Moseley Hall, it could have been so much better, I could have had more physio, instead of the little bit. I mean I didn't get much physio in there, they hadn't got the staff, they hadn't got the resources so they couldn't, I mean there was some days I had no physio at all because they just couldn't fit me, in some days that they just couldn't see me. I had nobody come and see me. Oh Sam do I have physio today? Oh no you're not having any today. And that upset me because I thought well if i'm not getting physio, i'm not getting better. Yeah they could have done a lot better, but again, it was the pandemic I think.

Researcher: yeah so the physios can come more often during Moseley Hall, what about the in-patient period in HOSPITAL?

Participant: I mean that is the in-patient period. When I was in Moseley Hall, some days I don't have any physio, I didn't have it every day.

Researcher: So say during the hospital period in HOSPITAL, do you think more people or more care can be given?

Participant: Oh absolutely yeah.

Researcher: Any other advice that you think we can improve on in terms of care?

Participant: Well, there was so much room for improvement, the whole overall. I mean, I feel very, very sorry for people who have a stroke, because mainly they're old people anyway, I believe. I know younger people do have strokes, but and it's very frightening and it's not like any other illness, where you're, I mean you're not in pain, thank God, but just your whole life, it's like flicking a switch, go from being completely normal to being absolutely helpless like a baby. You have some everything done for you, you can't dress yourself or feed yourself. So you need an awful lot of support and I don't think they take care of your mental health.

Researcher: Mental health, what do you think can be done better in terms of helping?

Participant: Oh, I think they should talk to you more, tell you about what's happening, what the prospects are, what you can do to help your mental health. Give you more encouragement. You know I mean it is time, I suppose, but you're just left in a bed, and you're helpless.

Researcher: And during your stay anywhere, besides, apart from nursing staff who talked with you about how you're doing, taking care of your mental health as a whole, anyone else you were in touch with, for example, like sister or anywhere anyone in the ward?

Participant: No, the sister on my ward was very good, you know speak to you alone, she hasn't got time to talk, I never had a conversation with her ever. I would have liked to have known, how am I doing, am I doing well, will I get over this, will I ever walk again, would I be able to look after myself again. I had none, there was nobody feeding mental house at all. There was nothing was said about your mental house, we were just left.

Researcher: yeah I think mental health is definitely one of the part and also how they allocate the manpower to get in touch with everyone.

Participant: And of course because of the pandemic, what was bad was you didn't see any family or friends! They couldn't even talk to your family, they couldn't help you, with your mental health. You were just left in a bed. I mean sitting in a chair all day.

Researcher: Do you ever had a chance to speak with your family and friends over the phone?

Participant: Yes I've got a mobile so I could see my family and friends on the mobile. But if I hadn't got a mobile no you wouldn't. And I had got an iPad but they got no wifi. Wifi wasn't working so I couldn't use my iPad or watch films. There was no TV in the ward at all. Nothing to pass the time away. It got nothing to do.

Researcher: So yeah that's three big good points that we would notice for our future care. I think that's really all the questions that I need to ask about your experience. As a whole, is there anything that you want to add, Participant, about your experience or anything that you want us to be aware of?

Participant: No, I mean it's just a very unfortunate time for me to come to a stroke, hopefully when we're not in a pandemic, things will be better uh huh.

Researcher: yeah hope you are all good.

Participant: It's done and I do feel sorry for the nursing staff because so we're under an awful pressure, awful lot of pressure, and I know i'm complaining, i'm not complaining but giving it to you as it is, and I don't like to put the NHS apart, they do a sterling job, but my experience, because of the pandemic, I think this horrendous. And I never ever wants to go through it again.

Researcher: yeah hope you don't, but then certainly it can be improved.

Participant: Oh, so much so.

Researcher: Is there anything that you want to add or else we can move to the next session.

Ahmad Sahely: We actually appreciating what you are sharing with us about your experience this evening, thank you very much.

Participant: Thank you.

Researcher: So now we'll stop recording.

Researcher
So first of all, thank you very much for participating in our study about your experience with physical rehabilitation after your stroke during the pandemic time. And before we start, if I can just have your name and your consent to agree to record this interview.

Participant
Yeah, so my name is Participant Ali Khan, and I give consent to record the interview.

Researcher
Alright, thank you very much Participant. So thank you, again, for your participation. And as you know, the ultimate goal of our study, just to collect and to explain the situation of rehabilitation and other surfaces for people after stroke during the pandemic, so, the NHS and other surfaces can be more prepared if something happened in future and if we have any pandemic, or even now, still, we are affected by the pandemic situation. Just to let you know, and I think you have read that in the information sheet, it is your right to stop at any time, if you feel uncomfortable, or if you feel fatigued, or you want to take a break or rest and then we can continue just for relax. At any time, you can stop, we're fine with that. And at the end, we'll have some assessment about how are you doing with your daily activities. And, you know, because of the structure we can accept, explain the situation of our participants in this study. Alright. Do you have any question before we start?

Participant

No, no.

Researcher
All right. So just let me know, tell me the story, and tell me your experience with stroke

first. Participant

Yeah, so this year, I had a stroke in June. And I, I had have a heart defect that was from birth, just minor, but I always had high blood pressure because of it. And just before the pandemic, they realised, Oh, that one of my vessels is narrow. Yeah, that procedure basically caused the clot that ended up being the stroke. So my role is better. Obviously, you know, very rare complication happen. So I was, yeah, I was in hospital in July, because after the procedure I felt a bit weird. They said, because I had a procedure. Yeah, the stroke got a bit confused in that after effect of the procedure.

Researcher
But that was your first stroke experience. You have…

Participant
no, I've never had any, and to be honest, I work in hospitals. Okay, guys, I work at XXXX. Obviously, I'm on sick leave now, I must admit my experience of stroke, I didn't have that much experience. And then to know more about epilepsy heart attack. When you see the commercial on TV, you think very dramatic. My one because I had the procedure, maybe a nerve was touched, so I had weakness, I couldn't really explain it properly. And it looked like maybe a nerve was pulled or touched. I found that my stroke looked like something else. It wasn't, it just looked like a stroke. I wasn't really believed. And then I went to hospital like a couple… I called the ambulance a couple of weeks after the procedure. And then…

Researcher
What was the reason for calling the ambulance?

Participant
Oh, cause I still felt I still had the weakness. But my family couldn't really see it. But I could feel like you know, my little, because I had a left sided body stroke right in the head and my arm was lifted not properly, but because the stroke was on the left, you're still getting confused. So called an ambulance. And because my doctor didn't really want to know, because they were like, you've had to stay in, and I did call the hospital where I had my heart thing done. And they still for always to do with the procedure. I called the ambulance and they said, oh, like my issues. Everything was clear.

Researcher
What was there? Any other other symptoms or signs of stress was there?

Participant
Yeah, so my left side was heavy. I felt like it was dragging a little bit like I've, and also like, I felt at the time that it was going towards the floor, like it kind of bends down the floor. I don't know if that's to do with gravity and it can't, and it also wants to bend into the center. But the thing is that these are not that obvious, and I could always like my swallowing, talking, all of that was fine for me. It wasn't a dramatic stroke. I think that's why got myth, but now I'm more educated. That stroke doesn't have to be dramatic and that somebody will have it in the night and awake. I will pay more attention next time. But I think because I just had the procedure, to actually not do, and they said that I'm quite lucky because it happened because the procedure like I don't have to see, like, you know, it's not I don't have any pre-stroke, like, anything that could make it happen again. So hopefully it was because of the procedure. I wasn't predisposed.

Researcher
And tell me after you arrive to the hospital, how long you stayed in the acute care?

Participant
Yes, that day with an ambulance again, they didn't want to even take me to the hospital. They were like, Oh, we think he's just a touched nerve. He was to hold it on my body. And so and obviously, you know, I'm not a doctor, but they were like, you can come if you want to. So I went with them for like, seven, eight hours. And finally, while at the end, they did a brain scan. That's when they saw the swelling. And then first, they said it was a tumor, or… I said, it doesn't really make sense, because I've had the procedure two weeks ago, and afterwards, people told me that left sided weakness, that dramatic is not really because of a tumor.
They were basically trying to say always because of the cause of a mass or, I was a bit of a confusing time. But they took me in, they gave me steroids to get the information down. And then we did an MRI within a couple of days. And that's when they realised it was a stroke. And then from the hospital, local, they took me to the stroke like that. And I was there for about five days. It was a bit weird, because obviously like…

Researcher
Participant did they tell you it was like bleeding or is had it?

Participant
It was an ischaemic, it was a clot. It was right -sided. They said at the time, it was like an infarct stroke. But I have had some eye damage on the left so my GP has included octuple…? And they basically said posterior circulation stroke. At the moment, I have all these different words. Because I remember at the time, I was getting a little bit confused. Luckily, they spoke to my sisters on the phone. Because obviously, I wasn't allowed any visitors on it.

Researcher
Yeah, this is my next question is just to see, during that time of staying at the acute stroke unit, how was the situation with COVID? And what do you have received in terms of care? Tell me the situation during that time.

Participant
Oh, I think was like it. I mean, they didn't make me wear a mask or anything. I did ask them in the beginning, like when they were coming because they come in and out of the room. I was like, Do you want me to wear masks, but that's fine. But obviously, they're always wearing a mask. So you can't really connect as much. But I do find like, because I wasn't allowed any, I couldn't have any family came, they couldn't even leave anything at the door for me.

Researcher
Like if somebody from your family brought something to you, they won't allow it to be like…

Participant
Not on the ward door, it was only on the main door, but I didn't really trust that the main door would get to me. Before I left for the hospital I made sure I had everything, like, hairbrush, all of that stuff. And I feel like it was a bit alien because obviously everyone's wearing full PPE all the time. It makes you think that you're quite sick when obviously it was a stroke, but I wasn't infectious.

Researcher
Did you think that affected your care by any means, like the level of care that you should receive?

Participant
I think the care was good. But I do think that they wanted to get me out of there quickly because I'd already had my stroke and because we pretty much, for it was because of the procedure. So I don't know if COVID specifically affected it. But I know it is seems like sometimes, you know when they're going around like as if it was just like, oh, like I'm just another job. And he didn't seem so personal. Obviously they like stressed

out as well. So, because I know that when my sister had to call and the doctor was surprised that I didn't fully understand. I still had this swelling on my head, you know, it was a bit difficult to understand everything. And I feel like they didn't want to give you as much time. And that might be because of COVID as well because they don't want to spend so long in the room with the patient. I think that might, yes, I fell a little bit. Because I was quite lucky. obviously, I can talk, I can walk, I can do like big main things.

Researcher
I want to ask just a little bit. How did the stroke affect your physical, like ability, or maybe doing your daily activities? If you can just tell me that?

Participant
Yeah. So I think like, because it was right side head, left side body, and I'm left handed, and I am a dental nurse. So the area I work in is very small. And I have to be very accurate. I work with XXXX, I have to be very accurate, obviously. So I'm not back to work at the moment. They're not letting me back until I've had my first drug review, which is not till the end of November.

Researcher
Yeah. But now from what I'm seeing, like, your hand movement, you are moving, like, I think you have got a good recovery, comparing to other people who have got a stroke, so I think you were lucky to have just a minor thing. Maybe I forget. But tell me how was the your situation from the beginning? Was it like this or has it improved?

Participant
Oh, no, I have confused in the beginning. Yeah, I was getting confused. And like, I was forgetting like for dinner, I mean, it's only small things, but if I have like, ketchup, I bring it in twice or bring water twice, that just made me laugh. And I lost the garden door key before. And I think I, and I remember, I felt like my sisters, had my sister as a child, and the climb was in front of the stairs. I fell, instead of getting out of the way like, well, I fell a couple of times in July when I was only in hospital for I think nine days. And in July, and even August, I was falling a little bit and getting a little bit confused. And also, like when I was going outside, my hearing, there was a noise, I could know where they were, but the doctor said that is no more. And I do have some body weakness. Like I've had a physio come to the house, and they did you know the tests. I pushed, the left is quite, mine is more sensation based. The left is quite a bit weaker.

Researcher
But other like your body movement, working, was it affected by stroke? Or they were okay?

Participant
So I feel more fatigued and again, more tired. And because I have a left visual field deficit, so I have to be more aware when I'm outside. So it has changed my normal habits because I used to wear headphones, loud loud music, not even looking, you know, cuz you know, that's how you are, you know, I'm very careful with my like, make sure I look. They said I'm not allowed to drive. But I don't drive anyway, to be fair. But I made sure across the road properly and stuff. But my left side, he tries to go down to the ground is still doing that. On my left leg, lift up, like now I'm using a lot of energy just to keep it in a relaxed position. So I know that when I go back to work, and I'm sitting chair, and I'm doing my jobs, and might try and lift up stuff. But I think it might happen more than I'm tired because I went out with friends like a few weeks ago, and then my left leg was dragging like I don't know what we call it, dragging.

Researcher
I think maybe that's because of weaknesses in your muscles, but are you using any form of assistive devices or something when you are walking?

Participant

I'm not when I'm walking. I've been lucky, only when I get in and out of the bath because my brother had an accident a few years ago. The council put in these things like rails and the banister, you know on the stairs they put two banister. So when I got down to, say, I use both and I make sure I'm going slowly. I did fall over before, I'm fine because it's very low. I never realised how low the bath was. Now I kind of twist backwards when I get up. I used the wall and like you know the handle on the wall again.

Researcher
Yeah, a little go back to the time you spent in the hospital during the in-patient. And when you moved out to your house, tell me what sort of care have you been receiving since then? Like, what you have been receiving in terms of care from NHS and when moved house from the community services? Tell me, what did you receive?

Participant
So I had, I've had quite a few appointments like, because obviously, I'm still under heart care. So from the stroke side, I haven't really had anything from the hospital.

Researcher
When you were in-patient during the time where you stayed there?

Participant
So as a patient in stroke, the doctors came to see me every day, just to give me an update. So they did an MRI that first day that confirm. And then they did, like an echo, you know, Echo. Because they still want it because they want to. They were trying to work out why the stroke happened. I feel like they were trying to do like a blame game. Because I just had to send to you. And they said, like, we had a stroke. They said that they can't say 100%. It was because of the strong cause of defense. Probably and because they are two different hospital. They weren't communicating properly. So yeah, I had a MDT meeting, you know, the MDT on the last day I left basically in the hospital, they just, there wasn't much care that they just like came to check. Tell me, you know, update me. And I had a sack of physiotherapist come. But she just did those tests, you know, where they tell you there's a heart and finished heart shape, and can you read these words, they were very basic tests, I had to keep telling them about the eye because the eye is only slight, still notice. And had to really push for that referral.

Researcher
So the physical therapists were fighting with any sort of exercise or maybe training for your walking or any other activities?

Participant
So they said, because I'm left, I'm very left handed as well. Make sure you're using the left, because in the beginning, it was easier to use the right. I find it hard to like brush teeth, that because I am very left - handed. So they would just say make sure you keep using the stroke side. But I had to really ask to get some exercises, then they gave me a sheet of exercises. But I had to be really ask for that. And she was just, you know, like the hand like this.

Researcher
It wasn't too maybe enough for you. Like, did you feel that was enough for you? Or you maybe wanted or maybe needed more of physiotherapy?

Participant
For me it was because mine is more sensation based. So I think it might just be that it might go with time. I'm not sure, like, just because at the time, I was a little bit annoyed. When I looked around the ward, there were people in a lot worse ways. So yes, I do understand, you know, and the fact that I was only there for a few days is a good thing. Because I can go. In humidity, you can see the ward for your family's bit better. I

do understand, but at the time, I was like, this is a bit weird, you know, that I had to really ask for the piece of paper. And, and like, because at the moment, like I do have, so I can't really clean myself properly with the left, like, toilet cleaning. My sense, is a little bit embarrassing, but I just used that other hand, obviously. But you know, because I had to kind of learn to do that. Like, you know, there have been like impacts for me. But I think you know, what is more about a tick box exercise? We've seen that patient, tick, we've told…

Researcher
If you think also about other needs, like you need to during that time other than physiotherapy and rehabilitation, what were your needs during that time?

Participant
When would it be nice if they let me see my family because, obviously, one minute the thing I've got a tumor in. And also when I left the hospital, they still thought there might be a tumor. My situation was a bit confusing. I mean, now it's confirmed now that was to do with the stroke. And because I work in a hospital, but I can't access my file because it's a different hospital. For me, it's a little bit different because I was like, Oh, if it was my hospital, I could…

Researcher
see what's going on.

Participant
Exactly, because now I have to be if I want the MRI, I'm gonna have to request in fourteen days, all of this rubbish. So it was a bit difficult for me to be patient because I though because obviously I work in dental, hospital dental we do deal with cancer stuff. I deal with people that I want pegs and, you know, like we deal with CV stuff. So, but it was a bit annoying, but I do feel like at the time yet it was more like, you know, okay, have you spoken to a doctor, you know, but I do understand now because obviously the rehab starts. And like, and also I think at the time I, there was some stuff that I didn't notice like, yeah, even eating, I eat a bit slower. Because there is a Boston facial like it wants to go like this a lot. And I didn't notice that in the hospital because I think, because obviously they're coming every hour, check your BP, food. Don't get much time to think, for now, so you know, it's a bit different now.

Researcher
Yeah. Can you think of any other means or we should move to next question?

Participant
I think it was just that. Yeah. Yeah.

Ahma
Okay, so sent me to the time when you discharged from hospital to move to your home again. I wouldn't catch that, after maybe five days?

Participant
So I was in-patient on the first. But that was the normal bit of those. I went to the stroke bit on the fifth. And then I left on the ninth of the stroke which I was in hospital eight, nine days.

Researcher
Yeah okay, so you're discharged after nine days? Tell me at the time of discharge from hospital, what have you received in terms of maybe advice for?

Participant
Yeah, I mean, that day, it was very weird because, like, they said to me, Oh, we're gonna do the MDT meeting because I went to Queens Hospital in Watford. But my scan was done at Bart's in London, that

didn't disappear. The team meeting with them on the Friday, they said, We will probably send you home. But you know, so then we decided that there's no point me staying because, you know, my, almost stuff is going to be in community, if that makes sense. They didn't really tell me anything at that time. Like what was gonna happen? And it was really weird because when I left the ward, like, before, when I was trying to walk around the ward, they wouldn't even let me walk there. It was like over the top. But that day, I didn't, none ever took me to the door. They let me leave, and I had to carry all my bags. Because when I bought I bought too much stuff. I know, like, my family sent some stuff for me like, yeah, I don't know what age your family like they said. I thought a little be like, Okay, now they don't care. Because, like, they just didn't even send me with anyone. Oh, let me go to the and you know, because the COVID, my dad and my sister were outside, but you can't come in, you're not even allowed to come in to get me was like, oh, this is so weird.

Researcher
This is a shame on them. I don't know why they're treating people like this. I wanted to know, have they taught you anything? Like when you go, when you move home, how to work safely? Or maybe how to use toilet safely? Or maybe how to do your stuff

Participant
No, so they haven't done anything like that. I mean, I had a lot of appointments after for the heart thing. And I have another MRI.

Researcher
I mean, from rehabilitation side, from like, physical therapists, or nursing or…

Participant
I'm still on the waiting list for the hospital. I've got like an orthopedic because of the bones. So yes. They said that was a 12 week waiting list. And the doctor put me on that on July. He was after the hospital. They haven't really done much of rehab. I have had to chase it up. So I got in touch. I know I think the stroke. So you know the Stroke Association, they got in touch with me locally, and then they set me up with physio, so from the hospital.

Researcher
This is after your discharge? This is when you are at your home after discharge? Stroke Association sent you with somebody to maybe carrying out your therapy at home?

Participant
Yeah, when I left, I told you I got the sheet, I had to ask for that sheet, but now when I think about it make sense, because other people in the ward were in a very bad way. But obviously, I still am not 100%, so like, I still needed help. But I think sometimes when you need a little bit of help, they don't help so much like, you know, because mine was not so dramatic. Like, no, I can talk.

Researcher
Did they visit you at home after you discharged?

Participant
No no, not the hospital hasn't visited me at all. I think I'm on the physio waiting list with the doctor. So we've, I'm on a physio waitlist and now an orthopedic. But these are things that I had to chase with a doctor, if I made sure that I got a GP appointment.

Researcher
How long have you been on waiting lists now?

Participant

July, basically since end of July. But what I did was because the Stroke Association contacted me, then they sent me physio. So I've been lucky at the Stroke Association, they sent me one guy that came twice, he gave me exercises. I think that's called grasp, you know that grasping…

Researcher
How do you think that is going like helpful for you?

Participant
Yeah, I've been doing it a little bit. And it's good because I, before the physio came, I didn't realise that my, kind of hand movement, is not so good. Like, because he checked in and like, obviously, it's moving now, but the fine movement is not so good. That's what he was saying anyway, because my job is like, into dental. Like, I didn't think like, cause I was thinking, oh, I can drink. I can open like, stuff. You know…

Researcher
From my understanding, maybe this is your main goal of getting rehabilitation after stroke, right?

Participant
Yeah yeah

Researcher
So if I can ask you, what is your main goal of recovering after stroke? What would be your, your main goal?

Participant
The main goal? I mean, I would like the sensations to go if they could, because at the moment, is that left side is it feels very, like it's pulling down. I mean, it's trying to go into like this. And you know, like fetal, you know, fetal like baby like that. But I know that's a protective thing. Because I'm a bit worried when I go back to work, what I'm going to look like, my face looks okay, like it's not dropped or anything. This side, it does go down. And I always say I'm a little bit worried about that, because I've got peripheral vision, a deficit because of stroke.

Researcher
Have you received care for that, or are you still on waiting list?

Participant
Yeah, I had an appointment in July. And they did, you know, like when you follow the eye, all of these kinds of tests. And then they said that it's an upper, is like on the left side, but more upper left. And they said, I can't drive. Obviously, I wasn't going to learn. But that does show you that there is something there. And they said that might get better, might get worse or stay the same. I'm young so…

Researcher
Yeah, hopefully, you recover for that. Yeah.

Participant
And they gonna see me again in July… sorry not July, January, January.

Researcher
Okay. Hopefully by that time you will be fully recovered for that. So now, let us move to another question. If you know anyone who have had the same experience, or maybe similar, like any, anyone will get a stroke during that time. And do you know anyone have had that?

Participant

I don't know. There's this Facebook group called different strokes. I probably got mentioned to you, because that's where this research was mentioned. I found that like a really good resource. Because obviously, that being an Asian and stuff, like, I do have older relatives that have gotten this disease and that disease, they have everything by…

Researcher
I'm talking about the management of stroke during COVID, because of COVID. If you, anyone who have received the other way of help, maybe from some association or organisation, or maybe from NHS, if somebody has different experience.

Participant
Yeah. So only from the group from different strokes that they, and I know a few them now, and I know that they said that some of them have had, like they're in a real bad way, they got no contact. But I think that you have to be a little bit proactive as well, obviously, like, I can talk and stuff so it'd be easy for me. But some of the stories on there have been quite negative. Then, you know, there are people that have a lot more clear needs. So but yeah, so they've had like worse treatment, I would say.

Researcher
So. What have you received from the therapist who came from Stroke Association? Can you tell me further? Yes. just summarise what they have given to you.

Participant
So they gave me the gross, the gross sheet, you know? And first they checked me and like I did the test. So it was good, because they confirmed and legitimise my feelings. there was an issue with the left. And they gave me the sheets, some kind of yoga.

Researcher
Are they visiting you regularly, or it was only one session?

Participant
So I had one person came twice just for monitoring. And then one person came four times to watch me do stuff. And then they've just finished, and then tomorrow I have an occupational therapist coming, which I'm not sure

Researcher
How often the traditional therapist come?

Participant
It's the first time she's coming to me. I'm not sure. She's gonna come, but she said that she's a bit different so I was getting confused with the physio

Researcher
No, she is like, fine thing, how to do hand functions.

Participant
Because the first guy, the main physio said that he's gonna send me for cognitive referral, and because my memory is not great. And also he was going to send me for fine motor skills referral, as you said, obviously, I need that for my job. But the gross stuff is like, you know, the flipping, and all of that, the fine motor. And also because I work at the hospital, I went through that as staff, we call it occupational health. And I talked to a physio from the hospital yesterday, they gave me that gross sheet. I made sure I do that more. And they've, they're gonna refer me to occupational therapy as well. I thought it'd be better to do it in the hospital, coz the managers as well.

Researcher
Well good. Hopefully, that will go smoothly with you. Just to let you know, because Zoom meeting allow me for 40 minutes meeting. And then after 40 minutes, we might disconnect. But you can use the same link just to join again, just to let you know, okay. And if we finish by before, I don't think we will finish because we still need to do assessment after that. I just have a few more questions to go and then finish.

Um, there is something called self-management. This is like a concept in the rehabilitatiom after stroke, where we encourage people to do their care on their own, not to be independently doing that. But instead of having therapists working 24/7 with you, or supervising your therapy all the time, then you can do as much as you can of managing your activities at home, your therapy. Doing your exercise may vary according what you have done so far. So I'm just asking, have you engaged in any of self-management activities since you moved from the hospital?

Participant
Um, so I did. I haven't really heard of self-management, but I guess like them giving me the exercises to do it myself.

Researcher
Yes yes, like, teach you what to do. Yeah, maybe before that, they might involve you in setting some goals of your rehabilitation.

Participant
So I haven’t been setting any goals but I have I've been doing exercises myself and I looked on YouTube.

Researcher
Yeah, this is great thing. I mean, I want to know if you received anything from NHS staff, before your discharge.

Participant
No not really, because I had to really ask for the physio exercises, I put the gross stuff now but that's only this week, I got given nothing from the hospital now.

Researcher
How about the therapists who came from the Stroke Association? Have they told you anything about this?

Participant
Stroke Association they gave me the gross list to do it myself, so they gave me stuff. Yeah.

Researcher
You know, I mean, did they tell you anything about self-management? Or how you might do that?

Participant
So what are they trying to do is like an hour of exercise a day, to do it myself. Yeah.

Researcher
Did they ask you what's your goals that you really wanted to achieve?

Participant
Goals? I think because mine is like, it's not like big, big effect. That I think that they have a bit you know, but I'm not even back at work, like, I mean, they haven't set any goals for me, not the NHS, but my work has, you know, been communicating with the work obviously.

Researcher
But as you share, as someone who is working medical, and also you are maybe well educated. So you said you did self management without even somebody taught you that. So you have been looking for, maybe YouTube and other resources, how to manage your stroke and all these things. So tell me how did you find that? Like doing your own thing? Or your self-management? You are self-managing your situation?

Participant
Yeah, I mean, like, there's a lot of resources online. And then there is some stuff that is obviously not going to be relevant. But I tried a little bit like that stretching and all of that would be good. So I'll say some days, I'm really good. And some days I get really tired, and I don't do anything.

Researcher
This is okay, this is okay with everybody. I think everybody will have this.

Participant
But the thing is that I am a little bit like that anyway, like, sometimes, I'm like, Oh, I do loads that I don't like, I'm a very up and down kind of person. So I need to be a little bit more in the middle like that. So I have to change some of my own habits, because I did join the gym again. And then it was saying, like, if they said, like, another gym is good, but I need to be doing like smaller stuff. Like, I thought the gym is easy, you know, the rowing machine, you know, like, it's not so boring. Because I got bored easily, that's why, with my exercises, I don't look at the sheet, I follow a video line, the lady does it and I followed her. Because otherwise, she's so boring looking at the sheet. I think like for me, like, I feel like that's what they don't think about in a hospital, you know, and you kind of get left in the community. And I think, obviously, with a pandemic is even worse, because I can't even see the doctor. And like, when I spoke to the doctor, he was just like, oh, just to the physios, you know, you have to be motivated. And at the moment at the Stroke Association, they're not doing meetings yet. Like, it would be nice, you know, if it wasn't a pandemic, I could meet local people that have had the same thing. So you know, it's been a bit unique having a stroke in the pandemic, but then at the same time, I've never had a stroke before. So I've been quite lucky because you can, there's loads of things like that. So I've been in the beginning when I was I felt kind of like I was left to it. I was watching like a follow up exercise on YouTube and stuff. And like I said, that charity, different strokes, they really did, because they have like physical therapists doing it, you know, that is the right thing. It's not like a fitness trainer.

Researcher
Well, this is good. Actually let us go to the last question, so the last question, I just want to know if the situation with COVID goes to normal and I'm assuming that it is going now to normal, what would you see as priorities for your rehabilitation?

Participant
Like if it was normal time?

Researcher
Yes. Yeah.

Participant
I think, then because I think the hospital would be more in touch, then. I think maybe when I left, it would have been more of a plan put in place. I think that they kind of rushing me out. When you know, like, because mine was a bit weird, because they were, they would, they didn't want to, like, admit that it happened because of the stroke so I think there would have been more communication. And even when my, when I was feeling unwell from the stem, I think it might have been taken more seriously. Because, you know, because they, but now it's kind of like, they just want to get you in and out very quickly. And like,

when I was calling the line, I called the helpline when I had the problems, and they were like, oh, you know, it sounds like a… And the same with the GP. But if it wasn't a pandemic, you know, they would have been able to see me and they would have seen that I was a bit more, you know, I think that might be the stroke may be may not even have happened. Rehabilitation, was I'm not sure if it would have been different because it's still just wouldn't be more in touch.

Researcher
Maybe, maybe they need to carry out more of intensive, maybe a therapy for patient after stroke.

Participant
I think for me, I feel especially during the in-patient, definitely that time as the in -patient, that physiotherapist only saw me one time. And then I had to watch, she come to say, she waved every morning and said hello, came to see me one time when she saw like, as I said, we did the heart thing. And, in words, I can read words. But yeah, and would it be more like that, rather than I'll just to get me out of it.

Researcher
Any other priorities that you will set for your rehabilitation?

Participant
My priorities, I should be doing more exercises like, and I want to go, I want to do more gym stuff. And I want to go back to work, obviously. So I need to do more like fine motor movement, like small movement.

Researcher
And maybe just maybe, maybe to get more prepared when you are moving home. Instead of just moving on?

Participant
Yeah, definitely because I feel like at the time of discharge, I was just, I left to it. And like, I did have like, I was positive in the beginning. But then like, you know what, because I never been in in-patient as well, like, I never had a procedure. It was always, it was all like a lot of new for me. So it was that. And then I couldn't, my family couldn't come to see me. And then like, I had to really ask the doctor to talk to my sister, because I was getting a bit confused. And I remember I tried to write notes. They will…

Researcher
But yeah, let me ask you a question. Let's say, if the pandemic extended for another time, if we experience another, I'm hopefully not, but I just think that we, the way you're thinking that the services should be tailored for people with stroke. Like, how the services can be tailored for people with stroke?

Participant
I think even if you just, like, calling you, check that you are okay, because he might not always, like, ring, and, you know, reach out just to check that you're okay. Because I feel like because obviously, I'm quite lucky not to use computers, like I'm very modern, but if I was like an older person, I'd say like you would be a little bit lost in the community? I would say Stroke Association has been really good for me.

Researcher
This is at the time when you are moved to the community. So you're maybe…

Participant
Yeah, at the moment, the Stroke Association is the charity, not NHS. So even my doctor like, has a lot of patients, but how many patients had a stroke in June, it can't be that many.

Researcher

For easy communication, they just have maybe a phone call or maybe a Zoom meeting, whatever, so it's very easy maybe for them.

Participant
Because it's not like, coz I know stroke is a bit weird, because obviously it's happened. So it's not like oh, I'm at risk of it happening but the before and after effects. So they can check on people more. Yeah, because I do feel like I'm flattered. And then it's become a bit lazy. You're like, I know, I'm bored. Like, I mean, and that's kind of, I'm a little bit like that anyway. I try to keep a check on it. You get a little bit your mind gets tired, you know, as well. Um, and I felt like all I hope it's been like the Stroke Association and the different strokes. And that's all like charity stuff that's not NHS funded. It's a bit sad. But obviously, because I've not had a stroke, I didn't know what the services were like in this area. So also, I'm lucky like, you know, the physio came to the house and stuff. But it was good to see the physio because he said, like, you can see there is a difference. I'm not making it up in my head, you know, because my foot was dragging that side was going down. And it was really good to have someone explain that or that is normal. Like, because they said it's like a protective thing to go. Yeah, you know, I'm not going crazy. You know? Because I remember in the beginning, when I, after the the heart procedure, like none was believing, then the other issues. You know, it's kind of nice to have someone believe if that makes sense.

Researcher
Yeah yeah, well, thank you very much for signing up for this. I'm really interested in hearing your story and having your thoughts. I just want to ask you, before we go to assessment, if you want to add anything about your experience with your stroke.

Participant
I mean, that's pretty much all I would say, like, obviously, I don't wish on anyone, but I'm trying to be more positive, obviously. I think that there needs to be more education in the public, though. Because, for me, like, even though I'm a dental nurse, is tend to, it's not medical. I do work in a hospital. So I am very aware of, like, heart attack? But not so much stroke. And like, you know, because I had the procedures, that was all a bit confused. I feel like I miss signs as well. And as well as the hospital missing signs. So I think maybe the needs to be a little bit more education on there. But definitely, yeah, the pandemic did affect the services quite a bit. And there needs to be more contact in the community after then, you know, like, my appointment is going to be in November with the stroke people on Zoom. They might say, oh, you know, I've got worse, got better, but it needs to be the community connection. Yeah, yeah. That's it and I'm really glad to doing the research.

Researcher
Thank you. So I'm gonna stop the recording.

Researcher
I want you just to confirm your name and consent for the recording, please. You can just say that.

Participant
Yeah, it's Participant McLaughlin. I'm happy that the meeting is recorded for the purposes you need to use it for.

Researcher
Alright. Thank you very much, Participant, for your time today. And as you might know, from the information sheet, we are only interested in your thoughts and your experience of having stroke during the pandemic, and how was your physical rehabilitation during that time. There is no wrong or true answer, just feel free to explain what is your thoughts or, you know, ideas that you have. We are happy to have that. You can stop your interview at any time if you feel tired, if you are not interested anymore. It is your right,

if you want to stop at any time, just let me know. And if you need to take a rest, let me know. We're happy to do that. So it is your right for you. So don't worry at all.

Participant
Okay.

Researcher
Thank you once again for your time, we're going to start this interview. So please, first tell me about your experience with the stroke and how it was at the beginning.

Participant
So I had my stroke pretty much exactly a year ago, so I was admitted on the 17th October to XXXX hospital. I didn't quite, I was probably, looking back should have gone a bit early, I didn't recognise the symptoms until it was a bit later. But it took me on the blue lights and, you know, aside from the sort of going through the bit in… any… which was very good, actually. And the doctors knew exactly what was happening and were experienced and understanding that, you know, the symptoms and what I was presenting. And then they had one night where they moved me up onto the high dependency unit, I was quite fortunate because then the next day, they moved me down to the stroke ward, which had literally just been just been opened on, I think Friday, so they just opened it after they just had a big COVID outbreak and I had to go to the cleaning. So yeah, so luckily, when I went to get down there, it was all okay. I was monitored in there through till Wednesday, I think about the Wednesday. And then as soon as I got on, got in there, the guys on the ward, because I knew it might take a while, put me in for a transfer to the rehab center in my local hospital.

Researcher
Yeah. And what was it, sorry, was it ischemic or a bleeding stroke at that time?

Participant
It was ischemic. So I had the… I think I had a CT scan on the Saturday. I still don't understand how I…how I had the MRI. MRI was taking place on Monday. So by the time I had the MRI, because it was a brainstem, so they knew I'd had a stroke, but they couldn't see it until they had the MRI scan.

Researcher
But before that, given, have you had any experience with cardiovascular issues before that? Or this was like all of a sudden?

Participant
Yeah, no, this was all of a sudden. So I had no symptoms prior to, you know, no symptoms, no risk at all. I mean, the any, any bizarre thing why I thought it couldn't be a stroke, although I kind of knew the symptoms was, it was statistically I went well, you know, one, I'm very healthy and two it's bizarre, my aunt's have literally had a stroke on a Wednesday, you know, and it was the, you know, statistically what's the chances of two members of the same family having a stroke within a week. Particularly, when I put my risk rating on stroke and CP is less, well, is less than 1%. And was well. And was then so yeah, you know, it's, it's just one of those things. So they woke me up, they did all the tests that they booked me in for a lot of the stuffs. And as I said, they literally straightaway, as soon as they got in there, they put a request in to move me into a community- based rehab, multidisciplinary team. And it was by luck that literally a place came up in that hospital on the Wednesday. So by the Wednesday, they'd done all the tests that they wanted to do to make sure I was stable. Other than that bubble echo, which was the only thing that was outstanding. And yes, so they moved me out to the rehab unit.

Researcher
So how long did you stay in the acute room? Stroke acute?

Participant
So I was in stroke acute from the Sunday to the Wednesday.

Researcher
All right. Wasn’t that long. And then after you move to the rehabilitation, hospital or ward, what was the impact of stroke on your daily activities and physical abilities at that time? Like, what did you notice new with you after stroke?

Participant
So yeah, so I lost the complete right hand side of me, so I think they just about got me out of bed before I left on the Wednesday, but, so I wasn't allowed to, you know, I was under support from from physios. Other than that I wasn't allowed to transfer to bed or walk. I couldn't walk at that time.

Researcher
At that time? Because from what I've seen, I think you are doing great now.

Participant
Yeah, no, no, at that time, I couldn't use this arm at all. You know, when I moved into the rehab, other than, you know, I had quite bad facial drop, and, all of those things. So, they set about a six week program, which I engage with, you know, intensively. And then sort of, I was one of those patients that if they, you know, their physio sessions are able to pop them in during the day wasn't enough. So I'd be asking, What can I do in my room? I was, I was quite lucky because I was moved into a side ward because COVID initially, but because of my age, because the ward are mix of stroke and Alzheimer's. He felt it was a little bit much to put me on the main ward, so left me on side ward.

Researcher
And is that consider as like NHS facility or a community facility?

Participant
It's NHS community.

Researcher
Okay, I see. All right. What was that Participant?

Participant
It's XXX Hospital.

Researcher
Okay. All right. Tell me when you're moved there. You said they moved you and decided to put you there for six weeks, right? During the six week, can you tell me what sort of therapy did you get from them in terms of maybe physical therapy and other therapies during that time?

Participant
So during that time, I got at least two hours a day physical therapy. Physiotherapy working mostly initially just working on my legs. Once I got up, then also working on my arm as well.

Researcher
Okay, and do you have that therapist working the whole two hours with you, or they just give you maybe an advice to do something and they leave?

Participant

No, no, the whole time. Sometimes it was with, there was a few senior physiotherapists and then a few trainees then a few health care professionals. So some of them might be, if someone like sit on the motor bed, motor medic, you know, often there'll be someone around but it could then be supervised by a healthcare system. But yeah, in most of the time, I had one-on-one with a physiotherapist. And then we also had, I think the one formal speech and language therapist session a week. Yeah, with a specialist. And then they gave what was being worked on to the patients or the physiotherapists. And some of the assistants would come and work on that on me as well. So I normally had, you know, 15, 20 minutes of that a day.

Researcher
Before that, what was the problem other than your mobility, you said you cannot move to chair at that time, what other impact of a stroke in your physical abilities other than transferring?

Participant
I had significant slurring, so the whole right hand side of my face drops as well. So that's why we had the speech and language therapist trying to rebuild up the muscles inside the face.

Researcher
Did you have any problem with vision?

Participant
No, I was lucky that vision wasn't affected. So for me, one of the reasons why I think I've recovered pretty quickly is it didn't affect my cognitive ability. So I was able to sort of override the the lack of brainstem, using my emotions by visualising and working on it.

Researcher
Yeah, alright. So tell me also about your therapy during the six weeks in-patients?

Participant
Yeah, so they put in place a therapy program for me and then I worked on it. And then as I recovered, they modified it accordingly. I was, to be honest, I think I was very fortunate because knowing how a lot of the NHS, once you're independent, they kind of send you home. Whereas, I managed to get myself probably independent within about three weeks but didn't they committed to a six-week program to me and they want to make, you know, really get on.

Researcher
Well, this is good. Yeah, to be honest, this is good, because I have heard from other participants in our study, they were discharged from the rehabilitation fairly fast, like they couldn't stay for maybe one or two weeks. But again, it depends maybe how, where you live, or where you received surfaces. So I think you were lucky to have six weeks.

Participant
I don't know whether it was any pressure on the beds. Certainly, probably because I was really engage with what they were trying to get me to do. I kind of wanted to encourage that. And, and I don't think it helped but I bought my coffee machine as well. (laughters). I think they took the view that they committed to a certain date, and that was in the diary, so therefore, their view, again, you know, work to deliver as much as we can within that time. And yeah, so I also had counseling as well during that period, there was a good psychiatrist. Mainly, I think her role was normally for people who have cognitive impairment working on that side. But she walked through, you know, walk through coping strategies and all the various stuff so we had a few conversations during the period so it was a very well and I know from other people haven't had a good experiences but actually, for me was okay. For me, I can't fault what… yeah, I mean… we're limited by certain things and equipments and availability. But that's, you know, was made up for by professionalism.

Researcher
This is good to know. If you can think during that time, what was your main challenge after stroke? If you can tell me like, during that time, what was the main challenge or the thing that you need? You felt you needed, maybe some therapy or care to deal with?

Participant
I think it was surely about getting that physical elements and trying to get them working. I always have that focus that, I don't know why, but I kind of knew that I would get it back if that makes sense? So what we kind of worked on was, and it was a joint thing, it wasn't which, which is another really good thing about therapy that I had was, you know, we worked on lots of things together. And, you know, I would say, Well, what about this? And then we changed it slightly. And then we did. Because I had a lot of strength, certainly on my left hand side still, they were able to push me into doing, you know, certain things that they wouldn't even consider doing with other patients, so we did getting up off the ground, in a case you fell over and you know, as I said, we're walking over rough ground and walking outside. Yeah, soon as I was able to, with a stick and without stick and that sort of thing. And they were quite good as well, because, as I got more able, they negotiated with the outpatients, physios downstairs so I was able to use their facilities.

Researcher
Yeah while you were in-patient at that time?

Participant
Yes. Yeah.

Researcher
Yeah, this is good. Participant I want you to tell me just in general, not just about your mobility during that time, talk about your challenges, about your needs at that time. And if you feel the therapy or the care that you received, has fulfilled the needs that you needed at that time.

Participant
Yeah, I mean, it was the team was really good as well, in terms of, you know, getting the independent washing, cleaning, going to the loo. It was more the nursing staff worked with that, and, but it was a kind of, as I got more independent, you know, that I was able to use, and I think they're sort of, I did certain things myself, but I did it within conversations and saying, Well, I'm going to do this, and this is how I'm going to do it. They were trusting me to do that kind of thing. The other thing, they made a good assessment, or I've heard other people, they wouldn't let me home until they knew that the most have been done in my house to be safe, on the stairs. So it, you know, slightly frustrating. And the other thing we had a good discussion with normally on that kind of program, once they've got the house safe, and they believe you're coming towards the end, they'd probably let you go home for weekends but I knew if I did that, then obviously I lose four days of rehab, because I've had to go into isolation while I do another PCR test.

Researcher
Well, let us move on for now, since you're talking about the isolation and this thing, what do you think was the impact of COVID restrictions and measurements on your, like rehabilitation and services that you have received?

Participant
So it made, well, it made the whole six weeks quite lonely.

Researcher
They didn't allow your family or friends to visit you at that time?

Participant

In the six weeks, I had two visits, I was allowed to visit. So when I was in reading, there was no visit because of a man. And then they were allowing one visit a week when I first moved to Newbury. And then the second lockdown hit so we weren't allowing any visit which made it, you know, quite difficult. I mean, in some ways, I can look back and say, well, there was some benefits to that because instead of probably all my, some of my free time was getting to see my family and being with friends. I still did that using FaceTime and video links and stuff like that, but probably I have more time resting and more time where I could work on my own rehab than I would have done if you kind of got your friends and relatives there too.

Researcher
They won't allow you to be full time working. Yeah.

Participant
Yeah. So so, you know, I can see, you know, I wouldn't, I would rather they could have been there, whether there were benefits to them or not. And I looking back it's, it did make it kind of lonely, particularly at the weekends, when because you're not getting a therapy sessions, and it's a long time to kill when you…

Researcher
You said the therapy was five days a week, so the weekends you didn't receive anything?

Participant
No, no, no, but I am… what they did to once I, you know, by the latter stages of my, my time in there, they sort of kind of let me borrow a lot of the equipments to keep working on things over the weekend. Yes, I do it myself. So, yeah, it was standing joke, there was more exercise equipments in my room then their entire suite.

Researcher
So, yeah, give me about the other thing that affected your rehabilitation because of COVID, other than visiting of family and friends.

Participant
There was, I'm going to suppose that fear factor because at that stage, the vaccination wasn't around. So you, you kind of, you know, if anybody, you know, got it on the ward, they're always in the isolation rooms opposite to you, so you can always see what was going on. And there was always that worry that, you know, it's only six feet across the corridor. And was it gonna catch you but, and the other thing that was quite hard, as the second lockdown went in, there was a lot of, you know, having to live in a mask in the hospital, which, you know, the staff had to as well but sitting there doing it, doing your exercises with a mask on, you know, having to put a mask on every time anybody came in the room, that was, you know, that made it difficult, particularly with you know, it's not, not the nicest the things to constantly live in a mask for six weeks. But even worse, the worst one was the first night they didn't actually sort of went in through A&E got any mask, and then I didn't, wasn't allowed to remove it until I got on the stroke ward 24 hours later. They didn't make you take it off.

Researcher
That that is a long time. Yeah. Well, if you can just summarise your needs during that time and tell me, what were your needs during that time, while you are in-patient? Like, if you think back, you felt like you need this and this and this during that time? What would be your needs?

Participant
I think it would have been probably just a little bit better family contact time. I think that would have been the, you know, that's so important. And not just not just from me, but for my family because literally, you know, when my wife was away, when I had the stroke, oh, and my two sons, were here with me. I mean, they're teenagers. And then literally, they seen me carted away in the blue lights, and then that's it. The first

time I saw them was like, five days later for half an hour. Well, and then, you know, then again, the following week, so it's, you know, there's only so much you can do and express over a phone and sharing that information and talking about what's happened, what's the prognosis? What's, you know, is this the end of, you know, our normal life? Or is this something different?

Researcher
Was it allowed for you to call them and to maybe zooming to them?

Participant
It was that nurse, you know, the nurses and doctors are trying and call and explain. But as usual, you know, with hospitals sometimes that message gets garbled. And, and what necessarily, I get told isn't the same as what my wife has been told. When you're then trying to compare stories. When you're talking over the phone. I mean, it's lucky. I remember I didn't think about it, but the ambulance man who took me in, he just said, right, you got a phone. You got to charge for everything because you're going to need it.

Researcher
Participant, if you tell me not just about having family visiting or contacting you, but also other needs in terms of maybe your physical rehabilitation and other things. Like what were your needs during that time?

Participant
I think it was really access to the equipment, and those kinds of things that you needed to get there. And, you know, we had the occasional bit where certain of the equipments were because you know, that they're there, their equipment is on different wards or different levels, so we couldn't access certain of the gyms because they, they might have a COVID patient on those wards and there was restrictions on getting downstairs. But in general, the team seemed to find a way through it so it was that that was…

Researcher
maybe remember the main thing that you wanted at that time, maybe just to keep moving out to see some involvement with your, maybe functional abilities.

Participant
So that yeah, that was it. I mean, the main thing, or my focus was literally, you know, can I move my legs or lifestyle to move? Can I? Yeah, can I control it? And then oh, you know, feeling that my arm was coming back, and then yeah, you know, then trying to build it up to control that and do things.

Researcher
So this probably was your main goal of having rehabilitation after stroke, just to have feedback in terms of movement and moving your legs and your maybe arms and all these things?

Participant
Yeah, yeah. So I mean, that was my real, I was looking back, I was so focused on, you know, I've been put in there. I got a chance. It was really good that that was, you know, it's almost, I kind of viewed it as like a training camp, athletics training camp, you know, been put into isolation. Well, let's just focus on the one thing I can do then. And, and, you know, that and, you know, as a team, everything was there, you know, in terms of the food turned up. The support was there, everybody was happy and encouraging. Yeah. So, you know, I got everything I needed to make the best recovery.

Researcher
Yeah, during that in-patient.

Participant
Yeah, during that impatient and those six weeks, you know.

Researcher
Because you want to move to other stages of your rehabilitation, where you have to live at your home or in the community after the discharge from hospital. So I wanna you to tell me a little bit when the time to move home kind of came? And how they move you to live outside of the hospital? Yeah, back in your home.

Participant
Yeah, so our, my local unit, they were warning that the link, although the community team is, is the same team, in-patient,

Researcher
Okay, so they're also going to see you at home as well

Participant
Well yeah, they're one team, normally there's, there's community-based people, and then there's also in basically for the work in -patient people, but it is one team, effectively, but they were warning me that it was like, flaw, but there wasn't, the waiting list for that community-based support was long. And I, by the time when I went home, I probably would be left on my own for a long time, for four months. So, they did, which is quite as part of going like they…

Researcher
How often did they used to come?

Participant
So they didn't come… So, I was discharged in December and the first time they came was in April this year

Researcher
First time they visited you?

Participant
Yeah when I came up on the waiting list to be seen was April this year.

Researcher
Wow. And during that waiting time, what have you been receiving in terms of rehabilitation services?

Participant
So during that period, December I was that, so I was sent home with a program for working on my arms. So I…

Researcher
You were working alone or help with therapists?

Participant
On my own, on my own

Researcher
They taught you how to do the therapy?

Participant
Yeah yeah. I was an athletic coach so I… give me the exercises, as long as I can see what we are trying to get out of it I can work with them if that makes sense. So that they sent me on that. And then we tried to, they were very keen, I tried electrical stimulation. Well, I bought a unit because unfortunately, my health

authority, this is one of the negatives that doesn't support electrical stimulation, even though the team's really keen on it. So when I was in hospital, we bought a kit, I bought it on Amazon, and then they kind of watched me use it, and then they made the comments, you know, again, I looked up all the settings on the internet, and I know it. And then so I tried that. And then I privately, well, it was a NHS site. I didn’t want to wait around because it was to me, my recovery was more important.

Researcher
You did that on your own, but did the NHS staff help you with that? Or…

Participant
So they referred me down to that place. So the consultant, an introduction to that unit. And then I was able to give them a call and sorted out privately. And then I basically that, set me up in December. And then in January, I was getting concerned that it wasn't getting any support in the community. And, you know, I knew that I was doing things I was working, you know, and I was and was still improving, that was kind of bit. Well, I'm making all this up as I go along and seeing where I get to, it would be useful to have some coach or some guidance from somewhere.

Researcher
Did you ask for any help for that?

Participant
Yeah, at that stage, there wasn't anything from a community-based on the NHS. So yeah, I ended up biting the bullet and finding a private physio team.

Researcher
So you did pay for that?

Participant
Yes, I did pay for that.

Researcher
Okay. And how was it like? Do you think you have a lot of improvement because of that?

Participant
Definitely, definitely. The other team, the private team had, because they were in, they're based in the national jockey center. So although they'd shrunk down their operations a little bit, I was able to use all those services because they were open, because of them being elite sportsmen, so I was able to get in and use a lot the, you know, facilities that was, that weren't available through the NHS to do certain things. So that, that was quite good.

Researcher
But during that time, you will have to drop into their location to get your therapy. Are they… is it your house?

Participant
They would visit my house, Yes. But I kind of at that point, you know, I could walk with my, just about without a stick by then. And my son was living at home. So I took the view that I'd rather than pay them to come to my house and then not have the facilities available. I'd rather travel to them.

Researcher
So you can have the facilities and having a bit…

Participant
And have all the time. So yeah, when it's that's the one thing I…

Researcher
So that was your choice, probably not, like they are advising you to stay home.

Participant
Yeah, yeah. No, I definitely wanted to… It was, is funny, because a lot of the stuff on stroke and opinions stuff has been in the home, which is good in some ways, but it's kind of limited. Limited by what you got in the house or what space you got in the house. Whereas if you go, I suppose is different if you're housebound, and then I can understand it, but if you're not housebound, it's kind of better to go, you know, and maybe you're younger, it's probably better to go something.

Researcher
Yeah. What was given? Was there any issue because of COVID thing to attend your session, therapy session, or everything was okay?

Participant
I think we took the precautions, everything, as usual, as everything's, you know, sized on the mask and everything like that. Things got a bit more easy. The longer you knew them, the fact that, you know, once everybody got jabbed a bit and everybody relaxed, so yeah,

Researcher
yeah. Okay. So if you think about other people, maybe who were at the same ward, where you had your ambition to rehabilitation. If you think about them, did they have other way maybe to manage their life after stroke and to maybe have care by any other means? Or maybe they had the same background? Like what he did?

Participant
I think most other people on the wards talking to them before they were released. I can't imagine if they're under the community team, they would have anything else,

Researcher
they would have to wait probably for a long time for us to wait unless they are able to offer maybe a private physio or something.

Participant
If I look, probably, I know that the trust got some extra funding towards end of March, April time to put on more community programs and try and burn down the waiting list because they were worried about it. And then that's probably that's why I then got my eight week program. Yeah, in April and May.

Researcher
I'll just give you a notice, we might disconnect at any time. Because of the zoom, 40 minutes limit, but you can use the same link to call me back. Yeah. Sorry about that. Probably, I will up grade my account so I can have longer meetings. But now it just disconnect after for a minute and we have to call back. It will happen automatically and we just rejoin the meeting by the same link.

Participant
Yeah, no problem. So that when I had the community sport, I didn't give up my private stuff, either. So I was doing the same. And I was using the both to effectively give me an extra booster sessions through that period.

Researcher
So you did the private physios for eight, eight weeks, right?

Participant
No, no, I did… I'm still doing the private physio. So I did, there was a community base for the eight weeks. So I did my private and then it was kind of you establish a relationship with that person as well, so I didn't want to, to lose that and also it was the team that did it was very good, I got a lot of inputs from the, they used me for training sessions and things like this, so I got to see that top physios who are then you know…

(Zoom meeting reconnected)

Researcher
So from my understanding, you have had a time, I think in everywhere you're still receiving your private services, and also you received NHS community rehabilitation as well, so you did both, you work with both of them.

Participant
Yeah, so because the other thing was I could really only afford like one session a week, but not even really afford. But yeah. I couldn't afford more than one session a week with the private, and then, so, you know, getting a two extra sessions with, you know, different therapists, what in the community-based area was, was really useful. Yeah. And also, the other thing that really was important was the key part of the community package, there was, you know, for multi-disciplinary. So yeah, I had a chance to discussion on, I didn't need a lot have helped, but yeah, I had a session talking to speech and language therapist, where there was nobody else.

Researcher
And then the private services, you didn't have this team? Or maybe you just seen by physio.

Participant
Yeah. And the private it was all, what I asked to deal with was physio at that stage. And, the other thing was, which was really useful, although I didn't feel I needed it. My wife, that was because it was under this program, and having a psychologist and had some time assigned to that psychologist, she was able to work through my wife to get through some of the issues that she'd suffered from supporting me if that makes sense.

Researcher
Yeah, yeah. Yeah. Well, this is great.

Participant
Yeah, other than the gap and that sort of, once you get out into community, you're sort of left alone and you kind of feel a bit, sort of, I'd see if that makes sense. That it's, it's been, it was a pretty good program.

Researcher
Yeah. But given if you go back during the in-patient time, where they decided to discharge you to go home, I just want to ask about your preparation for to go home, did you think they prepare you well, to go home?

Participant
Um, they made the best? Yeah, I think they did the best they could. Yeah.

Researcher
You can tell me about thing that they did to prepare you.

Participant
So I think that, you know, though, they were very honest, they were very clear that it was going to feel a bit like I was left alone at home. We did talk about, you know, how I was going to work on the period from December up to Christmas, which you know, as partly that was, you know, they kind of wanted me to spend a bit of time just re-establishing myself, not necessarily, you know, still doing some of the physically, but learning to be in a family again, learning to engage with the friends and that sort of thing.

Researcher
Did they teach you how to do your, maybe therapeutic exercise at home? Or maybe how to work safely, how to maybe use toilet safely? Did they focus on that?

Participant
Yeah, I mean, the toilet safety and all of the safety, I'd already, you know, covered in the ward and there's no issue. Then when we had an OT visit just before I left, they discharged me when I went home, you went through all the, you know, all the stuff around the house to make sure that you know that was going to be safe. Yeah.

Researcher
Yeah. Then did they teach you anything about self management? There is a concept in the rehabilitation called self management where we empower people with stroke to do part of their daily activities, to do their exercises, to do their training with safety, so a therapist will teach you maybe how to do stuff at home with safety, if they introduce self-management to you, as a concept?

Participant
Not as a concept, I mean, that we, we discussed, you know, how I was going to do things, everything was based around how I could be, you know, safe and in myself. And you know, looking after himself how, how I could, one of the important things that, you know, they taught was like, the ability to get up from the floor, we did a lot of practice and things like that. So I could go out and, you know, push myself outside. So if I did fall, like, I wasn't stuck on the ground in…

Researcher
well, this is very important.

Participant
Yeah, yeah. No, it gives you that confidence. And then again, when, as my walking was improving, we kept looking for different terrain to walk on, so I was able to stable, you know, we did it in a nice, benign environment in the garden. But then when I was there, we're confident that was good. I then went, you know, into the rough grass around the hospital buildings, from going up and down.

Researcher
Yeah, but if you think back, because self-management also includes a wider picture of maybe involving you in setting goals for your rehabilitation, or maybe setting maybe some action planning, like what to do in terms of exercise, how much you do every day, where to go to do your exercise. Do you think they did involve you in this thing, like in making decision about therapy, and maybe asking about what are your goals of rehabilitation?

Participant
Yeah, 100%. You know, right from the early days towards even the ones recent setting and then also, although they've been write them down as formal goals, we had discussions how, how was going to take it for when I got home, what kind of things…

Researcher

Did they ask you what do you need for your rehabilitation? What are your goals that you are

reaching? Participant

Yes. Yeah. I mean, I always find that really hard in I get the concept. But as a stroke survivor, it's very much psychologically, you're still at the point, I want to get better. You know, I don't know, I get the concept of I want to learn to ride a bike, I want to learn to do this, I want to do that. But to me at that stage, I was still I want to be able to do everything I did before I had the stroke. Although I do understand that maybe some compromise, do you know what I mean?

Researcher
Yeah, yeah. But if you think about that, what do you think it was driving to engage you in the process? And they asked you What do you want to do for your rehabilitation and how you want to do, you think this was something that you felt encouraging and maybe a positive thing in your rehabilitation process?

Participant
Oh, definitely. Definitely. Because you know, one of the things I said I want to be, you know, do things and build things in, and that was part of the the team's work with me, was some of it was written down, some of it wasn't but it was definitely I know I've always had the experience the whole way through. There was never you can't do this. It was always this is you know, obviously you know, putting an element of realism into it.

Researcher
Yeah. Participant, I want you to think also. So it is good that you were informed by therapists and NHS staff in your rehabilitation process and the view for self management and engage you in setting goals and plans for your rehabilitation. But also I want you to think back, if you personally have set some goals for yourself or set maybe some plans to follow during that time above what you are receiving from therapist or what you advise to do.

Participant
Yeah, well, I kind of straight away, one of the things I decided to do is try and I want to say thank you for the team. So I set myself a goal when I was in there, or I just always done triathlon before I had a stroke. And when I was in there, one of the team was talking about it, I thought, well, you know, why not? Why not aim for that? Why not try and get it back and completely do a triathlon this summer. And so I then set myself goals of like, learning to ride a bike, or learning to swim again, learning to run again. So,

Researcher
so this is something that you set for yourself as goals.

Participant
Yes, yes. Yes. I mean, it was something I did discuss with the therapist as well. So but not straightaway. So yeah, we're already in my head before…

Researcher
You book them in your mind first and then you discuss it with…

Participant
Yeah, then I vocalise it later. Yeah. Once I can feel it's credible, if that makes sense.

Researcher
Yeah. Yeah. Good. And hopefully, you have achieved all of your, like, goals that you thought of.

Participant

I've managed to do everything that I've set myself to do. You know, and each one is, I kind of work on little things on like, so you've got the big goals like that but it's also like being in control. And just, you know, because one stage you can look at me and I'm, you know, 90% or 95% recovered. But at the other hand, I know, there are little things that aren't working as they did before working or twists. And it's all you know, it's trying to work. You know, I met with so little goals. Can I walk smoothly? Can I, you know, can I make sure that I brush my teeth from the shoulder? And those kinds of things. I kind of do to try and keep things going and keep things improving.

Researcher
So I don't want to take that much time, we promised it'll be one hour. So the last questions, and they are all about future care. Let us think when the health care surfaces go back to normal after we have had the pandemic for a while now, so what do you think would be your priorities for rehabilitation?

Participant
Um, after stroke I think my priorities. Personally, my priorities will be about getting mobile, getting active, and you know, the one thing I would love to have is, uh, you know, the ability to sort of go somewhere that didn't, you know, wasn't there wasn't an drop off. I mean, not necessarily going every week doing everything but there would be some, some way where you could just have that, either someone on the end of the phone or you know, maybe you've got an hour where you can drop in and go through exercises in the gym with someone that knows what you're going through and knows.

Researcher
So probably a communication with someone who understand your situation and can help with that.

Participant
Yeah, it's kind of like with NHS, you're either in rehab or you're not. And the reality on a journey from stroke as it is. It can be years in the way and it seems to be with the NHS. It's all very sectioned.

Researcher
Yeah. What else? Can you think of priorities at that time that as someone who have had a stroke during pandemic?

Participant
I think the other thing is, access to, is to I suppose this is about the right through care? My access to my GP? Nurse, she's been quite good, and have a lot of telephone consultations with her, but I've never actually seen her the stroke.

Researcher
So this is one of the things probably, if things go back to normal, you want to go and have physically attend that? GP?

Participant
Yeah, I'd like to know that there's, you know, normally there's meant to be a progress where you're under the care of the hospital, and then you get transferred back into the community GP. That kind of, and they're going to be looking after your needs. And thinking about is there anything else you need from rural station and refer you back or something, that kind of pathway. Because of COVID…So this is one of his, one of the ridiculous things I remember when I was trying to get my driving license back. And I discovered that DVLA said, well, as long as your medical professional, because I'd given it up because the consultants that I couldn't drive when I was first discharged. And when I was sort of about three or four months later, I started to feel like I was able to be in control of a car. And so DVLA said, you know, as long as your doctor says you're okay to drive, you can drive while you're waiting. You filled out your form, I found out the

doctor and she said, “Well, can you drive?” “Alright, I think so because that's fine.” “Oh, yeah.” She hasn't even seen me.

Researcher
They should see you before make a decision that…

Participant
I think well, yeah, I suppose there's probably more that they can actually tell from the fact from the way you're just talking. But yeah, I mean, I could have been bagging it.

Researcher
Yeah, yeah. And another way, Participant, I want you to think if you have your stroke during another pandemic, hopefully not. And hopefully, this is the last one. But let's just think what because one of the aims of our study is just to figure out what could be done better by NHS staff and other community services. So let's say if we have another pandemic, or maybe another lockdown, what could be done better by the services?

Participant
I think one of the things, I think, you know, which would have allowed a little bit more freedom and contact would be if the actual individual hospitals had on-site testing. On-site was by blood testing, so a lot of the delay was particularly when we were in the pandemic earlier on. It was you waiting for a result, waiting for PCR results, and as you got moved around wards, and the fact that I probably could have been released home, but for the fact that then, I then had to have a PCR test before I could come back on the ward. Now that's, you know, I get that sensible precautions. But actually, if you could have run a two hour test or a lateral flow to mitigate the risk or something like that. Then you would have been…

Researcher
Yeah, there will be. So if you are negative with the test, then you can receive the services and treat just as normal person.

Participant
Yeah, exactly. Yeah. I mean, particularly in reality on the ward, you know, the precautions are there, you're wearing a mask, we didn't really interact as patients that much, you know, going into the rehab work zones.

Researcher
And also, there's social distancing as well. So I think the professional cannot get closer to the patient, they have to stay, you know, so all these things I think you are raising very good suggestions.

Participant
Yeah. So that the ability to, you know, put the precautions in place, but test quickly so that you can clear the precautions out of the way. I mean, I suppose our biggest risk was, in the end, you know, because they were isolating people. Probably, you know, other than the fact that I'm going, the Delta variant wasn't around but I knew the most of the staff had all been, you know, the only real risk was the staff bringing in anything rather than anything else. It wasn't patient yet. They were the ones that were being tested.

Researcher
Well, yeah, what are also other things that you can think of, to do better if we have another lockdown, or maybe isolation?

Participant
I think it's certainly, from my perspective, how our IT systems, how are you going to engage and talk to the families and keep that family contact in place? It’s all very well, someone like me, who's very tech savvy,

working out, but even then it's like, it wouldn't have been good if, you know, the free WiFi on the wards was pretty awful. I found a way around it, I actually managed to sort of achieve it by switching my network onto unlimited so I could manage it that way. It's not necessarily for streaming everything, which I think the Wi Fi in the hospital is all set around that sort of service and community. If you get a bit, if you get a lockdown, you need to think how are we going to increase the band's exactly to allow, you know, if you're not able to contact their families and to be more in touch with them? And how are they going to do it? You know, how are they going to do it? People that don't deal with IT that well?

Researcher
Yeah, this is a good point. Is there anything you can think of doing better?

Participant
No, I mean, this is purely those two things. I mean, the other thing that I did manage to achieve, which was soon as I was able, where they were confident that I was safe on my feet, and they get permission to walk around the wards. So as long as there was none in them, but that did give me the permission to get a few unofficial visits from my family was they dropped in clothes and food.

Researcher
You're lucky because I have had participants who had no chance of visit.

Participant
Yeah, no. Well, it wasn't official, but they kind of were quite happy to turn a blind eye for you. It wasn't, you know me meeting them at the door was no different to them, a nurse meeting them at all.

Researcher
Well, thank you Participant for this. Thank you very much. Thank you time. We want to move to the assessment. If you are okay with that. I'm just asking if you want to add anything that you think we missed about your experience with the stroke during the pandemic, so you can add that or otherwise we'll move to the assessment.

Participant
No, no, move on. That's fine.

Researcher
Can I have your permission to record this session, are you okay with that? Can I have your consent to do that?

Participant
In terms to what sorry?

Researcher
Yeah, to record this interview. Are you okay with that?

Participant
Yes, that's fine.

Researcher
All right, thank you very much. And can we have your name, if you can just say your name and that you are agreeing to record this session at the beginning, so we can start.

Participant
Okay, my name is Participant Sutton. And I agree to the recording of this session.

Researcher
Alright, thank you very much, Participant. And, as you know, from the information sheet, you have the right to stop at any time, if you feel uncomfortable, or tired, or for any other reason, you just tell me to stop or if you want to take a break or rest. This will take about 45 minutes to an hour, hopefully to finish everything, and the assessment afterwards. And, yeah, as I say, as you know, there is no wrong or true answers. We just are interested in your thoughts and your experience with rehabilitation after stroke. So feel free to explain what opinion you have or what is your thoughts, you want to tell us, we'll be glad to have your your thoughts. Thank you again, for your time today. Do you have any question before we start?

Participant
No, I don't.

Researcher
Alright, and thank you. So first, tell us about your experience with the news of having stroke. What was your actual experience of having a stroke? Like, the first time when you notice that you have had a stroke.

Participant
Okay, let me tell you about my stroke. And it was just right, very beginning of the pandemic. I've gone for a walk that day. And when I was walking home, my left foot started dragging on the floor. And I thought that's a bit weird. Why is that happening? I wasn't unfit. You know, I was walking, training for marathons, walking mountains and stuff. So I walked a lot all the time. So it's very unusual. A foot started dragging. And then when I went outside, and to put the washing on the line, and I couldn't hold the pegs with my left hand, so I'm struggling to put the washing on the line. And then I think it happened, probably, because then I fell, hit the floor. And then again, because it was right in the beginning of the pandemic, it was very, very quiet, so it was just nobody around. I live alone, so I was there all night. And fortunately, my next neighbor heard me calling out the following morning, came round and got me. So that was my, my stroke experience. I was outside all night, on the ground. And then when they came, I lost consciousness. So I can remember up to being carried up. It was being in the process of being carried out in back garden. When I woke up. I was in hospital.

Researcher
And then when you move to the hospital, did they tell you was a bleeding or… what was the problem with your stroke? Was it a bleeding or ischaemic?

Participant
It was a bleed, was a bleed.

Researcher
Okay. And then you are admitted to maybe acute unit for stroke?

Participant
I was in a stroke ward.

Researcher
How long was you there?

Participant
Three and a half months in total in hospital in the stroke ward, probably for about a week. And then I caught COVID, in the hospital. And then I got to COVID ward and then they came back to the stroke ward. It was all backwards and forwards.

Researcher
Why did you go back to stroke ward, have you had any recurrent stroke or…?

Participant
Walk? I had no therapy. But when I went to the COVID ward, and you know, therapy or anything.

Researcher
But did they tell you if you have had any other stroke after that, or it was only one?

Participant
Oh, no, I've had one stroke.

Researcher
Okay, I see. All right. So Participant, tell us how the stroke has affected your daily life, you said you were unconscious for maybe some time and then you were awake again. So what did you notice, how the stroke affected your physical abilities and your daily activities?

Participant
I was completely paralysed on my left side, and I'm still pretty grey. I couldn't feel or move anything at the beginning. I've had subluxation of my left shoulder as well.

Researcher
Okay, so sorry. I'm sorry for that. Yeah, just tell me more. How would that affect your daily activity?

Participant
Well, I used to work full time and drive and I can't do any of those things anymore.

Researcher
Okay. So what do you think were maybe the main challenge that you experienced as a result of having stroke during the pandemic time?

Participant
And I'm gonna say it was being alone. In hospital, I think what they do is they take, they're being called to be kind approach to making you do stuff. There is no, because we got no visitors around things. I suppose it's bounced off by the crew to be kind approach, to be bounced off with your family. Because I didn't see any family for three and a half months, it was a long time.

Researcher
Yeah. Was that also because of the COVID thing? Or you didn't have maybe someone available to visit you at that time?

Participant
The hospitals weren't letting people come in.

Researcher
Oh, I see. No visiting in hospital. And what are the challenges because of the pandemic do you think you had at that time other than family visits?

Participant
That was the main one.

Researcher

Okay. So let us move to your mobility, Participant. Could you tell us how this sort of, affected your mobility and walking after you get your stroke?

Participant
Okay, well, I couldn't walk at the beginning. And I came home in a wheelchair, couldn't walk when I came home initially. I can walk now.

Researcher
Okay, and how long was that for? Can you walk now?

Participant
Yeah, I can walk now.

Researcher
Okay, with any help? Or you can do that alone?

Participant
I can walk now. I can walk now.

Researcher
Okay. Okay. That's good. That's good. Yeah. But do you use any maybe crutches or maybe canes or something?

Participant
I can walk now. I can walk now.

Researcher
I see. All right. Okay, so let us talk about the care that you have received by NHS and other maybe community services after during the your stay in the hospital as in-patient and then after you're discharged home? So can you tell us what help or care did they provide you?

Participant
Yeah, so I've been quite lucky. I think, actually, I think at the beginning with getting COVID, it meant that my physio was restricted at the very beginning. And it didn't start for quite a while, because of course, I was infectious. And then the drugs that they gave me for COVID, gave me C diff. And I was also then doubly infected, and so on for quite a bit longer. I think it delayed my beginning physio and everything. It was quite difficult with that so I felt like it took a long time to get going. But then by the time I got back home, I had community physio, which was very good. Thing that I found difficult though, there was no commitment to what you'd get around. I think it was just kind of, because they didn't really know what was going on with COVID either or might come the week, then again, they might not.

Researcher
Okay, so what there was a disruption to the maybe you're scheduled with their care so they are not coming regularly because of the pandemic.

Participant
Yeah, they wouldn't commit to anything. They usually phone the day before and say Right, we'll come tomorrow. I mean, as it was, I have nothing else going on today. So I could, didn't make any difference. But yeah, but now I knew how often they were coming. Actually, towards the end, they were coming like five days a week.

Researcher

But, Jen, did they teach you anything about self-management, about doing things for yourself or maybe taking some of, carrying out some of your therapy at home by yourself? Or help of somebody who's close to you, maybe at home?

Participant
Myself monitoring, do you mean occupational therapy?

Researcher
Not occupational therapy, it's about carrying out maybe some exercises at home for yourself, or maybe recording some of your activities to share with your therapist, or maybe telling them if you have a problem, or if you have a certain goal that you need to achieve, or you want to achieve? With your maybe working, or maybe with your movement, or any other thing?

Participant
Yeah, so I didn't do many exercises at home by myself, because really, I needed somebody trained to be with me, because I couldn't walk or move all the beginning very much.

Researcher
How about how about now Jean?

Participant
I'm fine now. I'm fine. I'm okay now. Yeah, I think I should get up and dance for you or something.

Researcher
Alright, good. So Participant, during that time, did you see any of other people who have had the same thing, in regards to it, like they have had a stroke? And the way if you didn't have any contact with anyone, like sociate? Or maybe you work in a group therapy or something with them?

Participant
I would have loved that, to be honest with you. But no, there was nothing going on because of the pandemic. And some of the groups that haven't come back, also, I'm online. So the Stroke Association called me but I'm going to say they were a bit rubbish. So I would, you know, I didn't get much from the Stroke Association. They called me out of the blue and asked me questions. One of the things I need to do a bit more after my stroke is think about things ahead of time. So I know what I'm going to be asked. So I can give the answers to it. Just getting a question out the blue I find quite difficult to deal with. Stroke Association ringing me up, go into your, I just say, Yeah, in fact, the only thing I asked them actually to help with was my assessment. And they said they couldn't do that and just told me to phone up certain device. I phoned up and they weren't operating at the time. So…

Researcher
Jen, if you think that you will summarise all that thing that you need. You need it at that time, since you have had the sort of, until now, what thing would be if you can just summarise them for me, like, what would you like to have in terms of support? Or maybe therapy or everything?

Participant
Yeah, I definitely think more support from organisations like Stroke Association. And I know it's difficult, but during the pandemic, just a phone call, I found really, really difficult to deal with. And also because I live alone, it's just like, you need to then do that, which I couldn't do because my brain wasn't working properly. People just tell me what to do, fill in this form, fill in that form, I couldn't do it. It takes me months to fill in the form. You'd have to do it all yourself, which was my biggest problem. I couldn't do it myself.

Researcher

So maybe the biggest need was to have support and to have somebody who work with you on daily basis. Probably maybe this is the best way we can describe it. What could be other thing that you you need? You needed at that time?

Participant
I would say occupational therapy. The community OT that I got, she wasn't very good. I felt like she'd read a book on it. And she had no experience. I couldn't, couldn't map what she'd read as a person.

Researcher
Yeah. And how did she deliver the care to you? Was it by phone or she came to your home? Or how was?

Participant
She came to the house.

Researcher
How many times if you remember?

Participant
Oh, she came, we probably for about six weeks.

Researcher
Okay. And did you feel that like helpful to some extent or?

Participant
No, because I think what she did was she infantilised me, made me into a child, forgot that I was a grown woman. And although I did have a brain injury, I still had a brain. And I didn't appreciate the way she spoke to me sometimes. And she'd also say, just reach forward and do that. And I was going I've got a shoulder subluxation, I can't just reach forward my left hand and do stuff. I asked for help around stuff because I was struggling with my memory, short term memory. Her advice to me was write down and get a piece of paper and write stuff down. I can't write left handed and my left hand doesn't work anymore.

Researcher
So before stroke, you were left handed, right?

Participant
Yeah.

Researcher
Okay. And so now, can you write like, did you get to manage your writing? Or maybe if you want to?

Participant
Um no. I've got no feeling in my left hand, so I can still write with my right hand but I can't, I can't control the pen. Inability to write and an inability to control the writing fluency.

Researcher
Yeah, I see. All right. So, Participant, if you can tell me now, how are you doing with your rehabilitation? Do you still receive any term of rehabilitation?

Participant
I do not, not through the NHS or the local council or anything like that. I'm paying for a private physiotherapist.

Researcher
And this was provided by, like, you search for that and like, there was no help from NHS or other community services for selecting a therapist.

Participant
No I did get some physiotherapy from community services, but for quite some time, actually. Overall, probably for about eight months worth of physiotherapy from them.

Researcher
Yeah. And how you feel with support of the therapists that you are being for? How do you think your involvement…

Participant
so community physio was great. One, they provided me with things to help me so I've got like a walker from walking stick. Well, so I've got a Roma, I've got walking sticks, they sorted ou balances on my stairs and a grab rail in my shower. They were great. They helped me with a lot of things. The first part of it was the early support service. They were great. They were really good. They got me walk in and got me up the stairs.

Researcher
Was that at your house or another facility?

Participant
It was in the house.

Researcher
Okay good. So, Participant, if you tell us by the time of discharge from hospital to your house, did the healthcare professionals teach you any strategies? Maybe to do, like, to be independent or maybe to do some therapy or maybe, like how to manage at home?

Participant
At what time period are you asking me this question around? Are you asking me when I was in hospital when I got home?

Researcher
Yes. At the time of discharge from hospital to your house.

Participant
Yeah I mean, they'd helped me a bit about getting dressed and stuff like that. And how to stand up and transfer from the bed to a chair. So I did have a little bit and I had also done a few steps of them, literally steps, I've walked maybe a meter with zimmer. But it was very scary transition.

Researcher
So why do you think was a scary transition?

Participant
maybe lack of the support. Well, you know, you're going from having people around you 24/7. I mean, I came home, they would have sent me home alone as well. But fortunately, my sister and her husband came to live with me. You know, I was there. I was lucky in that respect, but you would have sent me home alone, which I think would have been a massive struggle for me, I could see.

Researcher

So was there anything else that they teach you? And not just at the time of transition from hospital to your house but during the service you have? Like you have had your stroke, did they teach you anything about self-management? Or you feel that you were involved in setting goals for your rehabilitation?

Participant
I set all my goals, I set all my goals, they didn't set any of them.

Researcher
Yeah, but did they help you or they mention to you that you should be involved in setting your goals? Or this is something that you looked out, like, independently and you worked on that?

Participant
No I set them with them, so I said what I want to do with them.

Researcher
With whom?

Participant
With the physios

Researcher
Physios, that was before you, you came home?

Participant
No, no, I never seen the physios from hospital again. When I got home, I set my goals.

Researcher
Yeah. Okay. But when you were receiving care at hospital, you were also involved in setting some goals for your rehabilitation as well?

Participant
Gosh, it's a long time ago. I'm going to say yes, I think I was.

Researcher
Okay, was there anything else like, a part of setting your goals? Did they follow few of selecting, maybe some of the exercises that you feeling better with your situation and to do them? Or this wasn't the case, they just maybe teach you you have to do this and this for your exercise?

Participant
They were very focused on setting my goals so that I could get home. I think that was their main aim. Their main goal was that I could sit down for four hours so I could be home. They're more worried about that than they were about anything else. Their goal was about getting me home. I think the social services, it's about, you know, get for our carers turn up. So they wanted me to able to sit down for four hours. That was the main goal in hospital. Yeah, I felt

Researcher
But what was your… what were your goals? You said you set some goals for yourself? What were your goals? And how are they different from the ones at hospital?

Participant
My goals were about becoming independent. Not about, not sitting down for three hours. The exact opposite they wanted me to do, I'd never in my life sat down just for three, four hours in a row. Apart from

watching a film, really. It wasn't something that I did. I tended to do stuff. So Mine is all about doing stuff, you know, I walk every day at the moment. I'm achieving 5000 steps a day.

Researcher
Wow. That's great Participant. Do you have other goals to increase that amount of steps? Like in the future?

Participant
Yeah, I'd like to good.

Researcher
Good luck with that. What else?

Participant
I've also ridden my bike.

Researcher
Um, for how long?

Participant
Oh, not very long, but, but it is a good start.

Researcher
But it’s a good start. So yeah, good for you.

Participant
If the DVLA actually did any work to get me license back, and take a driving assessment.

Researcher
Um, if you can also think of any other goals, to be independent or any other goals about that.

Participant
Get back to work.

Researcher
Okay. Yeah, and what steps have you taken to maybe achieve this goal?

Participant
I've been logging onto my laptop. It's really about a brain thing that it's not a physical thing as much as about remembering, stuff.

Researcher
Yeah. Well, that's good. Participant. Have you heard about self-management? Or did the therapist or anyone else from NHS or community services, mentioned self-management to you?

Participant
No

Researcher
Because this is a concept where we actually involve people with stroke or any other health condition in the care of delivering care for themselves. So we have maybe some people to maybe record their daily activity in order to achieve certain goal or maybe to say to their words, or their therapeutic exercise plan, this is called self-management.

Participant
So I'm going to say I self-manage myself every day. I go for a walk every day. I've started reading again. So I couldn't read very much because my concentration kept slipping. So I've taken chance to read every day. Yeah, I've also playing the piano, I cook my dinner every day. This is self goals.

Researcher
This is great Participant, you will be independent, more and more, I hope in future.I was asking if anyone have mentioned that as a concept or something to you, while they are teaching you to move out your house, but you are aware of it, like by yourself and doing it to some extent that, this is actually a good thing. Participant, let us move to, hopefully, because we actually we need to see what you think about services that should be ideally delivered to people with stroke. So if you think if the pandemic comes back at any time, and there will be lockdown, and another major pandemic, so how the care or the rehabilitation should be delivered to people with stroke? What do you think the best way to have care after stroke if the pandemic is still there?

Participant
I definitely think I mean, the physio in the house was invaluable. Um, absolutely invaluable. I think other stuff would have been, I don't know, some way of trying to carry on with groups about meeting other stroke victims. Because I'm doing all that online, member groups online. But basically, from looking at what they can do has given set some of my targets, they can do that, I can surely do this. That's been very good. For me, actually, this, I think, the thing that's, I've got the most from mentally is definitely online stroke groups.

Researcher
So, yeah, I mean, if you can think like, back to the services, or maybe any care that you have received, and how that can get more better during the pandemic time, if anything can be done, like more efficiently and effectively, to people with stroke. Think during your in-patient and when you move to your house as well.

Participant
When I was an inpatient, I felt a lot like I was being treated like a child. And I know it's to make it easier for them to care for you. I think they forgot I was an adult. And I know not everybody with stroke is the same. And that's quite difficult but mentally, I was struggling. I struggled a lot mentally because I spent my home time with carers who I'm convinced just take on my occupation, because there's nothing else they can do. That sounds a bit harsh, but lots of…

Researcher
Participant, do you think that treatment, how you were treated at that time, was because of the COVID thing or this is maybe the way they treat people and they should change that?

Participant
I don't know one of the things I kept thinking when I was in hospitals, healthcare assistants forgot the word care. And they were very much focusing on the assistant stuff and there wasn't any care given. I mean, I got told things in hospital by the healthcare assistants, things I wasn't usual for stroke victims to have depression or anxiety. Now I completely see that differently. I think it's very usual stroke victims of anxiety or depression. But in hospital, they told me that it wasn't usual. And that made me feel worse actually because anything, oh my god, I'm taking this worse than everybody else. To know that I was reacting in normal way would have been so much better.

Researcher

This is shame on them to tell you this thing. I think all people after stroke will experience some of the anxiety or distress. And this is normal thing. This is normal. They shouldn't tell you that this is unusual thing.

Participant
Yeah, they did tell me, a week before my stroke, unfortunately, I found a friend of mine dead in his house.

Researcher
I was sorry for that.

Participant
So I was obviously suffering from that still. Yeah, they are calling me.

Researcher
Okay, I can wait for you if you want. That's fine. Participant.

Participant
No it’s gone. I switched it off. All right.

Researcher
Thank you very much, Participant. We are at the end of the interview. If you want to add anything about your experience. If anything you think we have missed maybe, in regards to your experience with with stroke rehabilitation, during that time.

Participant
I think if I was going to ask my sister who looked after me, really when I came out of the hospital, thumbs in the way, it just moves from it, takes a whole life of its own. And my sister would have said support from like the council or social services. The first time I heard from my social worker was to phone me to ask me if I had enough money to pay for my care.

Researcher
You found that very helpful having, maybe a sister or a family or a family member with you?

Participant
Oh, yeah, without the family member, social services were atrocious. She didn't get touch with me for eight weeks until after I'm on laptops. And that was eight weeks before I heard from them. So, first question was how much money have you got?

Researcher
I see. Yeah. What else do you think we missed of your experience with stroke?

Participant
Yeah, I think my sister felt a bit like she'd been abandoned. You know, she'd been given me and her, my life was in her hands. And she found that quite intimidating, worrying, yeah.

Researcher
I see. All right.

Participant
It’s there the other thing that happened to me in hospital was I kept being sick. I didn't eat. I lost about 30 kilos of weight.

Researcher
Wow. That's, that's a lot.

Participant
Yeah, just from not eating and being sick all the time. When I was sent out she was given no guidance about food, how to deal with that.

Researcher
Okay, so this was maybe one of your needs that you need at that time, maybe having some advice about your food and nutrition and all these things maybe?

Participant
Yeah I think the hospital could have given any advice on that because they weren't. I wasn't being treated very well for that in hospital either. I thinks it was a broke ward. Stroke doctor isn't a specialist on what's going on with me gastricly. I mean, every day in the stroke ward, they count how many wees you do, how much liquid you take, your bowel movements. No one was counting how often I was being sick. I could be sick about six times a day, none wrote it down once.

Researcher
Oh, sorry. I'm sorry for that, Participant. One more last question, if you want to add anything, and then we'll move to that question. Do you have anything to add or we move to that?

Participant
No, I don't think so.

Researcher
Okay, so I'm just interested to talk about your mobility and the thing that you have received in terms of exercise, or any, maybe fear, or before you're working on mobility, if you can just tell us a little thing about that. Like, like, what were you after stroke in terms of mobility? And when…

Participant
I couldn't walk, okay, well, once I've learned how to walk, when I got home, I used to take myself outside with somebody else, friends or family. And I walked down the street a bit. And I have a target for myself. So every day, I want to walk a bit further, sort of walk to a lamppost. And then the next day, the lamppost after that, or the tree after that, depending how far I think I'd go. Gradually, faith in myself further.

Researcher
But during the in-patient time, you maybe didn't receive a lot of help with that. What would you say?

Participant
No, not really. I mean, it was my ambition that was to walk further. The community physio did take me out once. And they wanted me to walk down the road, but they were timing me. Oh my gosh, that was awful. I hated that being timed. I felt like timing me was about a measure for them. Nothing about me. What I wanted to do is walk well and confidently, not walk fast. The walking fast bit, as soon as they put their watches on time, it stressed me right out. I found an awful, awful measure. It's not about walking fast. It's about walking properly.

Researcher
Yes, yes. But maybe because of them, you know, backlog they have, maybe they have a lot of people, maybe to help. This is an issue that has been with NHS, and a lot of service actually around. People are not getting useful help from the therapists, because of the timing and the restraint on the services.

Participant
I think their way of measuring an improvement, I don't think it's a valid way of doing it. And it made me feel quite stressful when they did it as well. They also did the thing when we sat in a chair and we standing up sitting down and times, how many times I could do it in a minute.

[Moderator explains market research and that the interview will be recorded]

If you can just Participant, start please with telling us about your experience of having a stroke, especially during COVID time.

Yes, do you want to know how it happened, or what do you need to know first of all?

Yes. Like how it happened and when you were admitted to the hospital, and how it affect your body and activities.

Yes, okay. So I was moving house and [signal breaks up 0:02:33.7] house, and I was painting the new house. I had my head up high and I was painting the ceiling … got lights. I had those before probably three times a year, and they last about three minutes. It's just a bit of flashing lights and it just goes, but this time I could tell it was something different. So I got off the chair, but as I got off the chair I felt like a sensation, like on my head all the way down my right side, it all tingles, numbness and then I felt really sick and dizzy. So I had to kind of sit on the floor with my head down because I couldn't sit up. I managed to shout my little boy, he was downstairs, so he called my partner, and he came to me. My friend came as well, and my sister because my little boy rang everybody. When they saw me, they said, 'We need to ring an ambulance straight away.' The ambulance came really quick, that was good, and they did a few tests on me, to see if I could say a certain sentence, which I could say but it was a bit gobbledygook.

They lifted my right arm up and it stayed up, but it didn't stay up, it just slowly drifted down. They weren't too sure, they never hinted at anything. I didn't know what was wrong. I thought it might have just been from the paint. I wasn't sure. They took me into A&E, that was about five o'clock. A nurse…

When was that, sorry?

July this year. They took me to A&E. They took blood from me quite quickly and did a heart trace to make sure my heart was okay. Then I didn't see anybody again until one o'clock in the morning. So that's eight hours. [Signal breaks up 0:04:29.8] a CT scan of my head. They said, 'Everything's fine, you can go home,' even though I had no feeling in the right side of my body and I couldn't walk, because I wasn't strong enough to walk, I had to be in a wheelchair. They sent me home and they said come back to another department called, I think it's GPAU, something like that. It's like a GP department. They said to come back there in the morning. So I came back the next morning, all the time not being able to feel anything. I was really scared; I didn't know what was happening. They did some more tests, some more heart tests, and eventually they diagnosed me with hemiplegic migraines which is some kind of migraine that can cause temporary paralysis. They said, 'After about a week, it should go.' They gave me some tablet, [?amitriptyline - signal breaks up 0:05:23.8] and said, 'You'll be fine in a week.' That was it. I left it about four…

Participant, I have trouble hearing you. Maybe…

Okay, can you hear me now?

Yes, now is okay. Can we just switch the video off so maybe it will be stronger, if we just keep the call without video?

Yes, okay. Do you want me to end it and ring you back?

No, just turn off the video and I'll turn mine from here, then we'll see how that goes. Yes, go ahead.

I forgot where I got to now. So yes, diagnosed with that, then I woke up on the fourth morning and I couldn't see out my right eye properly. It was completely black on the side, and I'd got flashing lights. So I rang the doctor and they said, 'It sounds like it's a problem with your eye, you need to go to eye casualty.' I went to eye casualty; they did lots of tests on me. They said my eyes are fine, that there's a problem with the back of my head but they didn't say what, they said I need to go back to the GPAU department. I went back there, they did some more heart traces and more blood work, then they left me sitting there from I'd say midday till eight o'clock at night, I hadn't seen anybody, and they close, the

GPAU, it doesn't stay open, that particular part closes. So it got to about ten to eight and I managed to find someone, and I said, 'I've not been seen yet, I've been here all day.' They said, 'Oh, okay.' They took me in a room, and they said, 'Really sorry, you've been sent to the wrong department, you need to have an MRI scan and you won't get one here, you can only get one in the stroke department or the A&E department.' So they sent me home again.

Came back the next day and I was took [sic] to the stroke department. Still no one had said they suspected a stroke. No one ever said anything. Went there, they did some more heart test, they scanned my carotid artery, did an MRI scan, this was all quite quickly. Then they just took me in a room and said, 'Really sorry, you've had a stroke.' They showed me a picture of my brain, they said…

Was it Participant, your first stroke, right?

My first stroke, yes.

Sorry about that.

Thank you. I didn't really know what they were. I'd heard of strokes, but you don't really ever know what they are properly. So he kind of said to me, 'Any questions,' and I was like, 'I don't know,' because literally I've just been told I've had a stroke, I don't know what to say. So I said, 'No, that's okay.' He said, 'The stroke team will contact you in a couple of days.' So I got given some more tablets, blood thinners and statins, told to stop taking the other ones, the amitriptyline because that wasn't the right diagnosis, I didn't need to take them any more. Sent me home and then…

So after MRI they sent you home.

Yes, straightaway, just sent me home. They signed me off work for four weeks, and they said, 'You might get better, things might improve, things might not, you need to just kind of get on with it and just see what happens, the stroke team will be in touch.' They rang me the next day, the stroke team and arranged to come and see me. They were really good. They are allowed to come and see you for six weeks, but they only came to me for I'd say three weeks. What they do is they check that you can get up the stairs, check you can go to the toilet, have a bath, do simple little things like cooking….

No I mean, when they did the MRI was there any intervention to release the stroke at that time?

No, because I'd had the stroke on the Wednesday, I didn't get an MRI till Sunday, so you have to have the stroke intervention literally in the first few hours. When I went to A&E at five o'clock, I didn't get my first CT scan till one o'clock in the morning so that's eight hours they did nothing with me. I think that's the most important part, you have to do something in those first three to four hours, otherwise it's too late. So it was too late for me. They just didn't suspect I had a stroke because I was too young, I was fit and healthy. I don't know why because the things they tell you, so my speech was a bit funny, it wasn't really bad, but it wasn't normal. I couldn't hold my arm up properly. My sister said my mouth was a little bit slanted. I was confused and I had no strength in my right side but still nobody thought that I'd had a stroke. They just said it was a migraine. It was an awful experience in the hospital. I just felt like I was…

Yes, sorry to hear that. So Participant, tell me after that what happen after you sent home and after they confirm that it was a stroke. What care did they provided you? What they did after that.

So they took me in a room, told me I'd had a stroke, showed me the picture of my brain, told me I'd need tablets to prevent any future strokes. They said, that was it really. 'Any questions?' I said, 'No, not really.' I couldn't think of any questions at that time. They sent me home and said, 'The stroke discharge team will be in touch.' That was the last thing I heard from the hospital, that day. It was literally, 'Sorry, bye.'

They didn't follow up with you.

Till now.

Yes, I mean after they discharge you, did stroke team reach at you or not?

Yes, so they didn't discharge me straightaway. The hospital kind of said, 'The stroke team will follow up.' The stroke team came, not the next day, the day after. They were really good, they send like a nurse round as well to see you, she came once I think it was. They check your blood pressure every time they come, make sure that you're okay. They checked my mental health. They gave me a few exercises to do for my arm and my leg because it was really weak. By this time I could walk again but I was walking with a limp, and my right arm was very weak still, and I still couldn't see. They kind of helped me for

three weeks, get me back on my feet a little bit, helped me to walk with more confidence and things like that. Then they discharged me because they said there was nothing else they could do….

After three weeks. They discharge you after three weeks.

Yes. They said I've just got to get on with life, just carry on doing my exercises, there's not a lot more they can do, 'You've just got to carry on doing the physio, and just do as much as you can with your life as normal to try and get back to normal.' So yes, they discharged me. At no point did anybody do hardly anything for my eyes which was my biggest problem. They kind of just said, 'Wait and see what happens.' In the end somebody said to me, 'Go to the opticians.' So I went to the opticians, they said, 'Yes, you've got a problem with your peripheral vision,' and they referred me back to the hospital and I got another appointment then with the hospital. They retested my eyes again and confirmed I had got peripheral vision loss. They do like this special visual field test to see how much is missing. They checked that and then they've seen me once again since then, done another check to see if it's got any better. It has got a little bit better, but I still can hardly see anything towards the right side.

I've got one more appointment with them I'm just waiting to get in the next month or so to see if anything's further improved since then. Once it kind of gets to six months that's the most amount of improvement you'll get. You might get a little bit, but once it's the six months, they say that's pretty much all you'll get vision wise.

Well, hopefully you will get better for that.

Hopefully!

Thank you, Participant, for telling me the story.

That's okay.

I just want you to tell me, because you had your stroke during COVID times, still at that time it was COVID time, and all the facilities are taking management seriously. So what do you think were the main challenges that you had experienced because you had a stroke during COVID, yes?

So obviously when I was taken into hospital in the ambulance nobody was allowed to come with me, so I was very confused, I didn't know what was happening to me. I couldn't really speak for myself because I couldn't, I could understand in my head what was going on, but I couldn't say it properly because I'd had a stroke obviously. No one could come with me and when I got to A&E, again nobody could come with me so I kind of just laid in a bed waiting for them. Whereas if somebody was with me, they could have gone to the nurses and said, 'Something's wrong with her, she isn't normally like this, somebody needs to come and help her.' Instead there's me just lying there in a bed, because I kept falling asleep as well, I think that's a sign, another sign, I was just drowsy, in and out. I think because I was just, I probably looked okay, just because I was lying there, they kind of just left me. Whereas I think if someone had been allowed in with me, they could have been on the doctor's case saying, 'What's going on, what's happening, this is not normal.'

When the nurses come and see you half the time nobody even knew what was wrong with me, nobody knew I couldn't walk, so when they said, 'You can go home,' I said, 'Well how, I can't walk.' They said, 'Can't you, oh okay we better get you a wheelchair then.' Surely you should know what's wrong with me and even if you don't know what's wrong with me you should know I can't walk. I think if people were allowed in it would be a lot better because they were letting people in with the elderly, which I can understand, but I think if you've had something like that happen and they don't know what it is then surely that's more important than maybe catching COVID. In my opinion. It would have made my experience a lot better if someone could have come and spoken for me, especially because I couldn't speak for myself. Then I guess getting appointments, it was hard because everything's delayed because of COVID. I need help with my mental health afterwards, I've got really bad anxiety now, but I couldn't even get help with that because of COVID. You have to wait, well I'm still waiting, six months so far and when you've got mental health problems, you need it pretty soon, not six months later.

Yes, especially after stroke, because it shakes everything up. Like you have to get really a good support for that. So hopefully you'll get that very soon.

Yes, I hope so.

Participant, how did the stroke affect your mobility? Like walking.

Originally when it happened, I could still walk but it was very slow. I limped a lot, I couldn't feel much.

Which side of your body is affected?

My right side. My dominant side, yes, I'm right-handed and everything. So yes, my leg wasn't too bad, I could still walk, it was just numbness. My right arm, I couldn't really do a lot with that. I couldn't use my fingers for I'd say a week or so. They did come back quite quickly but I've got no feeling now in my toes or certain points all the way up the right-hand side of my body. It just feels numb, and pins and needles. My arm was quite bad, I couldn't do a lot with that, but gradually over time up until now I can just about use it properly. Three fingers are quite weak still, but they are getting better. There wasn't much strength in it, but it is a lot better now six months down the line, but it's not normal and it doesn't feel like my side of the body. It feels like someone's squeezing my arm and then it feels like I've got pins and needles all down my right-hand side. Then obviously I've still got the eye problem so that's not great either.

Yes, sorry for that. How do you feel that the mobility, or the walking is coming?

The walking's really good because before I had my stroke, I was a runner, so I'd done the London Marathon and half marathons and all sorts, so I think that helped me get my movement back, and also it happened during the school holidays, so I had six weeks to be able to walk because I have to walk my little boy to school and it's quite far, and I'm not allowed to drive. So I literally had to practice, I was out every other day just walking round my estate trying to get the strength back in my legs. That came back.

How is it now?

Now, I can walk fine. I just sometimes walk with a little bit of a limp if I'm tired or if I'm not concentrating properly on walking, then I do walk with a bit of a limp. I can walk fine, I can walk five miles, six miles, seven miles.

Without any stick or any assistance?

I've got a symbol cane which is, that's more for my eyes though. Yes, I don't need a stick, like a walking stick or anything, no that's fine.

This is good for you.

Yes, I was lucky in that sense.

Yes, because walking is really important.

It is important.

This is good to hear, and hopefully the other things, your eyesight, your vision, it will be better soon.

I hope so.

Participant, let us talk now about other aspect, about the care that you have received. Can you tell me about the care provided by NHS, or after you were discharged, by community services for your stroke?

Yes, so the NHS I've really touched on, which I didn't think they were very good at all in the hospital, just not interested in the slightest. I was ringing the, do you know the bell that they give you on your bed, the little button, I'd rang that a few times and you can see the nurses, and they just ignore you. They're not bothered. They come to you eventually, but I saw them sitting around having a chat with people, and they just look at you, and carry on with their day and come to you when they feel like it. I don't think at any point during my hospital stay I felt like I was being looked after. I was scared. I was on my own. I didn't know what was going on. I mean they were all nice enough people, I guess they were overworked or, I don't know. I just did not feel good at all. The stroke team that came afterwards, they were really nice. They really cared. Made sure you were okay. They [sic] was only for a short period of time.

Do you mean the one who came after the discharge?

Yes, after I was discharged.

So did they have a chance to visit you at home?

Yes, they'd come to your house every day because I was discharged straightaway, I didn't stay in hospital. So yes, they come every day apart from weekends, for three weeks they came to see me.

Okay, and after that.

After that, that's it. It's bye. That's what I said to them, I said, 'Well, what do I do now?' She said, 'Just get on with your life and ring your doctor if there's any problems.' So I've heard from the consultant, he rang me last week, that was six months since my initial stroke. He kind of rang me to tell me what they thought had happened, asked if there was any other questions, and I said, 'No.' I'd asked him a couple of things. Then that was it then. That was all I'd heard from him. So not much at all from them. It took, I'd had a few tests, so they do like heart tests, and CT scans on your heart, and they did one on my neck. I had one in November, but I didn't get any results. The images weren't very good, but it took them till end of December to tell me that the images were no good. So I had to go back in and have the same tests done again, that I'd had done in early November.

This is shame on them, they are taking too long time actually to process.

Oh, they're taking far too long, yes. If I'd have had something wrong with me then it could still be going on six months down the line. They need to find out these things soon, so that they can fix them otherwise I could have had more strokes.

If you want to summarise, what do you think the impact of the pandemic on your care and rehabilitation?

I think it's awful. The pandemic has made a massive impact on healthcare and the healthcare that I received. I think more damage is being done by the impact of COVID than the actual being sick with COVID, if you see what I mean. If you understand what I mean. I'd much rather have COVID than have what happened to me. I think it's causing far more problems than what they think it is, than what they're trying to save.

Yes, you are right. Can you tell me about things or people that you feel were helpful to manage your situation?

Yes, so the stroke discharge team, they were amazing. Stroke consultant, he was nice, but I think, I just didn't know what to ask him. I had no clue what was going on. I was in shock, no idea. He was very helpful when I did get to speak to him, you just didn't get to speak to him very often because obviously he's a busy man. Other than that, the best people, it's not the NHS, the best people I've found to help me were the Royal National Institute for the Blind [sic] and the Stroke Association, the two charities. They've both been by far the most helpful people that I've spoken to at all over the whole process.

Did you seek help from them, or they reached you?

Yes. I seeked [sic] help from them. They're fantastic. They still ring me now, weekly, different people to check that I'm okay, to see what help I need, helping me get back to work.

This is very nice.

It's fantastic, yes. Oh they're amazing, yes.

Do they just follow up on certain things like mental support or physical support or they are covering different things, maybe just checking on you, how are you doing with everything?

Different things, so the blind charity obviously they're focusing more on my eyes, but they've helped me fill out, do you know the PIP that you can claim for, the monetary help from the government. They helped me fill all of those forms in. They've helped me get into contact with certain schemes that help you get back to work. They've arranged counselling for me that I'm just waiting for, so mental health as well.

This is great.

General chit chat, just to say, 'How are you doing, how are you getting on.'

Yes, I think this means a lot for people after stroke. Like this is doing good job especially with the lack of proper care from the NHS and other services.

From the NHS, definitely, yes. They've been the best people so far.

All right. So Participant, do you like me to call you Vicky or Participant?

Vicky's all right. Participant's my posh name for when I'm in trouble!

I think I should have ask this before, but…!

That's all right, don't worry. It's fine.

Thank you. So we want to talk about needs and goals. So after stroke can you tell me about needs during this period in terms of healthcare and rehabilitation services.

So what I need now?

Yes, what were your needs since you get stroke?

So I needed obviously physio therapy to try and get me moving again. I needed mental health help. What else.

Vision.

Yes, obviously vision, I needed massive help with my vision, which I didn't get any help at all until I went looking for it.

So if you would say which of your needs that you have mentioned so far were not fulfilled till now.

Were not fulfilled?

Yes, were not fulfilled.

I'd say the eyes was the biggest thing that wasn't fulfilled but obviously they are doing something about it now, but I had to go looking for it and keep ringing them to get someone to help me. The physio was quite good but obviously they only give you three weeks' worth.

Were not enough for you to cover…

I don't think it was enough no. I mean it was good enough to get me started but I think they should see you a little while after that just to check how you're going. Once they've discharged you from the stroke team that's it, you never see them ever again.

So Participant if I would ask you what is your main goal for rehabilitation after stroke, what would you say?

My main goal now is to be able to see again, which I don't know if that will happen, and I want to be able to run again because that was my life before. It's been a major goal.

Well, hopefully you will get that and at the end of our interview I want to speak to you about a study that I know can help with walking and mobility after a stroke. So if you would like, just remind me later and we should talk about that.

Okay.

Participant, let us talk about management strategies for your situation. Did the healthcare professionals teach you any strategies like to self-manage your situation at home, or something that you could use or build on for your recovery at home after discharge.

So they gave me exercises to do just to try and strengthen my arm.

They would prescribe the exercise for you, but did they engage you and ask you what is your goal for exercise or for rehabilitation?

Yes, they did. They asked me what I wanted to achieve….

And how you can achieve that, or like you felt like you are involved in learning for your care.

Oh yes, they asked me if I could pick five goals, what they would be. So obviously at the beginning it was smaller things like being able to walk to the shop, going to the shop on my own, being able to walk to school, little things like that, being able to run, being able to ride my bike. So they did set goals and then they ticked off the goals when you could do them or couldn't do them and what else you needed to do to be able to do them. So yes, they did do that.

How do you see that? Was it helpful?

Yes, very helpful. It helps give you a goal to work towards. Something to aim for, and then when you've done it, you can look back and think, oh wow, look before I couldn't even walk to the shop, now I go shop easily. It's nice to look back on and see what you couldn't do and what you can now do.

Well this is good enough. So Participant, I want you to also tell us about from the self-management strategies and being involved in planning for your care. If there is anything that you personally did to manage your situation for yourself, like action, or plan, or something, or strategies that you have taken to manage your situation.

Yes, so what I did was I kept like a diary and obviously I can't write very well so I try and do it as best I can, but I'd write down every day what I managed to do. So I'd write down like at the beginning, today I managed to walk to a shop, or I'd put this week I want to go and visit my mum or something, and I'd tick that I'd done that. I'd try and think of something good in the day that had happened, that I could write down and say, this day I did this, I managed to do this, I managed to walk a kilometre, or something like that. I did that. What else did I do? I'm trying to think now what else I did, but most of it was around exercising. Try and get myself up and try and keep myself busy and not go into a shell and not go anywhere any more because it was really scary going out and I didn't want to end up not going out. So I tried to give myself goals, just little things, like walk to the end of the road.

Were any of these goals targeting mobility.

Yes, so I'd start off saying I want to walk one kilometre, so I'd do that. Obviously to walk to school, it's two miles each way, so I had to be able to walk nearly eight miles, because I have to walk to school, then home because I was on sick leave at that time, and then walk back to school, and then walk back home again. So I think it's about seven miles altogether. I had to work up to be able to do that in the

school holidays. That was my biggest goal that I set because there was no one else to do it. I wasn't allowed to drive, I can't drive anyway, and my partner had to work. So that was the big thing that I had to do, and I managed to do it. It was hard, but the more you do it, the better it got. It definitely helped me improve, I think. If I didn't have to go to school, I think I'd be further back than I am now, definitely.

Yes, it's very nice to hear this. If I ask you, being experienced with doing thing for yourself and self-managing your situation, was that useful for you and why?

It did help because it gave me things I could look at to do. It gave me goals, it made me feel better myself because I knew what to work towards and what I'd achieved. It definitely did help me, but I think it's because once the stroke team went that was it. I kind of had to sit there and dwell on it or try and do stuff myself to make myself feel better. So I kind of had no choice, I just spent my whole time on the internet, researching who I could speak to, who would help me, where I could go, things like that to make myself feel better. That's how I found out about the different charities. There's Facebook groups for people who've had strokes, so I joined them, spoke to other people about it. You kind of have to help yourself.

These are very good strategies to improve your situation.

Yes, definitely.

Thank you very much Participant. We want to move to the last part of the interview. It's about future care and hopefully when the healthcare system go to normal, and they can cover everything. So what do you think will be the priorities for your rehabilitation if things go to normal?

What now, or before?

Let us say now, if things go back to normal, what are priorities for your rehabilitation?

Definitely mental health care needs to be a big help, if things went back to normal. Being able to get appointments quicker because it's a lot of waiting around for something that you need help with immediately or soon, rather than having to wait for so long. What else?

If you remember anything later, you can tell me.

That's the main two things I think, the mental health and the appointment system. Being able to see people face-to-face would be nice. Sometimes you can see them, but my stroke consultant, you don't get to see him.

Do you mean while at hospital?

At hospital yes, and now when I speak to the stroke consultant, you have to do it over the phone, you can't see them and it's hard to have a conversation with someone over the phone, it's easier face-to-face isn't it. I don't know, it just feels like they care more, and you can ask them more questions when you're sitting in front of them.

Yes, I can tell that. I'm just collecting some information for the study, and I can tell that. I'm not confident, I'm not comfortable at collecting my data online, while it could be more effective if we do face-to-face, or we can meet. That actually make a difference in people talking and expressing their thoughts and ideas. Participant, last question, if, and hopefully we won't have another wave of COVID or other pandemic, but if we have that, what would be a good plan to ensure that stroke patients can have a good plan for their rehabilitation after stroke?

I think letting people go into the hospital with you if you've had something like a stroke, or even a suspected one. I think visitors should be allowed to come in with you definitely.

Even if it is lockdown and people need to keep distance.

Yes, but I think being in the hospital on your own, when something like that's happened to you, I think you do need someone with you fighting your corner and being able to speak for you. It's hard to be able to be in hospital and have nobody when you aren't able to speak for yourself. I think that's a massive thing. What else? I think just getting the help quicker really. You need to be seen quicker; you need the appointments quicker.

Is that all you can think maybe? How about people if they got maybe a COVID during, while they are in hospital, so how can therapist or NHS staff reach them and deliver the care?

If the patients have got COVID.

Yes, they need to be isolated for some time.

Okay, I guess video calls might be helpful if they weren't able to see them. That would be good.

You're absolutely right. Participant I'm done with my question, thank you very much, but I want to give you the time now, if you feel that we miss anything that you want to share with us today, this is time for you. So please feel free to share whatever thing you feel we might miss during the interview.

I think we've covered most things. I mean the worst thing was just the treatment of the hospital, them seeming not to really care about you. When I went back to the hospital on one occasion, because I went back like four days in a row, my partner was parking the car and obviously I couldn't walk properly and I had my little ten year old with me, and I was almost kind of bent over, leaning up against the hospital wall, to try and just keep my head up, and the amount of nurses and doctors and other people that just walk past you and just ignore you. I guess they thought I was drunk or something, I don't know, but I had my little…

Oh, this is shame.

It is a massive shame. It's just like, they just didn't seem to care. It was, 'Oh let's just ignore them,' and they just walked past you. I think it's a lack of caring that got to me the most.

Yes, well they should have that sense of caring for other people.

Definitely. I feel if they'd have seen me quicker in A&E, they might have been able to do something to reverse the stroke whereas now I've probably lost my vision for the rest of my life and if they'd have seen me a few hours quicker, they could have - even if they couldn't have, if they'd just said, 'I'm really sorry, you could have had this medicine but it's not for your kind of stroke, so it's not going to help you,' but at least we thought about it and you could have maybe had it. At least then I wouldn't have been bothered because I'd have thought well, at least they thought of me, tried to help me but couldn't,

whereas now I didn't see no one for eight hours, there's no chance I'm getting any help. Nobody came to even check on me if you know what I mean. I think that's the worst thing, just thinking about how my life's changed and how it might not have done had I been seen quicker.

Well, hopefully things will be better.

Yes, hopefully!

Thank you very much Participant. Do you want to add anything, or should I stop the recording?

No. Yes, that's fine. That's okay. I can't think of anything.

If you remember anything, you can at any time, you have my email, you have my WhatsApp, so please feel free to share whatever information or experience you want to share. We would be happy to receive it at any time, not just during this time. I'll actually now…

[Recording ends abruptly]

[END OF TRANSCRIPT]

EBVTT

1
00:00:00.000 --> 00:00:00.299
Participant's iPhone: yeah.

2
00:00:00.359 --> 00:00:01.680
Researcher: You can do it now.

3
00:00:02.820 --> 00:00:03.090
Participant's iPhone: Okay.

4
00:00:04.799 --> 00:00:07.649

Researcher: We know that recording on you can state your name.

5
00:00:08.610 --> 00:00:10.320
Participant’s iPhone: Okay, my name is Nicole.

6
00:00:12.690 --> 00:00:13.889
Researcher: And you I agree.

7
00:00:14.790 --> 00:00:16.650
Participant’s iPhone: I agree to the recording.

8
00:00:17.369 --> 00:00:29.940
Researcher: All right, thank you very much surrender and just a refresher with the study information, so in this study we only actually interested in.

9
00:00:31.320 --> 00:00:47.820
Researcher: Listening to your experience with stock during the coffee time and what was like your journey look like and what care, have you received from NHS or in the Community services for your.

10
00:00:48.600 --> 00:00:58.200
Researcher: habitation after stroke, there is i'll ask you some questions, there is no right or wrong, so i'm just feel free to share whatever information you want to share with us.

11
00:00:58.530 --> 00:01:01.440
Researcher: feel free to just tell us about your story.

12
00:01:02.100 --> 00:01:09.840
Researcher: And if you feel tired or fatigued or gonna stop the interview at any time just let me know this is your right.

13
00:01:10.200 --> 00:01:15.150
Participant’s iPhone: So sorry interrupt along with this recording for this.

14
00:01:17.340 --> 00:01:18.990
Researcher: It will be around an hour.

15

00:01:20.220 --> 00:01:22.110
Participant's iPhone: Oh it's gonna be too much yeah.

16
00:01:22.320 --> 00:01:31.590
Researcher: yeah but if if you need to break it depends actually on your like responses and how the questions are going, so there is, you know certain time.

17
00:01:31.980 --> 00:01:43.680
Researcher: We can finish early, we can do like it depends on how we, we can share like talk or right so don't worry about timing, we can start anytime you can have a break just let me know right.

18
00:01:46.080 --> 00:01:56.820
Researcher: yeah, so this is all about the study, so now i'm going to start with you asking the question, but before that, let me know if you have any question or you need any more information.

19
00:01:59.460 --> 00:02:00.510
Researcher: you'll get to start.

20
00:02:01.080 --> 00:02:14.790
Researcher: Yes, all right, thank you cylinder again for taking part in this study, so if you can at the beginning, please tell us about your experience of having a stroke with have been at that time.

21
00:02:17.130 --> 00:02:26.130
Participant's iPhone: Well, I was at work, because my normal shift and i've just done, the T rounds and everything else come about just have to turn.

22
00:02:28.140 --> 00:02:35.100
Participant's iPhone: The lights will switch we were going to switch the lights off, so our routine is like closer, we can go and close the curtains and everything.

23
00:02:35.970 --> 00:02:36.690
Participant's iPhone: And I.

24
00:02:37.860 --> 00:02:55.830
Participant's iPhone: approached a patient, and I said, is it OK, to close your curtains on your window and he says no leave it and then at the hospital, we still have them big Poles, so I had the palm of my hand and I just lifted my head up to pull the other window.

25
00:02:56.700 --> 00:02:57.450
Participant’s iPhone: That point.

26
00:02:57.540 --> 00:02:59.010
Participant’s iPhone: I felt a bit dizzy.

27
00:03:00.030 --> 00:03:02.400
Participant’s iPhone: And I didn't think nothing of it, so I.

28
00:03:02.850 --> 00:03:13.530
Participant’s iPhone: Try to move fast, because he says leave it don't have a look, and as I tried to move back it was like i'm trying to lift my leg, but it was like I.

29
00:03:15.000 --> 00:03:19.920
Participant’s iPhone: couldn't lift it's like lifting it in the air it's just air, but you couldn't move it.

30
00:03:20.370 --> 00:03:22.710
Participant’s iPhone: yeah so I shot I dragged myself.

31
00:03:22.950 --> 00:03:28.830
Participant’s iPhone: I just put the pole just it wasn't too far, the mercy station is just a couple of feet away.

32
00:03:29.550 --> 00:03:30.780
Participant’s iPhone: And I went and sat down.

33
00:03:31.500 --> 00:03:40.530
Participant’s iPhone: And at that point, I was really feeling lethargic and everything and then stop there, and I just says to her my blood sugar because I want my diabetic.

34
00:03:41.040 --> 00:03:42.660
Participant’s iPhone: yeah and then.

35
00:03:43.950 --> 00:03:49.410
Participant’s iPhone: Maybe for a couple of seconds a carton of what happened, but then she's telling me to move on, because i'm gonna fall.

36
00:03:49.860 --> 00:03:54.060
Researcher: yeah so when when did that happen.

37
00:03:56.550 --> 00:03:59.220
Participant’s iPhone: A housing, what do you mean when did that happen.

38
00:03:59.250 --> 00:04:03.240
Researcher: Like struck when did it happen, which day or Monday.

39
00:04:04.380 --> 00:04:07.020
Participant’s iPhone: The 25th of September.

40
00:04:07.650 --> 00:04:08.610
Participant’s iPhone: This year I was on.

41
00:04:08.790 --> 00:04:10.530
Participant’s iPhone: I was on the night shift yeah.

42
00:04:12.300 --> 00:04:13.530
Participant’s iPhone: I was on the night shift.

43
00:04:14.400 --> 00:04:17.670
Participant’s iPhone: Okay, and then she.

44
00:04:18.720 --> 00:04:20.940
Researcher: asked me what are you working as.

45
00:04:21.720 --> 00:04:23.130
Participant’s iPhone: yay yay.

46
00:04:23.550 --> 00:04:24.030
Okay.

47
00:04:25.770 --> 00:04:27.780
Researcher: Good okay continue.

48
00:04:28.380 --> 00:04:36.750
Participant’s iPhone: At that point, she called the other nurses to say adopting surrender is right, one of them checked my blood sugar.

49
00:04:37.890 --> 00:04:46.920
Participant’s iPhone: One was saying, can you move yourself but, but I just couldn't my right side of my speech, well, for me, and then I just vaguely.

50
00:04:47.760 --> 00:04:59.880
Participant’s iPhone: remember them getting upset and calling the team and asking details of my husband who to call, and I still can't believe it that's what happened because I just said it's my blood sugar.

51
00:05:00.450 --> 00:05:04.770
Participant’s iPhone: And they were saying no, you know, we need to call the team, then.

52
00:05:05.010 --> 00:05:15.510
Participant’s iPhone: next thing I know is everybody trying to move me on to the bed, and then the colleague saying, can you lift your right side lift to chinos I couldn't move it.

53
00:05:16.140 --> 00:05:19.530
Researcher: yeah so you're right side was Africa to.

54
00:05:20.130 --> 00:05:21.630
Participant’s iPhone: My right side was affected.

55
00:05:21.750 --> 00:05:22.200
Researcher: yeah.

56
00:05:23.130 --> 00:05:23.490
All right.

57
00:05:25.980 --> 00:05:34.890
Participant’s iPhone: That point, the only thing that was going in my mind was what's happening and i'm not gonna make it to me, it was a scary experience.

58
00:05:35.250 --> 00:05:40.320

Researcher: yeah i'm sorry to hear that at the show us what happened after that.

59
00:05:41.400 --> 00:05:42.180
Researcher: Like if you.

60
00:05:42.210 --> 00:05:45.210
Participant’s iPhone: Get admitted to the hospital yeah.

61
00:05:45.600 --> 00:05:50.490
Participant’s iPhone: yeah they took me to sign well that blue lighted we designed well.

62
00:05:51.120 --> 00:05:52.680
Participant’s iPhone: yeah and then I went.

63
00:05:52.800 --> 00:05:57.090
Participant’s iPhone: They took me down to have a CT scan.

64
00:05:57.510 --> 00:06:07.740
Participant’s iPhone: yeah and they said that's Okay, and I stayed up too early hours in any in one of the side rooms.

65
00:06:08.160 --> 00:06:16.950
Participant’s iPhone: yeah and my only thoughts was I had to try and keep moving, but my leg or mom or I could and.

66
00:06:17.790 --> 00:06:20.190
Participant’s iPhone: Then they moved me to another war.

67
00:06:22.560 --> 00:06:27.390
Participant’s iPhone: There was no beds available on the stroke unit yeah, then I.

68
00:06:30.960 --> 00:06:32.220
Participant’s iPhone: Had a scan and then I had.

69
00:06:36.150 --> 00:06:37.560
Participant’s iPhone: another symptom of throw up.

70
00:06:39.210 --> 00:06:40.950
Participant’s iPhone: An autoresponder and I had discussed.

71
00:06:41.550 --> 00:06:49.620
Participant’s iPhone: On a then they said that i'm going to wait for an MRI because that would be only confirm that my side was week.

72
00:06:50.220 --> 00:06:53.820
Participant’s iPhone: yeah I tried a lot to try and do because.

73
00:06:55.830 --> 00:06:57.540
Participant’s iPhone: When you see people around you.

74
00:06:58.890 --> 00:07:05.100
Participant’s iPhone: The ways it affected them more sometime they say we didn't know we tried to push ourselves to be.

75
00:07:05.580 --> 00:07:06.840
Researcher: yeah yeah.

76
00:07:07.290 --> 00:07:08.280
Participant’s iPhone: which I did.

77
00:07:10.980 --> 00:07:11.970
Researcher: So, have you done.

78
00:07:13.050 --> 00:07:13.650
Researcher: After that.

79
00:07:14.070 --> 00:07:18.750
Participant’s iPhone: After the MRI was carried out, but I am terrified of MRI so they.

80
00:07:20.010 --> 00:07:24.510
Participant’s iPhone: Give me something to calm me down a bit and the MRI confirm that I had this.

81
00:07:25.530 --> 00:07:29.430

Participant’s iPhone: As a small region of a stroke on.

82
00:07:30.900 --> 00:07:31.560
Participant’s iPhone: The Left.

83
00:07:32.040 --> 00:07:33.240
Participant’s iPhone: that's why the affected.

84
00:07:33.240 --> 00:07:34.770
Participant’s iPhone: The right yeah.

85
00:07:36.060 --> 00:07:39.210
Participant’s iPhone: denied had I had physio coming home.

86
00:07:39.930 --> 00:07:40.350
Researcher: yeah not.

87
00:07:42.390 --> 00:07:46.860
Researcher: In the stroke unit, how long did you stay there.

88
00:07:47.550 --> 00:07:49.530
Participant’s iPhone: I stayed there about three nights.

89
00:07:50.370 --> 00:07:55.140
Participant’s iPhone: three nights yeah yeah I mean has a hate CA I.

90
00:07:56.610 --> 00:08:08.640
Participant’s iPhone: Someone towards us so show you don't get the full care and I have a really supportive loving family and kids that support would support me if I came home.

91
00:08:09.060 --> 00:08:14.850
Participant’s iPhone: yeah I just said to them, I don't need no help because I know my family would help me okay.

92
00:08:15.420 --> 00:08:18.240
Participant’s iPhone: You been on the board, you are.

93
00:08:19.710 --> 00:08:22.800
Participant’s iPhone: i'm not putting your trust, but they're trying to do what they could do.

94
00:08:23.430 --> 00:08:25.140
Participant’s iPhone: To me, I needed to be home.

95
00:08:26.730 --> 00:08:29.640
Researcher: to your left your left the hospital after three days off.

96
00:08:30.990 --> 00:08:31.800
Participant’s iPhone: I came.

97
00:08:36.510 --> 00:08:40.140
Participant’s iPhone: So I think I was in hospital total about six days.

98
00:08:40.620 --> 00:08:42.630
Participant’s iPhone: Okay five to six days.

99
00:08:42.750 --> 00:08:44.430
Participant’s iPhone: yeah yeah.

100
00:08:45.120 --> 00:08:49.440
Researcher: Okay, thank you signed up for this, so now I wanted you to tell me.

101
00:08:51.030 --> 00:09:00.270
Researcher: How was that the stock affected your daily activity and like what changes have you noticed have been at that time.

102
00:09:00.540 --> 00:09:03.660
Participant’s iPhone: Oh, I you can't wash yourself.

103
00:09:04.260 --> 00:09:10.530
Participant’s iPhone: yeah you obviously need somebody to take a walk to the toilet with you.

104
00:09:13.710 --> 00:09:20.280

Participant’s iPhone: You can't do the things you want to like say, for instance, you want a cup of tea you're waiting on somebody to make it for you.

105
00:09:20.820 --> 00:09:24.810
Participant’s iPhone: yeah you know the little things you take for granted, you can't do them.

106
00:09:25.470 --> 00:09:25.980
yeah.

107
00:09:27.630 --> 00:09:30.750
Participant’s iPhone: I mean in the beginning, I couldn't hold a big cup a cup of tea.

108
00:09:33.090 --> 00:09:34.290
Participant’s iPhone: So it was like.

109
00:09:35.610 --> 00:09:39.030
Participant’s iPhone: Have a small little cup of tea not fall to the top.

110
00:09:39.570 --> 00:09:40.110
yeah.

111
00:09:41.160 --> 00:09:48.570
Participant’s iPhone: It says a little things you totally depend on each day you don't know until you can't do them you feel hopeless.

112
00:09:48.990 --> 00:09:49.470
yeah.

113
00:09:50.820 --> 00:09:51.330
Researcher: Sorry for.

114
00:09:52.500 --> 00:09:56.580
Researcher: Sharing that I want to go back and remember.

115
00:09:57.630 --> 00:10:06.930
Researcher: How did the coffee situation at the hospital and African affected your your decision to go home and not to stay.

116
00:10:07.320 --> 00:10:07.650
Researcher: I thought.

117
00:10:08.820 --> 00:10:15.510
Participant’s iPhone: It was very bad because, even though I work at the hospital you're alive, no visitors.

118
00:10:15.960 --> 00:10:20.100
Participant’s iPhone: yeah because my daughter works in the hospital as well.

119
00:10:20.490 --> 00:10:26.790
Participant’s iPhone: yeah that you are allowed somebody to drop off some food or clothes, or whatever you need.

120
00:10:27.150 --> 00:10:33.780
Participant’s iPhone: yeah you're not allowed to visit and one of my colleagues they've come to see me on the Ward.

121
00:10:35.070 --> 00:10:40.410
Participant’s iPhone: Training and she wanted to come and see me she is a nurse.

122
00:10:40.710 --> 00:10:50.520
Participant’s iPhone: yeah and i'm sorry to say that i'm not mentioning our Ward because i've been on about towards one Ward would not let her come and see me.

123
00:10:50.910 --> 00:10:51.450
wow.

124
00:10:52.770 --> 00:10:59.280
Participant’s iPhone: They says no, is the policy she says, but our work in the trust, I just want to see my colleague see if she's okay.

125
00:10:59.670 --> 00:11:09.000
Participant’s iPhone: yeah so one of them came up to me and she said you've got a you've got a work colleague, but we can't let our end due to the call we tone to stand.

126

00:11:09.300 --> 00:11:09.720
Researcher: yeah.

127
00:11:10.260 --> 00:11:15.960
Participant's iPhone: It was just a bit would have been just a brief high and she was concerned that's why she wanted to come and see.

128
00:11:15.960 --> 00:11:18.870
Researcher: me, and it means a lot for you in that situation.

129
00:11:19.170 --> 00:11:20.490
Participant's iPhone: yeah yeah.

130
00:11:21.780 --> 00:11:22.530
Participant's iPhone: So.

131
00:11:22.770 --> 00:11:35.400
Researcher: But you know, maybe If anyone was care at that time on things that maybe weren't a clear like what is like they don't want to take maybe the responsibility of breaking the.

132
00:11:36.870 --> 00:11:46.680
Participant's iPhone: Day to me if she didn't have to come right into the debate, I will say just from a distance, I could have lived in halfway and she could have just waved.

133
00:11:47.250 --> 00:11:53.160
Participant's iPhone: yeah I didn't know who it was until I saw her from a distance to see where it was.

134
00:11:53.760 --> 00:11:54.030
yeah.

135
00:11:55.770 --> 00:12:01.830
Participant's iPhone: There was nobody to take me take me down to see her so I sort of dragged myself.

136
00:12:02.970 --> 00:12:09.120
Participant's iPhone: Because I even though they said I was mobilize a mobilizing on the Ward but it's dragging you know you're like.

137
00:12:09.600 --> 00:12:09.930
Researcher: yeah.

138
00:12:09.960 --> 00:12:21.180
Participant's iPhone: I thought halfway up the war to see who it was and then away doctor and then I came back I just say it is heartbreaking because Okay, I could just sort of drag myself.

139
00:12:21.630 --> 00:12:24.780
Participant's iPhone: yeah this call, has done a lot of damage.

140
00:12:24.900 --> 00:12:26.220
Participant's iPhone: To a lot of the.

141
00:12:26.730 --> 00:12:27.750
Participant's iPhone: Other patients.

142
00:12:27.900 --> 00:12:35.910
Researcher: yeah well, could you think of any other like challenges, because of coffee at that time.

143
00:12:38.520 --> 00:12:52.230
Researcher: Like while you were ambition and like can you think of any other like challenges that afflicted you at that time because of coffee, other than having someone to visit you or to take care of you.

144
00:12:52.980 --> 00:12:54.270
Participant's iPhone: Well, I suppose.

145
00:12:55.290 --> 00:13:05.340
Participant's iPhone: Normally, you could probably go if it was normal under nortel you could just if you knew, if you could just go up to the other patient and speak to them comfort them.

146
00:13:05.670 --> 00:13:10.470
Participant's iPhone: yeah calvi do you can't do you know there's a lot of barriers nowadays.

147
00:13:11.490 --> 00:13:13.470
Researcher: So maybe lack of search.

148
00:13:13.530 --> 00:13:15.540
Researcher: Social socialization with if anybody.

149
00:13:15.570 --> 00:13:20.100
Researcher: yeah and and patient or they will outside the hospital as well.

150
00:13:20.340 --> 00:13:33.630
Researcher: Yes, alright, so today, let us move to another aspect, can you please tell me how did the stroke affect your mobility and working in particular.

151
00:13:35.340 --> 00:13:36.990
Participant’s iPhone: What like I says, I.

152
00:13:38.220 --> 00:13:40.650
Participant’s iPhone: couldn't I couldn't go up the stairs.

153
00:13:41.490 --> 00:13:44.790
Participant’s iPhone: yeah so my bad my bad down.

154
00:13:45.390 --> 00:13:46.740
Participant’s iPhone: yeah and.

155
00:13:48.450 --> 00:13:52.440
Participant’s iPhone: it's like I said earlier, there's things you can't do because you can't walk properly.

156
00:13:52.860 --> 00:13:54.570
Participant’s iPhone: You know you're scared.

157
00:13:55.770 --> 00:13:56.730
Participant’s iPhone: You might fall.

158
00:13:57.930 --> 00:13:58.320
Researcher: yeah.

159

00:14:00.600 --> 00:14:03.090
Researcher: And yeah right.

160
00:14:04.470 --> 00:14:08.310
Participant’s iPhone: Well, your daily life is totally depending on other people like isn't it.

161
00:14:09.180 --> 00:14:14.940
Researcher: yeah yeah yeah but how do you see that, like embarrassing my time.

162
00:14:16.320 --> 00:14:16.920
Researcher: you're moving.

163
00:14:17.340 --> 00:14:19.200
Participant’s iPhone: Praise it's improved a lot.

164
00:14:20.670 --> 00:14:22.950
Participant’s iPhone: My physio they've been great.

165
00:14:24.540 --> 00:14:26.040
Participant’s iPhone: seeing them supported.

166
00:14:27.210 --> 00:14:27.660
Researcher: yeah.

167
00:14:27.750 --> 00:14:33.660
Participant’s iPhone: I did get a purchase in store she's all she encouraged me was to do what I can do, but don't push yourself.

168
00:14:35.550 --> 00:14:38.580
Participant’s iPhone: And I have been trying to do little bits myself.

169
00:14:39.300 --> 00:14:42.990
Participant’s iPhone: And like I said, my family have been very supportive and there's things like.

170
00:14:43.200 --> 00:14:46.710
Participant’s iPhone: That really nice that will encourage you couldn't do that.

171
00:14:46.800 --> 00:14:47.340
Researcher: mama.

172
00:14:47.400 --> 00:14:52.650
Researcher: You know we will we will we will actually cover this in particular questions.

173
00:14:52.950 --> 00:14:57.120
Researcher: Because you want to ask this in in in some details so.

174
00:14:58.170 --> 00:15:03.720
Researcher: Soon, that can you tell me about the care about care provided by NHS.

175
00:15:05.010 --> 00:15:11.160
Researcher: staff or other Community services after you left the hospital, can you tell me what care, have you received.

176
00:15:11.730 --> 00:15:20.880
Participant’s iPhone: The only care, I received from them was the physio the other care, I said I was Okay, because my family would help, they give him a lot.

177
00:15:22.230 --> 00:15:22.680
Participant’s iPhone: For me.

178
00:15:23.280 --> 00:15:27.240
Researcher: and start right, you were impatient.

179
00:15:27.420 --> 00:15:30.510
Researcher: And the hospital or that the stuff that after you discharge.

180
00:15:30.990 --> 00:15:35.250
Participant’s iPhone: Because due to call the they couldn't come in, so, while I was there I.

181
00:15:36.510 --> 00:15:37.380
Participant’s iPhone: There was one or two.

182
00:15:39.210 --> 00:15:47.460

Participant’s iPhone: Days that was nice that was willing to help me shower whatever I needed that they did give me that they did support me.

183
00:15:48.180 --> 00:15:54.660
Researcher: yeah Okay, and after you discharge what what can have you to shift.

184
00:15:56.070 --> 00:15:57.570
Participant’s iPhone: What from the NHS.

185
00:15:57.690 --> 00:15:59.640
Researcher: Yes, either community services.

186
00:16:01.140 --> 00:16:05.430
Participant’s iPhone: from them, the only it was only the physio I.

187
00:16:06.720 --> 00:16:08.610
Participant’s iPhone: required because, like I said.

188
00:16:10.380 --> 00:16:17.100
Participant’s iPhone: my daughters, they did a lot for me there's a washed me what whatever help I needed.

189
00:16:17.430 --> 00:16:21.960
Participant’s iPhone: They come in the morning and be here until the evening so.

190
00:16:22.020 --> 00:16:22.320
Researcher: They would.

191
00:16:22.740 --> 00:16:29.220
Participant’s iPhone: Come prepare my breakfast do the dinner, and the lunch, and then they would go and then they would come back in the morning.

192
00:16:29.760 --> 00:16:31.110
Researcher: yeah yeah.

193
00:16:32.490 --> 00:16:32.850
Researcher: and

194
00:16:33.930 --> 00:16:38.130
Researcher: Tell me tell me from time you this shark till now, what have you received.

195
00:16:39.210 --> 00:16:39.450
Researcher: Right.

196
00:16:40.290 --> 00:16:42.690
Participant's iPhone: Well, the thing is i've already received.

197
00:16:44.970 --> 00:16:45.780
Participant's iPhone: The physio.

198
00:16:46.140 --> 00:17:00.150
Participant's iPhone: yeah self has been and i've had a seven day tape and i've had a code on on my managers she's a five phone calls from her they've all been supportive.

199
00:17:01.620 --> 00:17:02.790
Participant's iPhone: If there's anything they.

200
00:17:04.560 --> 00:17:06.030
Participant's iPhone: If anything I want from them.

201
00:17:06.540 --> 00:17:08.310
Participant's iPhone: yeah nothing to the well being.

202
00:17:09.000 --> 00:17:24.450
Participant's iPhone: Of the well being of been there for a massage slice and they do a one to one, so that is the care that are after care obviously from the NHS the theory the carries there if a person wants to take it.

203
00:17:25.050 --> 00:17:37.230
Participant's iPhone: yeah they do offer a lot if you want to take it, but like I said I only took up the well being because I thought I need to get out of the House and socialize and get back to normal.

204
00:17:37.770 --> 00:17:54.210

Participant’s iPhone: But my husband and my son lives with me and they're very supportive they do as much whatever they can do, but now I am doing better, and I am doing the cooking I can almost do my daily chores I do get tired.

205
00:17:54.690 --> 00:17:57.420
Participant’s iPhone: yeah I can do everything myself.

206
00:17:59.400 --> 00:18:04.380
Researcher: Because this is great to hear, because not everybody after so can do this.

207
00:18:05.400 --> 00:18:10.590
Participant’s iPhone: I have been sort of pushing myself to think i'm has they'll say you're.

208
00:18:10.590 --> 00:18:11.250
Researcher: still young.

209
00:18:11.430 --> 00:18:17.760
Participant’s iPhone: And everything and I recently started exercising number by.

210
00:18:18.210 --> 00:18:21.240
Participant’s iPhone: yeah for about 510 minutes.

211
00:18:24.750 --> 00:18:36.120
Researcher: shown that can you tell me about what did the physio actually gift to you or how did they, like many of you.

212
00:18:36.480 --> 00:18:41.070
Participant’s iPhone: When the physio cable she says your walk properly.

213
00:18:42.120 --> 00:18:45.240
Participant’s iPhone: Slowly so she gave me a walking stick.

214
00:18:45.720 --> 00:18:50.220
Participant’s iPhone: Okay, which and then she gave me this thing on my.

215
00:18:51.420 --> 00:18:54.420
Participant’s iPhone: New issue on it's like a little lamb.

216
00:18:54.930 --> 00:18:56.220
Researcher: yeah if.

217
00:18:57.450 --> 00:19:03.390
Participant's iPhone: it's I forgot that the body it's like it's like a strap you put on your foot.

218
00:19:03.600 --> 00:19:03.720
You.

219
00:19:05.760 --> 00:19:12.480
Participant's iPhone: know from the front you put it into your in your trainer and then you put a strap and it brings you for top like that.

220
00:19:12.720 --> 00:19:13.680
Researcher: yeah yeah.

221
00:19:13.890 --> 00:19:17.730
Researcher: And maybe I if i'm Kim food orthotics.

222
00:19:18.600 --> 00:19:20.310
Participant's iPhone: And he.

223
00:19:21.780 --> 00:19:24.540
Researcher: just told me you're fortunate to have.

224
00:19:25.230 --> 00:19:25.770
Participant's iPhone: This one.

225
00:19:26.490 --> 00:19:28.680
Researcher: OK OK, I see yeah yeah.

226
00:19:28.710 --> 00:19:29.460
Researcher: yeah oh.

227
00:19:30.330 --> 00:19:33.630

Participant’s iPhone: Santa Maria that this was really good it really did help.

228
00:19:34.110 --> 00:19:43.500
Participant’s iPhone: Okay, because she was telling me to like to lift my fault, but I just couldn't lift my foot like you normally would walk.

229
00:19:43.830 --> 00:19:52.980
Participant’s iPhone: yeah but with that and she she would want to come, she would take me outside, for you know for about five minutes walking.

230
00:19:53.100 --> 00:19:59.160
Participant’s iPhone: yeah day and she did say do some exercises she left me some exercises.

231
00:19:59.250 --> 00:20:03.900
Researcher: yeah how many times, did they visit you for physio.

232
00:20:04.980 --> 00:20:09.390
Participant’s iPhone: It was, I think, in total she's been about seven times.

233
00:20:09.810 --> 00:20:13.770
Researcher: Okay, so be done in in three months, are you today.

234
00:20:14.790 --> 00:20:17.040
Researcher: Oh yeah yeah yeah yeah.

235
00:20:17.370 --> 00:20:21.780
Participant’s iPhone: And she did say I mean she got me the Perkins to to sit on.

236
00:20:22.260 --> 00:20:22.950
Participant’s iPhone: yeah and when.

237
00:20:23.070 --> 00:20:24.240
Participant’s iPhone: We looked at how.

238
00:20:25.290 --> 00:20:32.010
Participant’s iPhone: You know how what getting it out of the shower she got me a seed that really helped a lot yeah what.

239
00:20:32.130 --> 00:20:32.430
Researcher: This is.

240
00:20:35.160 --> 00:20:38.370
Participant’s iPhone: Yes, sorry I couldn't climb the stairs.

241
00:20:38.610 --> 00:20:39.060
Researcher: mm hmm.

242
00:20:39.330 --> 00:20:41.400
Participant’s iPhone: And then I had the real for two days.

243
00:20:43.110 --> 00:20:45.120
Participant’s iPhone: So i'm doing better.

244
00:20:45.660 --> 00:20:54.870
Researcher: mm hmm and hopefully by time you still have a time to improve So hopefully, you will be moving much more.

245
00:20:55.110 --> 00:20:57.480
Participant’s iPhone: And while i'm hoping to go back to work.

246
00:21:00.420 --> 00:21:16.830
Researcher: yeah hopefully let's just pray for that saying that if you just go back and think what do you think that was the impact of coffee on care and rehabilitation services.

247
00:21:23.490 --> 00:21:26.550
Participant’s iPhone: Is a lot of impact on it, because.

248
00:21:26.820 --> 00:21:27.180
Researcher: yeah.

249
00:21:27.600 --> 00:21:38.490
Participant’s iPhone: You get on some boards if there's an lds there's some things that only that they're familiar with the families and family would come and do that through to cultivate they not.

250
00:21:38.940 --> 00:21:48.750
Participant's iPhone: The loved ones are not getting that care because you've got patients that are violent but because their family can come in, they can talk to them and they reassure them.

251
00:21:49.920 --> 00:21:57.990
Participant's iPhone: I mean we encourage before the call video that we had an elderly patient that refuse to eat or anything we would encourage the family to come in and.

252
00:21:58.440 --> 00:21:59.730
Participant's iPhone: We are coming.

253
00:22:00.270 --> 00:22:01.560
Participant's iPhone: yeah now given.

254
00:22:02.910 --> 00:22:04.440
Participant's iPhone: The reassurance.

255
00:22:04.800 --> 00:22:13.230
Participant's iPhone: yeah but that's impacting you can't be there, feel of ones, and I think being in hospital, you need to be a support from your family's.

256
00:22:14.850 --> 00:22:18.690
Participant's iPhone: seeing them, you know and it's reassuring for them as well, to know that.

257
00:22:19.860 --> 00:22:26.040
Participant's iPhone: Your state it's fine for me to say all i'm Okay, but until they haven't seen you.

258
00:22:26.490 --> 00:22:26.910
Researcher: mm hmm.

259
00:22:27.180 --> 00:22:31.860
Participant's iPhone: they're not going to receive ease I mean my husband was worried, I mean.

260
00:22:32.250 --> 00:22:35.700
Participant's iPhone: They used to I used to text them or form them to say i'm

okay. 261

00:22:36.240 --> 00:22:36.570
Participant’s iPhone: But I.

262
00:22:36.960 --> 00:22:38.460
Participant’s iPhone: wouldn't see you.

263
00:22:40.080 --> 00:22:41.700
Participant’s iPhone: me saying i'm okay.

264
00:22:42.060 --> 00:22:46.020
Participant’s iPhone: yeah Okay, for me, but for the person, on the other side yeah.

265
00:22:46.050 --> 00:22:46.800
Participant’s iPhone: They don't know.

266
00:22:47.280 --> 00:22:48.690
Researcher: yeah, it is a half.

267
00:22:49.410 --> 00:22:50.430
Participant’s iPhone: Until you see.

268
00:22:51.480 --> 00:22:53.040
Participant’s iPhone: yeah I think this cold feet.

269
00:22:56.430 --> 00:22:57.240
Participant’s iPhone: it's terrible.

270
00:22:58.860 --> 00:23:00.750
Researcher: Okay, so um.

271
00:23:02.160 --> 00:23:16.050
Researcher: So how How would you see the inbox after you just show you talk about being in the hospital, but how the impact in, for we will have to be discharged from hospital.

272
00:23:17.130 --> 00:23:20.670
Researcher: Like how did, that the coffee afflicted their rehabilitation.

273
00:23:21.120 --> 00:23:31.320
Participant's iPhone: cold data that would come, the only thing is, is like you know before you say hi you know sometime you shake hands with somebody but you can't know.

274
00:23:32.220 --> 00:23:33.030
Participant's iPhone: The mascot.

275
00:23:33.330 --> 00:23:37.080
Participant's iPhone: And sometimes because you've got your mask on you can't always hear.

276
00:23:38.520 --> 00:23:40.950
Participant's iPhone: Properly clean what the other person is saying.

277
00:23:41.310 --> 00:23:42.540
Researcher: yeah yeah.

278
00:23:42.960 --> 00:23:45.750
Participant's iPhone: And then obviously you got to try and keep your distance.

279
00:23:46.020 --> 00:23:47.670
Participant's iPhone: yeah so.

280
00:23:48.420 --> 00:23:53.970
Researcher: I think you were lucky, because you had your surgery, in a time of some release.

281
00:23:55.020 --> 00:24:05.070
Researcher: For the services, I have interviewed some people who had it earlier in 2021 or 2020.

282
00:24:05.520 --> 00:24:10.770
Researcher: Where there was locked down and they didn't have a chance to have any physio our tool.

283
00:24:11.100 --> 00:24:13.260
Researcher: or anyone to come through and help them.

284
00:24:13.830 --> 00:24:16.200

Participant’s iPhone: Oh well, I think i'm lucky that.

285
00:24:16.440 --> 00:24:18.870
Participant’s iPhone: yeah if I hadn't.

286
00:24:19.950 --> 00:24:34.230
Researcher: Like seven sessions in in three months, this is the maybe that the the them, I think the most one who had session in three months, like second session in famous This is like.

287
00:24:35.370 --> 00:24:37.710
Researcher: The most visited when.

288
00:24:38.790 --> 00:24:48.060
Participant’s iPhone: I had my pastor coming from hospital think about two weeks later, I had this low and then every week, she would come.

289
00:24:50.880 --> 00:24:57.360
Participant’s iPhone: The, the only thing when she put her appointment to come, she didn't come because she says, let me know.

290
00:24:58.710 --> 00:25:00.330
Participant’s iPhone: When you have the staircase.

291
00:25:01.770 --> 00:25:02.250
Participant’s iPhone: and

292
00:25:02.820 --> 00:25:09.300
Participant’s iPhone: She did offer me most part, because I actually said i'd rather sleep down here because I told him that his bathroom is downstairs.

293
00:25:09.510 --> 00:25:14.010
Participant’s iPhone: yeah I come down from upstairs would be too much for me, but.

294
00:25:15.480 --> 00:25:18.930
Participant’s iPhone: Eventually, I did sell would want to go back upstairs and then.

295
00:25:20.850 --> 00:25:27.570

Participant’s iPhone: They offered me the rail, but she says we can't promise, how long it'll take but surprise the rail was done.

296
00:25:28.320 --> 00:25:30.900
Participant’s iPhone: yeah within the three months.

297
00:25:31.200 --> 00:25:47.160
Researcher: Good good about about from the help of the physio cylinder, can you tell me about things or be able that you feel were helpful for your situation and how do you to to to go for this situation.

298
00:25:48.240 --> 00:25:52.140
Participant’s iPhone: Well, my family and my kids play a great part in it.

299
00:25:52.530 --> 00:25:52.890
yeah.

300
00:25:54.900 --> 00:26:05.550
Participant’s iPhone: Like I said they would come in the morning do my breakfast do everything and then my family and friends were colleagues have woodring and texts to see how I am.

301
00:26:06.120 --> 00:26:07.500
Participant’s iPhone: yeah my manager.

302
00:26:08.040 --> 00:26:10.800
Participant’s iPhone: reassuring me that if there's anything she can do.

303
00:26:12.090 --> 00:26:13.200
Participant’s iPhone: Just let her know.

304
00:26:13.800 --> 00:26:16.080
Participant’s iPhone: yeah and I had hockey health.

305
00:26:19.200 --> 00:26:21.330
Participant’s iPhone: They have to do their review anyway.

306
00:26:21.720 --> 00:26:26.430

Participant’s iPhone: yeah and then my manager saying that there is the well well being that I can go to.

307
00:26:28.230 --> 00:26:32.940
Participant’s iPhone: In overall i'm happy with the care, I received the only thing was the.

308
00:26:34.050 --> 00:26:37.290
Participant’s iPhone: downfall was colby and my family were allowed to visit.

309
00:26:37.890 --> 00:26:38.310
Researcher: yeah.

310
00:26:38.400 --> 00:26:43.560
Participant’s iPhone: And then, when you're in hospital and something like that happens, you feel down yourself.

311
00:26:44.130 --> 00:26:44.640
yeah.

312
00:26:46.260 --> 00:26:50.490
Participant’s iPhone: Probably can't see you that's The worst thing because you don't know what's gonna happen.

313
00:26:51.180 --> 00:26:51.600
yeah.

314
00:26:55.440 --> 00:26:56.370
Researcher: Sorry for that.

315
00:26:57.750 --> 00:27:07.350
Researcher: But at least now you are with them, and you will enjoy your life with with family all the time, hopefully, and you won't have this again.

316
00:27:08.220 --> 00:27:18.600
Researcher: Soon, that lets us move to another aspect to talk about the needs your needs after stroke and how you, you can fulfill that.

317

00:27:19.020 --> 00:27:35.490
Researcher: So I want you to tell me about your needs during that period after you, you had your stroke, in terms of health care and rehabilitation services like what What did you think that you, you really need at that time.

318
00:27:36.240 --> 00:27:49.530
Participant's iPhone: Well, like I says, I really needed a physio and which I got and, to be honest with my physio she suggested suggested things that before I even I even thought about them.

319
00:27:49.980 --> 00:27:51.750
Participant's iPhone: So I was quite happy with the.

320
00:27:51.750 --> 00:27:53.070
Participant's iPhone: Care that my physio.

321
00:27:54.420 --> 00:27:59.430
Participant's iPhone: gave and the option she gave to me, so I can't complain about that.

322
00:27:59.490 --> 00:27:59.850
Researcher: So.

323
00:28:00.210 --> 00:28:03.090
Researcher: So you'll feel like your needs for physio were.

324
00:28:03.480 --> 00:28:05.460
Participant's iPhone: need from physio side were.

325
00:28:05.460 --> 00:28:10.500
Participant's iPhone: filled mean I didn't even think of having a purchasing store because.

326
00:28:12.900 --> 00:28:14.640
Participant's iPhone: If I go in the kitchen are.

327
00:28:14.910 --> 00:28:17.760
Participant's iPhone: done for a couple of seconds, and I feel tired and not come back.

328
00:28:18.210 --> 00:28:27.180

Participant’s iPhone: But she says to me during the home depot I says, I don't because it's it's a kids do it so she got that, for me, and she even says to me that if I wanted a commode.

329
00:28:27.570 --> 00:28:31.950
Participant’s iPhone: If you're wanting to stay upstairs I could have that versus know i'm quite happy down here.

330
00:28:32.160 --> 00:28:33.030
Researcher: yeah good.

331
00:28:33.300 --> 00:28:34.530
Researcher: And yeah.

332
00:28:35.040 --> 00:28:39.480
Participant’s iPhone: If I need anything feel free to bring her up.

333
00:28:41.490 --> 00:28:46.170
Participant’s iPhone: From that from the physio and the support they've given me i'm very happy.

334
00:28:46.710 --> 00:28:51.480
Participant’s iPhone: That you said I was a lucky one to have that support when I came out.

335
00:28:51.900 --> 00:29:03.960
Participant’s iPhone: yeah I did say when I was at the hospital they did say if I needed anything and it's for us it's not because, due to some waiting lists that we've experienced when we've had patients seen.

336
00:29:04.380 --> 00:29:09.690
Participant’s iPhone: Yes, long waiting list and I thought I don't want to stay in hospital waiting.

337
00:29:10.260 --> 00:29:11.370
Participant’s iPhone: for things to be fitted.

338
00:29:12.090 --> 00:29:21.960
Participant’s iPhone: So I said I don't need anything but when I came home and my physio suggested yeah we could have a try she says it might be a long way, but it might not be but.

339

00:29:22.530 --> 00:29:22.830
Researcher: yeah.

340
00:29:22.950 --> 00:29:27.000
Participant’s iPhone: I was one of the lucky ones, because when she says the bedroom I mean the rail.

341
00:29:27.960 --> 00:29:28.710
Participant’s iPhone: It was, I think.

342
00:29:29.100 --> 00:29:33.270
Researcher: I think you are working in in health sector, how do you understand.

343
00:29:33.510 --> 00:29:35.670
Participant’s iPhone: The situation, I think.

344
00:29:37.440 --> 00:29:39.840
Participant’s iPhone: That was the case when I was in hospital.

345
00:29:40.200 --> 00:29:40.620
Researcher: yeah I.

346
00:29:40.800 --> 00:29:43.050
Participant’s iPhone: Think they'd let your visitors come.

347
00:29:43.380 --> 00:29:52.680
Participant’s iPhone: yeah just let one I mean what thing my husband's face when he when I got transferred to Sandwell they're.

348
00:29:53.100 --> 00:29:53.730
Participant’s iPhone: giving me.

349
00:29:54.960 --> 00:29:56.460
Participant’s iPhone: The only let him on to my Ward.

350
00:29:56.490 --> 00:30:02.490

Participant’s iPhone: Because the water work on, because obviously my work colleagues knew him as well.

351
00:30:02.700 --> 00:30:07.290
Participant’s iPhone: yeah i'm that he's a right to anyway.

352
00:30:08.820 --> 00:30:16.710
Researcher: So it's good i'm sharing that can you tell me what is your main goal for habitation after stroke.

353
00:30:17.280 --> 00:30:23.040
Participant’s iPhone: My main goal is get back to normal do the things I would have liked to do.

354
00:30:23.250 --> 00:30:25.530
Participant’s iPhone: Okay enjoy the time with my grandkids.

355
00:30:25.980 --> 00:30:48.690
Researcher: Good good good and now we'll move to the way, how did you manage your situation on a personal level or based on the care that you have received so did the health care professionals to teach you in the strategies to self manage your situation at school, after you get.

356
00:30:49.350 --> 00:30:51.660
Participant’s iPhone: A physio did not physio date.

357
00:30:52.200 --> 00:30:56.040
Researcher: yeah so, can you explain that, like how did that.

358
00:30:56.250 --> 00:31:00.720
Participant’s iPhone: To just says to me if I will this is how I should try a walk.

359
00:31:02.850 --> 00:31:17.310
Participant’s iPhone: not like this, this is what I need to do is take a step by step if i'm going to do live something to it like this it all came with the exercises so some of the things was some things are common sense.

360
00:31:17.760 --> 00:31:18.780
Researcher: yeah but.

361
00:31:19.890 --> 00:31:24.960
Researcher: Did you feel like they engaged you in planning for your exercise.

362
00:31:25.380 --> 00:31:26.220
Participant’s iPhone: or the other.

363
00:31:27.720 --> 00:31:29.400
Researcher: If your rehabilitation.

364
00:31:31.050 --> 00:31:31.530
Researcher: To do.

365
00:31:32.370 --> 00:31:33.030
I think.

366
00:31:34.050 --> 00:31:39.000
Participant’s iPhone: I was offered more support from a physio when I came home.

367
00:31:39.360 --> 00:32:01.620
Researcher: yeah that's good that's good and so about about from self management strategies that professional the euro physio taught, you can you tell me please what else you have been doing on your ambition and living back you encourage yourself, or you set goals or like how did you manage.

368
00:32:02.490 --> 00:32:03.630
Participant’s iPhone: To do is.

369
00:32:05.400 --> 00:32:11.940
Participant’s iPhone: I with my fitbit I tried to do so many steps a day with it, and that was my goal.

370
00:32:12.450 --> 00:32:16.530
Participant’s iPhone: Okay, so each day, I will try and do a few extra.

371
00:32:17.700 --> 00:32:22.860
Participant’s iPhone: started off with your couldn't even do about 200 to 300 up in the early one or two days.

372
00:32:23.100 --> 00:32:26.820
Participant's iPhone: yeah slowly, it will just literally walk into the toilet and back.

373
00:32:27.180 --> 00:32:28.800
Participant's iPhone: Now my goal is.

374
00:32:28.890 --> 00:32:30.960
Participant's iPhone: I mean my doctor keep saying.

375
00:32:33.600 --> 00:32:35.250
Participant's iPhone: you've got to try and lose a bit of weight.

376
00:32:35.880 --> 00:32:47.220
Participant's iPhone: Okay do will be exercise so in the last couple of days, what i'm doing now is I tried to go for a walk for about half an hour, which leads it becomes more than a half an hour.

377
00:32:47.520 --> 00:32:48.660
Researcher: yeah yeah.

378
00:32:48.750 --> 00:32:50.280
Participant's iPhone: And I.

379
00:32:52.500 --> 00:33:00.330
Participant's iPhone: got an exercise bike I tried to spend about 510 minutes on there and or even more or less depending on how tired I get.

380
00:33:00.990 --> 00:33:02.040
Researcher: you'll get that at home.

381
00:33:02.550 --> 00:33:04.290
Researcher: yeah okay good.

382
00:33:04.830 --> 00:33:12.810
Participant's iPhone: And then i've got a yoga mat I tried to do i'm trying to do a little exercises with my arm on my legs specially.

383

00:33:13.470 --> 00:33:16.650
Researcher: To show you have been busy and keep trying different.

384
00:33:18.330 --> 00:33:19.110
Researcher: lucinda.

385
00:33:20.550 --> 00:33:26.190
Participant’s iPhone: And if I can't do much of them what I tried to do is run on the spot slowly.

386
00:33:26.760 --> 00:33:27.240
Researcher: yeah.

387
00:33:27.390 --> 00:33:28.200
Researcher: yeah that's great.

388
00:33:28.800 --> 00:33:34.350
Participant’s iPhone: it's just trying to strengthen the left side, I mean the right side.

389
00:33:34.740 --> 00:33:48.630
Researcher: yeah yeah, can you tell us more about mobility thing, so you said you using the bike using some exercise you're using your fitbit to to measure that the steps and try to brush your.

390
00:33:51.210 --> 00:33:51.810
Participant’s iPhone: cooking.

391
00:33:52.440 --> 00:33:52.860
yeah.

392
00:33:54.750 --> 00:33:58.110
Participant’s iPhone: There was some point where you couldn't see I couldn't cut.

393
00:34:00.300 --> 00:34:04.140
Participant’s iPhone: Even a potato, because it was too, it was too hard.

394
00:34:04.620 --> 00:34:10.380
Participant’s iPhone: yeah the hand felt weak, but now has gradually I am getting into that routine where.

395
00:34:11.850 --> 00:34:19.950
Participant’s iPhone: i'd say about 80% of myself getting there it's just that little fatigue and some time if i'm doing something I will have to stop.

396
00:34:21.000 --> 00:34:21.480
Participant’s iPhone: But.

397
00:34:23.880 --> 00:34:29.520
Participant’s iPhone: kids that making adjustments to it, you know, like washing machine now.

398
00:34:29.700 --> 00:34:30.150
yeah.

399
00:34:31.980 --> 00:34:36.060
Participant’s iPhone: i've got something I can do my doorway for much a party.

400
00:34:36.810 --> 00:34:39.900
Participant’s iPhone: They are helping this things you tried to.

401
00:34:40.350 --> 00:34:42.990
Participant’s iPhone: do to make your life easy.

402
00:34:44.190 --> 00:34:45.540
Researcher: that's fine if even.

403
00:34:45.660 --> 00:34:48.780
Researcher: Like even without stroke, sometimes you just use.

404
00:34:49.020 --> 00:34:50.910
Researcher: yeah well, the thing that tells you.

405
00:34:51.330 --> 00:34:54.180
Participant’s iPhone: Something so you've got a just, temporary, but obviously.

406
00:34:54.480 --> 00:34:55.230

Participant’s iPhone: Try to do.

407
00:34:55.260 --> 00:34:56.190
More my hand.

408
00:34:57.840 --> 00:35:02.940
Participant’s iPhone: While trying, while i'm sometimes teaching i'll just do this with my grant my leg.

409
00:35:03.300 --> 00:35:03.750
Researcher: yeah.

410
00:35:03.870 --> 00:35:05.790
Participant’s iPhone: I think, can you do helps.

411
00:35:06.420 --> 00:35:07.440
Participant’s iPhone: will get you there.

412
00:35:08.160 --> 00:35:08.610
Hopefully.

413
00:35:10.050 --> 00:35:14.040
Researcher: This is great actually i've been doing so many things to achieve your goal of.

414
00:35:14.310 --> 00:35:27.330
Researcher: Going back to normal life and, hopefully, you have achieved that so last question about that the self management strategies so we're any of them.

415
00:35:28.200 --> 00:35:39.570
Researcher: strategies that you are using to self manage your situation, useful and you feel like your filter This is like very useful for me like what you have doing.

416
00:35:41.040 --> 00:35:47.010
Participant’s iPhone: I would recommend anybody with a stroke don't pity yourself fight, you can do it.

417
00:35:47.910 --> 00:35:51.030

Researcher: yeah in this specific strategies.

418
00:35:51.450 --> 00:35:52.980
Researcher: Like how to self manage.

419
00:35:54.390 --> 00:36:11.820
Participant’s iPhone: Well, the thing is each person's individual I mean I don't know the depth of the pain, the other person is going to have, but when i've even been sitting down, and I have all i've been doing is moving my leg a tiny bit this way that way any little movement is better than normal.

420
00:36:12.690 --> 00:36:21.330
Participant’s iPhone: yeah so all I say is don't just sit there Doc just lying in your bed, even if it's just walking up to the door and back.

421
00:36:22.350 --> 00:36:23.220
Participant’s iPhone: just keep going.

422
00:36:23.790 --> 00:36:24.960
Researcher: With us firestone.

423
00:36:27.300 --> 00:36:30.480
Participant’s iPhone: Well i'm going to do it from our own kids go my grandchildren kids.

424
00:36:30.870 --> 00:36:35.340
Participant’s iPhone: They climb up pick them up there, you know you you like a child, with them yourself.

425
00:36:35.610 --> 00:36:38.460
Participant’s iPhone: yeah and then missing all yeah.

426
00:36:39.210 --> 00:36:48.300
Researcher: All right, thank you, Sir, for this information last two questions and we done within a few seconds I want you to tell me.

427
00:36:49.800 --> 00:36:57.270
Researcher: Like after the coffee thing got all far and hopefully the healthcare system will go to normal.

428
00:36:59.340 --> 00:37:05.490
Researcher: Like, what do you think the priorities for habitation after service should be.

429
00:37:06.960 --> 00:37:10.980
Participant’s iPhone: Well, they should start straight away, make it clear.

430
00:37:12.270 --> 00:37:24.960
Participant’s iPhone: And I know this isn't going to happen, but what they should try to do any adaptations, they need to do, they need to do them quicker for the patient to come home, so the current quarter, they can.

431
00:37:26.100 --> 00:37:28.620
Participant’s iPhone: start their recovery procedure procedure.

432
00:37:29.130 --> 00:37:34.230
Participant’s iPhone: because some people they they wait and wait and wait before everything's done.

433
00:37:34.860 --> 00:37:36.060
Participant’s iPhone: And that is delaying.

434
00:37:36.270 --> 00:37:46.020
Participant’s iPhone: them getting better because they think, obviously, because I have all got that in place, I can't do it, but you can start doing things, because if, when things are not in place.

435
00:37:46.500 --> 00:38:01.410
Participant’s iPhone: Do what your body can do don't overdo it, but do what your body is you know when you, you know you when your body is telling you you tired just leave it, but then get back up and do it again yeah personally, you will get there slowly.

436
00:38:02.160 --> 00:38:08.610
Participant’s iPhone: I didn't think I could say this now, but I do feel a lot better now hundred percent when.

437
00:38:08.790 --> 00:38:10.170
Researcher: You are just cube.

438
00:38:11.940 --> 00:38:13.200
Researcher: Hopefully you will reach that.

439
00:38:13.740 --> 00:38:22.620
Participant’s iPhone: yeah well yeah i'm getting there, and you just look at Head of you you've got a bright treat everybody score for each other and be there for your kids.

440
00:38:22.830 --> 00:38:23.340
Researcher: As you.

441
00:38:23.370 --> 00:38:24.450
Participant’s iPhone: see that for yourself.

442
00:38:25.980 --> 00:38:27.180
Participant’s iPhone: mean there's nothing else.

443
00:38:28.260 --> 00:38:29.820
Participant’s iPhone: Like doing things for yourself.

444
00:38:30.360 --> 00:38:42.960
Participant’s iPhone: yeah when when you have depend on some political things it's not that Nice, but obviously he he can't help it, but you want to do it, so you know you can do it your way yeah.

445
00:38:43.230 --> 00:39:06.630
Researcher: All right, thank you for this last question sent in that if hopefully we won't have any live spoke or Banda make in the future, but if it happens again and we need like I look down or whatever So what do you think should will be the ideal land for treatment of stroke people.

446
00:39:08.070 --> 00:39:09.600
Participant’s iPhone: early treatment for people.

447
00:39:09.900 --> 00:39:16.830
Researcher: yeah like how we ensure that there, we will be in good hands, while coffee
or whatever is.

448
00:39:17.280 --> 00:39:25.050

Participant’s iPhone: pretty well then try to keep it well, like I said there's no I mean NHS is supposed to be good.

449
00:39:25.770 --> 00:39:27.030
Participant’s iPhone: Once you're in hospital.

450
00:39:27.420 --> 00:39:28.410
Participant’s iPhone: You get the care.

451
00:39:30.390 --> 00:39:42.300
Participant’s iPhone: So get to the hospital as soon as she can, but I think that is the lack of getting you know, sometimes the lack of ambulances, lack of people get into your hours a lucky one because it happened to me while I was at the hospital.

452
00:39:42.780 --> 00:39:53.340
Participant’s iPhone: wow so I got seen to literally straight away, but can you imagine if that was somebody else somewhere else yeah people have lost their lives.

453
00:39:54.450 --> 00:40:01.290
Participant’s iPhone: Do two strokes, because haven't got there quick enough for the you know the support they need it yeah.

454
00:40:02.730 --> 00:40:04.320
Participant’s iPhone: But just hope this cold leads all.

455
00:40:04.350 --> 00:40:07.530
Researcher: Gone and things we all have that is just.

456
00:40:08.880 --> 00:40:10.560
Participant’s iPhone: A shortage of nurses hates.

457
00:40:11.190 --> 00:40:26.070
Researcher: yeah yeah I see yeah hopefully we won't have any future waves it just so you know, we want to think how we kind of define the health system and to be very bare if something had been infused shows.

458
00:40:26.220 --> 00:40:28.230
Participant’s iPhone: me yeah and then.

459
00:40:29.430 --> 00:40:32.190
Participant’s iPhone: You know the thing is everybody's needs a difference.

460
00:40:32.790 --> 00:40:33.270
yeah.

461
00:40:34.800 --> 00:40:35.310
Researcher: So right.

462
00:40:35.460 --> 00:40:41.370
Participant’s iPhone: So just I think what they have the NHS doing is, it is good, but they need more.

463
00:40:43.050 --> 00:40:54.030
Participant’s iPhone: nurses, you know more support behind them, I mean with this cold read the lack of support you get a nice because a lot of people are sick isolating you know everything.

464
00:40:54.690 --> 00:40:56.010
Participant’s iPhone: that's the downfall of it.

465
00:40:56.580 --> 00:41:03.120
Participant’s iPhone: But hopefully once this is all over everything's back to normal just leave a happy life.

466
00:41:03.630 --> 00:41:09.420
Researcher: yeah all right, thank you very much Center for your rich information i'm done with my question here i'm just.

467
00:41:10.350 --> 00:41:15.030
Researcher: i'm just giving you a time here, if you want to add anything that you feel we missed.

468
00:41:15.120 --> 00:41:18.270
Researcher: During the interview about your experience with stroke.

469
00:41:20.370 --> 00:41:21.360
Participant’s iPhone: thing is.

470
00:41:22.380 --> 00:41:25.770
Participant’s iPhone: The only but feeling I have this while I was in hospital.

471
00:41:26.430 --> 00:41:26.760
That.

472
00:41:28.260 --> 00:41:39.270
Participant’s iPhone: You know, at least, even if it's cold we don't know it's not a you know they still don't bend the rules, but even if it's cold coffee, they should still allow one family member.

473
00:41:39.720 --> 00:41:40.710
Participant’s iPhone: To come and visit.

474
00:41:42.330 --> 00:41:44.370
Participant’s iPhone: It would it would encourage.

475
00:41:45.900 --> 00:41:51.150
Participant’s iPhone: The patient to get bit more, better less depression with
whatever's happening around us.

476
00:41:51.600 --> 00:41:52.530
reassuring.

477
00:41:55.740 --> 00:41:59.250
Participant’s iPhone: Having no visitors is you feel more depressed.

478
00:42:00.690 --> 00:42:13.560
Participant’s iPhone: And both sides of field, because your impact you're in hospital you're feeling depressed because you don't know what the situation at home and the family don't know what the situation is at the hospital.

479
00:42:14.280 --> 00:42:15.750
Researcher: So you have to be right yeah.

480
00:42:16.590 --> 00:42:18.840
Researcher: it's time for for family.

481
00:42:19.170 --> 00:42:24.750
Participant’s iPhone: yeah I mean if you just see somebody, even if they love 510 minutes, just to see the person.

482
00:42:25.200 --> 00:42:28.740
Participant’s iPhone: yeah it's reassuring and things would be a lot better.

483
00:42:29.430 --> 00:42:31.980
Participant’s iPhone: yeah but obviously that see your me thinking.

484
00:42:34.230 --> 00:42:40.920
Researcher: Thank you very much cylinder is there anything you want to add or I should still recording.

485
00:42:41.550 --> 00:42:46.380
Participant’s iPhone: No one i'm quite happy and i'm a human Maria my support Thank you

---

Researcher: So I can I please confirm your name and state your consent for our meeting to be recorded, please.

Participant: My name is Participant and I consent to this meeting being recorded and to take part in the research.

Researcher: Brilliant. Thank you so thank you again for agreeing to take part in this study, so today, we would like to discuss with you how have you been managing and dealing with life after your stroke. The ultimate goal of this study is to provide an insight into the needs and challenges people with stroke might experience during the pandemic and to help identify strategies to improve services. This meeting will take about one hour, so please remember there's no right or wrong answers for any our questions, we just want to listen to your opinions. And you can also stop your interview at any time, or, if you want to take a break in between, please let us know it's all your right to do so, it's all right. Thank you again so um, the first question will be about your experience of your stroke, can you please tell me about your experience of your stroke.

Participant: Right um and yeah I had my stroke on the first of March 2021 this year. And basically, I woke up on the first of March, and I woke up in the morning, and it has happened, basically. On the Sunday night I had some symptoms, so my arm, my head was very strange. And just felt strange but i've put it down to my vaccine because i'd have a vaccine earlier that week. I had my vaccine on the Wednesday, so I was blaming the jab in my arm. It was still affecting me and… My arm was a little bit strange, it was a little bit, it was almost like slow motion. It still moves, still did everything, but it was slow motion, then when I got up to go to bed I almost lost my balance, my balance wasn't quite right. And I struggled up the stairs we thought about calling an ambulance then, but as I kept saying, I’m just a little bit weird, what do you say, can we have an ambulance

because i'm a bit weird? You can't, so I went up to bed and went to sleep, woke up to go to toilet in the middle of the night and my arm was numb, I just assumed that slept on it.

Researcher: Is your speech or facial muscles still okay?

Participant: Everything was okay at this stage.

Researcher: Okay.

Participant: And in the morning, when I woke up in the morning, I got up to go to the loo as is what happens to a middle-aged woman, and I got out of bed and took a step to go into the toilet and the next thing I knew I hit the deck, I had fallen. And at that stage I realised that my leg didn't work. My leg wasn't working, my arm wasn't working. And when my husband said, Well, you know what's happened, he realised that my face had dropped. So that point, it was a 999 request.

Researcher: Okay so you went to the hospital with your husband, was it?

Participant: No I went to the hospital alone and that was the worst bit, my poor husband was stood on the doorstep, unable to, you know, he was told that I had to stroke, or they suspected a stroke at this stage and that i'd be going to new cross which isn't our local hospital, it's the stroke centre. And he was left at home with my daughter, and they were stood at there going, and he didn't know whether I was really ill or what was happening. I went off in the ambulance and, to be honest, I didn't really i'm aware, I was aware of what was going on, but I was, I didn't believe it, I felt okay. I didn't have a headache, I didn't feel ill, my body just didn't work, I didn't understand it, and when I couldn't understand why I was going to New Cross so they were trying to explain things to me repeatedly, so there was obviously something going on in terms of me understanding things.

Researcher: Okay, so at New Cross, do you know that is it ischaemic stroke, or is it a blood clot?

Participant: It was an ischaemic stroke.

Researcher: Okay and do you have any cardiovascular disease before besides this one?

Participant: um no not really, I have a faulty valve in my heart which only ascend when i'm pregnant, so I am not doing it again. So I had a hole in the heart as a bit as a as a young child, and then it only comes up every now and again when it's put into lots of stress, I suspect.

Researcher: Okay, so how long was your stay at the XXX Hospital?

Participant: Hospital at XXX, I think it was probably a week and a half.

Researcher: And they basically discharge you back home or?

Participant: No, what we have in Warsaw, we have a really good, you go into rehab rehabilitation, so I was transferred back to Holly Bank, which is the rehabilitation unit.

Researcher: And how long do you spend time at Holly Bank?

Participant: Seven weeks, I think.

Researcher: That's pretty long.

Participant: yeah I think they tend to have you for six weeks anyway, but the way that I, I think I arrived on a Thursday, the way it fell, I ended up getting the seven weeks.

Researcher: Got it. So what would you say about the physical ability at that time after you get admitted to the ward? I know you've mentioned your arms, your legs, and your face drop and understanding.

Participant: So yeah. When I was admitted to the ward, i'd still got a little bit of movement. So I could still move my foot and my ankle, not massively but i'd still got a little bit of movement.

Researcher: Was it left side or right side effected?

Participant: Left side.

Researcher: Okay, so it was a bit about the limbs, arms and legs, how about visual and cognitive side?

Participant: Nothing was really mentioned about my facial side, cognitively nobody ever mentioned the cognitive side until I went to Holly Bank and then I remember saying something, I don't understand things the same, but I think they just, they didn't really have a, how I was beforehand, so I could do just been a little bit, um they didn't know that I was, I was quite a bright person anyway pre-stroke so it was almost, like the cognitive, I don't think anything was particularly obvious.

Researcher: Okay, so what therapies did you have during the acute ward admission and Holly Bank?

Participant: When I was in, because new cross has got almost two wards within one ward so there's a hyper-acute and then there is an acute. So in the hyper-acute, it was very much, first of all, it was a lot of bed therapy, because the first couple of days, not a lot happened because I was having lots and lots of scans. When I came back from my MRI scan, I was quite distressed and quite upset because I had, not been left a long time, but I was waiting, I was sort of hanging around a long time.

Researcher: And a bit overwhelmed about all the thing.

Participant: Yeah it was really overwhelming, and I wasn't able to see my husband. Mmm me and my husband do things, we always do things together. We've gone through hard stuff before and we've always gone through it together. And now we weren't, we were very, very separate so that emotionally really affected me.

Researcher: Did your husband ever got the chance to visit you during the admission.

Participant: No, not at all, he saw me through a window. But even then that was almost not really supposed to happen.

Researcher: hmm and so that's why not until you transferred to Holly Bank you've got to.

Participant: Even at holly bank I didn't see him. We didn't see, I didn't see him until probably five weeks after my stroke or six weeks after my stroke, thankfully we've got things like, facetime and zoom and all of that, we can use all of those things, so we did a lot of face timing, but I needed to cuddle, I needed the physical, I needed somebody to hold my hand. And as simple as it sounds, that's what I really missed. And I needed to know that he was okay, and he needs to know that I was okay. And I needed to him to know that whatever we face now we'd be okay.

Researcher: Would you say that is the main challenge that you've experienced stroke during the pandemic?

Participant: Oh, I think so yeah yeah. I think so. I'm not being able to see people's faces, it's really hard when you're trying to understand people and you can't, you can't see their mouths moving. And as somebody, i've got autism within my family, I don't have an autistic diagnosis myself, but then i've never sought one, however, I think there is a certain degree of autism in me, most of my kids are so it's logical that there's probably there as well, but I don't need a diagnosis, i've never needs the diagnosis as such. But I found when I was in the acute ward particularly, I wish i'd sorted that diagnosis in advance, purely because, um, things happened to me that I don't think would have happened to me if I have been a known autistic, so a lot of the physical care is just done to you. And it's not, it doesn't involve you as such, because obviously there's not a lot, I couldn't do a lot. But it's just done to you, so you've got nurses touching you and all of that, and I found that very, very, very difficult. I also found particularly intimately, I also found things like going to the toilet, i've got big hang ups about toileting. And, and my theory was, dignity wise that's what should be the priority. I know it should be the priority, because I do a lot in terms of patient care and caring for people. But all the way through, this isn't just at the acute stage, but all the way through it is for the staff and for carers overrides what is best for the patient or the service user or whatever. So everybody's quite happy for you to be able to use bed pads, commodes and those sorts of things because they're easier for staff to deal with rather than work hard to get you to a toilet.

Researcher: So did they ever discuss with you, for example, like bad caring or commode before getting you to use it.

Participant: Not really, it was things like, I'd need the toilet so they'd say so, I'll get you a bedpad and and I would say no you won't. And I want, if you can transfer me to a bedpad and I know I can stand because i've done that with a physio. I knew I could stand, which meant that I could be transferred to a commode which actually means I could be transferred to a toilet so I had to argue my case quite a lot in terms of take me to a toilet. But I had to go through the argument every time and it was with every new nurse once you've got a regular nurse, they knew and they were like on there let's do this. yeah but I had to argue that and I had to almost go no, I want to go to the toilet. And I think that was one area that was that I found particularly hard, do I think that was just the pandemic? I'm not sure it was. I think that is hospital care.

Researcher: Overall care, I would say.

Participant: yeah I think the pandemic has a house because they're on they were physically and visually so tired, they worked so so tired and I was, I was very fortunate, because the pandemic had meant that some staff had been transferred, so the lady that was looking after me probably the most, was somebody who was used to working in a different department, because she'd be seconded to a safer department, shall we say, but she was because of her needs and she had been subconscious to work on the ward that I was on so she did quite a lot of working with me and actually she understood my needs, my personal needs quite well. So I found that really helpful and I think the pandemic helped with those sorts of things.

Researcher: Okay, we might cover the needs at a later stage, which is a good point and we'll come back to it later. So at this point of time looking back to the therapies that you mentioned, there was physios coming to you during the acute ward, what therapies you had, like physio, speech, or occupational or what kind of therapies, did you get?

Participant: I saw the physios probably a couple of times a day. And I didn't see a speech therapist because I wanted to see one, but I didn't see one and I don't remember an occupational therapist at that time, that was in the acute ward. When I went to the rehabilitation, I saw all of them.

Researcher: Okay, including speech?

Participant: Including speech and the speech therapist spend time working with me in terms of, in fact, all of them, the way that it works at Holly Bank is you come up with your targets, what do you want to do so, I wanted to have a shower, I desperately wanted to shower, so it was they work together to enable my targets. And it was a proper join up multi-agency team working together. And I can't tell you how incredibly they worked, they were phenomenal actually.

Researcher: That’s good to hear, like let's say the physios where they all present during the section or were they like give you a program and let you to do it.

Participant: No they saw me daily, except on the days when the fatigue takes over with life, but then they would come in, poke their head in and say how you’re doing, no you need to rest today. So I saw them probably daily, in fact I probably saw them the most because that's what I needed the most, that's what my targets were about. The OTs I probably saw once or twice a week, and speech therapist I probably saw once a week however in between the speech therapists and the OTS had put together, they have, almost like physio assistant, OT assistant, but they have assistants that do everything. And he saw me, Simon, the guy that worked with me, saw me, probably for at least an hour daily. In fact, when he went I was quite glad he’s gone, because I was wrong dry. He worked my speech and my hands, particularly on my OT stuff particularly.

Researcher: And how long did it take you to get independent overall?

Participant: I’m not independent. No I’m not independent.

Researcher: Okay, so yeah. It will be down to the challenges during pandemic, which you mentioned, about your husband, the fewer times that you get in contacts and under mask is communication difficulties.

Participant: Yeah, and I suspect, I mean in the pandemic, in terms of the physio and all of that, I didn't really see any other stroke patients, where it was all done in our rooms, or in the gym on our own. I didn't see anybody else so again the say was socially that I was affected. And that was purely because of the pandemic and the other thing is, is that stroke, the majority of the other patients were quite old, and the power network wasn't, which was quite nice.

Researcher: Cool, so I want you to think about the care you received overall and not only about the mobility side, is there any experience you can tell me about the care provided by NHS or the community service that you had?

Participant: Right, the care that I had with the NHS when I was in, it's been incredible the entire time, however, I would say, in the acute in New Cross, they don't have time to do the same level of care as they do in Holly Bank rehabilitation. But in the rehabilitation unit I just can't even fault it, I can't even tell you, if I said no to something, or can we try this way, or I don't like that, or something like that, it was never done again.

Researcher: Besides the thing that you mentioned, for example, the care dignity part, was there any other impact that was done on your care because of pandemic?

Participant: Oh apart of having a thing shoved up my nose daily, oh, so many tests. I have been tested to death (laughter).

Researcher: They do it on a daily basis or?

Participant: It wasn't a daily basis, but I reckon it was probably at least twice a week.

Researcher: And it's not pleasant.

Participant: No. And the other thing, I don't know when this is, you know it is down to the pandemic really, is it was done at sort of six o'clock in the morning, so that everything, they could get round everybody to be sent off to the lab before 10 o'clock etc. But it was very early, we always used to joke about it being the rude awakening.

Researcher: yeah it's not very good being waken up in the middle of the night.

Participant: No, and then having the thingdown your throat, i've got a terrible gag reflex. And he used to be the same nurse used to come and do, he was on a shift at that time and we used to really laugh about it, but at the same time, I can imagine, some of the old people be really grumpy.

Researcher: So besides all these like awful experiences, is there something or people that will make you feel helpful or easier to manage your situation afterall?

Participant: Oh there's so many people, particularly the student nurses. The student nurses were well and truly thrown in at the deep end, now whether that it was the pandemic, I have a feeling it was, but they were literally, they were doing a full nursing job and some. But they almost had the time to, spend that little bit of time with you talking about things and getting to know you really.
Because in my world, I mean i've been working all the way through the pandemic, i'm a teacher. And I've been working all the way through the pandemic so for everything to suddenly stop. And

then, being faced with this huge amount of, gosh my life has changed dramatically. It was almost like how do I manage this, how do I work out, how Participantfits into all of this and i'm quite a positive person. So i'm trying my best to make something, find something positive about it, and it was almost like, Okay, how we can do this. And actually it was probably the student nurse that gave you that little bit extra because of the time. Hmm, the holly bank, the rehabilitation unit i've got to say, they're not well funded because I I missed the sunshine, because I was in when we had when the beautiful weather was on and I couldn't really go out because they didn't have the extra people, obviously, if they took me outside, I couldn't go past any other doors or any other people, and so was quite complicated to get me outside.

Researcher: Okay, so they helped you to be mobile.

Participant: yeah.

Researcher: OK cool so let's go down to the needs and goals that you mentioned. Im terms of the needs, can you tell me more about your needs in terms of health care or rehabilitation services?

Participant: Well, in terms of needs, until I went into hospital on the first of March, I didn't think my needs were particularly great. However, I am i'm quite sure now that I do have specific needs. I think that care, in general, has been affected by the fact that they are exhausted. And that's, and that is, you can just you can almost feel. You can always feel their exhaustion you can feel they're, not bad morale, it's almost like they've lost their their shine doyou know what I mean, people have lost their shine. And that's what I think is a real shame is a lot of the people that we're working with me, I think, have lost their positiveness. So in terms of care, I think that people aren't particularly positive about everything. And I noticed that, because when they came out of the Nw Cross and into the rehabilitation unit, it was almost a shock for them, because they were really positive. It was almost like a shock to the system that they were positive and they weren't. And whether that's the difference of them in the hospital and the unit, it was very separate, the unit was in a totally different place, is sort of in the community type stuff.

Researcher: So you are saying the staff, maybe they are overwhelmed by…

Participant: Yeah, overwhelmed is a really good explanation, they felt like, they were just getting through each day, and they were not doing their jobs, they weren't certainly not carrying, because all of that was there. But it was almost like right. And i'm assuming that it's the pandemic to be fair because i've never noticed that before, i've worked in hospitals and i've never noticed that before. I went into the rehabilitation unit, it was almost like Wow, these are my kind to people.

Researcher: Yeah, they have the time, they have the resources to get it.

Participant: The resources maybe not. They definitely seem like the poor, it was like, everything seemed to take a long time to get to them. They didn't really have many, the equipment, they didn't have many of them so they were having to, we were having to share equipment and then everything was having to be wiped down in between. And you can see why people get exhausted, it's all the extra stuff that is being done. And a lot of them has had COVID previously in the unit, a lot of the staff that have got COVID initially, you know, during the first few months because of their nursing, because of what they were doing. And they worked really hard to make sure to ensure that the rehabilitation unit was a COVID-free zone. And they worked really hard to keep it that

way. And that was quite encouraging to me because the last thing I wanted to do, i've not had COVID all the way through, and I was like we're in March, I haven't had it and if I if i'm going to hospital i'm probably going to get it in hospital. I really didn't want it then, I mean, I have had it now to be fair, but because of the vaccinations that we've had that i've had the impact on me was really okay.

Researcher: Let's say that's the needs like what they do well for the care, but for yourself, for your needs, for example, like showering, eating, feeding in the ward, any kinds of these needs, were they fulfilled? like anyone helped you?

Participant: Well, I had in terms of the eating the food. For the first few days they insisted, I had to be on a mashy food diet and wasn't allowed real food. And I was like, are you kidding me, and so I had mashed sweet and mashed potato every day, it was like, I cannot please do not give me mashed sweet and mashed potato surely there is some other mashed foods, but no, they just kept giving me mashed sweet mashed potato. And then I asked for toast and they decided that was allowed toast and then after i'd had toast, they decided I wasn't allowed to. And so I was on the mashed for two more days and I think I was more days then I need to be. But food wise it was fine.

Researcher: How about going to the shower or toileting?

Participant: I wasn't showered, in fact, my hair was washed once when I was in hospital. And that was the nurse that I developed quite a relationship with, the one that had been seconded, but I just kept saying, I I was quite down, I was quite emotional and I was quite down over things and she was saying how about we wash your hair that make you feel better. One lady came in one night and said come on let's get these nails painted because I have quite nice nails, they're always painted and they weren't because obviously we'd been in lockdown and I wasn't able to have them done. And she said so, one of the nurses, one on a night shift painted my nails and that was really nice, so those but other than that there wasn't really a lot of anything else. I was washed, bed bath probably about four or five times, I hated every minute of that. I hated it and on one occasion, I mean, I remember being, although the other nurse was a female nurse, I remember a male nurse being there. And I wasn't given the choice of him being there or not being there.

Researcher: Okay.

Participant: I remember being, I I don't remember, whether it was him or not, but I remember being washed him in my private areas. Him being there, and whether he washed me, but I was too busy having a complete internal meltdown over the fact that i've got a male nurse down there, and nobody touched me down there other than my husband. And I hated being touched by people, hated by being touched by the nurses.

Researcher: Have you ever told them that you don't want that, being touched.

Participant: I don't think that I have the opportunity as such.

Researcher: Okay.

Participant: However, when I gone to the rehabilitation unit and I built up quite a relationship, I had a male nurse quite a lot, and we did a lot of laughing. And a lot of there was a lot of humor,

shall we say, we quite enjoyed working together, mainly because we just hit it off with very similar character type people and we joked quite a lot, and he did a lot of my showers. But I was asked where that was okay and it didn't bother me in the slightest because he'd lost his sex by then. It was just a person, it was almost different in my head.

Researcher: So regarding the rehabilitation part, so that was the care part, so for the rehabilitation part, we're there any goals that you had for yourself?

Participant: Yes, yes! I am a goal-orientated person. My first goal was to stand up. And I did that in the hospital. I was determined to do that and I did that in the hospital ward with the physio. But again, the physio team were just they were second to none, all of them were incredible. And I just said, I want to stand up, because I need stand up so that I can be transferred onto one of the pieces of equipment so I can move to the toilet and so. And he went, Right let's do it. So we did it, and it was really straightforward and he gave me some really good tips about my hip positioning and things like that, but just how steady me that I didn't need to rely on my left leg. I got strength in here, but I haven't got stamina in it at all. And he helped me work out to how to position my body which helps hugely and those thoughts or tips are really useful, not to compensate for things but just to make sure you're safe. Because I just said to him I don't want to put myself at risk.I have other pre-existing conditions, I have something called Ehlers-Danlos syndrome, so my joints are very hyper-mobile. And I have various things that mean that my body is already, let's just say, my body is already a little bit broken, so I had to keep reminding people that they weren't trying to get back to normal, what they think is normal, my normal was in this place here. And they just took it on board, they just they just seem to know.

Researcher: It's good you know your standard and you know how people adapt to you as well.

Participant: yeah that's my job so that's what I do is I help people to communicate those needs with other people.

Researcher: Okay, so at the acute ward, your main goal was to stand up right?

Participant: That's toilet on my own, be able to go to the toilet so that they could shut the door, and let me go to the toilet. Unfortunately, I was left for quite a long time, sitting on the toilet, however i've got, I went to the toilet, I cann't go on a bedpad. It doesn't work, I ended up catheterised because of the bedpads and that wasn't that wasn't nice.

Researcher: So um let's say that's the mobility side. After discharge from the acute ward, from the hospital, did anyone teach you any strategy to manage yourself, for example, physios was there any program?

Participant: Yeah I mean, since discharge, I mean it's worth saying in the rehabilitation, I set myself goals like I wanted to stand up, I wanted to do this, so I wanted to do that and the physio would almost, she got how I worked, and she would almost go, okay. I obviously set my target of this, soon as we reach that target, she'd be like, right what's the next one, how should we do this, she challenged, she just challenge me in a really gentle way, which was, we work together, really, really well. After discharge, well, what can I say, this is where I hope it's the pandemic because this is where it set down, really. I've had two different people, so the first lady I had, I already knew

her from the hospital, we had a really good relationship, but she didn't really… We talked about a lot, but we didn't do a lot, shall I say.

Researcher: Was she a physio?

Participant: No, she was a physio assistant. When she left, i've now had a physio, but we really haven't hit it off, it's much more directive rather than collaborative in her working so she says, Right, i'm coming in next week to do this, and it won't be anything to do with my goals.

Researcher: Okay, so does it mean that you got a physio visit to your home after you got discharged from Holly Bank?

Participant: Yeah. So i've had a physio come to the house, I had that many OTs you wouldn't believe. We had an OT come out and see me at home, but really it was in batches she'd worked with my husband prior to me coming home because the room that I had done downstairs is almost always been set up the wrong way around but nobody gave my husband any any guidance on that.

Researcher: So how long did you wait for all these therapies like physio or occupational therapists to come to you?

Participant: Straight away, I mean they came pretty much straight away. I'm quite a bossy person in terms of I know what i'm expecting and know what I should have. So I made sure that those things had happened pretty much quite, well, once I was home. The OT came home with me, so on the day that I came home, I came home in the ambulance and she came here in the car. And she just made sure I was okay, and I knew what I was doing and i've got no immediate needs.

Researcher: To set your home up basically to make sure you are safe.

Participant: yeah it's made sure I'm safe but they already ordered to me like a commode or a shower chair and they'd already ordered me, but I was using them in hospital, they just came home with me.

Researcher: Did the physio get you any program then?

Participant: Not as such, no I don't think so.

Researcher: So during this gap, like, after you got discharged from the bank till the physios coming to you on community basis, what did you do for yourself, did you do anything like to self-manage or to self-help in terms of mobility?

Participant: I'm, i'm… yes.

Researcher: What kind of thing?

Participant: I mean, I would, I didn't have a bathroom downstairs at the time, but the toilet downstairs and the sink downstairs so I did all my washing and everything in sitting on the toilet and in that little hand sink. We managed, it ran perfectly okay. My husband wash my hair over the kitchen sink, and we ended up buying various contraptions off Amazon trying stuff, we found one

that worked eventually. But my bathroom wasn't done for another three months, so we did that for quite a long time to be honest. But in terms of physio, I continued with the exercises the bed exercises, because I know they work. Plenty of walking, I was, they've already been out and put grab rails on there, or they were putting grab rails on quite quickly at the front of the house, so that I could get in and out of the house. We'd already practiced when I was in Holly Bank getting in and out the car.

Researcher: yeah okay that's good.

Participant: Because I texted the physio that's what I want to be out to do, I don't want to be sat in this house, in a chair or in a bed continuously. I want to be able to get in the car and go places. Okay, I might just get in the car and go places and come back.

Researcher: You think you are prepared to go home?

Participant: yeah I think I was, I was ready to go home. I missed the company from the staff. But I was ready to go home. Do I wish I had the physio and the community that I got while I was in the rehab unit? Yes. Physio that I have now, I haven't seen since I had COVID at the beginning of October, so I haven't seen her for over a month and they just give me some balance things to do, so i'm trying, i'm seeking out my own private rehab, because my hands starting to react now and it's almost like, you know what I need something else. While I was in the unit they hired a contraption, I can't even think what to name it, but they fasten my arm in it. It is not a regular thing that they have, they just tired it to almost test. And they thought of me straight away and thought i'll pull up, be really interested in this. I'm quite a driven person, so it was almost like they're see how far we can go with this, so it was about getting my shoulder movement and elbow movement, i'm glad they did because I now have that. Unfortunately, my hand doesn't quite work, but I knew that's the sort of thing I need, almost a functional thing, some kind of functional rehab so i'm gonna have to get that privately so.

Researcher: Okay, so, first of all, the physio, they didn't came up because of the pandemic to your home right? you said since October.

Participant: yeah no, my daughter got COVID at the end of, beginning of October, she's back at work and she works with the public, so terribly surprising. So, and then I got it straight from her. And although we cancelled the physio visit, she came out anyway, and obviously my husband said we've got COVID, no. She almost ran away. (laughter). But she hasn't been in touch since.

Researcher: Okay got it, so you're sorting yourself out, trying to go privately.

Participant: yeah I mean i'm gonna probably going to ring her again at some stage, but I didn't hit it off with her. And that personality that, I need inspiring and motivating, I need somebody to get in here and excite me.

Researcher: okay.

Participant: You know i've been doing physio for 40 years and i'm bored, i'm really bored, but you know i've got a pre-existing things so i've been doing physio for a very long time.

Researcher: Okay, so that was about physio, so other than that anyone taught you about how to do your daily activities technique, for example, like using one hand toileting, dressing, feeding and stuff.

Participant: No it's just it's pure trial and error.

Researcher: Okay.

Participant: we did talk about putting a bra on one handed, that was it really.

Researcher: Okay got it. So what we're saying is the self-management strategies here, which is more or less about how the therapists, the physio, occupational therapist to empower patients and to ensure your safety. Part of it is coming to your home to ensure that you're okay, and all the railings and stuff, but also how to empower you and all the techniques that you should learn. That also involve like goal settings, the action plan and all the decision making process, and so on, so do you think that you had it and was that useful or beneficial?

Participant: I think I had it, I think I should have it for the next two years. And well if I do I do, if I don't doesn't surprise me, I live in Warsaw. However, I think that I've almost lost faith in the system, but that's because of lots of reasons, not just what's going on now. I think i'll probably get in touch with some people if there's a problem, I now have a power wheelchair so, although I can get on my feet with my quad stick, see originally all we concentrate on was me walking because that was my target, I wants to talk. I can do that now, I can do that safely now. Do I want to use a quad stick absolutely not, I think they are the ugliest, most annoying things around. However, I know that a single stick wouldn't give me the support that I made because it's my balance, not necessarily my strengths that needs to support.

Researcher: So think back about that goal setting process, like with you, with the therapist or with yourself, like within yourself, for example, going to the toilet, getting out of a car. Are these beneficial to you?

Participant: Oh they are vital, absolutely vital, because I know what i'm working on. If i've got direction I know where i'm going. And I know what i'm working on, and I know what targets i've got, so it's just about providing direction really. The other thing i'm finding quite difficult to manage is that fatigue side of things. The fatigue comes with, i've already have fatigue from the pre-existing conditions that i've got so the stroke fatigue on top of that, is almost that's quite, that almost knocks me for sick. So it's almost like i've got to learn a new way of managing me.

Researcher: Okay, so that was about your experience so far. I'm aware that we're coming to the last bit of time.So the last bit will be about looking to the future, then. And if, when the health care services starts to reopen, what do you think are your priorities for rehabilitation?

Participant: I would like to get back to work. Although i'm doing a little bit at the moment, i'm having to change everything, and I think it's all about how to, my priorities will be about how to manage the new me. And like I said, the new improved me.

Researcher: You were all the way improving, we'll keep on going I suppose. So, would you be like expecting to go back to more frequent therapy section, more physio section, or more occupational therapy section, in terms of rehabilitation?

Participant: I hope it would be more OT, I hope it will be more physio. I was really like to be in a gym, i'd like to be able to use equipment to do things. Functional activities are really important, that's how your brain makes connections. Doing this with my hand continually is pointless because there's no functional ability, moving things with my hands is an activity, it's a functional activity and that's what I need to be doing, my brain needs me to do that. And I know about neuro plasticity, I know about these things, and I know how things work. And at the moment i'm doing more compensating with this side than I am functional ability with that side and I don't like that it doesn't sit well with me. So I know I shouldn't really need to explain that to physios, I shouldn't need to explain that, and I know I haven't in the past. But the new physio i'm really struggling with, she doesn't seem to get that, you know.

Researcher: So you were saying that you want more equipment in the gym, so would you say that you're quite limited in terms of equipment during the pandemic, or because of the pandemic?

Participant: I would say possibly not because i'm just getting it myself, i'm buying myself from Amazon, i'm buying myself from a different place. But it's almost like, they lack the knowledge I don't know whether, so in the past, I think there would have been space in people's lives to do the extra study, to do the extra research, reading about research, I think there would have been the extra space to do the extra stuff, if you know what I mean, that you do as a good practitioner. The pandemic because our brain is full of, I want to say rubbish, our brain is full of virus and masks, and when am I going to get my shopping, that I don't think people are having the time to sit and do more studying, go different training and develop that extra knowledge. And I personally i'm finding that quite hard, i'm also finding it incredibly hard that everything is delayed because my wheelchair was massively delayed even just a simple going out in a wheelchair, you know, a manual wheelchair. That didn't come for probably nearly 20 weeks since I was out of hospital, which was insane really cuz that I could have been starting my rehabilitation. My power chair, because one of my aims is to walk my dogs, don't even ask it's what I used to do, but i'm going to need my power chair to do that and actually that is rehabilitation too, to use your power chair is you, you need to be rehabilitated, you need to be taught how to use all of those things. But my power chair didn't come to the end of the late, the first lady that saw me out of hospital, the first physio assistant. And she took me out on the pavements around my own home, so that I was safe on the pavement. But actually, it's my daughter taking me out over the fields. i've got an amazing power chair, that is, we call it the beast, it's a bit of an off road type thing.

Researcher: I would take the points are like things can be faster because it's obviously delayed because of the pandemic.

Participant: yeah and my mobility car is massively delayed. The blue badge was really delayed because everybody had waited until the end of lockdown and everything is delayed. The blame is always COVID. At what stage is it going to stop being COVID's fault?

Researcher: And so, other than that, what else could be done, let's say two or three points at the most, that you think we can do better by the service if your stroke has happened in another pandemic?

Participant: The first one is find a way for family to come, I would say that is definitely a priority. That is what we found the hardest, but the other ones, are, I don't know i'd say improve patient care. But then. Actually that's pretty much i'd say it, whether there was a pandemic or not.
Definitely, the extra studying, and the time for this study, time for people's well-being, helping people succeed. So put in time for the well-being stuff.

Researcher: Okay, so yeah, I think I got everything. I am aware of the time as well, just not to let us overwhelmed by the end, so that's all about the interview part, thank you for everything and yeah I mean we might have one minute to have a break, and you can go to the assessment. Is there anything to add?

Participant: No, I don't think so, I think I said pretty much everything really.

Researcher: yeah that's very detailed and yeah definitely helpful to our information needed.

Participant: yeah I was gonna say, the only other thing that I would say is the care for the family. They had no idea what was going on for me, and they were certainly not prepared for me. My son, I mean obviously they're both autistic but none of them have learning disability, they are all aware, but my son spent the first, I don't know six weeks thinking, I was going to die. And we need better care for the families.

Researcher: Get them more informed as well.

Participant: yeah definitely.

---

audio_only DECEMBER 2021 SEP - DEPTH - 34 MINS

[Other comments:]

[Interview starts at 0:00:19.0] So [? 0:00:23.6], thank you very much for agreeing to take part in this study, to share your experience and thoughts with us. First of all, would you agree for me to record the session? Are you okay with the recording?

Yes.

Today we would like to discuss with you how have you been managing and dealing with life after your stroke, and we would like you to tell us about your needs for recovery and the care you have received from NHS and other rehabilitation services. Just to let you know, the ultimate goal of this study, actually, is to provide insight into the need and challenges for people with stroke during the pandemic time, and to help identify strategies that can help them to improve the services. This

meeting will take about 45 minutes to an hour, as we said. If we can do before that, then I will see how much we can do. I had your agreement to record the session. Please, can you state your name for the record?

Yes, [?Participant0:01:44.0].

We will not use your name again in any of our reports. This is just for the consenting, and then we won't use your name, clearly. You can stop your interview at any time, or if you want, you can have a break during the interview. Just let us know. Thank you again for your time. First, we will start, I will need you to tell us about your experience of having a stroke I think for your first time, right?

My first time, yes.

Tell us about your experience of having a stroke.

Where do I start? I had a stroke in March this year, 2021. I believe the reason why I had the stroke is because of the Oxford vaccine. I trained as a vaccinator back in February, March. I gave up my job to give back to society, and the opportunity came up for me to have the vaccine before I went to frontline, and unfortunately, on I think day four after the vaccine, I was very unwell. I went to A&E. They released me the same day, said, 'Unfortunately, it could be just the aftermath of the vaccine.' I think on day ten I ended up back in A&E, emergency, as I'd started losing sensations in my legs and I wasn't able to walk. I was reliant on my husband on doing everything around the house, and because they couldn't find anything wrong, I don't know what made them trigger, but they did a CT scan, and whilst they were doing the CT scan I was actually having a stroke.

Participant, have you had any symptoms or any complaint with your cardiovascular system?

Nothing. Absolutely. I don't have any pre-existing conditions at all.

Can you tell us also more about your experience after you were diagnosed with the stroke?

Pretty lonely, frightening, scary. I couldn't be with my family. Very emotional. I didn't know anything about a stroke at the time, apart from it only happened to older people. It has actually been a very emotional ride, to be honest with you, and I'm still very [voice breaks slightly]...

It's all right, Participant. Sorry for that. If you can tell us about how the stroke affected you and your daily activities, and before that, how long were you admitted to the hospital, and when did you discharge home after having your stroke?

So I was in hospital for seven days after the stroke. I suffered imbalance in my body, so I wasn't able to walk in a straight line; I had balancing issues. I've got peripheral vision problems in my left eye, and I'm left with anxiety. In terms of what I've got back from the hospital - the hospital that I was with have been brilliant. I cannot fault them enough.

Before we move to them, the care you have received, we need to know more about the effect of stroke on your other parts of the body, like on your mobility. You said you had an issue with your balance, right?

Yes.

What about walking and maybe walking speed?

So when I first had the stroke I wasn't able to walk. Yes, I could with, I call it a walking frame. I think around about day four I managed to get rid of it with physio and became independent. With physio, three months later I managed to get all my balance back, and being independent and being able to walk by myself completely.

Without any aid?

No aide. I have no aid.

No device, or no cane, no...?

Nothing. Absolutely nothing.

That's good to hear.

Yes.

Participant, what do you think was your main challenges during that time?

The first period, I suppose the first few months the challenge was not understanding the impact of a stroke. Everybody talks about the physical, but nobody talks about the mental trauma it leaves you with.

So that probably was the main challenge that you experienced after having the stroke...

Yes.

...not understanding the situation after you got a stroke.

No.

Participant, let us move to the care that you have received. If you tell us about the care provided by the NHS or other community services after discharge from the stroke unit.

So like I said, I cannot fault the hospital at all. I suppose if I had one issue it would be - well, two issues - one is I know, because of COVID time my family wasn't able to come and visit me in hospital. I suppose, if I had to go into more detail, the second issue would be the challenges of being a young stroke survivor in a hospital ward where you were surrounded by older people, because your body is working at a different pace, and you're not understanding what's going on and why. Also, day communication from the nurses and the doctors was fabulous. The challenge would come at night time, because I often found myself, whether it could be the hallucinations that I suffered after I had the stroke, but I felt that I was being talked about by other nurses and doctors at night time.

Would you see this as something that current healthcare people need to take care of, maybe?

Of course. Of course, because to go through it, someone like myself who's young, and to have an older person in the same ward being treated differently, and you're seeing visually how these people are treated, it's completely - for me, I couldn't stand the sight of how they were being treated, because they couldn't move. Whereas, there's me who's fighting to get back, and get my independence back. You get treated the same is what I'm trying to say to you.

Participant, let us talk about the most important thing that we need to get out of this interview, is the rehabilitation services after you were discharged from inpatient or acute care services, when you moved home, or when you discharged from acute care unit. Can you just tell us your experience with the rehabilitation services?

Yes, physio was absolutely brilliant. It was all done online because of COVID. I wasn't able to go in.

This is after you moved home?

Yes, at home. It was all done online. Like I said, I was determined, and I did the physio myself as well, and I've managed to get my independence back. I suppose the other challenge would be, I would say, the mental health service for stroke survivors.

At home, after you discharged?

Yes, because I've not had any support mentally, even though I've reached out to my GP, and I'm waiting for a consultation with a counsellor, there's a long waiting list, and I'm expected to wait eight weeks. I mean, I'm very lucky that I'm quite strong, and I'm able to pull through the dark times, but I can see a flaw in the system, if you call it a flaw; if someone's not as strong as me, to fight this, it could be detrimental on their life.

Participant, how would you see that, like the emotional aspect of your impairments affecting your maybe physical rehabilitation? I'm glad that you said you didn't have much physical impact of stroke, and you were able to recover.

Yes, apart from my visual. Yes, so I was still suffering with my peripheral vision. I'm not allowed to drive. That, again, is a waiting list. I guess people heal differently, so I'm still healing. I've been referred to an eye specialist, but again, there's a waiting list for that.

Have you experienced any sort of fatigue or pain during your recovery period?

Stroke fatigue is immense. I can have a really good day like today, or I could be having a really bad day, where I really can't get out of bed. I'm trying to work out my triggers. I know some of them, but I'm still trying to work it out.

Participant, let us go back to talk about the rehabilitation services that you have received from the NHS. Do you think that that was enough for you as rehabilitation services after your discharge?

For physio, yes. Only physio.

Only physio was enough for you, but you think the other thing, you still need more support.

Of course.

So can you tell me about the things or people that you feel were helpful to manage your situation during that time, after you were discharged?

After I was discharged?

Yes, because you were talking about the medical staff during inpatient, but after you moved home, who do you think were the people who were helping you, either family, or maybe medical staff, or maybe physios, or other people who are helping?

I'd have to say my friends and family; they really stuck by me, and Samantha, who's the physio lady, she was really supportive, and also my GP, who has been very, very supportive.

Can you tell us a little bit how family and GP and friends were helping, what sort of help they were helping?

Emotionally. Keeping me going.

Well, this is a very important thing, but do you think they also provided any sort of, maybe, like physical help, like if you...?

No, because I'm very independent, and I was determined that I wouldn't let anything bring me down. I'm a very determined lady.

So we go back to just summarise, what needs do you think were not fulfilled during this time? You said maybe you are still waiting for some maybe services to be booked for you.

Yes.

Can you tell us a little bit about those needs that you still feel are not fulfilled for you?

So the physio is a tick. What I'm waiting for is some help with my peripheral vision to strengthen it, so I can become completely independent again and be able to drive, and emotionally, I think not many people can understand my story of being a young 44-year-old independent lady who gave up her life, her work to become a vaccinator to give back, and to be left in a situation where I can't help my family financially, or even give back volunteering to society, because of the Oxford vaccine. Financially, it's been a burden on my family, because I cannot claim financial benefits for some reason, and it's a very hard situation to be in.

We are sorry to hear that, Participant, and hopefully, things will be sorted out for you.

Well, hopefully, and please don't say sorry, because for me, I'm learning every day, and every day is a school day for me, and that's all I can ask for.

This is good to know. I'm sure you will be able to actually overcome this time. It is shocking, maybe, having your first stroke, but hopefully - and I'm sure you will bash through this and you will be stronger enough to bash through this. Participant, can you tell us more about your rehabilitation? What did the healthcare professionals and the NHS staff teach you or provide you

with as a sort of rehabilitation, or any interventions that they prescribed for you, even if it was online?

So the rehab that I received during the hospital was brilliant. They really made sure that I was able to function in my own home.

This is before you were discharged?

Yes, before I was discharged, and after my discharge I really only just had physio. I'm very lucky that I've been able to get away with a small scrape.

This is good to know. Can you tell us more details? What have you received in terms of rehabilitation therapy, maybe?

Physio. That's it.

What sort of physio?

So physio is more getting my balance back, so it was more exercises to help gain my balance in my body back whilst I was walking.

What else?

That's it.

They focused only on balance?

Yes.

Do you think you were able to manage your rehabilitation therapy for balance at home, or there were maybe some challenges of delivering...?

No, the lady who dealt with me was brilliant. She set me weekly challenges; every couple of weeks challenges. We did everything online. We stayed in touch. So, no, on that front it was absolutely good.

Were you able to be involved in maybe setting some goals for your rehabilitation, or maybe...?

Yes, that was set whilst I was in hospital.

But after you go home - you went home, nobody visited you, or nobody provided anything at home; it was only line. Nobody visited you physically.

Yes, because it was COVID; it was lockdown, so nobody could come out.

Other than maybe not having you visit the rehabilitation centre or maybe the clinic, what do you think was another effect of the pandemic when you were in rehabilitation, or healthcare that you received?

I can't fault it in any way. The hospital were brilliant. The only thing I could say is my family weren't allowed to visit, and I understand why.

This is the only concern, or the thing that you faced. Other than that, everything was fine?

Yes. I mean, the NHS staff actually fought with my husband, because my husband wanted to move me to private, but the NHS staff really battled with my husband to keep me with them, and I'm so glad that they did, because I've come out seven months later fighting stronger.

Participant, there is something called self-management, where you can manage your emotional, your physical problems at home for yourself. It is actually encouraging and empowering people to work with themselves at home, and taking more of the care, instead of having maybe healthcare professionals delivering interventional therapy. Do you think your rehabilitation at home and only receiving online therapy, have you experienced anything, or did they mention anything, they maybe provide you with anything that you felt this is self-management?

No, but I have been self-managing myself.

But did anyone tell you that this is self-management and you have to do this and this as a strategy for your self-management?

Not that I can recall, no.

As part of self-management strategies, Participant, that professionals taught you, or maybe told you to do at home, can you tell us, please, what else have you been doing to manage your situation since the onset of your stroke?

Setting myself daily targets. Really pushing the boundaries out myself.

This was all by yourself?

All by myself. I've not really had much of somebody driving me and pushing me from the NHS. I've been driving myself. I've been also using holistic methods to drive myself, setting myself targets to walk, and become independent again. That's all been self-reliant.

Can you tell us a little bit the details about those things for your walking, what sort of goals, or what actions you have taken to improve your walking?

Yes, so two weeks after I came home, after I was discharged from hospital, I had my best friend and my sister-in-law come round every day, take me out for a walk, and we, literally, would go - because I could not walk really far, we'd literally just go around my road. I would say it would probably be about a 20-minute walk to initially start with. Every week we'd then increase it to see how we could do, because I'd already said to me friends and family, 'I want to be able to walk independently again', and hit a target of three miles, which I am doing now. I'm hitting three-and-a-half, four miles a day.

This is good. Participant, was walking the only, or maybe the main goal of your rehabilitation after stroke, or there were other goals?

No, there were other goals. I wanted to get my independence back. I cannot stress enough that I - before my stroke I was a 120 per cent independent Asian woman, and I wanted to get that back, to prove to my daughter that anybody can do it.

Participant, if you can just tell us, what does independence mean? Is it walking by yourself, alone without help, or...?

No, it's not just about walking; it's about being able to get back into society, being able to motivate others. It's being able to get back for other people. It's being able to be a mum; it's being able to be a nurse to your daughter, being a wife and a cook to your husband. It's everything in society.

How about independent for yourself, like to do your activities alone at home, or maybe to do like everything, shopping, maybe cooking, your daily activities at home or outside the home?

Yes. Yes, that is all part of independence, being able to do stuff for yourself, and not relying on anybody.

Do you think this sort of making yourself independent as much as you can, and working with your - maybe doing self-management for yourself or different things that you wanted to get independent with, do you think your role of self-management was very effective and you were happy with that during that time?

I'm proud of myself to be where I am seven months on, because I hear horror stories of people who have gone really downhill. I'm in a group of a charity online, and you see how they are. Emotionally, they're struggling, and I'm so glad that I've got the support and network and I'm strong enough to fight and get through it.

If you have anyone who recently had a stroke, would you explain or maybe advise him or her to also these self-management strategies?

Of course. Without a doubt. Look, stroke is a horrible thing. It leaves you in a really horrible mental state. I understand, as people who are not able to get out those dark places, that a tip for a stroke survivor is set yourself small little steps, and achieve those, and then increase those little steps to something else, and just go for it.

But, Participant, this seems holistic, like a plan or something for your different aspects of life after stroke.

Yes.

How about physical recovery, or physical rehabilitation after stoke, would you also advise people to do self-management for their physical thing?

Yes, isn't it the onus on the person individual to make better your life?

But how can you see the role of healthcare professionals in this, while you are taking care and doing self-management for your physical?

I think there needs to be more regular check-ins from, perhaps, GPs, for stroke survivors. Especially from a learning curve, my GP has been brilliant on the days where I've hit rock bottom. I've been able to go to my GP and speak to him and he's listened. Where, I know some people may not get that support from their GP or NHS personnel. So if you're going down the self-management route, then somebody has to govern and make sure that person's okay to do that independent work.

Let us think about future care for you and for other stroke people. When the health services, hopefully - and I think they reopen now again after the pandemic, and hopefully, there will be more released in the near future. So what do you think are your priorities for your rehabilitation?

I have none. I'm living day-by-day and enjoying life. Yes, I've been through every bit of testing that they possibly can give me, and they still haven't found anything, so my investigation is still ongoing.

So I have another question about how can we ensure that the services are tailored to people affected by stroke in the future.

You can't. You can't tailor it. It's bespoke to one individual. Each individual is different. Each individual has a different need, so I think each case has to be looked at separately.

Well, Participant, this is a general rule for managing stroke, but my question was about managing the services for stroke during the pandemic. If, let us say, another wave came, or another pandemic - hopefully not, but let us think that way - if another lockdown happened, then how do you think we should manage services for stroke people?

Like I said, you really can't, because my disability, it's my hidden disabilities are different to somebody else's needs. I don't know how to answer that question, because, like I said, my needs are different. If it was me in that situation, I would really want more mental healthcare support.

Yes, you are right, this is like a general thing in managing stroke cases, that people with stroke, they will have different impact and they will suffer different impairments of stroke, but I was thinking, for people who are having maybe similar impact or challenge after stroke, during the pandemic, how we can manage their services, or how we can deliver their rehabilitation, and how we can enhance the way of self-delivery for stroke people.

I really don't know how to answer that, because I've had all the support by online physio; I've had my GP.

You feel that was enough for you and for...?

For my needs? Yes. The only lacking that I have is the eight-week waiting to speak to a counsellor for stroke. So mental health is a big thing for me.

Is there anything you want to add for this interview about your experience that I have missed to ask about? Is there anything you want to add about your experience or your need during the pandemic time?

No, I suppose it's unprecedented times. You just have to work with what you've got at the time. Hopefully, we don't have another one.

Hopefully, yes.

Yes. No, I've been very well looked after.

Well, thank you very much, Participant, again, for your inputs during this interview. Shiva, do you want to have a go?

I2: Yes. Participant, thank you so much for sharing. It was really, really important to capture your thoughts. You've had a really horrible experience, but it's great that you have shared, because it gives us a better understanding and the collective knowledge would inform future professionals, so thanks a lot for sharing.

That's okay.

She has her story on different stroke websites published. She has written her story. She shared that with me.

I2: Oh, brilliant. That's amazing.

Have a read of it.

I2: Amazing. You're such a giving person, Participant. You're so generous.

Oh, thank you.

I2: You're so generous. Participant, I just wanted to ask you, you know the group - XXXX, this is nothing to do with the interview. Can you stop recording? This is just...

Sure.

[END OF TRANSCRIPT]

Researcher:
Thank you very much, Participant. First, thank you very much for actually considering your participation in our research study about what your experience with stroke during the pandemic time. In this meeting, we'll be discussing how have you been managing and dealing with life after stroke. And what sort of rehabilitation services or therapies have you received? And how was the

delivery during the pandemic time and the ultimate goal of this study, actually, to explore, and to also explain the situation of physical rehabilitation during the pandemic time. And at the beginning, before we start, can we have your name, and also your permission to record the interview for this meeting?

Participant
So my name is Participant. I'm a patient of the stroke survivor. And yes, I give you my permission to record for the purpose of this interview and I understand the research.

Researcher
Thank you very much for this. And just let you know, this interview will take about an hour. This will include your interview, and then we'll have a couple of assessment just to see how you are doing with your life after stroke, to explain and to describe our participants who participated in the study. You have your right to stop at any time if you feel tired, if you don't want to continue, just let me know, so we can stop at any time, it is your right. Also there is no right or wrong answers, we just, actually, want to know your opinions and your experiences with the rehabilitation after stroke. So let us first start and thank you again for participating. We'll start now.
Can you please tell us about your experience of having a stroke? And I think it was your first stroke, right?

Participant
Yeah, it was my first time, I'm hoping was last, but you never know. So yes, my stroke was on the third of May this year. That was Monday, was a sudden out of blue, nothing predicted my stroke. I wasn't any unwell or I didn't have any unusual symptoms, or feeling unusual, it was a normal day. So also, when I had my stroke, it was an unusual symptom because I'm quite aware about stroke and the FAST. The FAST because we see everywhere in billboards, adverts in red, your TV. So I'm quite aware about the stroke. So it started with suddenly losing balance, like something hit me in my head, and I was like, oh, what's that? And I said to my husband, because we were actually go for shopping. I said I just need to sit down for a moment. And my husband said maybe you'd stood up too quickly, sometimes you feel a bit dizzy.

Researcher
Yeah.

Participant
So I went to living room and I sat in a sofa. And I said to my husband, I think I'm okay now we can go and I stood up and again I had a balance problem. So my husband just like, sit down for a little bit longer, just have a longer moment, few minutes more. So actually I lied down, and then after a few minutes when I looked at my husband, I noticed I've got two husbands, because I had double vision. And when I put my hands like that I saw two hands as well.

Researcher
Yeah.

Participant
And I said: Is something wrong? Because I see everything double. So at that moment, I thought it's something wrong with my contact lenses, because I'm wearing contact lenses. So I thought it

might be something, you know, something is wrong with the contact lens. And so I said: I will go to bathroom and take them off. But when I stood, I pretty much couldn't walk properly, I was still having balance problems. My husband hold my hand, and we went to bathroom upstairs, and I took the contact lenses down. But I still have the same vision problem. I said, Let's go down again, I will sit for a moment on the sofa. When I looked at the stairs, the stairs were going funny. And they said to me I won't be able to walk downstairs. So he hold my arms and to be somehow we managed to go downstairs, I I lied down a little bit, I had the blanket, and I didn't have any pain or headache or anything.

Researcher
But have you had any history of cardiovascular problems before? Did you notice any symptoms before that?

Participant
No.

Researcher
Okay

Participant
I had vertigo before, vertigo was different. Because when we call the ambulance, I said to my husband, I can feel there is something wrong. So ambulance arrived within 10 minutes, and ambulance crew they thought… And when we called the ambulance, the person on the phone asked my husband if I'm able to speak to them. They asked me if it was stroke, and I said no because I knew the stroke symptoms is the arms, speech.

Researcher
And you didn't have any.

Participant
And I didn't have any. So I'd definitely noticed that. So when the ambulance crew came, they said maybe it's vertigo. And I said, well, maybe you're right, maybe not, because I had a vertigo before. I know vertical is different, it is also balance, everything was spinning around when I had vertigo. So I know vertigo symptoms so I said no, it's not vertigo. They took me to hospital. And in hospital, when I was admitted to A&E, they're quite young, I think a junior doctor saw me. He asked me to do this, do that, with your tongue, with your leg, with your arms. And at that moment, my vision was really bad, really bad. What I mean is, both eyes couldn't work together. If I covered one eye, I could see both eyes together. When I was looking right side, like right corner, everything was like, I felt chaos in my eyes, and at the same time chaos in my brain. It was like everything was spinning and everything was a total mess.

Researcher
Yeah, yeah.

Participant
So I said to the doctor, it's is going like that. And he just looked at me, asked me to look at this side and that side. And he said: Can I just disappear for literally 10 seconds and I will be back? I said yes, please. If that's necessary, then yeah, do it. He left and came back with someone else, another

doctor. This doctor introduced himself as a neurologist and he literally grabbed my bed and ran. And at this moment, I felt it must be something wrong because they didn't wait for porter, usually porter takes patients and the beds to move between wards. He literally grabbed my bed and ran. And I had a CT scan, and again, after the CT scan they grabbed this bed again and ran. I only noticed when we were running through the corridors it was stroke acute ward. I thought that’s odd because I don’t have stroke. They placed me in a bed, and this neurologist, doctor, came to me and he said we just proceeded with a CT scan and you had a stroke. That was something like, shock, and like someone hit you with the hammer right in your head. So, he said, can you quickly tell me, because obviously that's a time now, what you were doing when it happened and when you called ambulance?

Researcher
I mean, timing is very critical. Yeah.

Participant
Yes. So it looks like CT scan was proceeded within four hours. And he said, we're in good position, but we need to very quickly proceed with this. Because he said, I've got at the back of my brain and had this big blood clot. And he said, Who is your next of kin because we need to let them give an official permission to start proceeding with this. So yes, obviously, I gave my permission, please call my husband and I only had doctors spoke to my husband and suddenly a lot of people around me connecting this and that. They were very polite, like smiling and don't worry. And I only heard this doctor calling my husband and saying your wife had stroke, she had a really big blood clot on the back of her brain. As we are expecting a second stroke which will be probably worse and condition may deteriorate. If she survived this night, that will be a miracle. That’s what he said to my husband. And whole night they were proceeding, I was connected, and they were doing pumping, and well, that was thinning my blood. And whole night, I was lying in a bed and just checking my hands and legs if I can move because obviously I was aware about stroke, one side or I will be paralyzed. And I was like moving, constantly moving my hands and my legs. Checking if I still like can move because yes, I was waiting for the second stroke. And I thought this is impossible because obviously, why? Why?

Researcher
You were moving okay, so you didn't have mobility or movement problem.

Participant
But I was still really bad. So if someone was coming to see me and talk to me, I have to like, cover my eyes. And I said, Can you move to the left side? Because yeah, if I tried to look, I hurt again, it wasn't physical pain, but the chaos I had in my brain, it was just unbelievable.

Researcher
Sorry for that. And, uh, tell me about that night. So how long did you stay in the hospital after they applied the subject and thing.

Participant
A week. Next day, I was moved to stroke rehab.ward, and I stayed there for a week. So next day, I had a an eyepatch, hovering my left eye. So when they were covered, I can, like, see properly because as I said, both eyes didn't work together, and I was okay. Which is apparently when they covered my eye, I tried to Google because obviously that’s what we do. We Google and I tried to

read and I found out that is diplopia. So the next day, I saw a consultant that was a different person, different from the neurologist who admitted to me to this acute stroke. It was different specialist, stroke specialist. And I said: Okay, doctor, we need to talk about this diplopia because obviously, I'm quite concerned. So I didn't have any physical impairment. I didn't have any hands or legs paralysed.

Researcher
At that time or till now you don't have any?

Participant
I never had this

Researcher
So you didn't have any physical act of a stroke.

Participant
No.

Researcher
Okay. That is good.

Participant
Yeah.

Researcher
I think you were lucky.

Participant
Yeah I think I was very lucky. Because I think, well, because the time obviously, we reacted very quickly and from the ambulance, but also when the next day I saw this consultant, he said he doesn't believe I had a blood clot in my brain. And he, he said he wanted to proceed with a MRI scan. So the next day, I had MRI scan, and MRI scan showed it wasn't a blood clot. I never had a blood clot. I had bleeding in my brain. And the issue…or not issue, the first doctor… no the neurologist was mistaken. It wasn't his mistake, it was easy to be mistaken. Because I was born without some vessels in my brain. So every human has two main vessels covering through your spine, and then when it reaches your brain, it goes, the shape is like that. Yes. Like that.

Researcher
Round shape.

Participant
And from this two vessels, the veins taking the blood and feed your brain. In my case, I was born with the two vessels going up through your spine, branching your brain and stop. They never grow the bits, the branches never grow. However, my brain is clever brain. Well, I think not mine, like everyone's. And the small vessels are small, not the vessels, but veins took the function and my brain is still fed nicely with the oxygen and blood. So that's why doctor was mistaken on CT scan, he thought it's blood clot in here because there is nothing. Yes, grey area. And he thought that's the stroke and that's the blood clot. So I was also lucky and unlucky, I didn't have blood clots,

so maybe that's why I wasn't paralysed. It was bleeding.

Researcher
So that affects your vision. I need you to tell me how that affects your daily activity when you move to the rehabilitation ward? How did you feel like your daily activities doing, maybe like your routine activities? How did you feel like the impact of stroke on your activities?

Participant
So obviously, the first weeks were quite difficult, because no one could tell me if diplopia will revert if will if will not, Because the brain is, although we have this very smart machines, MRI scans, and all the you know, other stuff, still the human brain is, I think, still quite a big mystery. And at that point, no one could tell me how fast or not fast my brain will recover, if ever this part will recover. So they say don't worry, because if for example, diplopia will stay for life or for longer and little longer, you can wear parts or you can wear special lens. Because that's what patients and that was a little bit complicated.

Researcher
But how do you feel now? Moving like, after a couple of months of your stroke, how do you feel your vision?

Participant
It is coming back to normal, kind of, it starts reverting itself. I was wearing this pouch and one morning I woke up and I thought I just take a little peek to see if anything better. And then I thought I don't have this huge mess in my eyes like I had before. And he said, Oh, let's have a look. Let's check because then I thought, well, your psychological perspective or like, you need something you want something and you think, yes, it is better. Yeah, maybe it's not. So the same time I was happy, but I didn't want to put false hope, this kind of stuff, because then you will be disappointed. When Doctor asked me to take the patch off, he took a guy in the store and checked eyes and he said: I think it is a little bit better, it’s not completely, it's a little bit better. And then he said: Stop wearing pouch, stop wearing patch, start training your brain to learn and be trained again, so the nerves and all the functions got to be trained again. And I stopped wearing the patch, but my balance was still quite bad, and I think I had a little bit headache when I took the patch out, but that was normal.

Researcher
Did you seek any help to control or to improve your balance?

Participant
I had quite a lot of people seeing me in hospital and after hospital. So I had physio therapy people coming, so we had some sessions.

Researcher
It started during the rehabilitation time when you moved to rehabilitation ward, right?

Participant
Yes, yes. Straight away. Yeah, right away I had massive support. I was pretty much and I'm still overwhelmed, because from the first second, constantly people were coming to me, and I mean, professional. And they were doing a lot of stuff, they were determined to help me. And consultant

said, we will do everything to put you on lights and to come back to normal life.

Researcher
Yeah good, well, Participant, let us go back. During that time, I think it was, maybe lockdown or something because of the COVID situation, did you notice any challenge, like, did you have any challenge? Or maybe you feel like you were unfortunate because of the COVID-19 situation?

Participant
I had when I started all the rehabilitations. I've been also told when I was discharged from hospital, they referred me to vocational rehab services. And they said, expect them to contact you straight away when you come back from hospital. And when I came back when I was discharged, pretty much I think, two or three days after, I received a letter and the letter said due to COVID, obviously, the backlog is huge. And we are aware about your situation, your stroke, and we try to obviously do our best. Disregards this NHS. So I thought, okay, probably I won't see them. Because I understand due to COVID the backlog is huge. The waiting list is massive. And I'm probably not priority for them. Even if I am priority, I'm at this moment, I'm at the end of the queue because people are waiting. And surprisingly, I think week after or maybe 10 days after I can't remember exactly, obviously, but that was…end of May. Someone contacted me, phoned on my number and introduced herself.

Researcher
But do you think they delayed your service because now you were in good shape than other people regarding your physical condition?

Participant
No, that was what I thought what would happen but no. The service contacted me straight away. As I said, end of May. And I started my rehabilitation in June, July. So I wasn't waiting extremely long. And they were in constant contact with me by phone and email like updating checking on me.

Researcher
Do you remember what time you started in July? Was it like mid-July, right?

Participant
20th of July. And six-weeks rehabilitation I had.

Researcher
What sort of rehabilitation like? Did you have a home therapy? Or you were going to the hospital or rehab center? Or what was the kind of rehab center?

Participant
It was rehab center, I was going. And that was a session within a group of four people and two therapies.

Researcher
Every time you get a group of therapy?

Participant
Oh, yes, yes. And it was extremely helpful. And they also helped me with the process of coming

back to work because I decided to come back to work. And I came back on face return, and they helped me because they contacted my health department and suggested only four hours. And they also prepared plan for my return, so I need to have time for resting, obviously, when there was the rehabilitation, I can go without any acrimony from the work side. So pretty much they…

Researcher
Was there any home program they described for you like any exercise, or any therapy to do at home?

Participant
Yes, we received. I mean, we as a group, we had every week, we had a kind of program, which was printed for us with suggestions, and also the help if we need. And we had also all accessibility, I mean, if you had to do something at home, you're not sure there was always person available.

Researcher
Okay. So you can contact them at any time.

Participant
Absolutely. Although I finished this therapy, a few weeks ago, yes, few weeks ago, I still have contact to those people. And I saw this lady, one of the therapists last week, and she said, if anything unusual happened, I want to come back to therapy, I can refer myself to them. Also, if I have any questions, any doubts, and I need any help…

Researcher
Have you had any like home visit or home therapy? Or they just were explain, like, requesting you to do at home? No one visited you or maybe…?

Participant
No, no one visited my home.

Researcher
Have you done any, like zoom meetings or online meetings with your therapist, or just was my phone whenever you need to?

Participant
I had the zoom meeting with other therapists because I had a few therapists. And the other one I had also and that was straight away when I left hospital. I can't remember the exact date, but that was pretty much straightaway. And that was over zoom.

Researcher
Okay. And how did you… How was your experience with having some of your therapy plans delivered by zoom or by online?

Participant
I didn't have any problem because obviously since COVID started, Zoom and Teams became a main contact for people. So I'm quite aware or quite used to it, because at work we are doing this.

Researcher

So it seems like you were confident with that way of delivering the services.

Participant
I didn't have any problem. I was thinking if I have to wait for one-to-one or like physical contact with someone and not having this or delay this. I really appreciate what I had.

Researcher
Sure. And how was the group therapy? Like, what is your thoughts on that? Like, how was your experience with doing therapy with other people in a group? Was that encouraging? Was it like what was your experience with that?

Participant
So first, when they referred me and when they contacted me, I was very skeptical because I, to be honest, I didn't believe in any therapy. But obviously when you want to come back to normal life and you want half your life back, your stroke life, you do everything. So I said yes, I will join this therapy. And it was amazing experience because I met people like me.

Researcher
If I go back, why, like why did you believe that? Was it based on a previous experience or, like, why did you say that?

Participant
Because whatever happened on therapy. Yeah, people, I mean, therapists, they tell you things, you know, you read in books, or I learned at university. So pretty much you think you know, everything. And also, I don't believe people who didn't experience something like me. So if someone, you or someone else will tell me now, I'm sorry, I need to understand you. I will ask Researcher, When was your stroke? And you will tell me, Oh, I didn't have to tell you.

Researcher
But even if they are therapists, or doctors, or like somebody who are taught about your, your health condition, you still have…

Participant
Yes, you don't have choice, and you have to trust someone because you're in a position, you're in a big pool, excuse my language. And you, you have to trust someone. So you do trust that they doing. If you say doctors, this medics, they physically doing something because they, let's say, they pump some medicine, they connect machines, they take your blood, so you're doing physical things, if comes to other side. This therapy is not… You think you know everything, and you don't need those people. And when I joined this therapy, that's why I was a bit skeptical. How? How they can help me? They're only therapists, you see, that was a bit of ignorance from my side. How they can help? They are not, like real doctors. They are doctors, different kinds of doctors. Well, so I said, Okay, I will join. And that was amazing. Only because yes, it was in small group, people experiencing something like me, so we understand each other so we encourage each other. And those people, the therapists, were kind of guides for us, leading us. So we know what we feeling. They said, We didn't know what you feel, we don't know how you feel, we only know from, you know, like you read something in a book but before you only read, you don't experience. It's like with love. You can read everything about love, before you experience it, you don't understand that. So it's about feelings. That's why I say when people say, Oh, I'm sorry, Participant, I fully

understand, you know, you don't because you didn't understand. It’s only a word. It's nothing you experience. It’s thank God, you didn't experience that because I don't wish anyone to experience something like that. But I don't want people only saying sorry. Because it's kind to say something like that. No. That was this kind of me towards the…

Researcher
This was your feeling towards that. Yeah, I get you. So, Participant, let us move a little bit. We want to talk about impacts of stroke on your mobility, exactly. Mobility and walking. So you said physically you were okay. Maybe the diplopia problem and vision problem were maybe the most striking problem after and that maybe affected also your balance. Have you received any therapy for your balance? Or for your mobility? After your discharge?

Participant
No. No I think I didn't need to because when diplopia started reverting, the balance also started getting better so I think I didn't need therapy in terms of balance or mobility because I was fully fit. Maybe not 100% but still better than other people.

Researcher
Yeah. Yeah. Like you can manage yourself maybe?

Participant
Exactly. I knew I've been told in hospital before a visit from my and other doctors, consultants and they told me exactly how I should live and, to pretty much to become familiar with your body and to know the symptoms. I knew what I can or cannot, if I do this, or I do that, and I can expect something else. So pretty much I didn't need any physical or this mobility therapy.

Researcher
Okay. And did anyone, the therapists or professionals or at the time of decision… You said they taught you about your condition? And how to manage that and how to get familiar with a new life after stroke. But did anyone talk about how to self-manage your condition at home? Like self-management? Did anyone talk about that?

Participant
Yes. And that was my consultants, nurses and also the people came next day, the therapy people. They like pretty much put me on legs, on two legs, from other therapists, physiotherapists, physiotherapy. Two girls they told me how to manage myself, they pretty much train me quite well, I think. And also, they contacted me with different stroke, which you are aware of. And with Headway Brain Injury Association, plus, I got a lot of information taken with me. And obviously, the most crucial thing was neuro fatigue, because this is an invisible side effect.

Researcher
How was the fatigue after stroke? Was it a lot?

Participant
You can't ask how was because it stays with you for life after brain injury. It's not what it is.

Researcher
Yeah, no. I mean, was it a challenging thing? Or was it something manageable? Because I know it

will affect probably everyone after stroke but with different degrees, was yours like a highly affecting fatigue, or you feel it was okay?

Participant
It was very highly affecting me. Still it is. It’s not that intense like it was the first few months. And that was also on this group therapy, we've been told how to manage your fatigue. But fatigue is something, well, when I left hospital I thought, because they talk about it, talking through the fatigue, and taking me aware about this and I got a lot of information taking about fatigue. I saw something, you know, like you've got snotty nose, and that will all cough that will when your call is finished. Your not well is also finished. It's not like that. So I was naively thinking, yes, that will stay on for few weeks, this fatigue. And, I was quite glad when I was…

Researcher
Yeah, what was it to the extent that might prevent you from going outside or maybe participate in some…

Participant
Yes, oh yes, absolutely. Because when fatigue started kicking in, and that can be any time of the day, my husband always said: Oh, you're gone now, you need to lie down because he said you’re watching but you do not see, you’re listening but you don't hear. When you have fatigue, the brain suddenly get foggy, it was always a cloud over your brain. And it's like disconnecting electricity. Everything is like cut off and you're not functioning.

Researcher
You just need to rest and stop anything?

Participant
I usually was going to bed and sleep for few hours, literally deep sleeping with very vivid dreams because I don't know if you're aware, probably you are, post-stroke people shared there are two kinds of people. Insomnias, which don't sleep or people who can sleep like me because I never slept better before stroke like I sleep after stroke. But I have extremely vivid dreams. And that's what people with brain injury experience. So I can sleep day and the whole day when my fatigue start kicking, you wake up, and you're ready to go back to bed again. It’s not that intense now. However, I felt also I was that naive and was thinking the fatigue will be worst when you're physically tired and you're over-did something. No, I can do nothing and I still can get fatigue. So it's not a rule. It's unexpected. You only know it will come but you don't know when, and whatever you do, or you whatever you're not doing. It will…

Researcher
Well Participant, we are interested to know also what you have received when you were visiting their rehabilitation center. As a rehabilitation, you said mobility therapy wasn't one of the maybe elements. But what did you receive in terms of rehabilitation therapy? Like, what are the other thing that you received?

Participant
I had neuro-psychology, psychologist. She was working with me, which was also helpful because maybe she was saying the things I know already, but sometimes you need to hear things. And you need to hear loudly. And when people telling you and you realised. Oh, yes. So maybe you aware,

but you have to hear this things so that was very helpful as well.

Researcher
And what else? Especially with the group therapy, like what sort of therapy you had. Participant
So obviously, the most important was managing fatigue. The one was or two, we had two, they invited a person helping us with… I've gotten

Researcher
Maybe with safety at home walking.

Participant
Yes, it was one of the sessions was like that, planning and organizing your day because obviously, you can have memory, short memory problem after brain injury, and how to organise, how to manage your day daily activities. Also, how to deal with the calm situation, with relationships, with the children, with relation, relationship, partner, husbands, wives, etc. Also relaxation techniques, so, breathing techniques. And meditation, had some introduction to meditation. If people can't sleep at night, what kind of even like on YouTube what to play or not to play. What kind of mindfulness music or stuff we've been told.

Researcher
Was there any exercises? Maybe it's exercise, the first few.

Participant
They have but I wasn't participating because that wasn't for me so they providing…

Researcher
Even at home, they didn't ask you to do any exercises at home?

Participant
No because I didn't need, because the exercises or the the activities they providing were for people with physical disabilities, kind of like for example, knitting, so you have to force or using both hands or making something with hammer.

Researcher
Yeah, so this is more of, we call it occupational therapy than maybe physiotherapy. So how to do things with your maybe hand or maybe that's …

Participant
Yes so that’s what this therapy was providing. I didn't need that, for me was more about managing fatigue post-stroke and about relaxation technique, which also leads to the fatigue.

Researcher
Well, you talked about self management, and how they maybe showed you a therapist how to work safely at home or how to use maybe something at home. Did that include also maybe setting some goals, personal goals for your maybe rehabilitation, or to do maybe some therapies for that goal, or that wasn't one of the elements that, you know…

Participant
It was element because when we started this therapy, I think on second session, they asked us about setting goals, what do you want to achieve and what you expect from this therapy, and what is your goal.

Researcher
And then did we fulfill also setting a plan to achieve that goal?

Participant
Yes. Yes. So my goal pretty much was come back on full time. Work. That was my main goal because I like what I'm doing and job is quite important for me. And that was always my main goal for me to be able to self-manage, fatigue, self-manage and organise myself. And also, because I knew that will help me to come back to normal life because obviously at home, I had great support from my husband and daughter so that was easier to achieve at home. But when you have to come back to work, you have to be fully fit and reliable.

Researcher
Was there any impact of the pandemic time? Or let's say pandemic era, on your rehabilitation? Like program or setting your goals? Or, like, was there anything that you had to consider because oh it is time we have pandemic now?

Participant
No, I don't think pandemic in this area affected me or my rehabilitation.

Researcher
Okay. In general, if when you're attending therapy session, when you go to a rehabilitation center, was there any problem because of the COVID, or everything was like normal?

Participant
No we have to be only aware, we're doing this in pandemic time. So obviously, PPE and the stance, that was the only thing, which was a sign of the pandemic.

Researcher
Yeah, if you go back during your in-patient, you said you admitted for one week in the hospital. During that time, also tell us if there was any impact of COVID on your invasion time?

Participant
Well, obviously, it was because even the simple, well, it's not simple, but thing like visiting, family visiting. I was lucky and I was only one week in hospital, but I was in a room with a few other people. And they were weeks in hospital, and obviously there is no family visit. And in any rehabilitation, in any situation, medical situation, when you can have visits from family, friends, close people, you getting better, quicker, your mental health is or well being is better so obviously, that affects people and it affected me as well. Even I was only a week in hospital. But I remember when I had one day really, really bad. And I was all day crying in my bed and the nurse was sitting with me and try to talk to me and try, you know, hold my hands because the only contact with my family was through the phone I even couldn't message properly because I couldn't see properly so I couldn't see what they're texting me. I couldn't text back. So obviously that was, yeah.

Researcher
Was there any other problem because of COVID.

Participant
No, not from my side. I didn't, if I was for example, if I was referred to any specialists during this time, like eye doctor, I have to go to optician and eye doctors, I had two and half hours of consultation with them. It was straightaway, I didn't have problem to get this visit or to get appointment, nothing like that. With the MRI scan when doctor said yes, he doesn't believe that was a blood clot. So he said tomorrow, please we proceed with MRI scan. That morning, the nurse came and said, Participant I'm sorry. There's a huge backlog, 90 people waiting because of this, they have outpatients, inpatients, so 90 people ahead of you. And I was thinking, wow it’s impossible. I was waiting only half an hour, only half an hour. So still, I am aware there is a backlog and I'm not silly saying, oh, nothing is happening, everything is perfect, but didn't affect me directly.

Researcher
Okay, good. How about other people who were around you maybe in the ward where you were in-patient or something, did you know or notice that maybe they had some challenges? Or maybe they face some problems? Because of COVID other than visiting of family members or friends?

Participant
I'm not aware of that because I was younger in the ward and the ladies were quite in mature age, so they didn't speak much to me. I think they were lonely. Everyone was lonely because of the visit but every single day, I saw a therapist coming and taking one of the ladies for activities or exercises, because I heard what they were talking about. And also they were given some equipment exercise when they in bed, lying in bed, like, you know, like some balls to exercise or something lifting. I can't remember exactly. But yes, they were also given after this session, something to exercise. So I never heard any complaints from anyone. Or I didn't receive, I was waiting. I don't know.

Researcher
Yeah. So let us go back about yourself. I know maybe the time is running late so we need to stop this, we don't have much time because we still need to do some assessments. But let us think about your situation, did you have any needs or something at that time since you had your stroke, when you were in-patient or out-patient, did you have any needs that you felt, oh, this need was unmet, or maybe unfit for you? Like something you hope you get care of at that time.

Participant
The only issue is, that is not only me but this is national situation is I don't have access to GP. So after stroke immediately, like with medicine, I'm on a regular medicine, and they said the prescription, that repeat prescription will be with via your GP so you have to contact GP. I was given some tablets when I was discharged. And then you calling obviously GP, and there was quite an unpleasant lady on either side from reception, like pretty much what you want, and why you disturbing us, pretty much like that. So that was the only issue and I said, Yes, I need to please speak to doctor. It’s not available, which I didn't expect different answer. But I said, Oh, I tried to explain my situation, and that I'm on medicine. So go and register, now go to this NHS app, and go through the process, and then you will find out the outcome. So I went through the process, and you have to answer the question or tick the boxes. And every time I tick the box correctly, they

said that was like red, red, warning, immediately call 999. Because I think the system recognised stroke. The system is designed, obviously you ticked the boxes, and you jump through the pages, stuff and then the outcome is called immediately 999. I wasn't in emergency so I was so fed up, then I started lying to the system. I said, if you can't recognise what I need, I will cheat you. But I couldn't take the system either. Because every time I had to call, immediately invert, immediately call 999. So I came back again to after listening to the music for thousand minutes, playing back to GP or to receptionist. Fortunately, it was the same person and she said again, the same thing like, probably she says 2000 other…

Researcher
Sorry, I know. I mean, I mean, yeah, there are some GPs who are like this, and I have personal experience with the same actually, thing. So hopefully, they will enhance their way of delivering surfaces for people. Thank you very much, Participant. One more last question, hopefully, is about what do you hope to see in future in terms of caring for people after their stroke? Like, once you get the news about your stroke, since now, what do you think NHS and other services can do to improve the way they take care of people after stroke?

Participant
I would wish everyone got immediately the treatment I got and immediately all the services, because I said I was I think lucky, or I don't know what's happened, but I cannot complain. But because I am quite active on different stroke websites. I heard people this is national website, it’s not only Southampton where I'm based, it is National, and people talking and people waiting for this for that, and they waiting for months, they waiting a year. And I feel absolutely sorry because I know…

Researcher
Do you think this is maybe because of COVID. Or this is a situation, regardless of pandemic?

Participant
I think it is regardless of pandemic. I know, we get used to blame pandemic for everything but this is wrong. And I know it’s incorrect. We just, this is a good excuse sometimes because I'm working also in public services, I'm working for police and I heard every time because it's pandemic, and I know this is not, because NHS or police or any other services, public services, we’re not doing a job we're doing a duty. And this is regardless… when I hear let's save NHS because of pandemic and let's stop services all treatments for people, it is wrong. Because it's the same like telling army today if we have a war, and we will keep saying please save army and hide army somewhere, like we do and quit other services is wrong. Because NHS, regardless, I know we didn't expect the situation but NHS doctors and other staff, they all trained for any situation. They are not trained only for wiping our noses in this pandemic. They should also very often sent to Africa, let's say where they have completely different situation, and this is also different situation, and I know is unusual situation. But using COVID for everything is wrong. Absolutely wrong. And I think we are using this as an excuse. I was lucky. So I think it's not people don't get treatment because of pandemic, because as I said, I'm from Southampton and I got excellent service. Southampton isn't excluded from pandemic, we also in pandemic, but I think it’s… I don't know, it depends on the ward. I can't say. But when I say when people say, Oh where did you get this service from? How did you get this because we're waiting we delayed because of this and that? Where did you get this from? Is that private? I said no, everything was from NHS, I didn't pay any penny. General Hospital in XXXX, so I don't know, NHS is NHS, but whether this is the same in across the country, I don’t

know

Researcher
Yeah, yeah. Well, thank you very much, Participant for your input on this. So we will stop now recording because we have done the interview part.

(Transcribed by XXXX)

AS: So now we are going to start recording. If you can state your name, and that you agree for us to record this interview so we can proceed please.

E: So my name is Participant and I'm happy for it to be recorded.

AS: Alright thank you very much. Participant, thank you once again for your participation in our study. As you know from the information sheet, our goal is just to actually see your perspective and your experience with stroke rehabilitation during the pandemic time. There is no wrong or right answer. You have the right to withdraw or to stop the interview at any time. if you wish to take a break just let you know, we are happy to do that. We won't take that much of your time, I know it might be a little bit late. But will do a very quick interview. Yeah, do you have any questions before we start?

E: No I don't I think so.

AS: Alright. So if you can tell us please about the context where you are working and how that was affected by the first wave of COVID, how did that affect your daily practice for a stroke rehabilitation.

E: Yeah of course. So back in the first wave, I was on the inpatient stroke rehabilitation ward. And then since April 2021, I've changed to the early supported discharge stroke team, which is obviously community base. So, the first wave, I'm assuming you thinking kind of April 2020?

AS: Yeah.

E: Yes, so at that point I was on the stroke ward. Obviously, I guess from what your studies about, it did have a huge impact on the stroke ward. So the ward I worked in is a 20 bedded unit, and we take patients for rehab from mainly from Warwick, but also from other places in Coventry or Worchester if they repatriated in those kind of things. As I said, it was twenty bedded, but times during COVID there was possibly one stroke patient on the whole ward. So we did notice a huge shift from seeing stroke patients to seeing much more kind of medical, elderly, frailty fallers that kind of thing, rather than actual strokes.

AS: And what was the impact on the system, on the whole system, like do they discharge people earlier, what is the situation for people after stroke?

E: Yeah of course. So I guess you are talking about the patients who actually did present onto the ward with us?

AS: Yes.

E: Yeah, I mean, firstly, I guess the patients who were with us, the first thing I was going to say is they were obviously they were really quite frightened. So a lot of them although usually they would come to us being really quite keen for rehab, a lot of them actually were just wanting to get home. That's probably because of a few reasons obviously, the media hyped up understandably COVID so much that I think they were just so terrified being in a hospital setting. So you can tell a lot them were just so worried about catching COVID, that they wanted to go home and not have the rehab. And then the other side of it I guess, obviously relatives are such a big part, usually coming to visit them and lift their spirits. And I think not being able to have any relatives come made such a difference as well. So from the other side of it, yeah, I did find a lot of the patients would wanting to be home a lot sooner than we would usually do. The other thing I would say is, obviously we are a rehab ward and they come to us for rehab, but because obviously with the pressures of COVID, there was such a push from kind of the discharge coordinators in the hospital and people in charge of patient flow. We were having really regular meetings once or twice a week. And there was constant questions of “why they were here?”, “why they were here?”. The push was definitely on discharge, get them home, get them safe, rather than any real kind of rehab. Obviously, that is the case in a lot of hospitals anyway, but it was definitely a bigger push for that point on, just discharges. And obviously, that meant for even the stroke patients who were being discharged, a lot of our time was then spent on discharging other patients. So, whether that be, you know, the elderly fallers or the medical patients, which meant that actually we didn't really have time to rehab them if they did stay on the ward anyway.

AS: Yeah and do you think there was enough preparation for patients before they discharged from hospital?

E: Definitely not, no. I mean, I think there's different aspects to that, but like I say, a lot of the patients themselves were wanting to go, so they're kind of self-limiting their rehabilitation. But the ones who did stay, I think we all felt a bit bad really, because you want to encourage them to stay to get their rehab if they had potential to improve, but like I said, because the pressure was on us really, to get people out of hospital, it meant that actually our time was really on discharge planning. And actually in that period, a lot of time was spent on the phone to relatives because obviously they couldn’t be on the ward and hear about their loved ones. Obviously, the ward was full and there was twenty patients, we have most relatives ringing everyday and if you're constantly trying to give handovers by every patient, you're not actually able to see the patient themselves for their rehab. So it was a tricky one really, cause you wanted to keep them to rehab them, but then you felt like if they stayed, there weren't getting a lot of rehab. But as I said, the team that I am a part of now is the early supported discharge team for stroke, but during COVID that team wasn't running, it's actually only funded as a new team in south Warwickshire. So it kind of meant if we were discharging them, they weren't really getting a lot of physio at homes. We had a community stroke team, but obviously it was a very small team, and the intensity of that was quite minimal, so it’s once every one or two weeks really. So, I mean there wasn't really a great outcome for any of them, because either they stayed and didn't get loads of rehab or they went home and rehab was significantly less.

AS: Well, let us go back before they discharge from your care unit. While they are at your unit ,what do you think their priorities were for rehabilitation, and what seemed to be there, like critical needs for them at that time when they were an inpatient after their stroke?

E: I guess is different. Some of them were there purely for rehab, or they've been sent from the acute for rehab because, I don't know, let's say their independent before and they just had a stroke and now they were assistance of two. I would probably say those were the easiest ones to keep hold of them for rehab, because the community services and the care services were much better at picking people up who are singles. So we kind of had room to say, “actually we need to keep this patient here to rehab them to get them down to assistance of one”. But the ones who were assistance with one was slightly higher level. When they did present into hospital they were harder to keep really, because obviously they wanted to be home and the hospital managers were pushing for them to be out, because their needs could be met at home if their assistance of 1. I guess the other thing is, we're not used to a particularly medical ward. We’ve got one doctor who covers the entire 20 beds. Obviously if they were poorly they would go over to Warwick, which is the acute stroke ward. Rehab ward, yeah there was one doctor so. In COVID it was really bad and the patients were really poorly, obviously that really wasn't great, because we were trying to look after really poorly medical patients who were on IV fluids, IV antibiotics. And actually, it sounds really silly, but our nurses weren’t really, not trained, that sounds awful, but they had no experience in doing that similar if they had been on the rehab ward for 10-20 years and they haven't really done that. They usually would just go straight back to Warwrick, but it was full.

AS: Wow. Participant, if you think of any other problems from patient perspectives, what would you say their problems at that time, other than what you have said?

E: What whilst they were on the ward with

us? AS: Yeah.

E: I guess for them, I would say one of the biggest things was probably like not knowing. A lot of it was quite, obviously in the media it was everywhere, but when they're on the ward it was quite secretive, and lot of them were kind of, I think worrying that each other had COVID or worrying that it was around. And obviously, as soon as it was on the ward it travels quite fast. So I think there was a huge element of fear really, you know, “am I next?”. It sounds awful, and obviously everyone was fantastic with PPE, but it's such a hard thing to control once it was on the ward, it just swept through and I think a lot of people were quite fearful of that, and obviously they have heard about it in news, and I think fear was a massive thing. I think kind of loneliness, because they couldn't really communicate with the family. Obviously with stroke patients, it sounds awful but, they are quite old and tends to be typical populations quite elderly, so they're not the kind of people who just whip out their phones or go on Skype and that kind of thing to contact family. So, I think a lot of them felt quite isolated and little things that would usually help with this.

AS: Just to let you know, this was reported quite often by our participants, because we are also looking for the perspectives and experiences of patients as well with their rehabilitation and with their stroke during the pandemic. And this was one of their big issues at that time, not having a proper communication with their families and friends, and kind of feeling isolated at that time. Participant, if you think about patients also, because these questions we are also talking from about patients’ perspective. Do you think there were aware about uncertainty about their like rehabilitation programmes, or when they will be discharged, did they get enough care before they discharge or not, and if anyone will follow up with them when they move to live in community or at their homes?

E: Yeah, the only other thing, sorry that I was going to say about the previous point where you say how did they feel and the loneliness side of it is obviously when they're on the ward, usually we would run lots of groups, and we have like lunchtime meals when they all sit in the dining room together and chat, and physio groups, and OT groups, and breakfast groups, but none of that could run either. So they weren't even talking to each other on the ward really. But yeah, back to what you just said, I think probably from my point of view it felt very rushed and a bit kind of panic to trying to get people out. It felt like everything changed week by week, or even day by day. So for instance, the first week I think I moved into the rehab ward on the Friday where the managers rang and they said "you need to clear the ward today, Friday, you know this is a Friday, no one is working on the weekend you need to clear the ward because it is turning into a COVID ward on Monday". And then that actually didn't happen, but we then had 3/4 of a day to clear the whole ward. From my perspective, it felt very rushed. I think probably from the patients perspective, they felt probably quite confused, because everything just happened so quickly. I mean usually discharge planning on the ward, would have discharge planning meetings, we speak to relatives and get lots of kind of warning about things happening. Whereas with COVID, I think things happened quite quickly and particularly, when actual COVID cases came up on the ward, it was such a case of just "get the ones out now, you haven't got COVID get them home". So they probably from their perspective it was very rushed, a bit panicked, not very much warning. With regards to care, I think probably they didn't get, as I've said, a great deal of explanation about what was going to happen at home. But the one good thing I would say about COVID, obviously it is not a good thing at all, but the only positive to come from it was, I don't know if there was more funding or what in social care, but suddenly the social workers could really quickly get placements, or really quickly could get packages of care like that same afternoon, which is something. Often on the stroke ward we would have patients waiting for like a week for a package of care and they are just sat on the ward. I would say that was something that way better, but again probably came as a big shock to the patients when we discussed in the morning this is the plan, and by the afternoon it's sorted and they are going.

AS: Well, Participant, let us move to another aspect. We want you to tell us please about the time when you prepare them to discharge, or as you said, you were told to discharge maybe them over the weekend or over two or three days. So what was your priority at that time preparing them for discharge?

E: I mean that was really hard. I think it completely depends, like the time where they give you like a day to discharge an entire ward, obviously the priorities are completely different, it's literally just trying to get them home as safely as possible. But, you know, a lot of that time we were discharging them, knowing that they're not going to get any physio for weeks and weeks, but that was the only real choice. So I think it was obviously completely dependent on that time, depending on what was on offer. But I would say probably it was all fairly rushed and the priorities weren't fantastic, probably usually patients and relatives would get much more of a say with regards to their discharge. I think at times we had relatives on the phones saying "you can't send them home". But you know when the ward has been cleared or there is COVID on the ward, and they were at a level where they were safe enough to go home then there wasn't really much choice. Obviously, it wasn't to say if it was much different. But at one point when it was really really bad and it was the first wave, when in order to go to a care home they had to have a PCR test to make sure they were negative, before accepting. I remembered one of my first memories was, it sounds awful but I shouldn't repeat this, but they

swabbed the ward of the patients who were going to care homes that had their swabs. But the care homes were just saying “ have they had a swab?”, and they'd say “yes they have”, and then they send them home, but they wouldn't wait till the results. So just pushing them out before we did even got the results of the swabs. And luckily, like touchwood, it was okay, but I mean they could have been at that home for two days and then got their results and say “oh actually, you know that patients was sent to you two days ago is positive”. It felt very rushed and very manic.

AS: Well, hopefully we won’t have to face this experience again. Participant, just after they discharge, you didn't have any chance to contact them, or maybe to follow up with them, or to deliver any sort of rehabilitation to them right?

E: So it depends. At that point there wasn't an early supported discharge team, which obviously at the moment that's now a place, that we would pick them up within 24 hours off in the same day, and they can get daily rehab at home. Whereas that wasn’t running, but there was a community stroke team, but that consisted for the whole of south Warwickshire with one part time physio and two assistance from physio. So some of them, if it is appropriate did get community stroke input. But it was very sparse and it wasn't a lot of input. And also no care homes were accepting the community stroke team into it, so it was only for people who went home. So some people might have been appropriate for ongoing physio, but they weren't allowed it because they were in a care home. And actually, to be honest, a lot of people we found, once they got home, they didn't want the community stroke team coming in, because they were so worried that, you know, “their base was at the hospital and that they're going to bring COVID into my house and spread it to my family”. The team weren’t actually that busy at times because nobody wanted to see them.

AS: It was difficult. So Participant, if you think of any strategies that were used, maybe at your place, or maybe at another workplace, just to maybe manage the way of service delivery for stroke patient, maybe to provide them with any sort of maybe resources that will help them work at home, or maybe to prescribe a home programme for them. Have you had any experience with this from your perspective or from other maybe colleagues' perspectives?

E: Yeah, I mean if I’m being honest, obviously usually, the usual thing we would do in the ward is, we would take the time to give our patients a really specific home exercise programme if we knew they needed ongoing input, and they were waiting for the community stroke team. But if I'm being to be completely honest, during COVID, we didn't have time to be going around and giving everybody specific home exercise programmes. As bad as that was, knowing that they're likely to be waiting weeks and weeks for their rehab, a lot of them have either got nothing probably, or a very generic, like seated home exercise programme that's kind of one size fits all, rather than anything particularly specific to them. And I guess the other thing is that a lot of them who were in that point weren’t very well, because for a fair bit of COVID a lot of the stroke patients had COVID themselves. So they obviously had such huge fatigue because of the stroke and the COVID that a lot of them weren’t really capable of doing much anyway.

AS: Do you think this is still the same case till now, or can you tell us how that the thing changes overtime?

E: Yeah I think some elements, as in the first wave compared to what the stroke ward is like now?

AS: Yeah.

E: Yeah. I mean obviously as I've said I'm not actually on the stroke ward now, but my base is in the same office, so I've got a fairly good idea because we get a lot of the patients coming home to us. I would say, so they have continued, they used to call them "stranded patient meetings", it was like a big meeting every week over Microsoft Teams where the managers of discharges and patient flows would basically grill you about every patient, say "why they are here, why can't they go home, why can't their needs be met at home". And that has actually continued since COVID, although it's no way near as significant and kind of manic, there is definitely that push for us to follow through of "can we get them home". Particularly now we have got the early supported discharge team. Unfortunately, I don't think the benefits of social care carried over quite so well, so again I don't know if the funding has changed or staffing has changed or what. But yeah they're definitely not picking up patients as quickly with regards to packages of care and discharges to care homes and things at the moment. I'm trying to think what else you have asked, sorry could you say the question again?

AS: I'm just wondering if the case has been changed since COVID, and since, you know, the life is coming back to normal hopefully, and we won't see any other pandemic waves. So I was wondering if the case has been changed in regards of just discharging people immediately after their strokes, and they don't receive any sort of maybe rehabilitation or preparations for discharge.

E: Yeah no, it's definitely back to a more rehab focus. Although, as I say, the push to get them out is there, it's definitely not as significant as it was before, and they definitely able to keep them longer to actually give them proper rehab in the hospital setting, rather than just pushing them out when they are still quite low level and they've got goals. So it is definitely more rehab focus, and it's definitely more of a stroke ward now as I felt like for like a good 18 months where it was a medical ward.

AS: Are you still working with them at the same unit?

E: I'm in Leamington, but I changed in April to the early supported discharge team for stroke patients. So basically the initiative is when they're on the ward they have their stroke they would go for rehab, but if they need somebody met at home we would provide like a high intensity therapy at home every day. But that wasn't running during COVID, it is a new service.

AS: So now are you visiting patients at their homes now?

E: Yes.

AS: And how is that going?

E: Yeah no I like it. I actually feel like I'm back to being a stroke physio again, rather than an elderly or medical physio.

AS: If you could just tell us a little bit about your maybe experience now with your patient? Are you delivering some sort of maybe self-management strategies to keep them maybe engaged and involved in their therapeutic plans?

E: Yeah, I mean obviously self-management is really important, but I probably would say at the moment, but whatever means sometimes if it's needed, we're seeing patients everyday at home, so they are getting the same intensity of rehab in their own homes, rather than being in hospital, which obviously is really good for them. I definitely I think there was a shift back to being more engaged in the rehab and really having families being quite keen for rehab, as opposed to before, just not wanting anyone in the house, not wanting to actually engage with it, just wanting it to be over. Whereas, definitely people are wanting to do that a little bit more now, which is nice, so I actually feel like we are having proper physio sessions and doing a lot more. We're not really back to doing groups, obviously that's bigger on a ward than it is in the community, but that is still something that's not quite what it was before. And you still get patients who obviously are a bit more nervous, because you go in with full PPE and visors. I think some people don’t really like that in their own homes, or worrying what the neighbours think and that kind of thing. We still get that as a bit of a problem, but it definitely definitely doesn't feel quite like it did you know a year ago.

AS: Yeah, well if you want to go back to think about the situation during that lockdown and the pandemic time, I just want you to think as a therapist about the long-term impact of not getting a proper rehabilitation after stroke? Especially on mobility because we have some focus on mobility in my study. If you just go back and tell me a little bit of what do you think the impact for long term scale for those who didn't receive proper rehabilitation?

E: Yeah I mean I think it's massive. This is definitely a really big problem. I mean, when I was actually working on the stroke ward, I mean we had one young man who is in his 40s, and he was discharged as a hoist even though he was working and independently mobile before, and obviously we sent him home knowing he was going to get barely any rehab. So I don't know what he's doing now, but the likelihood of progressing from that with very little rehab is obviously quite poor. So I definitely think it had huge long-term implications. And I think the other thing which is very specific to where I work in South Warwickshire is, at the moment as I say I'm working in the early supported discharge team, but that's only been running since April 2021, so we're only funded the entire community stroke to accept people who were referred to us following that dates, so if their strokes been since then. So any of the people whose stroke was before April 2021, they are kind of falling through the net if they need re-referral, because they haven't had another stroke. So really they were the people who we were hugely missing out because they didn't have the rehab at the time. And if they were to go to the GP now and say “you know I'm really struggling with my mobility”, but they haven't had another stroke, then they wouldn't come under any services in South Warwickshire.

AS: Was there any plan from what you have seen for these people who had their stroke before that date? Before April 2021? Was there any plan for them to recover maybe there were cases?

E: I mean, no, and the other thing I would say, it's not obviously but it sounds like it's a huge population of people, but actually, we are talking really small numbers that actually presented onto the ward who have suffered with a stroke during COVID. It sounds terrible, but in January we’ve got COVID really bad on the ward and I think a third of the stroke patients died, some of them died at that point, and then there's others and we think “you’ve had a stroke and the prognosis after years are not fantastic anyway” in a lot of the elderly people. So actually I think it's probably quite a small population of people who still need that rehab and aren’t benefiting. At the moment, luckily we haven't had any

come through who we had to turn down, but obviously it is quite a real possibility, bearing in mind they didn't get the rehab back then, so I don’t know if they are having to go to private physio or a what instead, but yeah it's not great.

AS: Alright. Participant, let us go to the last part of this interview. We want to talk about, hopefully we won't see any other wave of pandemic or any other pandemic in the future, but let us just think in case if we have any of these waves coming back, so what would be your priorities for rehabilitation after stroke as a therapist?

E: I mean, this is kind of hard because obviously of the new service is in place, but I think we will be much better equipped in South Warwickshire to deal with it if it hit again, because we do now have the early supported discharge team for strokes. So it does mean that, I mean we're quite a big team, and therefore if patients come into hospital and they are having a stroke and they go home a day or two later because they're pushing them out of the hospital, then actually we are now much better equipped to actually go and see them and provide their rehab at home because we have a team that's purposely set up for that. So I actually think it would, touchwood, be much less of a problem now. But unfortunately the fact that the services running isn't actually anything to do with COVID, it is just to do it with government funding that has been in the pipeline for quite a few years. But I think because of that, it definitely would be a lot better, but then I guess on the other side of things, you still have the problem of if Covid is back, then a lot of those patients aren't going to want people coming to see them in the community again. So that problems is still there. But I think it's just thinking outside the box and seeing if we can do more over like Microsoft Teams, and things like that. But I know, from speaking to my colleagues when I was on the ward then, and some of them worked in community it was really difficult. Things like, I don't know, somebody's got impaired balance, and you're trying to challenge their balance over Microsoft Teams, its obviously not really safe is it, so yeah.

AS: If you would speak to maybe healthcare provider or maybe policymakers, what would you say or suggest to them in this case, as some somebody who have experience the whole story of COVID and how to deal with patients at that time? In regards to the healthcare system as well, what would you suggest to have during the pandemic?

E: Definitely more funding and more support within social services, because that gets them out of hospital when there is COVID in the hospital, we can obviously get them to a safe place. I would say, probably from a physio point of view, and I think a lot of my colleagues would say this as well but we just became a generic therapists, so we weren't physios or OTs or speech therapist, you’re just a generic therapist. So when the nurses were really short because half of them had COVID, you ended up doing your therapy quite functionally. So you'd be helping to feed them at lunchtime or walking them to the toilet, or helping them wash, things that as a physio I wouldn't usually do, but you would just try to make the most and try and get kind of bits of physio into more functional tasks whilst you’re helping out others on the wards. I guess just being positive and trying to get the most out of every session, even if it's not like a typical session that you would do usually with your patients.

AS: if you think you have somebody who has to isolate because of maybe testing positive or for any other cases, how would you see the plan for these people in future, how it should be?

E: As in isolating on the ward?

AS: Either in the ward or maybe they have to isolate at home after they discharge.

E: Yeah I mean that was something that was really tricky on the ward when I was like in there, because initially the plan was, infection control said that you try and isolate them in the side rooms, but on the ward of 20 beds we only had four side rooms. So when COVID kind of hit, those four patients will go in the side rooms, and then obviously, then and there it'll overrun and then it started getting into the base. But when it was just four of them in the side rooms, the recommendation and the advice from infection control was that you had to see them at the end of the day. I mean I'm not entirely sure why, because obviously you are wearing full PPE, but just to minimise any risk. So I do think sometimes their rehab was quite limited, because obviously you had to make sure you did everything else, and all the discharges and everything else that needed prioritising. And then if you had time, you would see them at the end of the day, knowing that was kind of the last thing that you could do, because you couldn't then go back on into the other bays. So I think they probably did miss out in the rehab. If they were isolating at home, I don't know on this point, I don't necessarily know what would have happened. At the moment what we are doing is, if anybody in the community has been in contact with someone with COVID or has COVID, we just do a risk assessment. So if they urgently need therapy then they'll still get that therapy, but we will try and see them as last person of the day. But we are kind of tending to find that actually, we are leaving a lot of them for that 10-14 day isolation period, and then picking them back up again afterwards. So during that time, we were just ringing them and going through exercises over the phone, and saying "look, we will come back and see you in 10 days". But obviously, if they're dangerous, or its unsafe, or they really need their rehab, then it's different. But so far we have just tended to not do a lot with them. And also, obviously a lot of them are poorly if they had got COVID, so they were not really appropriate for the rehab during that time anyway.

AS: Yeah, well thank you very much Participant for your time. Just give you the mic now if you want to add anything or you feel that we have missed anything about your experience with stroke rehabilitation during the pandemic, please feel free to talk if you feel that we have missed anything, or you want to add any point?

E: Well I don't really think so, I think you have covered most things to be honest. I guess for just a summary, it was just so noticeable how little stroke patients were presenting into hospital. And I think a lot of us at the time, we were trying to work out what it was. I think probably the main reason was obviously because the media had, not over exaggerated to talk, it was awful, but I think they created such a storm around hospitals and that kind of thing, that firstly, people were so terrified to come into hospital because they didn't want to catch COVID. But also like, a member of my colleague who worked in the community at that time had gone to see one of her old stroke patient who she had been seeing for a while. And she re-stroked and completely lost her speech, but she didn't come into the hospital, and her husband didn't bring her into hospital because they said "we know they're just so pushed". And it's like "you had another stroke", like obviously you're still a priority. But I think obviously it wasn't just the fear, it was also the people who were just trying to be nice and think actually "I'm not important", and they weren't really looking after themselves. I don't know, I can't help but feel it's not just like, people being scared because you kind of think if you've had a big stroke, you don't have much choice other than to come into hospital. So I don't know if an element of it would like, with more people working from home that less travelling, less stressed. But maybe like stressed levels are a bit less, and that as a consequence of minimised strokes a little bit, if that was like a tiny

part of it as well. I don’t know, I would say, surely these people aren’t just all sat at home like having their strokes and not coming in. I don't know, if you find out let us know.

AS: Yeah we’ll definitely are interested to see how that goes. Thank you very much participant for your time.

AS: Alright thank you very much for participating in this study. I’m just taking your consent, are you okay with recording this call?

L: Yeah that’s fine.

AS: Alright, the purpose of our recording is just to go back to the information later for analysts, because we are collecting a bunch of this interviews, and we want to go back for analysis, okay? So if you can please start with stating your name and that you are OK for recording, please?

L: Yeap, my name is Participant and I consent to you to record this interview.

AS: Alright thank you very much. Participant, I'm just remind you of what you have read in the information sheet. It is your right to stop at any time or to ask for a break if you want to take a break. And you have the right to stop at any time if you don't want to proceed with the interview. And then just confirm to you, we won't use your name on any of our reports or paper that will come out from this study, it is just for recording your interview, and then later we won't use your name at all, so feel free to share whatever your thoughts or opinions you want to share with us about your experience and also patient needs and problems during the pandemic with stroke rehabilitation, OK?

L: Yeap.

AS: Do you have any question before we start?

L: No, no that's fine.

AS: Alright, thank you again Participant for participating in this study. And if you can please start telling us about stroke care and rehabilitation delivery services since the first wave of the pandemic?

L: OK, from my experience, at the very beginning of the COVID pandemic, the numbers of patients admitted to our acute stroke unit with stroke was significantly reduced. So I think that was because a lot of people are scared to come into hospital and of the fear of the virus. So the numbers were significantly reduced, and also the pressure to get patients out of hospital increased because we needed beds for COVID patients. So, I supposed in the acute setting, the pressures were very much on discharging home rather than rehab, which was quite difficult from like a neuro perspective; you know, its all about rehab with neuroplasticity and practice and repetition, that was all very different at the beginning. I think as well at the beginning when we had to change wards because of COVID, and with new infection control guidelines, we lost our therapy gym and therapy assessment kitchen and any access to stairs to complete assessments, so

we had to adapt quite significantly to be able to assess and treat patients who were still inpatients as well, which was difficult. We still haven't got those spaces back even now, which is a shame, because that still impacts patient rehab and the ability to assess them, especially cognitively impaired patients, if you need to do a kitchen assessment to make sure they are safe at home, not having access to those facilities at the beginning was quite difficult. However, I guess we just had to adapt to what we had and ring them and have them sent up to the ward, or use of bedrails and use of a step within the bed space, but again obviously, not ideal. So, we were able to adapt but from a patient care and rehab point of view, it wasn't ideal for the patients.

AS: Well, could you tell us about some of the modified systems that were in place at your workplace for people with stroke?

L: Yeah, so when we last had access to the gym, one of the things we did do, is we would bring some plinths up to the ward if there was a bay free, so we would do that for sitting balance work. We weren't allowed to use stairs or therapy assessment stairs, so "can we modify that?". We got a step, put the bed up and bed rail up and then use the bed rail as the banister, and did the steps next to the bed for 12-13 times to mimic a flight of stairs. But obviously, that very abstract concept and a lot of patients didn't quite understand that. Um, what else did we do. I think as well a lot of the cognitive assessments, that weren't able to be completed in hospital. If the patient was safe and lived with someone else, we would get them home out of the acute setting, and ask the community team to do those assessments with the patient at home.

AS: Yeah and how was that going at that time?

L: Well, the community services were also quite significantly impacted. Some patients didn't want external therapist, or other members of the public going into their house, because of the fear of COVID. Also, from the Trust's point of view, they were recommending as much that could be done over the phone or virtually as possible to reduce any further risk of infection. However, after the initial first month or so maybe, the community services increased, and people were more willing to go in, there was more availability for people to go into the house, but the patients seems more blame for them to come in as well. But, It wasn't ideal.

AS: So Participant, if you could summarise what was provided by the NHS and other community services for stroke survivors during that time, during the pandemic time? What would you say as services that were provided at that time, during that lockdown time and the first wave of the pandemic?

L: What services were provided during the first wave?

AS: During the lockdown, yeah during the first wave of the pandemic?

L: I think the services were still provided, as in acute and community, however they were adapted to try and be of less patient contact as possible. In terms of increasing virtual and telephone appointments in the community and in the acute setting as well, reducing time in hospital. Obviously, visiting hours of visitors were restricted, so patients weren't able to have family members come in and it also significantly affected I would say the general understanding from the patient's family and what was going on, especially if we wanted to send the patient home, they

weren't really understanding necessarily what was going on. And I think stroke is quite a scary term to be diagnosed with.

AS: We will come to this point actually. So let us move to patient, the impact of the pandemic on patients, on stroke survivors. So what do you think were the problems for people who had a stroke during the pandemic in accessing services?

L: Sorry, could you repeat the question again?

AS: So yeah, what where the problems for people who had stroke during the pandemic to access the services, like the rehabilitation services?

L: So the problem, well for the access of service, in the inpatient stroke unit, the one in our city, they again were under a lot of pressure to get patients out of it as quickly as possible. So they would normally get 4-6 weeks of this acute stroke rehab unit like in a community hospital, however most of them were only spending like a week or so there while they've got a package of care ready and appointment ready, and attending for patient home immediately. So they weren't having time and opportunity for rehabilitation they should have been receiving. So that would have definitely impacted them, especially if we know that acute care and rehab immediately after stroke is what was needed. And then the patients who were more mobile or who had less of their symptoms who were able to go home straight from hospital probably had less access as well to EST and community services because of the virtual and cancellation increase, rather than face to face. So they have definitely sort of missed out, or would have missed out on rehab, compared to someone who maybe had a stroke the same time of year as the year before.

AS: Well, Participant, what do you think the impact of them missing their rehabilitation during that time?

L: Well, I think it is probably impacted them in multiple ways. Firstly, their quality of life, because they might have not got back to hobbies, or gained their independence back as quickly as they would if they had more acute, um, what was the word, um acute regular rehab. And also about, sort of neuro plasticity, its about repetitive task practice and this happening in the early stages.
They may never or they may not reach their full potential because they didn't have the advice and opportunity at the beginning. I think as well like family play a really important role in stroke rehab, especially engaging them and getting them to do activities and repetitive task practice with the patients when therapy isn't around, and not having the family in the hospital, or be able to visit them say at the stroke rehab unit, we cannot get that additional time and those additional repetitions of a certain activity which will also impact their prognosis and their ability to recover in general. I would have thought they are likely to have been more affected or them taking a much longer time to get back to where maybe they should have got quicker if they had more intense rehab in the initial stage.

AS: Participant, if I would ask you, how do you think this might affect mobility and recovery in the long term?

L: I think it definitely affected mobility and taking a much longer time to return to a more mobile and independent way, or if they ever got there, and that obviously affects their quality of life and their day-to-day function. I think with, well, with any neuro rehab, its all about time and repetition

and the patient also having the opportunity to do this and to keep practicing, then the carryover is going to be less, and it will definitely still affected them, sending them back to where they want to be in this amount of time.

AS: Thank you for this. Participant, from a therapist perspective, can you please tell me about the patients' needs during this pandemic, in terms of rehabilitation?

L: Sorry, say that again.

AS: I was asking about patients' needs during the time of pandemic, in terms of rehabilitation?

L: Okay , well I would say the needs during the pandemic would be the same needs as general. One thing is family and emotional support, they didn't have it. And I think that patients do need this after acute stroke. Uhm, so, families provide familiarity and reassurance, and also support, emotional support for the patient. In our Trust, it was resolved by trying to promote, or you know, giving patients more access to telephones and getting an iPad so they can speak to their families through zoom or something. However, it is not really the same. So I think family support was definitely missing and has definitely impacted patients. So even now, we are only allowed one hour a day for visitors and that hour is normally, we dedicated the hour to allow visitors to spend with the patients rather than getting them to watch our assessments or getting involved, because that is not really why they are there, they are there to see their relatives, especially patients who have been in for a long time, so that still definitely is missing. I think as well, communication, from a patient side of things how it has been affected with the mask wearing.

AS: Sorry. What do you mean by communication here, with who?

L: I don't know if I'm slightly going off track. But uhm. I think wearing a mask, in terms of patient recognition of the therapist and building that patient rapport, can sometimes be a bit more difficult, and that is all part of rehabbing in terms of being able to trust someone if you have a familiar face. The mask sometimes affects that recognition and remembering of the therapist. And also, for those who are severe harder hearing, or who rely on lip reading, we had to adapt to speaking more clearly and louder to compensate for that as well. However, I think that has generally been okay. I know some Trust introducing or has access to mask that are clear, so patients could see the therapist's mouth, but that hasn't been rolled out everywhere, so we didn't have access to that.

AS: Yeah. Do you want to add anything about patients' need during that time? Or we can move to therapist part?

L: Yeah I mean I think. Sorry I cant remember what I said there.

AS: Alright, thank you Participant. Thank you very much. Let us move to another part about the strategies for management during that time. What will the strategies that were being used to manage access to the services? Or maybe for you as someone who is working in acute unit. I think that the proper question would be, what were the strategies that have been used to deliver rehabilitation for patients during that time of pandemic?

L: Okay, so strategies we used on the acute stroke unit were adapting our therapies to what we had available on the ward. So, If we wanted to do standing, standing exercises, or sort of start working on gait re-education, having the wheelchair, patient having a wheelchair next to the bed, raising the bed up, tucking in bedrails, lift the bed up and using that as a bar, rather than access to parallel bars and having another therapist on the other side, and then using that as to mimic parallel bars so we could still get them working on stroke gait re-education, so that was the gym setting. I said about steps previously, so mimicking the stairs using a rail of the bed and putting a step next to it and practising that. At times when the wards were less busy, having bringing up a plinth, so we could do sitting balance work on the plinth rather than doing it on the bed. However, that wasn't always possible. The Trust has somehow got new beds as well which had functions on and you can max out the air in the bed to make it a bit firmer, rather than the really unsteady hospital beds, which we've done with sitting balance as well. I guess another strategy on how we manage the lack of visitors was getting an iPad for the ward, and having one of our therapy assistants go round patients and help the patient or set up for the patient so they can speak to their family over there, or just some access to a telephone. Another strategy we used is at times when maybe we were less busy, or have less patients in, some of the healthcare assistants or nurses, who perhaps had a little bit of spare time, was engaging them with the patients rehab. If a patient was given an upper limb rehab, one of the healthcare assistant might have a game of connect four with them, that gives them that little extra bit of repetitive practice and rehab, which they might have been doing with the family if they were allowed in, but the family wasn't allowed in. And that was also an additional element which helped with a bit of social contact and reassurance as well. The therapy assistants also did quite a lot of baths as well.

AS: Was there any example of good practice used during that time, you would consider?

L: Good practice you say, sorry?

AS: Yeah. I said during that time, if any of these strategies you felt was a good practice?

L: I think being a physio you have to be very adaptable to different situations and how to deal with what might be thrown at you. So I think we were able to adapt to what we had. So I wouldn't say it wasn't good practice. I just think that if we had access and availability to use the normal therapy gym and services that we had, but because from the parallel bars and therapy setting of stairs, then it would have been easier in a more meaningful approach sometimes to the patients, so they would be able to understand in a more normal context. But I think that the adaptations we used were sufficient to manage with the resources we already had.

AS: yeah, at least during the pandemic time. Participant, I would ask you was there any efforts to introduce self-management programmes or interventions to the patients for their recovery?

L: Yeah, so we would encourage the patients to do as much as they could independently. We have some gloves, a clutch and boxes which we often give out to patients for upper limb rehab, with different levels and different bits of equipment, and if they were able to manage certain activities and exercises themselves, giving them an exercise sheet with some prompts and reminders of what to do, and encouraging them to do as much as they can themselves. Obviously walking and standing is a lot more difficult, but if someone was using a ReTurn or a Sara steady, we would encourage them, as long as they were cognitively OK and safe to do so, that they could do some

standing practice with that with a couple of the nurses, and adjunct to their therapy sessions. And, yeah.

AS: Were there any specific thing of self-management was used to improve mobility in particular?

L: So obviously depending on how many factors, but if the patient had been assessed and was safe to do something with assistance of one or with the nurses or using a Sara steady for example for standing, then yeah we would encourage them to, you know, go practice walking up and down the ward with one of the nurses or with a therapy assistant, rather than with us as long as the physio has deemed it safe to do that, that has definitely encouraged other additional rehab to the 45 minutes that we would be giving them.

AS: So you would consider that as something that was beneficial maybe for patients?

L: Yeah, I think so. I think it was beneficial say for their walking recovery, but also the fact that they may be felt they had a little bit more independence and a bit more time to get on their feet for practice, even If it was still with assistance or with someone.

AS: Thank you Participant. We will move to the last part of the of the interview. It is about sitting maybe future plans, if we have another wave or another pandemic, and hopefully not, but let us put that in mind. If you can tell me first when the health services start to reopen normally, what do you consider as priorities for stroke rehabilitation, if everything goes back to normal and what would be your priorities for stroke rehabilitation services?

L: I think its difficult. So I work in the acute ward, but I think the priority would definitely be maintaining that access and availability of community stroke rehab unit and early supported discharge team, and their ability to continue to see patients. I think since the initial wave and all the stress of the first bit of COVID where everything was unknown, some of them sort of adapted and develop, but definitely continuing to allow patients for dedicated time and duration that they should have access to these services for support, definitely essential for rehab and recovery after stroke. I think there are ways that things can be adapted, for example, if the patient did have to isolate because they got COVID, we have technology now, and things can be done virtually, or at least things can be discussed with maybe a family member and the patient virtually, and get the family member to do things. And at least therapist on the other end can see through the videos what is being done, and give them specific techniques and advice. So that does give a bit more scope to allow rehab to continue while the patient may be isolated or if they were to be in another pandemic. Obviously it is not the same, but it is better than nothing. It might work really well for some people who are technologically savvy. Obviously, there are people out there who aren’t, so its not really ideal for them. At the moment on our ward we try one of these Gripable bits of technology, and I don’t know if you have heard of it, but its a device looking at upper limb, hand function rehab, and its connected to an iPad, or a version of an iPad. And there are games which the patient can do, so that can almost be linked to a therapist device, so they could see how well they are getting on and how much rehab they're doing with their hand each day, you can set targets, so something like that might also be useful if the therapist wont actually be able to go in.

AS: What is the name of this intervention?

L: It's called the Gripable. It's a new piece of technology and it tracks their progress and how much rehab they have done on it, range of movement and their accuracy. So even if someone couldn't go in, can the patient be provided with this so they would be able to track and see how well the patient was doing with these exercises.

AS: Was this one of the strategies that you have developed during the pandemic time, or this has been developed before?

L: Well, I am not entirely sure when it was made official, but I know it has been developed for a long time. This particular one has been going on for like years.

AS: Maybe now it is the time for using it?

L: Im guessing there is other technology out there which have maybe been developed during or since the pandemic, or the ideas that are at least out there to allow tracking I guess, externally.

AS: Yeah, well this is a good idea.

L: If people have got technology like that, firstly it will help engage the patient, being a bit more fun, and the patient can see progress visually. I guess it is easier to see progress if you are being given numbers and statistics on a screen, rather than just "how many cones you can stack" or something. So I think that definitely, that sort of idea, other ideas of technology to be used in rehab is something that will probably start coming through more now in the future to give patients more independence and to be able to continue this without as much face-to-face contact potentially.

AS: So let us go back to the question that I've asked initially about what can healthcare system do to ensure optimal services if we have a future wave of the pandemic to deliver rehabilitation?

L: Uhm, what can healthcare services

do? AS: Yeap.

L: Yeah, I guess it is ensuring that the appropriate PPE is supplied to stroke rehabilitation services, and to ensure that patient safety, obviously, is the top priority, but that services be continued to run aren't sacrificed because it is essential for these patients to get back their rehab. Um yeah not really sure what else to say for that.

AS: I think we have got a good lesson from the first and second wave that we already had. So I think people now are more prepared if they experience anymore waves or any other pandemics. How can you see the plan for people who need to be socially isolated? Is there anything that you would consider as optimal, maybe practice for them? Like how we can deliver their rehabilitation if they have to isolate?

L: Yeah I suppose what I've said before, virtual would probably be the way forward for those who have to isolate as long as the patient is able to use the technology, setting up video calls, so the therapist can see the patient, and maybe the patient's relatives or if there is someone else there and teach them certain things or give them advice on being able to assess through visual means. Obviously it is not ideal, but it is better than nothing. And also, like I was saying about technology,

I think that will become more prevalent in the future. So you know if someone was doing something and being able to record it through an app or through a computer, then this can be monitored externally, and advice can be given to help progress that. For example, if there was levels on a game, and someone is on level 1, the prompting to move up to the next level can be given by seeing potentially their statistics and the therapist can do that virtually or over the phone, rather than being face-to-face with them. I think I guess as well like guidelines at the moment, say that only the patient with COVID have to isolate, or the person with COVID have to isolate and other family members, I believe, don't have to, as long as they test negative and don't work for the NHS. So potentially, the therapist could visit the patient's family member outside the property, outside of where the patient has COVID and liase with them, as long as the patient consented and they can at least give face-to-face advice and demonstrate techniques, or provide certain things to their relatives who can then maybe go back and give that to the patient when they come out of their isolation or explain that to them which helps in other means. Yeah, that's probably all that I could think of. I mean until you no longer have to isolate, is always going to be an issue, and I guess one pro is that the isolation periods come down from 14 days to 10 days, but, well, I don't know if it is going to become less than that. And 10 days out of stroke rehab in an acute stage is quite a long period of time to go.

AS: Well hopefully services will go back to normal. And the question will be, later in the future, hopefully we won't see that again. Thank you Participant very much for your rich information that you have shared with us. We really appreciate that. I have done with my questions. I'm just giving you a time if you want to add anything or you feel we missed any point about your experience or patient experience with rehabilitation post stroke during the pandemic, so please add anything if you want to.

L: No, I don't think I have anything else to say.

AS: Alright, then thank you very much for your time Participant. We have a gift voucher for you as appreciation for taking part in this study, a 20-pound shopping voucher.

GMT20211108-170524_Recording FEBRUARY 2022 SM - DEPTH - 45 MINS [FEMALE RESPONDENT]
[Other comments: Over speaking obscured some speech.]

Start recording now. This is just for the purposes of going back to the information because we are doing that analysis after we collect all the responses from our participants. Thank you, again, for your time today. If you can just state your name and that you are okay with audio recording of this meeting.

Yes, it's Participantand I'm happy for it to be recorded, yes.

Thank you very much, Participant. I just remind you of the objective of the study. [Moderator outlines market research and reads privacy statement]. Do you have any questions before we start our questions?

That's fine, yes.

Thank you, again, Participantbefore starting. If you can start, please, telling us about a case of stroke care under the rehabilitation services since the first wave of the pandemic.

Okay, well, my role is community outpatients. Usually, the usual care pathway would be that somebody would have a stroke, they would go into hospital. They would, then, come out and have about six weeks with the early supportive discharge team. That's at the point that our team would pick them up after that. In the first wave of the pandemic, so going back to March, last year, in fact, I'd actually had an operation and been off work for six weeks. On the first day that I came back which would have been mid to end March - whenever we went into lockdown - that was the day that we actually closed our services. We basically had a few days to phone everybody we currently had on the caseload to say that we were basically stopping the service. We were redeployed to general wards. For those first - probably going March, April May - probably into about June or July of 2020 there basically was a very minimal service in that we answered the telephone and dealt with a few telephone issues that we could.

That was basically it. At that point, we hadn't got any technology in the department. Although we'd got computers, they didn't have cameras or microphones. We had no smartphones so for those first five months we couldn't even do anything virtually. The only thing we did really was a few telephone calls.

It seems like the services were completely stopped at that time, for maybe four or five months.

Yes.

Was there any modification to the system after that to maybe tailor the situation?

When we first started to see people again, by this point we'd actually been given cameras to attach to the computers. They downloaded Accutreat for us. Probably about four or five months in we started doing

some virtual sessions which are very difficult really when you're working with stroke. From a physical point of view, if you're looking at people that you're wanting to do physical treatment with that's very difficult to actually gauge from a movement and tone point of view over a video camera. That was challenging. Obviously one of the difficulties where we attempted quite a lot of these sessions but because of patients' cognitive difficulties or lack of experience with IT we just actually couldn't get the connections working. There were quite a lot of aborted sessions, as well. I have to say it was all a learning curve for the staff, as well.

It's completely a new experience maybe at that time and told all of a sudden...

Yes, we probably had another couple of months doing just virtual. Then, gradually we did risk assessments. Then, prior to COVID, we used to do a lot of groups. None of those groups has started again yet; we are just continuing to do one-to-one. Initially, we started only doing home visits. I would have said prior to COVID we were probably about 60 per cent home visits and about 40 per cent outpatients. Initially, we went back 100 per cent home visits. Then, we've gradually come back to doing outpatients but only one-to-one. We did that...

Participant, when was that when you returned back to 100 per cent of home visits?

That would probably have been the autumn of last year. We continued to do that until January-ish [sic] this year. Then, the whole team were redeployed again.

Yes, for the second wave.

For the second wave. We then closed down from January to...

March.

...probably about, yes, March/beginning of April. Myself and an assistant went back in April and I went back part-time. My other colleague has been back three weeks, so in the last three weeks, we've only gone back to the staffing levels that we had prior to COVID. Other than the fact that I originally was working full-time and I've now dropped to three days a week.

Yes, well, sounds good. Participant, let us look from another perspective. Let us look from a patient or people who were affected by stroke during that time. As a therapist, what would you think were their problems and challenges facing their stroke during the pandemic times?

I would say that the big issue that comes out time and time again from the people that we had at that existing time on our caseload is mental health issues, undoubtedly; massive impact. We used to do a lot of groups and activities and that supported people's mental health in coming to terms with having a stroke. Then, suddenly, all of that was gone. Whereas before, they would have had a number of activities and a bit of a programme that they had during the week, they went from that to nothing. That has had a catastrophic effect on people's mental health. Since we've started seeing people again, the majority of people that we had on our books, at the initial time, have gone backwards. They stopped doing their exercises. They not only from a mental health point of view deteriorated, but they deteriorated from a physical point of view quite significantly. It's affected relationships within the family because they've got nothing to get up for. Therefore, they couldn't be bothered to get out of bed often.

We have seen, from a mental health point of view, some massive deterioration over the COVID period. I think, still, because we still aren't allowed to do any group activities, like we used to do a lot of exercise groups like cycling, swimming, gym. Then, we did indoor activities like art groups, games groups; quite a variety of different things like that. None of that has gone back. Probably each week, we get calls from people saying, 'Why haven't you been able to start the groups back up again?' They're still desperate to actually get out and do things and get out of the house.

Yes, well, Participantif you can speak a little bit more about the impact of the pandemic on the rehabilitation services, especially from a physical point of view.

Yes, obviously, in terms of the whole service, obviously, during this period, I think there were - although when I was redeployed, I wasn't actually working on a stroke ward. I was working on a general rehab ward, probably of which the majority of people were probably people that had had falls and fractured neck and femurs and things like that. The hospitals desperately trying to get people out, promising the patients and relatives they'd get rehab at home. Obviously, the reality was all the community services had been redeployed into the hospital so there were no community services for these people to get rehab. From a stroke point of view, we were still getting referrals as people were discharged, but we couldn't actually act on them. Over that time, obviously, we have developed a

massive waiting list. Obviously, we have been seeing people and getting people off the waiting list, but we developed such a massive waiting list over the last 18 months where we've had some services and then we've been redeployed back on to the wards; then back again. I think a lot of people have obviously missed that window rehabilitation.

They had the hospital rehabilitation. They had a few weeks of ESD. Then, everything has ground to a halt. By the time we've been able to pick them back up, they've deteriorated as well both, again, from a physical and mental health point of view. I would say a massive impact has been on our spasticity management care pathway. [Unclear word 0:12:27.3] clinics were probably in the last 18 months; we've been closed a year out of that 18 months. Again, we've opened and closed, opened and closed. We are seeing people that have got contractures now that are really fixed and that we're having to refer more people for surgery because we weren't able to inject. The picture in nursing homes is absolutely appalling. Obviously, during that time we had a number of people discharged from hospital to care homes and, in some instances, we're just starting to get out to the care homes to see them now.

Actually, they've got significant contractures, quite often pressures as a result of the contractures because we hadn't been able to get out earlier. Their general management in care homes is so bad anyway that we have seen a lot of issues of having to safeguard and Datix because of how people have deteriorated physically and developed contractures.

How about mobility, Participant? How do you think this might affect them in terms of their mobility and recovery in the long term?

I think it will definitely affect probably the majority of people that we had on our caseload at the time. Definitely went back in terms of their mobility and we've had to go back and start that again. I think the ones, obviously, that were the newer strokes, probably in some respects have developed a lot of bad habits; and have missed an opportunity to probably get more mobile than they would have done if they'd have had a clear care pathway of care, as we used to have prior to COVID. Obviously, because people haven't been able to go out anywhere. There's not been anywhere to go to; they haven't practised their mobility so their mobility is fundamentally worse.

From a therapist's perspective, can you please tell me about patients' needs during the pandemic in terms of rehabilitation?

They would have had all the usual needs. It's just that we weren't addressing them. Obviously, usually, we would do a - well, I suppose going back when we had ESD we would pick people up straight from ESD. With the more complex patients, we would have done a joint visit, as in their last ESD visit would have been our first. Obviously, we've completely lost all of that. Prior to the pandemic, we would have done a holistic assessment. We'd have been looking at their physical goals as related to the stroke, cognitive rehabilitation, physical rehabilitation, ADL, work rehab. In general, looking at a quality of life after stroke. Although we are back doing that now, for most people that has resulted in quite a significant delay. There's been a big gap between finishing ESD and starting our element of the rehab care pathway. For a lot of people, they have missed I think the opportunity to have progressed as much as they would have done had COVID not happened.

Okay, let us move, Participant, to another question about the strategies that have been used to manage the situation. What were the strategies that are being used to manage access to services during the pandemic time?

Well, I have to say that I think from a management point of view there was no strategy, really, other than the fact that they wanted us redeployed. They weren't really looking at what the strategy actually was. I think there was a massive gap in IT, really. We have eventually started doing a number of virtual groups. We've got two virtual groups a week going. It took nine months for the Trust to identify which - I don't know whether I'm using the right word - platform that we could use.

Do you mean a technical platform?

Yes, we weren't allowed to use Zoom or anything like that because there was, allegedly, issues of being able to hack that, so it wasn't secure from a governance point of view. Eventually, the Trust agreed that we could use MS Teams but it took nine months for the Trust to actually give us the okay to do that. Obviously, initially, we hadn't even got computers that had got cameras or the audio on them. We then had to fight to get that technology so we could do it. I think there was no management strategy. I think from our strategy we could quickly see how we could adapt and interestingly enough I'm a member of the Special Interest Group in occupational therapy for neurology. Fairly quickly, we were actually holding our meetings via Zoom. The first probably three or four meetings that we held were basically discussing and sharing how different departments had altered the way that they worked to try and

accommodate COVID. I have to say I do think our trust was a bit behind from the technology point of view.

I think we could see what we could do but we were - it took such a long time because of the Trust's...

Again, Participant, this was between different teams within the health system but I mean was there anything delivered to patients through Zoom, or Teams, or whatever?

I mean eventually, we did but it took nine months, as I say. Initially, we did it via Accutreat. Now, we do two virtual groups a week using MS Teams.

When did you start that?

Probably the end of last year.

How it's going so far?

Yes, very good. We do a stretch class which is a bit of education about spasticity and stretching. We used to do that, obviously, in a small group, but now we do it... Obviously, people are invited and they can attend as often or not as they want. It's done weekly. Actually, the majority of people do attend most weeks other than if they have other appointments and things. Actually, we will continue that because that has worked quite well and has allowed some of the people to actually do that that maybe would have had transport issues in getting into the hospital. On the other hand, there are people that we need to do that sort of thing with that haven't got the ability to use...

Technology.

...the technology to do it. There are fors and againsts [sic] on each one. The other group we're doing...

How you see that in general Participant, for most people?

I would say for the younger people it's worked really well. Having said that I would say that probably the average age of people that we're doing a lot of active rehab with, I would say is probably

40s/50s/60s. That's just - I think in Wolverhampton they have strokes very early! We have a lot of young strokes.

I think it became a general issue for many places; not just your area. It has been - even in the last congress it was raised to people, younger people now they have strokes.

Yes, so I think that obviously, that is something that's worked well. The other thing I'd say that has worked well is the fact that we obviously had to stop to do initial interviews over the telephone. Obviously, you can actually almost plough through all of the referrals for that week in a morning. Actually, we've continued to do that so that when we book an appointment, we actually really know what we're going to be doing in that appointment as opposed to just doing that initial interview. Obviously, again, you have issues where you've got patients that have got obviously a lot of speech problems and haven't got other relatives that can be there with them, so it doesn't always work. I would say probably out of our - we try and do our initial contact via telephone or Accutreat. Probably we're doing that about 80 per cent of the time. Ten per cent of the time we are going out to somebody's home because they can't; they've got communication issues and they can't actually get out of the house from an access point of view.

Probably ten per cent of the time, somebody's coming in as an outpatient. We will probably continue that where we wouldn't have done initial interviews over the phone prior to COVID. There are a couple of things that have changed that we will continue to do regardless of what the future holds, really.

Yes, was there any effort to introduce self-management programmes to the patient during that time?

Well, that's an interesting one. We have been trying to get self-management programmes going for the last six years. Wheels turn so slowly that we still haven't got anywhere. I've been saying that this is an absolute example of how if we'd have had those programmes that we've been - we've got them developed and we know what we want in them it's just the Trust's response to getting them done and the IT backup for it. If we'd have had them, it would have been a lot easier and I don't think people would have deteriorated in the way that they have. Because we can't give somebody an information sheet, unless it's been through governance - and that process takes about two years - so all the materials we've got together still haven't gone through that process so we can't give them out.

Even though, did you feel that you have delivered some elements of self-management, regardless of...?

Well, yes. We always do in the fact that whatever we do, whether we're doing physical rehab. Whether we are doing cognitive rehab, we have a session with a patient and they have their homework that they have got to do. Obviously, we carry on doing that. Obviously, the stretch class is an element of that because it's a self-management programme for managing spasticity and that is a part of it. We hoped to have a much better, more comprehensive programme together by this point but we haven't been able to do it. Obviously, self-management I think is a vital part of rehabilitation.

I think it is a time, during the pandemic, to maybe invest on this programme, on self-management programmes. Especially when you have to deliver services remotely and people, they have to get more independent on their self in delivering their therapeutic exercise or home programmes or whatever.

Yes.

Participant, how do you see some efforts that you have been delivering some self-management strategies or using some of them, how do you see the benefit for patients?

I think that's a vital part of rehab. I think as therapists - if you have a stroke, whether you're looking at physical or cognitive rehab, that isn't something that is time-limited. If you're going to improve somebody and then maintain that level of improvement then it's something they're going to have to work on, on a daily basis. Actually, I feel it's the therapist's role to give the patient and the patient's family the tools to carry on doing that in the long term. It's when they don't do that, that things then, they start to deteriorate again. You want to keep an upward improvement going, even if it's a slow improvement or maintenance. The problem with stroke is if you don't carry on doing your walking, doing your stretches, using that hemiplegic arm, and doing your exercises then you will deteriorate. I think that self-care and self-management is a vital part of stroke rehabilitation.

Was there any effort particularly taken for mobility/to improve mobility? Like using some strategies including self-management or other strategies in terms of mobility. To be honest, we have some interest in discussing mobility as a big issue for stroke people.

Right, okay. I suppose the way we divide therapy up between ourselves and the physio; the physio probably would concentrate more on mobility. I suppose we tend to work a little bit more on mobility when it's like looking at mobility to get somewhere. In the past, we would have done a lot of mobility practice in - say them walking to a local shop to go shopping and things like that or getting the confidence with the mobility to get back on to the bus. Or, to go and visit somewhere or whatever their goal was that they wanted to go to. I suppose we divide it like that. Probably, the initial strategies would come from physio. Then, by the time we're getting involved with mobility, it's mobility to get somewhere and do something. Again, one of the difficulties we had that we have only just overcome in the last couple of weeks is we weren't allowed to go into shops with patients to do mobility practice; to practise getting into a shop and do shopping. We were not allowed not to wear uniform with a patient and full PPE and we couldn't go in in a uniform and full PPE into a shop.

It's only in the last month that we have been allowed to do what we did previously which would have been - if we were doing shopping practice or transport practice, we wouldn't have worn our uniform. Over the last month, now, we don't, we just wear a mask. It's taken a long time to get back up to that. I suppose our strategies have been delayed because of these protocols in what we could or couldn't do. I suppose in terms of the strategies we use with mobility it's been the same in that if somebody's got family, we would initially go out with them and then expect them to do that with the family; gradually get in a bit further distance until they reach the goal of where they want to go.

Great. Participant, let us just sum up. I just want to see from your perspective if you remember anything that you considered as example of a good practice of delivering the services or maybe dealing with the patient during the pandemic. Could you think of anything that you might feel it was a good practice and it was really a good thing?

Yes, I would say the good things that have come out of it are that obviously we now do the initial contact via telephone or Accutreat which focusses the first appointment more because we know exactly what we're going into do. The stretch class, which we will continue, that's done by an OT assistant and we do two sessions a week. One is for people that know the stretches and know what they're doing and have been practising on a one-to-one or one-to-two basis online first and when they know what they're

doing they go into the big class. The therapist delivering that virtual class can't see people well enough to know whether they're doing it right or wrong. Once you're confident that they can then they go into the bigger class. That works well. We'll definitely keep that going. We used to use a lot of volunteers and the volunteers are doing a games class virtually every week. That has got to quite a few of the people that we wouldn't have been able to get to and, actually, it's a lot of fun.

It's given them the ability to contact and chat amongst themselves again so that has been good. Probably, if there is a time in the future when we are able to get back to doing groups, we probably still will do something like that online, as well, to reach the people that actually can't get to a venue very easily. There have been some gains from it. I would say those are the things that have come out as good practice.

Yes, all right. Then, let us go to another area of this interview. It's about when the services go to normal, like as prior to COVID. What would be your priorities for stroke rehabilitation or for delivering services for rehabilitation?

I think our priority at the minute is to get the waiting list down so that we can go back to what we did previously in that we were picking up people as they finished ESD, so it was a seamless service. Obviously, we are a long-term service so we also see people in the long-term in spastic clinics and splinting clinics. Again, we've got massive waiting lists for those. We are looking at five months is the next available appointment for them. We really need to get back to that more responsive way of working that we had previously but, obviously, we just developed such waiting lists over the lockdown period that it's going to take, I think, quite some considerable time to get back to doing that. My first priority would be to get the waiting lists down so that we can respond very quickly to need. The second, I think, is to actually get some of the groups back up and running that people are desperate, from a mental health point of view.

It's bad enough to have a stroke, but to have had a stroke in isolation - and not being able to share those experiences with others that have had the same sort of thing and feel very on your own - has been really challenging for people. We have seen, with a lot of people, we haven't got as far as we would have liked from a physical point of view because of their issues around mental health and the reality is that those don't get picked up either because those services are overwhelmed at the moment. I think those would be the priorities. We used to have upper-limb group that people really worked well together and supported each other. Obviously, we've lost all of that. We're doing it on an individual one-

to-one basis. We had cognitive groups. We had all the physical exercise community groups in different parts of the town. It would be great to get back to that again.

Hopefully. Hopefully, we'll see that very soon.

Hopefully!

I just want to ask another question, Participant, about if we have any other wave or pandemic and hopefully won't see that in our life again, but let us think if we have another wave of the pandemic what would be a good plan for optimal services?

I think should it happen to the degree that it did before, at least we probably are a bit further forward in terms of technology. Hopefully, that would make it easier to respond to in some degree. I was going to say something else then; I just forgot what I was... Ask me the question again, I've forgotten what I was going to say.

Yes, I mean if we get another wave or another pandemic, what would be...?

Oh, yes. Yes.

How we can ensure optimal services?

I think the thing that's - obviously the overwhelming numbers of people that were in the hospital with COVID or if it happens again with a different sort of pandemic, so many services were stopped in order to deal with what was going on, on the wards. The challenges of that are - everybody's got their own speciality. You were asked to go and do something very different that you probably weren't doing that well because you didn't really know the systems and how to do it. The community element, from my point of view, was so neglected and it's just as important to provide those patients with services as it is with people that are an in-patient. Do you know what I mean? I think there have to be lessons learned about trying to continue with community services and not redeploying staff enough...

Do you have a good plan to manage that? It is hard when you have social distancing measurements in effect. It might be difficult to ensure that. Do you have any example or any plan to deliver that?

Obviously, the plans don't come down to my level. I just think it's interesting that in the wider context of ESD they do a very similar job to us, only we do it six weeks later and for far longer. Yet, ESD staff were never redeployed. Equally, some other community teams weren't but stroke was! I just feel that if you have - as I know a lot of the other neurological community teams were stopped as well. If you've got a long-term neurological condition or a stroke, you still have those needs that were completely neglected; certainly, for the first five months of the pandemic. I think we need to make sure that doesn't happen again in further pandemics.

Yes, yes. You're right. How about people who are in isolation if they need to be isolated? How we can manage their rehabilitation?

I think it's... Do you mean, actually...? Actually, do we not provide because of the risk of going in or for isolation [over speaking 0:39:29.5].

Yes, or maybe they are positively tested for the maybe COVID or any other thing. How we can deliver, maybe, their service?

I think, well, if you've got somebody that is in isolation it's difficult, isn't it, if they are high-risk. You would hope that what we'd be able to do now is to be able to test more accurately. We're doing LAMP tests now, but the results - like I did my last LAMP test on Wednesday and I still haven't had the result. I think actually one would hope that you could respond better. It's patient choice, isn't it? I was told by my managers these patients will not want to be treated. They won't want anybody coming into them. What I was getting, particularly during the first lockdown, was patient after patient, after patient, phoning and saying, 'We are desperate to be seen. We want to be seen.'

Wow.

It is a difficult balance, isn't it? They knew that if they didn't get the rehab that they needed at that time after the stroke that they were going to end up less mobile, less independent. They wanted those services

in. Now, they wanted every risk that you could actually do something about managed well. One would hope that, by now, that we would have devised a much quicker, more accurate way of detecting whether staff had actually got COVID or whatever the disease is that we get in the future. So [over speaking 0:41:25.2].

Hopefully, we won't have anything!

Well...

Let's just...

You could get...

...hope.

...into a whole new environmental issue about that and so, yes, the increased risks of animal viruses coming into the human population and, anyway, that's another story.

Well, thank you, Participant. Thank you very much. I actually have done with my questions for you. I'm just giving you the mic if you want to add anything or you want to share anything that you feel I have missed during this interview.

I'm just trying to think. I think from a patient point of view, they definitely have found it very hard. The poor access to services, their lack of social interaction with other people at a time in their lives when they really did need that support has been very challenging. I think it's been challenging for staff because we've partly felt that we've been letting those people down; that we've tried to do what we can. I think we've also got people - the staff on the grassroots level have also got frustrations with management, that probably have got very different agendas and didn't really appreciate some of the issues, really, that was happening out there in the community. Yes, I think it's been a very challenging...

For everybody.

And continues to be a challenging time. I think because we are now in a situation where they've changed how we work and we have to see patient after patient, after patient. We have no development time. We have no training time. We have new staff that have come into the service that has been fairly newly qualified and, actually, aren't getting the training and support that they need either. I think all around, COVID has caused a lot of challenges, really, and we hope it doesn't happen again!

Yes, it did.

Equally well, I think we are still a long way from returning to any sort of normality of services, as well. We are being told we won't be able to do group things. Because of that, because we use groups such a lot, we got through our patient caseload far quicker.

Yes, especially with the large number of referrals. I think group [over speaking 0:44:15.9].

That's going to impact on staffing levels, but there's not going to be any more money to pay for additional staff, because we're now doing one-to-ones where we used to do groups. It's a challenging time all around, really.

Yes, it is challenging for everybody. You're right. Thank you very much, Participant, for your time. Is there anything you want to add or...?

No, I think that's it, thank you.

All right. Again, thank you very much. I'll end the recording.

[END OF TRANSCRIPT]

AS: We can hear you now just state your name and that you are OK with recording this. It is just a type of consenting.

AT: So my name is Participant and I'm happy to be recorded.

AS: Alright thank you very much again for taking part in this study. There is no true or false answers, we only interested in knowing your experience and your thoughts about stroke rehabilitation during the pandemic, so feel free to express whatever opinion. Also, your name won't be presented in any

paper or report of this study, so we'll make sure we are not using any names. And you have the right to stop the interview at any time or if you feel you need a break or something, just let me know, okay?

AT: Okay.

AS: Are you ready to start?

AT: Yes, I'm happy to start.

AS: Alright thank you Participant. So could you please at the beginning just tell us about the stroke care and rehabilitation delivery services since the first wave of the pandemic that happened?

AT: So I worked in an acute hospital trust. I'm the band 7 OT across acute and inpatient rehab services for stroke neurology, so I follow patients through that pathway. During the first wave of COVID, we definitely notice the reduction in the amount of admissions to the stroke unit. And sort of anecdotally and also speaking to patients, I think that was very much due to being scared to come into hospital. They were being told to stay away as much as they could, so I think people were trying to manage things at home, trying to reduce some of the pressures on A and E. And I think that obviously has had implications longer term for those group of patients. The kind of environment was very very different to the usual experience that our stroke patients would have, as you would imagine. So I think just the environment in terms of staff wearing FFP 3 masks, and gowns, and aprons, and gloves. I would imagine and patients have said it was quite a frightening experience for them. And main communication with our patients very very challenging, they couldn't hear us, things were misinterpreted, just that not being able to pick up on body language, all of those things were quite problematic.

AS: If you can tell us please about the situation of the delivery for the stay for inpatients after stroke, and if they had to maybe discharge earlier than usual at that time?

AT: Yes so um there was a definite feeling of a push to discharge people early, I think. Because obviously being in hospital put these groups of patients at greater risk. So on our unit we had one of our bays was turned into a unit for COVID patients, so we had a mix of patients, either with or without COVID. The ones that didn't have COVID obviously there was a push for a quick turn around and to get them out. And often we were having to do that based on very different ways of assessing our patients and maybe not quite in the same way as we would have previously. So as an occupational therapist, obviously we're looking at functional assessment, so those things were limited because people were discouraged to bring in personal belongings, to wear their own clothes. So things like personal care assessments were very different, because we were assessing based on a hospital gown that people don't wear at home. We would usually bring people to our therapy kitchen, again we couldn't do that. So, I think as a therapist working to turn around these patients quickly, it felt a little bit more challenging because obviously you want to give justice to the patient, you want them to have what they need, but you're also balancing the risk of contracting COVID in the hospital. You also don't want to miss things by not being able to assess, perhaps this thoroughly. So, there was a big push obviously towards our community service. In our service we have an early supported discharge team that comes under our umbrella. And again the difficulty there that there was a lot of their work moved to virtual visiting

AS: Was that inactive during the pandemic? Like were they doing the same way as usual?

AT: In the community team, no. So it was a big push towards virtual visiting, so on setting people up with programmes and coming and checking with them online.

AS: How about the early supported discharge team, who were in inpatients? Are they also using the same way of treating people or they had to switch to another system maybe?

AT: So the early supported discharge team is the team that switched to virtual visiting.

AS: Oh I see, OK. And also the community therapist as well?

AT: So the discharge team is our community team for a certain group of patients. So, there is a specific criteria for early supported discharge in our trust as everywhere nationally, so independent mobile, transferring with one, have a GP that comes into our catchment. So the more quick turn around patients would go through to ESD, the community team, but they would be seen virtually via WhatsApp or those sorts of platforms which is different to usual because they would have been visited. So whilst the push was to get people home more quickly, we also knew that their usual rehab contact was different when they left hospital because it was move toward more virtual. Does that make sense? And then our longer term patients, so the more slow rehab, there was the potential for them to go home more quickly, more dependent, but to have community therapy or to be set up in a more compensatory way to get them home and out the hospital. And then we had a group of patients who had a stroke and COVID and they stayed with us for a very long period of time. As you can imagine with all of the precautions in place ,it was very difficult for them to be able to participate in rehab; one, because a lot of them were fatigued and didn't have the energy reserves to participate; and sort of the other side of that is we weren't able to offer the diversity of rehab that we would have had they not had COVID or being in that situation. Did that answer your question?

AS: Yes, thank you very much for this. I was also wondering if you can tell more of maybe modifications to the usual system at that time when COVID came out. So what other modification to the daily system has been in effect?

AT: Yeah okay. So, all of our groups stopped, so we have quite a few rehab groups, we had upper limb group, breakfast group, lunch group, all of those obviously had to stop.

AS: Completely stopped?

AT: Completely stopped, yes. All groups were suspended. Initially, we stopped taking patients to the therapy gym. So on both of our wards, we have a therapy gym where we normally take patients to access the plinth, the tiltable, a quiet space. So all treatment was at bedside. That's changed overtime, but that was very much in place back then. We stop taking people to the therapy kitchen, that again is slowly being introduced with the appropriate risk assessments, but patients are still wearing masks in the therapy kitchen. We have just restarted the upper limb group, and again a very strict risk assessment is being put into place and people have to wear masks in there as well. There was a shift just in terms of MDT meetings, because we have to reduce the number of people present in those

meetings to try and get that social distancing, so not everybody who was involved with patient care was always present. And there was a big shift to all meetings went to online. So it was a very long time before we got to meet face to face with people, which was a different dynamic actually, and is practise that we continued some what.

AS: How would you see that? Do you think it's like OK or it has maybe some impact on the quality of services?

AT: I think it's a bit of a mix really, if I'm honest. I think there's nothing that’s going to replace meeting face to face and networking from a face to face perspective. Obviously doing things virtually, you’ve got the screen that is in between those two people, it is difficult to pick up on communication and body language, and the subtleties of conversation. But on the other hand, you don't have to drive to a different site to attend a meeting. You can pass to attend a little bit more, sometimes there are shorter meetings because no one really likes this online. I think it's got pros and cons, and this definitely changes that we made during COVID that have continued ongoing, obviously we're still not fully out of the situation, but we obviously started making strides to normalise practise a bit more, so a mix.

AS: Participant, we will come back to this point of how you have developed strategies to normalise the way that you treat patients. But let us move to another point from patients perspective. If you can think during that time when the the wave started, what do you think were the problems for people who have had a stroke during that time in accessing services and rehabilitation.

AT: So from the inpatient setting from where I worked, I think those patients who perhaps English isn't their first language it was really tricky, because initially it was difficult to get formal interpreters to come to the ward. We were having to rely on things like language line, which is incredibly difficult to do some of the assessments that we do like cognitive assessments and functional assessments as a 3-way phone call. I think patients were incredibly isolated and very much felt that. There was a big impact on a higher percentage of patients’ mood, they just wanted to go home and be with their family. So I think the impact for those where English isn't their first language was actually huge, also from a rehab point of view, because they didn’t always have people on the ward who they could actually communicate with. So I think that was an issue.

AS: If you think about the impact of the pandemic on rehabilitation services for stroke survivors, what was then back during that time on their rehabilitation?

AT: What was the impact of COVID?

AS: Yeah, on rehabilitation for stroke survivors.

AT: I think it's the things that I've said really. I think it's that perhaps those who might have stayed slightly longer might have been discharged a little bit earlier. That wasn't always negative for everyone, but it was certainly a consideration when you are looking at discharge. Just accessing equipment was tricky, because of getting things delivered and there was a bit of a shortage of stock. The considerations about who's going to support this person when family would normally do that, because they go home to isolate for example. I think I'm not sure whether we didn't see quite as many patients in a day, whether the impact of donning and doffing all of the PPE actually meant that we saw slightly

less numbers per day. Maybe people potentially had slightly less rehab. I haven't got any stats, so I wouldn't bet on that, but I think that might be a consideration.

AS: How do you think this might affect their progress for long term, generally speaking? I know for some people it might not be a big deal.

AT: I think it’s difficult isn't it, because what we do know is that people benefit from rehab at different stages of the recovery pathway. And if somebody is preoccupied with wanting to be at home or are scared, actually then potentially they are not going to be able to access rehab in the same way anyway. So sometimes it's removing those barriers to allow someone to engage. I think obviously, the earlier we can get in and the more we can do is always going to be beneficial, but that doesn't necessarily mean that that will happen and that's true for everybody. So it certainly impacted engagement in rehab, definitely. I think that would be a very person specific.

AS: But again we have some interest in this study to see them back on mobility recovery. In particular, if you can speak about the long term effect of the situation on mobility for these people?

AT: From my perspective, I'm not sure I've seen the outcome when people leave the hospital, so I'm not quite sure where people have progressed and whether there has been slower recovery or not, does that make sense? I think from an impatient perspective, again it depends on the group. For those who had COVID and a stroke, their mobility obviously was severely limited by fatigue, mainly that was what we found. So that was a big impact with that group of patients. For the people who came in with perhaps a lacuna stroke who were up and mobile within a few days, we could get home. I’m not sure if there was a massive impact on that group of patients, but I'm not sure we've kind of got the stats, or the data, other feedback from community services and patients to really answer that question with any conviction really.

AS: Yeah, no problem. Participant let us move back to the point of strategies that have been used to manage the access to rehabilitation services during the pandemic?

AT: Hmm......strategies. Well I think we gave a lot more self-directed programmes that people could do either within hospital or once they were discharged from the hospital, which were then followed up. And then the strategy from a community point of view was that they move to online, virtual visiting. And they would try and do as much as they could that way. From a discharge point of view from the hospital, we moved to virtual access visits where we could. So, we would video call the family, get them to show us around, rather than actually going and visiting the property. We would do lots of video calls with family in therapy sessions, and we found that that was quite useful because one of the big problems that we found was because patients relatives couldn't come on the ward. When that patient was discharged, they were quite surprised by how their relative was following their stroke despite what we thought was quite good communication and showing them virtually and things. So from a family perspective, that was what we tried to introduce. We had to switch all of our family meetings, so we used to do family meetings with the patient looking at discharge and setting goals and things like that, so that all went to virtual. And we tried as an MDT to do lots of weekly updates via telephone call, so one person would be nominated and would give feedback from the team. So, I think a lot of our strategies changed in terms of communication with family actually. I think we still went to the patient and we still did the rehab that we would do. Obviously if someone was in a side room,

they're not able to come out that would limit. What we could do in terms of getting them into the gym, into the kitchen, we would do what we could do within that area.

AS: So what about the time of discharge, when you are preparing them for discharge from hospital to their community, to their homes. If you could speak what were your priorities for them, or what are the things that you are keen to teach them, or to maybe advice them at that time before the discharge?

AT: So i think there was a big move towards giving more education whilst they are in inpatient, so that they were more informed about things like signs, symptoms, FAST, things to look at in the future, and perhaps more focus in terms of medication, again who to liaise with. So I think there was lots of information given and trying to empower someone, whether they were able to more self manage.

AS: From what you're saying, I have seen like you have used a lot of self management strategies like patient education, goal setting, and probably self-monitoring and follow up by phone, like weekly phone calls. And so we will get to this, but I want to see the impact of using this self management strategies. How did that go with patients from your experience?

AT: Again is very variable isn't it. Some people really respond to that, and other people don't. I think it's been more effective where they've had family, or they lived with someone and they can actually help encourage them and talk through with how to do it and to do some of that self-directed work with them. We of course have some very motivated patients, but they almost do more than we would want them to, so that almost creates a different issue. I think I'm not sure COVID impacted that, I think that's just what we see usually. I think the only way COVID impacted is we were doing more of it, we were giving more self directed work. Some people did it very well, some people didn't. As I said, those with family or friends or people who could support did it better. I think upper limb work people did more self-directed; cognitive work, no. But maybe because of their level of insight and awareness into what they needed to do and why. So, the cognitive rehab stuff probably would be therapy led, generally.

AS: Yeah, it's quite difficult to deal with that with self-management. Was there anything specific, used specifically, for mobility training in terms of self-management? Was there any self-management strategies that were used specifically for mobility?

AT: Not specifically from an occupational therapy point of view. I think the physiotherapist probably would be the better placed ones to look at that. I'm just trying to think if there's anything specific.

AS: Sorry I forget that you are an OT. If you could have seen any good practice with that from your colleagues or maybe from your patients, you can tell us with that.

AT: Yeah, I was just trying to think of anything that was different or sort of not in the normal route, and there's nothing that specifically that jumps out. But from my point of view, mobility wise is no; it would be normal practice from our point of view. Physio might be able to answer that a little better.

AS: We actually have some participants who are physios and some OTs, so we will cover that with them. Yeah, don't worry about it. Participant, let us move to expectations. If we have a global,

hopefully won't have any other waves in future, but if we have another pandemic or another wave of Covid and we had to apply social distancing and lockdown measurements, so from your point of view, what should be the priorities for health services for people after stroke, if we have any other wave or lockdown? What would be the priorities for stroke rehabilitation during that time?

AT: Well, I think the priority has to be about maintaining the rehabilitation of these patients, encouraging them from the first point of view to actually access stroke services, so education, through the GPs, all of that sort of thing. I think rehab as I said did continue, I think it's the balancing act between “where's best and safest for that person at that time”. I think from an inpatient perspective, people did receive rehab. I think where perhaps things were different to the norm was when they reached community, because things had moved to online. And actually that's probably where, if we could do anything different, being able to see people on going in their own home, would be the better thing for these patients. How we do that......

AS: that is my next question.

AT: That is your next question, “how do we do that?”. So the community team has returned to visiting people, so it has been a process. So initially, there was a very strict process in terms of wearing your FFP3 mask, your gown, your gloves, your apron, which you have to put on at the front door. And then you obviously had to take all of that off, you had to gel your hands when you came out.

AS: It is not easy.

AT: It is challenging and it's something that we've all done. But the other side of it is patients and relatives don't want healthcare professionals in their property as well. So whilst we want to be there to give rehab, they are scared about what we're bringing into their home. So I've got a bit off a track there, but I think that is also important. There is people who have refused onward referral, because they don't want people in their home, and they haven't got access to technology or they are not in a position to use that. So they've really missed out. So I suppose going back to your question, is how we can access those groups of patients to meet their needs whilst balancing against their wishes and the potential risks. How we do that, I don't think I have a black and white answer. I think some of it case by case. I think it's about being creative with our rehab and working with people. I am not sure if I'm really gives you the answer you wanted.

AS: I know it is quite complicated with the nature of stroke, and how people after stroke might vary on that scale. So some people are motivated, even in normal like daily life, without having Covid, some of them are motivated to do self-management and to be contacted weekly, from by online resources or by phone. And some of them are less motivated, or they might refuse to have their therapy at all. So, it's quite of balancing between those two parties on the scale.

AT: I was just going to say I think it's about doing the best you can with the resources you have. And online virtual visiting, is better than no rehab. It might not be what you think is the best, as in the therapist thinks it's the best. But at least someone has regular contact, they are being monitored, they are being guided. That may be as good as visiting twice a week.

AS: In case, Participant, if we have another wave and you get people who are isolating at that time. So what would be your strategies for delivering the rehabilitation to these people?

AT: So the people who go home who are isolating?

AS: Yeah, they are isolating.

AT: Isolating in terms of COVID or?

AS: COVID yeah, if they are tested positive.

AT: So we do still have COVID positive patients on the ward, and it does keep happening recurrently unfortunately. And when that happens, any discharges from the hospital within a certain set time, they have to go home to isolate. So in that case, we do still refer onto community teams, they make telephone calls. We have stroke nurses who follow people up the next day of their discharge to deal with any sort of medication queries or issues that ever risen. And that's quite a new role to us, a bit of a safety netting procedure. So that's something that's new and it's working really well. We would then refer on to community services, so depending on what level of dependency the patient has, it could be a generic rehab service, or it could be our early supported stroke discharge team. And again, they may contact by telephone quite quickly, and they have the option of them visiting. And patients usually, their first face to face appointment is in their own home, so they are being seen at home, and then it's about negotiating joint goals, self-directed work, frequency of visits. So right now people are being visited. And all the way through the pandemic, there was the option if somebody needed urgent care, then we could go in, because we had the appropriate PPE. So it wasn't that we couldn't, but I think to manage risk of staff and the patients and their families.

AS: In that case, you had to maybe test them for PCR or something, or just go regardless what is their situation?

AT: From a staff point of view, we had lateral flow tests, and we have lamp testing now weekly. From the patient perspective, we didn't get them to do any sort of test.

AS: Yeah I was asking about patients.

AT: So, no we didn't get them to do any sort of lateral test or anything. We would always do screening questions, so they would phone before they visited, and they would ask a set amount of questions, just the COVID symptom. And it was very much reliant on patients and their families reporting things. If they did develop COVID, or there were symptoms and they were awaiting a PCR test, their therapy was almost put on hold until people were able to then go back in. But obviously in the hospital, it just continued.

AS: Yeah, alright. Thank you very much Participant. I'm done with my question. I'm just wondering if you have anything that you think we have missed about your experience with rehabilitation during COVID pandemic? So if you want to add anything?

AT: I would imagine there is lots of things that haven't quite come to mind. I think really just to summarise, from an inpatient perspective, people continued to get rehab. In some instances, patients were seen more frequently because we had less admissions, that changed very much, and then we became inundated, and it became almost impossible to keep up. We had lots of stroke outliers on other wards. I think the team responded really well, because it was a very frightening time for staff as well as patients, and I think it was really really good actually. And I think there were lots of things that we learned that we can take forward to improve our practice. It wasn't all negative, we've learnt lots like zoom meetings are quite good ongoing. We obviously had the opportunity to tighten up some of our processes and risk management. So I think there's lots of positives. It made us refocus perhaps on what's the right thing for the patient and not just "they need rehab". Yes they do, but is that the right thing for them at this point? Or do we need to do something else first? And as I've said, as an occupational therapist, our tools of occupation were really limited, because we couldn't do those things with people. So, the way we assessed and we made decisions was different, and for some that worked really well, for others it was more challenging, but we kind of come through that together. And yes, I think that's the summary really.

AS: If you remember anything just email me later. Thank you very much for your time.

KM: My name is Participant and I'm happy for you to record this interview and use it for your research.

AS: Alright, thank you very much again for your participation. So at the beginning can you please tell us about the stroke care and rehabilitation delivery services since the first wave of the pandemic. If you could just telling us what is the system at your workplace and how that was affected by the wave of COVID-19?

KM: Yes. So the first wave, which come along in the March, at that point I wasn't actually on the community stroke team. I was in a different community service. And then, when I moved across in August, what the guys were saying the experience seemed quite similar, that when the first wave hit it was all kind of......we were told not to go out and see patients, everything was kind of put on hold until they figured out exactly what we were allowed to do, the risk versus the benefit. And they actually repatriated quite a lot of staff from the community, they pulled them into the acute setting, so community kind of like rehab was left on the backbone so to say. There weren't enough staff sitting in the community to continue to do what we needed to do. Because at that point of the first peak, the priority was clearing the hospitals and making sure we have enough, kind of like "hands on deck" to see the patients and treat the patients. They kind of took priority.

AS: So at that time, like the service was affected completely by that wave, and to some extent maybe people aftershock didn't receive maybe any, or maybe they received only limited services at that time, right?

KM: Yeah. When I moved over to the stroke team, we the services at that point were up and running, as in the ESDs were still coming out of the hospital, CSTs were coming out the hospital. But from what the feedback from other patients were saying was they were just trying to get people out of the hospital's sooner rather than later, because they don't want them to be sitting there and contract COVID. So their experience it was kind of a straight-in straight-out, a bit of a whirlwind. When they had

a stroke anyway, it's a whole new world for them to kind of process and get to grips with, and being kind of in and out of hospital so quickly, that kind of set the people back a little bit. When they come out, they were quite keen to engage, cause they appreciate the rehab makes difference, and it was quite interesting the views on people and they were just a bit like, yeah, “we don't really mind” should I say, rather than care at the risk, they would rather come in and see us. This team I'm currently on now, when I joined the team they had a little bit of a waiting list, and that was because they had no staff. The service was completely depleted. There was a bit of a backlog, so for the ones that were sat on the CST pathway, they had to sit and wait longer than what normally we would have liked to have them waiting for.

AS: Was there any like new plan from the NHS, or maybe other community services for these people? Starting from wave one and during the lockdown one and two, there was any maybe backup or maybe any other plan for these people? What was that the plan at that time?

KM: No, it was just almost an initially it was “sit tight until we know” who you can go in and see. We kind of put a system in place, which was “red amber green”, and that was more to do with kind of like their COVID status. So if they were green, we could go straight in once they were discharged from hospital. Amber the vulnerable, and red would be even if they are showing signs of COVID. So even that, from the discharge from hospital, there were still a little bit of a delay getting into them if it’d been swabbed, if they’ve been in contact. So yeah the initial coming home from hospital, for us getting out to them, there was more of a delay than what they currently is now, because we've got the PPE, we can get straight in. The guidelines, half over the last 18 months just forever have been changing and fluctuating, and now for us we've adapted it it's not as much as “all covid is big scary thing”, it's kind of like we're learning to slowly live with it.

AS: So things are getting better now, I hope. At that time, at the beginning, I think it was shocking time for everybody, and maybe even for the recovery plans I think it's usually after a period of waiting, and everyone was uncertain about their plans and how things will be going at that time. So, Kristy, if you could tell me from patient perspective, maybe be with people who you have seen that you have been working with. If you have learned about their needs, and maybe their difficulties and challenges at that time. What would be their needs and challenges during that time?

KM: So, a couple of patients that had a stroke post like the first wave, they'd been on waiting list for total hips, the knees, and they were due to get an elective procedure, and then in the meantime they kind of had a stroke, and so for them COVID set them back even before they had the stroke. There was a lot of frustration in regards to that. And for some of the stroke patients that have made a good recovery, we've got them to the point of, say it was an underlying hip issue or the knee issue, that that is their main burden at the moment, and because services aren’t still up and running, they have a stroke, they have gone through a year of rehab, and we can’t progress any further because that limiting factor of their knee or hip is still there. Access, even just in regards to more urgently, if we needed the GP, if we thought that more of a face to face and was needed, especially early on in COVID, GPs weren't coming out, we certainly didn't have that support from the GP services. And then onward referrals, as in if we felt that a patient was safe enough and we didn't need to continue to treat, but they wanted to get back into some sort of group setting, there is nothing, no, none of this community kind of like groups exercise classes that were up and running. If you want to do a bit of therapy in pools, we didn’t have access to any pools. I was trying to think of the higher end ones, if they were looking to

return to the gym and stuff like that, it was just more of giving them the guidance of “when the gyms is opened, this is what you can do”.

AS: What was the way of communication with these people, like would the patient be referred to you first or they’re supposed to get their therapy by you or your team? What was the way of communication, or during that time, what sort of communication you have had with them?

KM: Yes so we have done a mixture, there's quite a lot of tele-therapy. If they needed a face to face treatment, we would have to triage and justifying reason, especially in the early days why we're going in.

AS: And was that for everybody or for people who, like there was a priority for people who much needed the therapy, or this was for everybody?

KM: In the initial kind of like wait bit, because they didn't really know “what we were meant to be doing? ”, “should we be going into people’s houses? ”, “what is the risk towards us? ”, “what is the risks of the patient?”. We had to kind of like clinically reason; “If we are going to see this patient face to face, is there a risk of them regressing?”, “do they need that hands on therapy?”. We had to kind of make that decision. Or if we felt that for a few people that were ongoing, they could oversee their own rehab just with a little bit of contact over the phone, or even through zoom, just so it was reducing that contact between the patient and the therapist.

AS: Tell me about your perspective, how do you see that went, went ok or maybe, assuming for some people it might be difficult, especially old people who are not familiar with technology and tailored rehabilitation stuff?

KM: Yeah absolutely. And it was patient dependent as well, whereas some people were more able to get on with stuff, but with other the people you couldn't do that with them. So with all the greatest will in the world, if you say “no, can you speak to family or relatives over zoom?” or anything like that, the effectiveness of the treatments, in my own personal kind of like experience, it weren't as good.

AS: Yeah, and also I think as a therapist you might be worried about their safety if you maybe prescribe maybe some sort of exercise or something for them. You might be worried about their safety.

KM: So certainly earlier on when we were trying to say do a lot of the initials over the phone and prescribing exercise programme for more of the higher end ones. Even still that point, yeah it's great you can do that, but you don't know if they're carrying exercises out correctly, “are you just going to run some more problems further down the line?”. It doesn't matter how effective your communication is, ultimately people interpretate things slightly different. I feel it was a great way to try and overcome and keep patients almost that contact with the patients. But over the long term, I feel it certainly isn't the way forward. Some good things taken away from that, but yeah.

AS: Yeah. Kristy I have a question. I have some interest about mobility impairments and mobility rehabilitation after stroke. And if you can speak a little bit about mobility for people you have been dealing with, like how was their mobility if they can have rehabilitation during that time, during the

pandemic, when you only provide the services maybe by phone, or maybe by zoom whatever, or tele-rehabilitation? (-16.34).

KM: yeah it's, well, I mean isn't it people coming out of the hospitals are all different levels, and you can't exactly progress someone if you were discharged on a Zimmer frame. You couldn't progress them over the phone onto a walking stick, you just literally couldn't do that, you'd have to do that face to face with them. So, yeah, I think for us it was kind of like even though we were asked to reduce contact where possible, and for a lot of our patients that were coming out, we could clinically reason that we have still got to go in and see these patients. We might not necessarily have seen any SD as much as we would want, but it was just getting into that safe level and making sure that they're still doing something. I was just thinking equipment wise, if you needed equipment I can't think what the OTs, I think I'm sure NRS and all the equipment services am sure they were still up and running. So if we needed certain aids, I don't think that was affected.

AS: Kristy, if you can clarify me with this point. You're supposed to see the people after stroke in the community when they discharge from hospital, right? And from acute maybe care, right?

KM: Yeah.

AS: My question, do you think they were able to discharge to their homes at that time when they move from hospital to their homes? Do you think this kind of preparation was quite well for people who you have been dealing with?

KM: it's a bit mixed. So, for some people they would get them home, but for the more severely impaired ones where even if they didn't have the care available within the community, they would get put in the homes. And then the problem with that was people getting put into care homes, the care homes were closed and especially in the first wave, even to healthcare professionals, they just kind of shut the doors and just said "no one is coming in, no one's going out unless they needed to go to hospital". For them individuals that got put and discharged into care homes, they were at more of a disadvantage until we were allowed all the care homes had put into place, kinda like their screening process. They could have been sat in the care home for 4-5 months without any input, and we know that input is really needed, it's vital after a stroke. So they were put at a disadvantage, and it's quite a lot of them patients, that when we eventually got to them, that they've been that deconditioned it's deemed that you know this is their new baseline, they don't actually have any rehab potential.

AS: So in this case because I was wondering, this is one of the questions for my interview, about self-management strategies, especially for people who are with maybe mild to moderate impairments after their stroke. So, have you introduced any sort of maybe self-management strategies for these people, while you cannot see them face to face?

KM: Yes, that's just generically even now and even before COVID, if they're higher end in cognitively they were able to do stuff, we would always set them up on a home exercise programme self-management, self-directed. That didn't really change and that is how we kind of have to work in the community as well because we don't have the luxury of people being in our disposal on a ward. And we can't get out of see people as much as we would like, especially in Hereford, the traffic is ridiculous, cause people live so sparsely. It would just be getting family involved, if they were happy to do that,

and then asking carers to do little bits if they could, well that would always affect if carers, you know different carers coming in.

AS: If you have had a chance to see them face to face, at least for one time or two times, would you suggest self-management strategies for them like to invest with self-management strategies that are over there, maybe prescribing some sort of self-directed therapeutic exercises programme, or maybe involving them in maybe self-monitoring and you know just following up by phone or something with them. Would you encourage them?

KM: Yes that's kind of like, even now that's kind of what we do. if we think someone's higher end, we will put a programme together to guide them. It would just be, you know, in 2-3 weeks will give you a call, see how you getting on. If it needs to be tweaked, great we can work to do that. Quite a lot of things were pointed to the Stroke Association, cause it was kind of a lot of that resources on there for them to digest and read.

AS: Was there any plan that was commonly used by you and maybe your colleagues in your team, like certain maybe programme or website or like system that you use for stroke patients.

KM: There weren't necessarily a set system though, it would just be a guiding and tapping them into resources, like the stroke association, as we felt was needed, there weren't a set, and there is still isn't a working guide in place.

AS: Because I'm going to ask after that if there was any good practice, maybe an example of good practise during that time. So, what would be good practice at that time?

KM: Yeah it's really hard to say isn't it. We would like to think that we give good practice.

AS: Yeah I mean if you would think something that you have done, or maybe one of your colleagues has done and you think "this is considered as maybe a good practice", and people after stroke will get most benefit from that maybe practice or whatever?

KM: I can't think of something on the spot on the top of my head, sorry.

AS: Yeah it's alright. During that time, I think people just want to try to catch up with anything that can help their patients, because everyone losing that face-to-face communication, and they weren't able to meet their patients maybe. My next questions is about if we think of having and hopefully won't have any pandemic in the future, but if we do have any lockdown or another wave of the pandemic, so what would be a good plan for stroke rehabilitation in that time during lockdown, and maybe another wave?

KM: Yes, so I'm guessing you are asking what we've learnt from this one.

AS: Yeah.

KM: So the traffic light system works really well, cause it helped us identify what patients definitely needed with the face-to-face intervention. The telecare was okay, but it was better to actually see

someone on a screen, so we've got that up and running now, that would be something that we would be able to adopt to make things a little bit more streamlined, such as pre pandemic no one even knew what zoom was. We are all just, I'm still working quite remotely and that for some people has worked great, as in we have therapists that sit quite north of Hereford, so for them to drive coming to the office, it would take a big chunk out of their day, and then trying to get patients back up north, it was an hour and a half, so a positive from that is we've developed communication pathways more efficient through the use of zoom and team meetings. So it actually gives the therapists more time within their working day to see patients, so that’s a good thing we’ve keep. And I think for certain stuff that we're doing within the team, we've kind of said people are happy to continue to using utilise the computers as much as possible. But on the downside, especially thinking of the new qualified physios coming through, some are in their third year of placement, because of COVID, the amount of hands on contact they've had with a patient is very minimal. So that’s going to have a huge implication going forward on how quickly we going to get out to patients, the quality of the patient care, and also it's going to take us a lot more time trying to train up the band fives and give them that confidence.

AS: Are they still not allowed to see patients at the moment?

KM: They are. For example, we've just had a student shadowing and they are in their third year, and I think just cause of the move and the way that some of the universities are doing placements now, like doing corporation placements, they are coming up to their third year and they've had hardly any hands-on experience with patients in a hospital, which is frightening. Cause you think that is where you learn so much, and so but we've tried to adapt even with the students, we have tried to get them involved even if they're not in the room with patients, and get them on the zoom and stuff like that. So these things that I think we've learned along the way that will definitely keep. And to say what will happen, I don't know, I really don’t know. If there was going to be another crisis, we will just get pulled into the hospitals again.

AS: Well Kristy, what would be your suggestion for maybe managers and for policy makers and care providers, what would be your direct suggestion or message to them in this case?

KM: Just about thinking more laterally isn't it. I think there is an initial panic of “Oh my God, and we need all hands-on deck”, people were just so unsure, we were being pulled pillar to post, and then they were just depleting all the services, so it is just that knock on effect. It would just be learning from this, look at how pulling staff away actually does impact on a service rather than just thinking of the here and the now, think more long term. It's a difficult one.

AS: Well thank you Participant for that. One more question and we will stop there, it is about what can healthcare systems do for people in isolation, who are in isolation because of COVID or any other maybe health issue? if we got to deal with people in isolation, what would be the ultimate plan for these people?

KM: Do you mean if they are like it in the 10-day isolation period?

AS: Socially, maybe isolation or maybe they are staying home and are not allowed to leave because they are tested positive, or maybe they have to be isolated? Maybe a good system for them to deliver their rehabilitation services?

KM: Yeah so ultimately if someone is being discharged and you know they are COVID positive, the therapist on the ward are their main point of contact to that point. So they need to give them, “A” the reassurance that they will get followed up within the 10 days, give them a home exercise programme appropriate for their level, and as they do anyway, referring to our services and then for us guys to make that contact to say “hey, we are aware you are home, is there anything urgent that needs addressing?”, cause within that isolation period, even if its something urgent and a patient is at risk, we can still go in. So its just about figuring out “are they safe, are they capable of waiting for 10 days or do they need that input straight away?”. Well it would help if they were being discharged and therapist on the wards, if they knew they were COVID positive, giving them a bit of a prescribe programme to continue with until we could get in to tweak it. Ultimately it would just be over the telephone.

AS: Yes maybe over the telephone or any other line, meeting or software or whatever.

KM: Not that I am aware of, we don’t have any programme that are in place with I don’t know, someone with fine motor or dexterity, you know, just trying to get them to do stuff on the computer. There is nothing like that in place. Where the world is going with technology, there is nothing in place like “if you go onto this website, or if you log into this programme, you know there could be a few games to play that would target upper limb movement, or we could potentially get you doing a little bit of drawing if its upper limb”. As far as I'm aware there's nothing like that we've got access to, so that might be something to consider, I don’t know, long term, if we can't get into patients, is there any programmes that are being developed?

AS: Yeah well I have seen some studies use this kind of, it is also part of self-management to encourage people to do, you know, using some maybe softwares or something to carry them to do more of their therapy exercise or whatever home programmes. But nothing now in place, they are all just for research purposes, but hopefully in future we would have some of them, maybe physios and OTs to use them in their practice. Kristy, I'm done with my question. I just ask you if you feel that we have missed any point that you want to mention or to add about your experience with stroke rehabilitation during the pandemic? So if you feel anything that we have missed just add it please.

KM: No, it's hard isn't it when you're on the spot, no, I hope it has given you a little bit of information and it's not just a load of waffle.

AS: Thank you very much for your time and I really appreciate your participation in this study. I’ll stop recording now.

---

audio_only (1) DECEMBER 2021 TS - DEPTH - 39 MINS
[FEMALE RESPONDENT]
[Other comments:]

If you can just state your name and that you agree with the recording please for me, so we can start,

My name's Participantand I agree to the recording of this interview.

Thank you very much again for your time today. As you may be read in the information sheet, there is no wrong or right answer, we're just interested in having your perspective and your thoughts and experience with stroke rehabilitation during the pandemic. Feel free to say whatever opinion you have, we won't use your name in any report. We asked for your name just for the recording, just to go back for data analysis, so nobody will be using your name in any report. Just to remind you it is your right if you want to stop at any time, you want to take a break, you want to end the meeting; just let me know at any time. Let us just start now. Can you please tell me about the stroke care and rehabilitation services since the first wave of the pandemic?

Okay, so I work for a team that sees both stroke patients and neuro patients, so it's a neuro rehabilitation team. We work with the same manager who manages the community stroke team and the early supported discharge team for stroke. In the initial phases of lockdown, we had to put our caseload on hold to support the other teams, and we were all acting as an early supported discharge team for stroke. [Signal breaks up 0:02:04.2] we felt there was quite a bit of inequity for patients, both the stroke patients that had already been waiting for quite a while, and for the neuro patients as well. The rehabilitation and stroke hours were very limited. A lot of patients, so patients who were being discharged from hospital were getting quite a quick service if they were choosing to have it. Some were isolating, but the ones who had already been referred and were quite a while post-stroke didn't receive anything. That was a difficult situation to manage.

All of that was because of the pandemic, like they used to have maybe taken care of, but because of the pandemic you have to facilitate early discharge and maybe just be moving to live in the community because of the pandemic?

Yes. It was to free up the beds I guess, because they were expecting COVID or had COVID cases in the hospital, so the wards couldn't provide the rehab that they would normally have done.

Okay, if you just go on and tell me more about how the pandemic affected the way of service delivery and how people maybe on the acute unit were treated, or how the rehabilitation was delivered to them.

The patients that were prioritised through the early supported discharge type service, they received phone call triages usually first, whereas their first contact would normally have been face-to-face initial contact. We obviously had to screen patients to see if they had got any signs of COVID, so we would be checking before we went out.

Yes, so probably this is something new to everybody at that time, something to consider before you start dealing with any patient on a daily basis maybe, just to have them checked if they have any signs.

That's right. Yes, and we were in a position - initially we didn't do lateral flow tests, that came in later. I can't remember when that started. It was probably, I think it was last year, the end of last year we started to do lateral flow tests, but at that point we weren't in the initial phases. We had to start wearing PPE, which can be quite difficult with communication with patients.

Especially for physios.

Yes. As physios we didn't do any virtual treatment. With patients that had been waiting for a while, we did do phone reviews, just to make sure that there wasn't any immediate urgent need with them.

This is for people who are still inpatient?

No, so we're not inpatients, we're a community team.

Oh, I see, okay.

We don't do any in-reach service at all, it is just community. It's difficult to sort of keep a logical order really, isn't it? The patients who were coming out of hospital didn't really have to wait, and they would get six to eight weeks of therapy from whichever team was seeing them as part of the [unclear word 0:05:49.6] sort of service.

Was there any time where the service totally stalled because of the pandemic?

No, we continued. As long as the patient didn't have any COVID symptoms we continued, with the newly discharge patients. The patients that had been discharged previously, it was done following risk assessment and response to letters or response to phone calls.

Is there anything you want to add about your experience during that time?

Was there anything…?

Yes, like any changes in the system or maybe in the service delivery, or anything that you want to add to this part before we move to the next one?

I guess physio continued to go in, but a lot of speech therapy and psychology was done remotely. We didn't feel that was something that we could do as physiotherapists because of the risks involved. Can't think, I have sort of looked through the questions, so I think things might come up as we go through.

It's all right, no worries. If you just maybe think in particular about the way that the service is delivered remotely for these people in the community, how did you feel about that, just contacting them remotely instead of visiting them or having them maybe physically attending some of the therapy sessions?

It's not a good quality of service really, I don't feel. I think patients when they did see us were very pleased to see us and felt that we were the first people that they had perhaps seen with any sort of medical health professional background. [Unclear word 0:08:00.0] people, they've got to be a good historian or have good speech to be able to explain what their difficulties might be. In terms of remote therapy, not everybody has got access to internet.

This is going to be one of the challenges, yes, especially people, maybe like with older people. I think not everyone will have access to the internet or maybe to the resources.

Birmingham is not, what's the word? There are areas of social deprivation where people just haven't got the money to pay for that sort of equipment or the internet service. It seemed a bit of a two-tier service really. If people could afford to buy the equipment and have the internet, they got perhaps a better service than others.

All right, so let us move to other parties, people with stroke or patients or whatever you want to call them, stroke survivors. What do you think were the problems of people who had stroke at that time during the pandemic, in terms of their physical rehabilitation?

A lot of the patients who were referred to us from the wards, they hadn't been able to have a normal handover from the therapist to the carers to support them. Patients' equipment wasn't always readily available. There was a big issue with getting stair rails fitted for some patients, so there was more risk of falls or people being confined to certain areas of their home. Sorry, could you repeat the question again? I've sort of lost my thread a little.

Yes, a general question about the problems and the needs of stroke survivors who have had their stroke during the pandemic.

A lot of them didn't get very much of an inpatient stay. The patients who were more dependent perhaps moved from a hoist to a stand aid of some sort, or might have gotten more mobile, weren't given that opportunity. Then they came to the community services and we were again on a shortened length of treatment. Somebody might have had only two weeks in hospital when previously they might have had four or six weeks, and then they came to community services and were only offered six weeks when maybe they would have had 12 weeks before. The patients who were mobile, I don't think it was such a problem for, but I think the patients who were more dependent just didn't get the opportunities really to have the amount of physiotherapy or other therapies that they really needed. I think patients were generally quite understanding about the fact that we were limited in what we could provide, but some of them were quite frustrated and quite upset and quite angry, so sometimes it meant that...

It's not an easy situation for them. Also the emotional and psychological impact of stroke make it even worse maybe, even if they don't need a lot of therapy after their stroke, but still not having therapists who follow with them and not visiting the rehabilitation centre will make them maybe discouraged about their cases.

Yes, the patients who perhaps normally we would have brought in for therapy into the gym didn't have access to that either. If they hadn't got the right equipment at home, we couldn't provide the rehab by bringing them in to use the equipment in the hospital. I think a lot of families and carers were quite distressed, because they hadn't seen the patient who had had a stroke since they went into hospital, so they left their home having just had an acute event and were previously mobile and independent, and then they weren't getting any contact really for weeks or months, and then there was this person coming home to them who was completely different and were maybe quite dependent. They didn't really have that preparation for having the person discharged home.

In terms of mobility, because our study has some interest in mobility in particular, so do you think most people, let's say in general people after stroke during that time who were discharged fairly early and they maybe weren't able to catch up with their rehabilitation on time, so do you think that maybe affected their recovery for mobility long-term?

I'm fairly sure it would have done, yes, because once our service stopped, there wasn't anything else to progress them further. A lot of them would have had more rehab potential when we had to discharge them, so instead of getting guidance, it would be very much self-management, guiding them to appropriate websites maybe, or giving them home exercise programmes. Some patients can cope with that and some can't, and it's not easy for them to self-manage.

It's good, you started to the next step in the interview, about the strategies for self-management. What were the strategies, and let me first ask you as a therapist? What were your strategies that you tried to manage, even on a personal level? Because it came all of a sudden, the pandemic and the restriction of services and all these things. Were there any strategies that you or your colleagues had used at that time to manage in some way, or maybe to take care of the patients in some way?

We prepared some resources that we could take out to the patients quite early on, so things like home exercise programmes. I put a pack together of sort of frequently used exercises, so that on the first visit I would be able to give them something that they could hopefully get on with. Whereas before, we would see the patient first and then design the exercise programme specifically for that patient. That worked quite well. At times we would have sheets and perhaps have to cross things out, and perhaps didn't look

as good and it wasn't a bespoke programme, but at least they had something that they could carry on with. Quite often it might be quite a while before we could go out to do a second or third visit, and so if they've got something from the beginning…

Sorry if I interrupt you; you mean a visit, you were doing a physical visit, or maybe online or by phone or whatever?

A physical visit, yes. I think we felt we needed to see them face-to-face rather than trying to provide something from just a phone conversation.

Even during the lockdown you were visiting them physically?

Yes.

This is good actually, because at that time I think no one would blame you maybe if you can't visit them physically at that time. I don't know the system, but I'm just generally speaking. All the services were stopped during the lockdown. Let us move, if you have any example of you working with any patient, or maybe any of your colleagues who have had maybe a good example of managing at that time with maybe using online services and resources, or maybe delivering rehabilitation in other ways, other than a formal visit.

For some patients, we did a bit of research as a group of physios. There were three physios working in our department, and we had a look at some online resources and tried to critique them, to find which ones we were happy with as therapists to give to patients. We developed a bit of a list that we could hand out to the patient. We always saw the patient first; it might be that that took the place of a second or third visit, or just gave the patient something to do in between visits, because we weren't able to deliver intensive therapy. I think for us as therapists, we continued to go out throughout the pandemic, so we felt that the safest thing for the patient in terms of risk was to see them face-to-face. I know other services across the Midlands stopped completely and that people had very different responses, but we did continue to go in.

Yes, compiling apps or online resources through the Stroke Association or through Different Strokes, so we all would look at certain apps or certain websites, critique them and discuss them and share them between us, and we sort of made a shortlist really of the ones that we felt were useful. In

terms of anything else, we made sure we triaged the patients after we had seen them the first time. We triaged them before we went out and sort of prioritised patients using a RAG rating scale, and then we would do that after the first appointment as well, so that we made sure we were targeting the patients who were at most risk. We as a team started to use our work phones more, so we would phone patients and text patients information if that was useful. In terms of the team for lone working, that was quite useful as well, to have the phones and we used a WhatsApp group so that we could all check on each other. Some people were working from home at that point as well, so not everybody was coming into the office.

If we just think on patient level about how maybe you helped them to self-manage their conditions at home, and maybe to use some of the self-management strategies, if you can just tell us about self-management, how you introduced that to patients, and maybe facilitate their using of self-management strategies.

As a team we always do self-management anyway. That's an important part of rehabilitation, to be able to self-manage.

Yes, but maybe during the pandemic it became even more essential to use that because maybe you weren't able to visit them every time, and as you said, you maybe describe them from the first visit instead of having it on the second or third session.

We used Physio Tools where people have got checklists that they can tick it off, and they can also use that on their phone. We would ask people to fill in tick sheets of when they've done their exercises, but again, that's something we've always done. We just tried to put everything in a folder as well so that they know where to find things, because quite often those exercise programmes can end up scattered all over the place. We would on our visits review them and progress them so that they've still got something to be getting on with.

Were they involved in maybe setting goals for their rehabilitation and maybe choosing their therapy component, like the type of exercise and the intensity of their exercise? Were they involved in setting these things?

I suppose not as formally as we would normally have done. Normally we would have interdisciplinary goal setting meetings, but we weren't working in a way where it was easy to do that really. The goals would be established at the beginning. We would encourage people to, if we can get them to mobilise with family members and things like that, that's something that we would encourage. That would be after the initial visit, so we would do a handover first and perhaps provide some information for the carer. Not everybody is able to self-manage, some people need support. We did quite a bit of work in care homes, taking photographs of patients doing exercises so that it was obvious to carers that the patient could actually do the exercise. With diagrams and instructions, you don't necessarily see that the patient can actually do it, so we did a lot of photographing the exercise programmes.

Was there patient education session for people when they first discharged to the community from hospital? Was there anything about teaching them how to maybe mobilise safely or to walk safely at home, to maybe do some therapy exercise at home safely?

We would do that in the patient's home, we wouldn't do that remotely or on the phone.

In the pandemic you still delivered this thing to patients?

Yes, we would still do that. That's something that hasn't changed during the pandemic.

This is very great, yes.

Obviously we do our risk assessments first and make sure that the carer or person supporting the patient is able to do what we're asking them to do. Yes, carers played an important part really in supporting rehab.

Was there any focus again on mobility, or this is like general speaking about rehabilitation, like for upper limb, lower limb, everything; or there was something maybe in particular about mobility and lower limb rehabilitation, if you want to tell us about that, using self-management to facilitate that?

I guess because we were still going in, it was the same as we would normally do. There wasn't anything different in the pandemic really. One of the main things we would do is people would have exercises

maybe in laying or in sitting, but then if we can get them up and walking and it's safe to do so, then we would encourage them to do that, with the carer supporting if necessary. We would try and get patients, tell them how often we want to do them, how many to do, what the priorities might be for each of the exercises, tell them what the relevance is in terms of whatever the goals are. Obviously if your goal is to walk and it's safe for you to stand and walk, then that is something that would be a main part of the rehabilitation. I'm sorry if I'm not quite understanding your question.

No, I wanted just to see if there is anything more than the normal practice that you are doing in terms of mobility or anything else. You said that your team was trying to manage everything as normal during the pandemic, and this is actually very great effort from you and your team. Let us move to think now when things are coming back to normal after the pandemic. As a service now, what would you see as priorities for stroke rehabilitation now after we came back to normal?

I think to get therapy in a more responsive way, so not have a waiting list. At the moment our waiting list has grown again, so during the pandemic people were maybe isolating and perhaps didn't want treatment, and there was a reduced amount of referrals. I don't think people were presenting to the GP or to the hospital because they were worried about COVID. Since the beginning of the year, referrals to the team have steadily increased and we now have quite a long waiting list, which at the moment is about four months, unfortunately.

There have been a lot of staff shortages. Recruitment is really difficult, so it means that we've got these patients waiting who desperately need to get back to mobility, maybe to be able to do the stairs so that they can access the community, people that live in flats. It's having more of an impact now, I would say, than during the pandemic. During the pandemic we managed, and now we're not managing. We can't get staff, and just so many more referrals, so I think that's the main priority that needs sorting out. People need the rehab straightaway, they need the physio straightaway. If people are sitting on waiting lists, they might get secondary problems, they might be more likely to fall, they might be more likely to get contractures.

Recurrent stroke maybe.

Impacts on mood. There's just so many things. Without the staffing there's nothing we can do about it.

We want to explore what could be done just if we have another pandemic. Hopefully we won't see any other pandemic, but let us think if we have any other lockdown or any other pandemic, what would be a good plan to work during a lockdown or another pandemic, as a physiotherapist working with stroke survivors?

I think the assessment in the beginning is key, the triaging is important. Obviously we would still continue to identify who wants to isolate and who wants to have therapy at home. For those that we can't provide therapy for at home, then I think that's still very useful. I think maybe depending on the volume of patients that we're presented with, perhaps not working as intensively so that we can spread ourselves more thinly, so we see more patients but less frequently, so that everybody's got some strategies. I think we would use the apps and the home exercise programmes like we have before. Phone reviews I think can be helpful, but not ideal for physios. I think we are more prepared, we've got more options now than we did have, but I think we need to think about how we manage the whole caseload.

I want to also think about people who are self-isolating maybe at home, if anyone has maybe some symptoms or positively tested for COVID or any other pandemic. What could be a good plan for them?

Something I haven't mentioned, when patients are referred to us, if we get contacted by the referrer, we encourage them to provide home exercises and to provide some advice about management, because they've actually seen the patient face-to-face. They're better placed than us in the community when we've never met the patient, so that's something that I think is quite important, if they're prepared for discharge really. I think it probably would be possible by phone to establish some needs and maybe send out some generic exercises for them to do. One of the services that we work fairly closely with has done virtual groups of therapy with patients, because they weren't able to see patients at home because they're a centre-based service. We could maybe look at something like that in the future.

It's just managing the risk that I find quite difficult. I think maybe upper limb exercises if people are sitting down are quite risk-free. We would have to be careful about risk assessing if we were going to do any sort of standing or walking type exercises. We could probably do some chair-based exercises fairly risk-free, but the limitations of that are that some patients won't have the equipment, as I was saying at the beginning, so then it would be a matter of just sending out home exercise programmes to those that are self-isolating based on what we know about the patient.

I'm done with my questions here, it's just for you now, I'm giving you the mic. If you want to add anything about your experience during that time, or if you want to add anything as a suggestion for future maybe plan if we have any other pandemic situation.

I know lots of people across the country have tried doing more virtual appointments with physiotherapy, so it would be good to learn from those. Certainly in the north of England there have been some things going on; I've got a friend who works in the north of England. It was a very stressful time for everybody really over the pandemic, for us as therapists as well as the patients.

If you just tell us what other people have done in north England, like what they have done as something new. How did they manage?

I don't know a lot of detail about it.

Just in general.

They've been running some virtual groups I'm aware of. I think that was more for upper limb rehab, so I don't know an awful lot of detail. I think it's something that needs to be shared really across the country. I'm a member of ACPIN and I haven't seen much come up on there. It would be good if people share their experiences and what they found worked really, so that we can all gain from it. I think we've been so busy trying to manage the caseload, there hasn't been an awful lot of time to reflect and to look at alternatives [over speaking 0:33:43.2].

Or to share the experiences, yes. I think a lot of studies are going on now just collecting people's experiences and how they managed that time. I think a lot of studies and sharing of experiences will come in the future.

I think it's only a certain group of patients that that can target really. I think also, people discharging the patients as well, if they can prepare them more and that becomes part of their routine. We can't provide in-reach unfortunately, but just have that sort of link between the inpatients and the community would be quite helpful. I can't think of anything else. We have also been sending out advice sheets, so not just home exercises but things like about fatigue management. We try and make the referrals early on if we can for spasticity management and those kind of things. Unfortunately in the pandemic there was no

access to those services, so I think in the future some of the services that were closed down and people were taken from to support other services, I think that needs to be reviewed. Things like the [unclear word 0:35:11.3] clinics, the spasticity clinics, they just stopped completely and that had quite an impact on what we could do in the community, because people's spasticity wasn't being managed effectively.

Is there anything you want to add?

I'm just trying to look at some of the notes I made before. The Stroke Association we linked up with, but again, they weren't going in to see patients either, that was very much remote therapy or phone call advice. I think we have to use all of the resources that we've got, so linking in with the Stroke Association. If the health service could provide maybe tablets or support the patients to access remote therapy, that would be useful as well. A lot of schools provided equipment to students so that they could study from home, so it would be good if that was taken across to people with health problems as well. I don't think I've got anything else to add, I can't think of anything else.

This is very interesting, Participant, I really enjoyed the interview. Thank you very much for your time, and thank you again for giving us the opportunity for doing this interview. You also have my email if you want to add anything that you think we missed out in this interview. Just feel free to email me to add anything, I would appreciate that, thank you very much.

[END OF TRANSCRIPT]

Participant's interview FEBRUARY 2022 JTW - DEPTH - 41 MINS
[FEMALE RESPONDENT]
[Other comments:]

[Moderator explains market research/respondent gives consent]

[Transcript begins at 00:02:35]

Participant, can you please tell us about stroke care and the rehabilitation delivery services since the first wave of the pandemic at your local practice?

Okay, so I work in outpatients at XXX Hospital, dealing with patients that have generally been discharged home, and it's kind of at the end of the rehabilitation, if you like. Their physical rehab. They have a service that goes in to see them once they're discharged, and then if there are further goals then we go and see them afterwards. Saying that, during the pandemic I was relocated to the acute stroke unit at New Cross, and also to the rehab ward at West Park, so I've been around and about quite a bit, to be fair.

Does that mean at that time you moved from no community services delivered at that time?

It was very sparse. I remember the first pandemic I was off poorly right at the beginning, and I was in contact with my manager, but there was no, it was completely shut down, our service. There was no outpatients whatsoever, so we were all redeployed. Then it was, I can't remember timescales, but we were redeployed to wards. Then it was slowly brought back. I think I came back to outpatients but then I had to go again. It was like coming and going quite a lot.

I think that was dependent on the cases, and the rise in admission and [over speaking 00:04:52]…

Yes, definitely, and staff sickness and all sorts as well.

What services did the NHS or other community services provide to people after stroke at that time?

Sorry, can you say that again?

What services were delivered to people in the community during inpatient at that time?

Well, we were delivering occupational therapy to inpatients, and as I say I was over on the stroke unit. Also when we were on the ward, we were seeing patients there. I think there was very limited service in the community for quite a while, to be fair. I don't know, because I wasn't working in the community, I can't really comment on whether the other teams like ESD were going out very much.

That's fine, if you just please talk about the situation, like the context where you were deployed to, so what were the case.

I think the worst thing for the patients was the lack of visiting. They were just so isolated, and that had a massive effect. It had an effect on us as well as therapists, because quite often when you're in the ward situation, you often want the relatives to see the patients, see what they're doing, and then you can ask them, and you can involve them in the therapy, and get some feedback as well, so there was none of that happening, which was really frustrating. The fact that patients couldn't have any visitors, that was awful for them.

Participant, we will come later in the interview, because we have order for the questions, so we'll come later for this aspect.

Okay.

Now, if you just a little bit tell us about the services or what rehabilitation you were delivering at that time for inpatients.

On the acute stroke unit it's as and if the patients can tolerate any therapy, so a lot depends on how poorly the patients were with their stroke, whether or not they'd got - they were COVID-positive or not. We could quite often still get patients, it just depended on what sort of location they were in, they would have a clean, maybe a non-infected bay, or patients that were infected or they'd have a contact bay. We'd do our normal therapy if possible, assessing patients, doing the full neurological assessment. Depending on where they were medically we could still get them up sitting on the edge of the bed or sitting in a chair. It was difficult with taking patients to the kitchen and doing OT stuff like that, that was a little bit more difficult to start with.

How about the duration of inpatient, was there any time where you was to just discharge your patients from inpatient unit, or you think that was okay as usual?

Yes, I think the idea was they'd be safer not being in hospital.

Yes, so you have experienced that?

Hmm [affirmative]. Yes.

Thank you Participant for this. Let us move to another aspect. What modified systems were in place at your workplace for people after stroke, if you would say?

What, sorry, can you say that again?

Yes, what modified system was there?

Modified system?

Yes. Like something was modified because of having COVID situation.

Yes, the amount of staff, because obviously some staff were at risk, so we didn't have as many staff, that was definitely modified in the number of staff because some were obviously off shielding. The PPE, obviously, and different types of mask use, that was quite a difference as well, and the locations of taking the patients.

How about the way of communication with patients, and maybe touching them or maybe working with them at the same room, or was there any modified system with that?

It depended on where the patients were, and if they were in a side room then we couldn't take them out of the side room, so we had to do what therapy we could within that confined space.

Was there any effort to use remote delivery of rehabilitation therapy, like providing something online to people after they discharged?

Yes, but that was in community therapy-wise. On the ward patients were using Zoom to speak to their relatives, but not really very much remote stuff on the ward, but we certainly did that in outpatients.

Thank you Participant for this, so let us move to another aspect of rehabilitation, which is the impact of COVID on patients and their needs during rehabilitation post-stroke. What do you think were the problems for people who had stroke during the pandemic time?

Do you mean the patients that had a stroke and COVID, or just having a stroke during the pandemic?

We focus on more of having stroke during the pandemic, and how that…

Not necessarily having COVID themselves, just the stroke?

Yes, like what were the problems in accessing rehabilitation services?

I think a lot of the time, well, first of all there were no rehab services. Quite often clinics were closed, and the whole service was put on hold. We're talking community now. Quite often patients were scared to come in as well, so a lot of patients stayed away.

At that time it was a difficult time for everybody, so people, they are not sure what they are going through, so everyone was worried at that time.

Yes. I think patients on the ward, sometimes when they came into the ward and they didn't have COVID but then they did get the infection, then they were really confused and quite devastated.

Participant, what do you think is the impact of the pandemic on rehabilitation services for these people?

For us it's on-going. Patients, they've just become so isolated. Patients with a stroke who are discharged home, it's a massive change in their life anyway, and in normal situations patients get very, very depressed once they get home, realisation is that their life's changed, but with COVID and they couldn't go out and they couldn't see anybody, it really had a massive impact. Also the facilities just weren't there. The amount of groups and support that we used to give patients, we couldn't do that, and patients were very isolated, very bored, very depressed, very scared. Physically they went down as well, downhill, because we offered so many physical activities as well, with all the groups that we offered. That was a massive blow.

How do you think that will impact their future life? How do you think this situation will impact on their rehabilitation?

I think you've got patients that are still, especially with the different variants that we get, you've got patients that will not go out. They're going to deteriorate possibly, physically, mentally. I can't remember the original question now, sorry.

You said people were isolated and they went down with their physical and maybe even mental status, so I'm just asking what do you think the impact of this situation on people's life, future life?

They're just not going to have the motivation or the ability to go forward.

How do you think for example this might affect them in terms of mobility and recovery in long-term?

We have seen patients that because they're not doing as much mobility, they're just poddling [sic] round the house, and they're sitting for longer, so their muscles are not being so active, they're not getting fresh air, they're not getting that social interaction with people. They're not moving enough, and then mood gets low, and then they don't want to do anything. It's a bit of a vicious circle, really. Also they struggle to get in touch with the GP, they struggle to have an appointment with the GP. They're just out on a limb, a lot of them. A lot of our patients have contractures, they are susceptible to contractures, and they have to do a lot of exercises for their upper limb, and without… We used to do upper limb groups and also stretch classes. We are offering a stretch class but it's online. I saw a patient today who, he can't do online stuff, it's beyond him, and they haven't got very good internet connection at home, so he was in a bit of a state. He really was so tight in his arm, and it has had a massive effect on him.

This is so sad.

Yes.

You wish you have done anything for these people at that time.

I know, yes.

It is what it is. Participant, from a therapist perspective, please can you tell me about patients' need during this pandemic in term of rehabilitation?

Their needs?

Yes, their needs.

They need communication. Are you talking physical needs, or?

Rehabilitation needs in general.

Okay, so we're looking at, like I've just said, upper limb therapy. They need education. They need assessments, advice, they need constant stretching exercises, they need splinting quite often. They need to attend appropriate clinics and classes to make sure that they're doing the correct exercises and they're not getting any problems. Quite often you have patients with cognitive issues as well. They can be struggling with issues at home, and they can't… Quite often cognitive work is like using the environment, like going out and being able to shop, but then patients are having to do it online. That's taking away the actual physical aspect of doing the therapy. We haven't been able to go and take patients to shops, which can be an important part of their therapy. There's loads.

Do you want to add anything about patients' needs during that time?

Quite often they need some social interaction, and that's the massive, massive thing that they can't get.

Yes, it makes a lot of difference for these people.

Yes. Like I've got a patient that has been just so, so depressed, and then I managed to get her on to one of our online groups, and she absolutely loved it, it just made her day. If she could actually come into the hospital and do the group physically, it would be so… You don't realise what we offered until it's gone, we just didn't realise.

When you lose something, you will know the value of it.

Yes, absolutely. We used to do groups in the hospital here, and we used to do groups in the community. Gym groups for example, so patients aren't getting that physical exercise.

When was that group, during the pandemic, or after?

No, this was before the pandemic, and we had to stop during the pandemic. Now, there are some groups that are opening up, but we just have to signpost patients to the groups rather than us starting the groups ourselves.

Do you want to add anything about that, or we should move to next aspect?

Okay, let's move.

We'll be moving to talk about management strategies, and if you please tell me what were the strategies used to manage access to the services at that time.

Like I say, we couldn't offer the actual physical groups and therapies, so it was a lot of online assessments, online classes. There was a time when we thought we could start a group, but then another variant came along. What we're having to do for example is if patients need some upper limb work, then we're having to do it one-to-one rather than the group situation that we had before, which takes more time, but also the patients used to enjoy being in a group with patients in a similar situation. Another thing that's been affected I think as well is the ability for us to do much training within the teams, because of the staff shortages. That's been affected. We have had to change things which in some ways are quite good.

Can you give examples of that?

I say it's good, it's not as good as it could be. We have taken to telephoning patients for an initial assessment rather than seeing them, because we can get quite a lot of information and it's a lot quicker

so we can get through a lot more. Also having meetings online with different teams, for example a regular morning once a week interaction with the rehab team that will eventually refer on to us.

Were there any other strategies that you used to deliver rehabilitation for patients?

I'm just trying to think on the ward situation now, because I've been working in all different areas.

If you think of any good examples of good practice that you used, you said you used a stretch exercise, you use maybe something online or by telephone for assessment or reaching people.

Yes.

If you could think of any examples of good practice that you used?

Yes, we do the stretch class and that's working online, we do that weekly, which is really quite good. Especially for younger people, they can get on with it quite well, and we can see a lot more people doing it online. It does take two people though, one to go through stuff and one to demonstrate, but that's okay. We also do the games group online as well, and luckily we still have some volunteers that help with that, as I say, online, so that gives patients quite a lot of chance to - they just have a bit of a giggle, really.

Participant, I have a specific question about strategies for self-management, so was there any efforts to introduce self-management programmes to any of your patients?

We've always done that, really.

During the pandemic?

Yes, I mean, we have. During assessment, then we would give them as much information as we could over the phone, we would send information out to them in the post. We did online… Sorry, not online. We got a volunteer who helped with the art group, so she took, or we took packages round to patients so that they could do it by themselves. A lot of our therapy is teaching the patient to do it themselves anyway.

Was anything other than maybe teaching patients or providing them with some information, like did they get involved in maybe learning their therapy plans, or maybe in goal setting, or maybe in self-monitoring or any other thing?

I'm not really sure.

If you remember where any specific effort taken to improve mobility with self-management?

Mobility?

Yes, like did you use self-management to improve mobility for patients at that time?

Not really, because our mobility practice is, from an OT point of view, we would accompany patients, especially if it was outdoor mobility, we'd want to accompany them and do a proper assessment. If we'd already done that, then we would involve relatives and ask them to supervise and assist the patient. I'm just trying to think, if relatives were available, then that's fine, and we've always involved carers as well.

Did you see any patients benefit from self-management that you have provided?

Yes, they have benefited. It's been better than nothing, especially with the upper limb group, patients have gotten on well with that, and been able to maintain their upper limb.

Participant, thank you for this information, so let us move to the last aspect of this interview, which is about looking forward when the health services start to reopen again as normal after the pandemic problem. What do you consider as priorities for stroke services?

Priorities from generally, I think from the inpatients I think the priority would be we've got to monitor and make sure infection rates, but I think some kind of visiting would be a priority for patients, and being able to interact with patients' relatives for therapy staff. For outpatients, I think if we can get the resources back, the community resources that we provided, both physical and stimulation-wise, some of the groups and our group sessions of therapy, I think that would be amazing.

Let us think, what can healthcare system do to ensure optimal services if we have future wave of pandemic? Like what would be optimal services plan in delivering care and the rehabilitation?

I'm really sorry, the phone was ringing then, can you just repeat that? I'll have to leave it to go to answer machine.

Okay, it's all right. I was thinking, let us think, if we have another wave in future, and hopefully we won't, but let us think that way, if we have any future wave, what would be a good plan for healthcare system to deliver care and the rehabilitation?

I think having enough staff to cover staff shortages, but that's something that's quite impossible, really.

Think about also maybe modifying some of the system, maybe the way of delivery, maybe remote delivery of services?

Yes, I think we're doing as much as we can regarding that, to be fair. That is a way forward, pandemic or not.

Do you think that would be enough, if we have a lockdown or something?

No. Patients need one-to-one, they need to go out and socialise. Not everything can be done online. Not in OT.

Also I want you also to think about people who are in isolation, what would be a good plan to deliver rehabilitation for them?

If they're in isolation did you say?

Yes, like if they have to isolate, and how we can deliver rehabilitation for them.

Again that's got to be done remotely, hasn't it?

I don't know, it depends on local context, so in some cases I think yes. I don't know, it depends on measurements at your context, I'm asking.

I guess education of family, that would be quite a way forward. Getting the family to… I mean, we do that anyway, but it would be even more of a…

Even though if somebody gets isolated, I think they will be isolated even from their carer and family members, so this wouldn't be maybe an ideal option for them? How about maybe online resources and remote communication with those people?

Yes, well like I say, that would have to be the way to go. We also get lots of patients in care homes, and that's been a massive issue. That was an issue before the pandemic regarding staffing and education. It's an on-going uphill battle.

[Thank you and wrap up]

[END OF TRANSCRIPT]

DECEMBER 2021 - TS - DEPTH - 39 MINS
[FEMALE RESPONDENT]
[Other comments:]

We are recording now so first of all thank you for taking your time and wanting to participate once again in our research study. So, before we start the questions, could you state your name and also mention that you are happy with us recording this session?

Yeah so my name is Participantand i'm happy to go ahead with the session, and for it to be recorded.

Okay amazing all right, I just to remind you a little bit about this study, the purpose of this study is to gain therapists' experiences on stroke rehab during the pandemic. So there is no right or wrong answer, so please feel free to express anything as we are interested in your thoughts and your experience. Your name in our report, reminder again, will be anonymised. And if you need a break, or you want to stop at any time, please just let us know when you want to. And finally, do you have any other questions for us, before we start?

No questions.

Alright, so we're going to split this interview into a few sections, so the first section will come under experiences of delivering services. So, can you please tell me about stroke care and rehabilitation delivery services, since the first wave of the pandemic?

Erm, do you mean like just what my job or what?

Yeah like, sort of like, stroke care and like how you deliver your service during the first wave of the pandemic, yeah?

Okay, and so my job roles as an occupational therapist, and I worked during the first wave and in like the acute stroke setting in a hospital. It was like a 29-bed unit and you had like the hyper acute side and then just the acute side. And so on the hyper acute side you'd have people who were in like their first few hours, and since like a concern diagnosis of a stroke. And then, after sort of that after like a 24-hour period they then just be moved into the general sort of acute stroke area. And reason being because the first 24 hours of having a stroke is really crucial period where you need a lot of monitoring and because people can unfortunately get really poorly, and there's also treatments that are very sort of time-specific and they can only happen within sort of a certain period within that 24 hour. And it's also crucial as a therapist that we really attend to, we call it, I think that's what I said, we call it HASU and it's a hyper acute side and because we aim to complete our assessments within 24 hours of that person being admitted for a stroke, really so that we have a baseline of what we're dealing with. And sometimes, so examples would be like sometimes we'll do that assessment within 24 hours, and then we go back the next day and they're different, they're worse.

Was this during the first wave of the pandemic how delivery services we're like?

So yes, I can move on to that, thank you. Um so, it was interesting really. During the first wave, a very noticeable trend we found was that, if people weren't having like a severe stroke, that went with them sort of like unconscious or very obviously needing medical input, because you can have people who have strokes where they just like experience maybe some tingling down one side of their body, or quite subtle changes. Because of the pandemic and the very sort of like, we were all told to stay at home and not to go out and people were really scared about coming into hospital. I had lots of people saying like “I didn't want to come in”. So the knock-on effect is that we were having people come into hospital who were actually like four days post stroke, and that was like a really interesting trend that we were finding. So unfortunately, their recovery wasn't as good as it could have been, because they were quitting off coming to hospital because they didn't want to come into the very place by the virus was.

Yeah, this is actually the next section of our questions, we'll dive deeper in later, but we just like specifically wanted to ask about, like sort of the delivery services that you provide. So, you mentioned about like hyper acute patients and other sorts of patients. So, what kind of delivery services were given to these people in terms of stroke care and rehabilitation?

So we would do like a neuro assessment within the first 24-hour period, so we did look at, is it helpful for me to describe the neuro assessment?

Um I think neuro assessment just briefly yeah.

Yeah, so we just we just assess like their cognition, “were they orientated?”, and then like their physical capabilities and write up the assessment from that. What I would say is that all our service, what were the words were you using, service provision?

Stroke care and rehabilitation delivery service.

Delivery service. So they never really changed, they always stayed the same despite the fact that we were in a pandemic, purely because strokes don't change. And I would say, maybe things changed within sort of therapy, as in like we had people off sick and we were very short of staff. So I guess our service delivery. I was just trying to guess like come up with things, I guess like we split ourselves, I don't know this is helpful, so we split ourselves into teams, and so we had people who would see people who hadn’t had COVID and we had people who would see people who did have COVID. So that was that changed, our service delivery in like that sense did change.

Okay, so like sort of what services did the NHS or other community services provide to the people after stroke?

Um yes, so we had a, and this has been across all of my experience actually because i've worked at other places as well. Um so we have like an early supported discharge team. So once someone gets discharged out of hospital, they’ll then be seen by the early supported discharge team, who are the Community stroke therapists, and they would aim to see people within a time frame of about 72 hours. Um again that was affected by the pandemic. Normally they'd see someone within like 24 hours but they were struggling as well just from a staffing point of view.

Alright, in terms of services could you like describe a little bit more about what sort of services was provided, or it's mainly just meeting them within 72 hours is it?

Yes, you'd see them within 72 hours, and we would sort of set sort of the goals, and so, whether it was a case of they were going home and um i'm speaking from example really, and you just wanted to make sure that they would be able to function at home. So they'd complete, so all of the assessments that we do in hospital, so washing and dressing, being able to walk, being able to go into the kitchen and make yourself a hot drink or a hot meal, all those kind of things we can't replicate their own personal home environment. So, then the therapists who are in the Community would go into the actual person's home environment and actually ensure that they were able to do it within their home environment. Yeah, like we can never fully replicate it, we can do as much as we can, but we can never fully replicate it. And we take precautions in the sense that we encourage family when someone's been discharged and to be in that person's home environment for at least 48 hours post discharged from hospital, that's one of the precautions we take, because it's never a 100% that we would ever know what was going to happen at home, but that's why the fact that the team are there to sort of see them in their own home environment. The other side is rehab at home, so it wasn't a quick assessment, we'd be looking at rehabbing them at home. So if they've lost function and when they initially came in down, like the left side of their body, but they were able to then gain function in their leg, but their arm wasn’t as good on the left hand side, they will then continue the rehab that we'd started in hospital at home, and they tailor that program to whatever it needed to be for like however long that patient needed, maybe like six weeks or something like that, so that's the other side, you can do quick assessments or you can do much longer ones.

Yeah, so my next question is like what modified systems were in place at your workplace for people with stroke. As you mentioned just now about the services that you guys provide already, were there sort of any modifications, with the systems that were in place?

Um because of the pandemic?

Yeah.

I would just say that the only real modifications we had were PPE, so personal protective equipment. We, the therapist, sort of in the hospital and then in the Community, we were obviously having to source that and use that appropriately based on whether the person had COVID or didn't have COVID. So that was sometimes slowing down, certainly the Community side they were struggling to get like the PPE, so that was like a modification that they had to do. And then as well, like not necessarily placing the same expectations of recovery on someone who had COVID compared to someone who didn't have COVID, in a sense that fatigue is massive with COVID. So you'd have to sort of grade whatever you were doing with them and potentially scale it back a little bit more than you wouldn't necessarily do with that person if they didn't have COVID because of the fatigue essentially, so yeah.

Alright, in terms of like sort of like modified systems in the workplace. So you mention about PPE, were there any more other things that were modified in your workplace or?

Only the ones I've mentioned. Like there was some therapists who had their own personal health needs wouldn't see COVID patients. And then the ones who, because we, I mean actually, this won't be appropriate, because it was never in the first wave, so I won't even mention what I was about to mention because we're talking about this over the first wave.

This is over the entire pandemic actually, so it also works.

Okay, yeah I'll mentioned it. So probably, maybe like four months in my experience into the pandemic, like personal risk assessments were introduced and you were sort of scored based on a criteria. So like whether you had any preexisting health conditions, those and your age, things like that. And if you scored into like a certain number of points, then you were like classed as sort of like a high-risk category, so depending on how high risk we were, you're basically told you can't see COVID patients, even with PPE. So then we had to modify our team structure into people based on their risk assessments. So my personal experience was that I was considered low risk, so I saw COVID patients all the time, essentially, because I wasn't considered to be at risk as much. So yeah, that was a modification that we did within our teams, it was pretty much half and half actually, and we just split ourselves and you try and stay, I mean the terms we use with not official terms, like we didn't use official times, we called ourselves clean and dirty. Somewhere else were known as hot and cold, I think it very much depends where you work. So I would stay clean as long as possible and then just get dirty.

So I was just asking in terms of like, what about like the people who are sort of moderate risk of like COVID, did they have any sort of like modifications in their workplace?

So, modifications would range from 'being able to stay in the ward environment wearing like PPE that was like appropriate to see anyone', so that's your mask, your visor, gloves and a gown. So it range from that, and just not seeing any COVID patients. I think the highest modification was being nonclinical. So you wouldn't even ever enter ward environment and you could work from home if it was appropriate or you'd work and sort of, I mean the most high risk of therapist would only, they would work from home, and then the ones right below that would work sort of in the offices but weren't used by therapists who are up on the wards, because obviously you've got that risk of transmission because there was sort of a school of thought, and I don't know if it's still the same, that you can transmit COVID on your clothes. During the first wave of the pandemic, people were really conscious of that, so that's another thing I'll mentioned actually, but on this train of thought, so those therapists would only work in sort of the admin offices, because they were with admin colleagues who would never go on to the wards. But another

modification that we did as well, is that we would all wear fresh uniforms daily. We would never wear the same uniform twice and we were washing all of our uniforms on like 60 degrees to wash out any bugs and germs, because that was a real school of thought that you could actually transmit COVID on your clothing. We'd never take our shoes home, would wipe down our shoes that type of thing.

Okay yeah, thank you for that um. So the next section we're going into is the impact and needs of patients with you slightly touch upon just now. So, what do you think were the problems for people who had a stroke during the pandemic in accessing care?

Sometimes they were so poorly with the COVID that it was difficult to get like an accurate baseline of what they were like in terms of the stroke from the fatigue and point of view, I guess. And yeah when it came to sort of rehabbing them on the ward when they still needed to be in hospital, a lot of the times COVID was really taken out of them if they were really poorly with the COVID, so their rehab journey wasn't like typical of them, like what we were expecting and what we were predicting because of the fatigue, because of the tiredness, because of the high temperature and the cough. I would also say as well, and we had to be careful with where we would treat people who'd had a stroke, who’d have COVID, and people who had a stroke and hadn’t have COVID because we didn't want to obviously mix the two do you? And so we had difficulties with having enough treatment spaces essentially that were either for COVID or for non-COVID. So, one of the difficulties we found was that we initially decided that we would see all the non-COVID patients in the morning and if they needed to like use our gym space, we'd see them in the morning. And then we'd start taking all of the COVID patients into that space in the afternoon, and we thought like that's going to work. But what we actually found is that the COVID patients weren't as good in the afternoon because of the fatigue. So, the issue we had with sort of assessment and treatment was that they weren't getting as much out of it as they could because we couldn't see them in the morning. And so, that was a real problem but we never, never really worked out our best way really, we kind of had to just do what we had.

So, what do you think is the impact of the pandemic on rehabilitation services for people with stroke?

Um, not being seen as quickly because there's not as many therapists because they're isolating with COVID and potentially just like a slower rehab journey and the results sort of taking longer because of the impact of COVID on that person, and yeah.

Okay, and how do you think like this might affect them in terms of their mobility and recovery in the long term? I know you've touched a little bit upon it already, but if you could elaborate a little bit more?

Yeah, and so I think um......

So their mobility and their recovery in long term?

Well, so there, there is a lot of evidence that says that someone with a stroke and how they I guess recover in their first few days post stroke will dramatically influence that long term recovery potential. There is a strong school of thought and lots of evidence and research behind that. And it's interesting if you want to go look it up. But often with the COVID and it was making our patients, just like if you would have COVID yourself, you can be really poorly and you can be very fatigued and not want to do anything. And sometimes more often than not, it actually wasn't appropriate to do things. But the knock on effects on mobility and recovery was that the patients actually, like their outcomes were worse

because we weren't able to do that sort of the level of assessment, and treatment and rehab that we normally would in the first few days because of the COVID. But then again, I can only comment on what I saw in the acute setting, I was never out in the Community. And I don't know sort of like six months on, a year on, two years on how these people are doing because I've never worked in that environment, I can only comment on people I saw maybe like four weeks post stroke, who were still with us.

Yeah okay, so how was their like mobility and recovery, so it wasn't as you would......?

Yeah, so I would say we were we were perhaps sending more of them than we usually would to inpatient rehab rather than sending them home, because they were needing that much more longer in a hospital setting and before they could go home.

Yeah okay, all right. So from a therapist perspective, please, if you can tell me about the patient's needs during this pandemic in terms of rehabilitation?

Okay. I must have a think now, what are their needs in terms of rehabilitation during the pandemic? Um, I would say they wouldn't they've never changed, they've always stayed the same. Like the pandemic doesn’t really......um actually one thing, so the needs of a stroke patient will really depend on how the stroke has affected them. So, whether it's affected them physically, whether it's affected them cognitively, whether it's a lovely combination of the two, and they never change. But what I would say did change because of the pandemic would be, um, we were having difficulties sort of supporting people to go back into their own home environments, because of the pandemic and normally we'd rely on sort of family members supporting that person when they were at home. But if they've got COVID, the issue was that the family members couldn't support them if they were high risk themselves. They didn't have access to PPE, which they didn't. So we had difficulties when someone maybe like older who lives by themselves comes in, and then, if we weren't in the pandemic it'd be appropriate to send them home, but then we're having difficulties because family members couldn't go into that home environment, things like that really it.

Okay, were there any other things that you would like to share in terms of patient needs wise?

I guess on like a social and emotional level, having COVID in hospital can be really lonely, especially if you're in like a side room and all you are seeing are bodies and eyes, because everything else is covered up, and so we have lots of really really emotional patients who were needing, I guess that they were needing longer sessions, because you'd spend the first 10 minutes potentially with someone crying because they were really emotional about the situation and you having to build that like rapport a lot more with them because they simply were lonely, yeah they were really isolated. So yeah that was definitely something a lot of our patients needed a lot more of, and especially as well, because we were having to keep them so isolated to try and prevent spread, we could only treat them in their own rooms, so they weren't coming out onto the ward and seeing other people, and so yeah.

Yeah, okay, thank you for that. Um, so the next segment we're going to look at is management strategies. So the first question is what were the strategies used to manage access to services during the pandemic?

Okay, I think I probably mentioned a few of these haven't I already, everything blended over doesn't it. Management strategies, so definitely the risk assessments for staff. I would say, like definitely PPE as

well, and those were probably two main ones to manage access to services and yeah. I think I've already spoken about them all already, sorry.

You have, it was really thorough as well. The next one is, I think it's quite similar as well, so what were the strategies that have been used to deliver rehabilitation for patients during the pandemic?

So rehab, I guess it was a case of, and I would say, and following to add on top of, it is a very similar question. And again, I would say PPE and risk assessments for staff. But I would say also, we had to do even more than we usually would communicating with our early supported discharge service, sometimes on like a daily basis just for us to know "how are you looking today in terms of staff?", and so a lot of words like 'capacity' were being thrown around. So it was like, we are really having to consider our discharge plans, and whether they are appropriate based on capacity, so "are we safe to send this person home?", "can the early supported discharge team pick them up in an appropriate time frame or is it that it's not appropriate to send them home because they've been waiting too long in our opinion?". So yeah, and also, I'd say sometimes like the rehab wouldn't be what we necessarily were getting pre-pandemic, maybe someone could be seen by the early supported discharge team for like six weeks, and it was going down dramatically to maybe only like two or three. So, lots and lots communication between the acute setting and the Community was happening.

Yeah, what sort of like communication strategies was in place like, how was it for them?

Just your basic ones really; phone calls, zoom meetings, emails, that type of thing really.

Okay, were there sort of any examples of good practice used?

I guess it's just like building those like relationships quickly. I mean they were already established anyway but making sure those relationships were in place between sort of like the heads of teams and the acute settings, and then the heads of teams in the Community settings. So good examples were those relationships were already built, so it was very much "we get it all in the same boat", "let's help each other out", yeah. It's sort of like a daily morning meeting that was happening.

So this was different pre-pandemic?

Yeah, so pre pandemic it was like the service was the service we knew what to expect from that service. So we knew what was appropriate to send home and have that service, whereas in the pandemic, it was changing all the time and so yeah.

Okay right. And was there any efforts to introduce self-management programs to the patients, for their recovery?

Yeah, I wouldn't say that there was never like an official like management program at the time during the first wave or second or the third, There's nothing like official, but lots of conversation around like fatigue management, things like that, so encouraging patients to rest and not try and push themselves too hard. And that's quite typical of a stroke in itself as well. A lot of people do struggle with sort of fatigue and tiredness because you've had such a huge event sort of in the brain happen and the brain needs a lot of time to recover. And then you just whacked a respiratory condition on top of it, and so yeah lots of conversations around fatigue management. And if they were young enough, having those appropriate conversations around "how are you going to manage work life?", "how are you going to manage family life?", "how are you going to manage just looking after yourself?", things that are really yeah.

Okay, were there any like particular resources or like things like that that we're used to deliver care?

So in recent sort of months that there's certainly things that have been bought out, like information packs and things like that rom various organizations, sort of appropriate to therapies. So whether it's like physio, or whether it's OT. But at the time, we were very much going off what we were seeing in practice.

So, it was much more like verbal communication with the patient?

Yeah yeah,

Okay, were there any specific efforts taken to improve their mobility? Based on sort of the self-management stuff that you've mentioned?

No, I wouldn't say more than usual. Just maybe again, doing a much more graded approach and "not pushing yourself too hard", but we would say that anyway with a stroke. So whether it's things like "you sleep upstairs and then you come downstairs and you don't go back upstairs till the end of the day so you're conserving energy because you're not going up the stairs multiple times during the day", things like that really. But I wouldn't say that's COVID specific because I would suggest that to anyone anyway if I was thinking that fatigue is going to be sort of impacting factors to them on their recovery.

Yeah it's also sort of like functionally like were there any specific efforts taken to improve like them functionally at home and like how are they able to mobilize better at home and things like that?

Yeah so we put in, again, it would be the early supported discharge team would go in and they'd assess them, and they'd look at what their goals might be, so wherever it was that they got home they had family to rely on them, and "did they usually were I don't know, like the homemaker of the family?". So, when they got home they were only able to make a cup of tea, so we've grade it and then they want them to be sort of making a sandwich, and then they can let them cook a meal, things like that and yeah.

And did the patients benefit from these things that you guys provided and how did they benefit from it?

Difficult for me to say because I was never in those services, so I can only ever comment on like the acute side. But in the acute side, yeah, absolutely. I think there's a sense of reward with it when you first brought them into the kitchen and they're only able to get through the tasks like halfway, and then the next time you bring them into the kitchen they're able to do a little bit more, and then the third time you bring them into the kitchen they're able to do it themselves, and there's definitely sort of like a sense of reward with it sure.

Yeah okay, thank you for that. So we're going to move on to our last section, which is outlook, so sort of moving forward from this experience. So the first question is when the health services start to reopen, what do you consider as priorities for stroke services?

I guess, do you know what, so that's an interesting question, but I would say, potentially not, don't want to say your questions is not relevant, but it's funny isn't it. So like the stroke service was never at a

scaled back or dial down or anything like stopped, if that makes it, like most of it continued in some capacity. So when you say like the health service opening back up again, I guess the only thing that ever stopped was like relatives being able to visit patients, that's the only thing that really stopped from the stroke point of view. So I guess like our priorities never really changed because they were always the same.

And what sort of priorities could you, if you could mention them?

Yeah so our priorities were always to assess someone and within 24 hours. And if we can and often we could, establish what we thought would be like an appropriate care plan for them. So in that care plan you'd be looking at what things do they need to achieve and do we think they can go home or do they need to go to Inpatient rehab. So those were our priorities and they never really changed yeah.

So just to confirm this care plan is sort of an Inpatient thing that you provide?

Yeah.

Alright, so our second question is, if we were to have a way for another pandemic, hopefully not, what can the healthcare system do to ensure optimal services in delivering care and rehab?

Oh my gosh, don’t really want to think about it do you. I would say, and whether this is even possible I don't really know, but I feel like you can figure out somehow, is that like actually allowing relatives to see patients. I think it’s, what are the words I’m trying to use, I don't think you can underestimate the power of having your family there to motivate you, that they know you far better than a therapist will ever know you. And there were times when we did have exceptional circumstances where we would invite a relative in for a sort of rehab session. Normally, we will get a much like a better result where that person would improve more during that session. Yeah and so, I would like to see, it's obviously got to be safe and it's got to be risk assessed, but I would like to, I guess what I want to say is, better access for relatives to test themselves and then actually be able to come in and be part of that rehabilitation process, because you can't underestimate the power of like having like your wife or your husband and they're the ones that know you, like they could literally say to you like “I actually think you'd be like”, I mean they're essentially like, those relatives can say things to patient that you could never say to them, and sometimes it was really helpful if they said, like “Come on, like, I think you mean like a little bit daisy you can do it more than this”, and then they did. Whereas as a professional therapist, I would never say to a patient “I think you're being lazy”, and never, never cross that line. So yeah, more access for relatives to see patients in hospital would be my one.

Okay, that's all particularly based on your experience when you were in inpatients?

Yeah.

And the last question is, what can the health care system do to ensure optimal services for people in isolation, so that means, Isolation meaning that they could have maybe tested positive or maybe one of their household members have tested positive and the isolating. So what can the healthcare system do to ensure optimal services for these people in isolation?

When you say isolation, do you mean isolation at home or isolation in hospital>

Isolation um based on your experience, so if you are able to speak about both, then that would be great.

I can't speak about both but I can only speak about Inpatient isolation. And so again, my thing would be more access to technology that allows them to communicate with the outside world, because again, based on my experience and like as an occupational therapist, I look at that person's entire world, I'm not just looking at one specific thing. And a huge part of who we are, like intrinsically as human beings, is like our social communication, our connections with those people that we care about. And just being able to say you're isolated you're in like a room by yourself, and having access to things like iPads or something that allows you to communicate and with those people that you care about I think can dramatically increase that person's motivation, improve their mental health, and ultimately, their engagement with us so that they were like ultimately like achieve a better outcome in the long run, because they're motivated to engage with the therapy and the rehab that's going to get them better.

Yeah I do agree with that one as well, it was quite hard, especially when I was in you know inpatients and seeing you know relatives unable to come in yeah, definitely.

Alright, so thank you for answering our questions, um we actually do have a few more. The last one is probably like open opening the mic up to you. So, if you like to add anything, or you feel like that I've missed anything in the interview, you can feel free to share it at this point.

I don't think so. I think a lot of my answers came off the back of your questions.

Yeah it was really helpful as well to tie it in. Um so i'm just going to pass the time to Researcher, maybe he has some other questions for you, and he will carry on from there yeah.

00:00:03.210 --> 00:00:03.629
Researcher: Oparticipant.

2
00:00:04.200 --> 00:00:07.649
Participant: Before you go every day school day, is it really.

3
00:00:08.250 --> 00:00:17.699
Researcher: yeah alright, so we are recording yeah can go um yeah yeah I just once again just thank you for taking your time and wanting to participate in our research, study.

4
00:00:18.359 --> 00:00:25.830
Researcher: So, before we start with the questions um, could you please state your name and also mentioned that your key with us recording the session.

5
00:00:26.670 --> 00:00:33.150

Participant: Oparticipant yeah yeah my name is Kamal and chip and i'm actually fine with you recording the session today no problem.

6
00:00:33.660 --> 00:00:44.700
Researcher: All right, fantastic Thank you K I just a little bit of reminders just to reiterate the purpose of this study is to gain therapists and patient experience on stroke rehab during the pandemic.

7
00:00:45.180 --> 00:00:57.480
Researcher: And there is no right or wrong answer, so please feel free to express anything, as we are interested in your thoughts and experience and know your name in the reports will be anonymous throughout the entire study.

8
00:00:58.950 --> 00:00:59.670
Researcher: Especially.

9
00:01:00.690 --> 00:01:10.110
Researcher: If you need a break, or you want to stop at any time during the interview, please feel free to do so, it is your right, and you just let us know when, if you want to take a break of stuff.

10
00:01:10.710 --> 00:01:13.800
Participant: Oparticipant, just have to excuse kids and dogs in the background that's all.

11
00:01:15.360 --> 00:01:21.150
Researcher: yeah there's absolutely fine yeah and before we actually started, do you have any other questions for us.

12
00:01:22.740 --> 00:01:27.150
Participant: No, not at all, no, no, just interesting to hear what you're doing and you're about it yep.

13
00:01:27.540 --> 00:01:43.800
Researcher: yeah oparticipant that's fantastic so we move on move on to our first section of our interviews, which is experience of delivering services so, can you please tell us a bit about stroke care rehabilitation delivery service, since the first wave either endemic.

14
00:01:44.340 --> 00:01:48.060
Participant: Well, as in my actual role or what author have observed, which sort

of. 15

00:01:48.810 --> 00:01:53.040
Researcher: yeah they've been like stroke care rehabilitation delivery services.

16
00:01:53.460 --> 00:01:55.620
Researcher: Oparticipant, and what it was like for you.

17
00:01:55.980 --> 00:01:57.690
Researcher: But the first week yeah.

18
00:01:57.750 --> 00:02:08.160
Participant: Oparticipant, well, the first way for the pandemic I like I mentioned, I work in outpatient I work in vocational rehab so my role is to support people back to work after the stroke.

19
00:02:08.550 --> 00:02:14.790
Participant: And so that's people who've only been admitted safer of two to three days to people who have had more long term and.

20
00:02:15.240 --> 00:02:28.860
Participant: Like rehabilitation period as well, so for me the delivery of care was all the outpatient services stopped, and we, as all outpatient therapist were dragged on to the impatient wards and so basically.

21
00:02:29.610 --> 00:02:38.430
Participant: My role was totally totally changed and we couldn't provide the support to the people who were returning to work or about to return to work.

22
00:02:39.420 --> 00:02:53.580
Participant: In the same way we did try to do some outpatient work, but it was all virtually and it was all by telephone or via and zoom meetings or teams meetings.

23
00:02:54.210 --> 00:03:03.810
Participant: The difficulty with our for people who'd had a stroke if they had got any language problems or cognitive problems being unable to.

24
00:03:04.350 --> 00:03:23.220
Participant: Ill I actually am participate in a in a while we, it was accurate was what we used it within our hospital, some of them really struggled with it, so there was a large proportion of people who we were able to provide the service and the level that they they needed really.

25
00:03:23.760 --> 00:03:37.590
Researcher: yeah yeah in terms of, as you mentioned about like zoom zoom online and sort of trying to get patients to be involved online we're just wondering what services in the NHS or other Community services provided to the people off the stroke.

26
00:03:38.490 --> 00:03:47.220
Participant: yeah well like I say we, we did a minimal service, and it was either a telephone support so trying to do initial interviews and then treatment sessions over the phone.

27
00:03:47.700 --> 00:04:00.480
Participant: or trying to do some of the assessments actually virtually using accurate, I think, was the one that we use, so I tried doing a couple of cognitive assessments over the phone over and that was really hard work as well.

28
00:04:00.720 --> 00:04:08.220
Participant: Like yeah really hard work, especially when you've got a lag as well halfway through you're doing a timed assessment and then you get a lag on the it so.

29
00:04:08.640 --> 00:04:12.630
Participant: So that that was really difficult to do as well, because we were not allowed to do.

30
00:04:13.020 --> 00:04:29.640
Participant: Any home visits at all and prior to the pandemic, most of our work was face to face going into people's workplaces or doing home visits, so it totally change your interactions and the support that you could give to people following a stroke.

31
00:04:29.970 --> 00:04:37.980
Researcher: yeah Oparticipant, so my next question is what what sort of modification systems were in place at your workplace for people with stroke.

32
00:04:38.490 --> 00:04:46.410
Participant: yeah yeah telephone call purely under telephone telephone call and zoom calls and there was no face to face contact at all.

33
00:04:46.950 --> 00:04:57.360
Participant: And, and I suppose, when I was covering on the wards and people seem to be in hospital for shorter periods of time as well that they were they were discharged a lot quicker.

34

00:04:57.900 --> 00:05:07.080
Participant: And, and I think one of the biggest barriers as well, was like passing the information on to the family and the family, not being able to to come in and visit the people who had a stroke.

35
00:05:07.470 --> 00:05:18.600
Participant: And so, people are they had like a life changing events and then their their family we're getting really worried about them, and I think that had a detrimental effect on on people's mood.

36
00:05:18.630 --> 00:05:23.490
Participant: Definitely your anxiety, both to the patients and to the.

37
00:05:24.570 --> 00:05:30.060
Participant: To the person and the other issue we are then as well, when people will be discharged home.

38
00:05:30.360 --> 00:05:46.470
Participant: They can have the ongoing rehab, which is essential, you know, with all the yeah the research, for you know the optimum time the treatment and a lot of the Community teams weren't providing the support and the rehabilitation like they were post pandemic as well, so.

39
00:05:46.530 --> 00:05:46.830
Researcher: yeah.

40
00:05:47.220 --> 00:05:50.400
Participant: A lot of people lost a lot of optimum treatment time.

41
00:05:51.300 --> 00:05:59.130
Researcher: yeah it's quite interesting what you said, because it actually leads on to our next point in terms of the impact and needs of the patients in stroke care.

42
00:05:59.460 --> 00:06:07.860
Researcher: So we just want you to elaborate a little bit more on what you think were the problems for people who had a stroke during the pandemic in assessing services.

43
00:06:08.610 --> 00:06:16.890
Participant: yeah I think it was, I think it was a really scary time I think like there wasn't usually a Canadian speak about in in Wolverhampton we've I think we've got an excellent.

44
00:06:17.340 --> 00:06:22.020
Participant: pathway of care and those pathways seem to stop that they went from the acute you in it.

45
00:06:22.260 --> 00:06:31.080
Participant: To the rehab unit and and there wasn't as much input because there weren't as many staff because of stuff absences as well, so they weren't getting as many treatments sessions.

46
00:06:31.380 --> 00:06:38.370
Participant: And then on discharge, there was there was minimal support as well, and that was very different because we take people across the country.

47
00:06:38.760 --> 00:06:44.760
Participant: And you know there were very different services, according to which area that the person was living in as well.

48
00:06:45.180 --> 00:06:49.620
Participant: And so it was very much like the discharge planning was making sure they got equipment in.

49
00:06:50.010 --> 00:06:59.190
Participant: And then they then had like some Community input, but again, that was more telephone based rather than face to face as well, and eventually the early.

50
00:06:59.580 --> 00:07:10.080
Participant: The more vamped in the early supported discharge team, they did start going to see people more face to face, but it wasn't as frequently as you know, there, they would have liked to have seen as well.

51
00:07:10.890 --> 00:07:18.270
Participant: And again, like therapists will be important here, there and everywhere, so that the work the staff to provide that service as well.

52
00:07:18.960 --> 00:07:36.420
Researcher: yeah Oparticipant, in terms of sort of the physical beings that was experienced by patients, what do you think is the impact of the pandemic on rehabilitation services for the people with stroke, so this sort of like your mobility or like your physical i'm impacts.

53

00:07:36.570 --> 00:07:45.270
Participant: or yeah yeah yeah they missed out on the the the physio ot speech and language therapy input, because the stuff just wasn't there as well, so.

54
00:07:45.840 --> 00:07:56.220
Participant: They weren't getting the intervention that they should be doing on delight there you know the stroke guidelines and stuff like that they certainly weren't getting as much regular treatment as they should have been as well.

55
00:07:56.460 --> 00:07:58.290
Participant: yeah and I think also as well, I think it's.

56
00:07:59.130 --> 00:08:11.640
Participant: Particularly with the people we like the emotional cognitive with the fatigue and and definitely with the language as well, when people are using masks and using advisors as well that interactions and that that.

57
00:08:11.670 --> 00:08:24.210
Participant: Rapport that was more difficult for people to engage with as well and, and I think it had a massive impact on a lot of people's well being and that and I think we're seeing the aftermath of that now as well and.

58
00:08:24.840 --> 00:08:33.990
Participant: You know people just weren't getting the the emotional support that they need, as well as their physical hands on rehabilitation as well, so yeah oparticipant.

59
00:08:34.170 --> 00:08:41.490
Researcher: And like sort of translating from that like, how do you think this might affect them in terms of their mobility and recovery in the long term.

60
00:08:41.970 --> 00:08:45.810
Participant: yeah I think I think people have missed a lot of a lot of time and certainly.

61
00:08:46.830 --> 00:08:59.370
Participant: Within the Community team we noticed that a lot of people had like their mobility or deteriorated, particularly with increasing tone people used to come to light and stretch classes or botox clinics.

62
00:08:59.670 --> 00:09:01.890

Participant: And they weren't having any of that input so.

63
00:09:02.940 --> 00:09:13.980
Participant: A lot of people, we know their their increased own it was a massive problem when, after like the first wave as well, so when the therapists were going out of that stays there will be.

64
00:09:15.090 --> 00:09:22.260
Participant: losers and and issues as well, and then people who become conditioned as well, because they were able to go out so.

65
00:09:22.830 --> 00:09:33.000
Participant: A lot of people have become very on fit and then that was it an extra problems to their their mobility problems as well, so it was like almost like a double whammy as well, really.

66
00:09:33.300 --> 00:09:44.130
Participant: And again, that social interaction not being able to engage with others as well, so a lot of people were very low mood and you know felt felt very isolated and very, very scared as well.

67
00:09:44.250 --> 00:09:50.430
Researcher: um yeah, especially the mental health it's it's quite it's quite a big impact for that yeah.

68
00:09:50.880 --> 00:09:53.760
Participant: Massive yeah massive yeah definitely here.

69
00:09:54.000 --> 00:10:04.590
Researcher: yeah oparticipant so from like a therapist perspective if you could tell us about the patient's needs during the pandemic in terms of rehabilitation wise.

70
00:10:06.150 --> 00:10:12.630
Participant: yeah that they got quite intensive rehab when they will in the the acute stages, but after that.

71
00:10:13.050 --> 00:10:22.980
Participant: Like if they needed more long term like you know, like if they were in just for a couple of days in the acute services they were seeing well, but they were discharged home quicker than they would have been normally.

72
00:10:23.400 --> 00:10:31.140

Participant: And then they they like say, then the the more long term rehab was wasn't happening as frequently at all, so they might have had just.

73
00:10:31.440 --> 00:10:40.650
Participant: One session a day, rather than usually having a couple of sessions, the other difficulty as well, was when people were doing home visits as well, and people couldn't go home.

74
00:10:41.370 --> 00:10:49.170
Participant: To do a home visit to see if there was any equipment required and so again, that was, like some of the discharge is where as safe as they should be, because.

75
00:10:49.980 --> 00:11:03.570
Participant: You can't do some of the visits as safely without actually physically going into the environment and seeing all the hazards and stuff like that trying to do a home visit via zoom meeting a zoom call this the easy yeah it's not it's not easy.

76
00:11:03.690 --> 00:11:06.120
Participant: it's not a realistic picture or soul so yeah.

77
00:11:06.510 --> 00:11:19.440
Researcher: yeah Oparticipant, thank you K for that um we're going to move on to sort of a different topic now management strategies, so the first question that we have is what would be strategies used to manage access to services.

78
00:11:20.730 --> 00:11:21.750
Participant: panic and.

79
00:11:24.150 --> 00:11:25.560
Participant: I think I think yeah.

80
00:11:27.330 --> 00:11:28.260
Participant: I think we.

81
00:11:29.280 --> 00:11:32.610
Participant: You know our managers were supportive, they can, but they just dragged.

82
00:11:33.690 --> 00:11:34.950
Participant: The therapists to.

83
00:11:35.160 --> 00:11:36.870
Participant: To the the acute care.

84
00:11:37.260 --> 00:11:38.520
Participant: The problem was.

85
00:11:39.180 --> 00:11:39.600
Researcher: Sorry.

86
00:11:39.900 --> 00:11:45.870
Participant: Go ahead yeah times we'd have an award that was just full of Kovac patients as well, so.

87
00:11:46.650 --> 00:11:54.360
Participant: It depends, which therapists could actually because, if some people had their own risks they weren't actually allowed to to work face to face with.

88
00:11:54.900 --> 00:12:03.660
Participant: COPD patients as well, so that light was was difficult as well, and then obviously you've got some very, very poorly people with the cove it's so they weren't.

89
00:12:04.710 --> 00:12:09.210
Participant: You know you couldn't work with them as the stroke as much because there were two poorly to engage you know you.

90
00:12:09.420 --> 00:12:14.100
Researcher: Were there sort of any strategies used to manage access to services.

91
00:12:15.180 --> 00:12:29.700
Participant: Not not the well not that we're aware of and stuff not not not not live, where i've it was it was almost like you know we just have to follow the instructions from our managers who will bring instructed by the the the chief exec and above really as well, so yeah.

92
00:12:30.330 --> 00:12:33.120
Participant: I wasn't aware of anything different going on, really.

93

00:12:33.480 --> 00:12:41.700
Researcher: OK so moving on um what, what are the strategies that have been used to deliver rehabilitation for patients during the pandemic.

94
00:12:43.170 --> 00:12:44.580
Participant: The structured years and stuff.

95
00:12:45.090 --> 00:12:55.770
Researcher: yeah so like the first one we discuss about is like solid manage access to services and then the next sort of like strategies that have been used to deliver rehabilitation for patients.

96
00:12:56.130 --> 00:13:07.020
Participant: I think it was the same it was just like constant need you're moving stuff around really as well, and you know it's just it really depended on the numbers of people with cove read on different boards.

97
00:13:07.350 --> 00:13:14.790
Participant: staff will be moved around all the time and patients will be moved around all the time as well, so and I think it was very much.

98
00:13:15.540 --> 00:13:27.420
Participant: You know, I suppose, some of the strategies were managing the covert people after isolate before they were see in and complying with all the ppa as well, really, so they were sort of like those sort of strategy is put in place, really, but it was.

99
00:13:27.930 --> 00:13:33.330
Participant: It was a change in picture every day you just didn't know who you'd be working with the next day and.

100
00:13:34.860 --> 00:13:36.420
Participant: supposed to be able to.

101
00:13:37.320 --> 00:13:44.850
Researcher: make a sentence of life, oh yeah your your reflection um, are there any examples of good practice that has been used.

102
00:13:47.940 --> 00:14:01.200

Participant: yeah and one of the Community teams, they actually developed a they used to do a stretch class like that patient stretch class so they managed to do that online and they've managed to carry that on as well, so doing something like that has been good and.

103
00:14:03.570 --> 00:14:17.850
Participant: I think what happened as well, he actually made people value or the people services so some people who perhaps worked in community with an impatient, so we actually helped with the communication and the understanding of like the Community intermediate care teams and.

104
00:14:18.090 --> 00:14:28.260
Participant: An additional charges as well, so I think it made people appreciate different people's roles and the roles that that got on the stroke pathway as well, so I think that was a real positive in itself.

105
00:14:28.920 --> 00:14:44.970
Participant: And, and I think I think the other thing was he I think they realize what a good service that we had which is nice to know, is it that you know once it was taken away, they realize what they've what service was there before as well, one of the interesting things was.

106
00:14:46.260 --> 00:14:53.370
Participant: Is that it seemed to be one stage where we didn't get many stroke referrals so we didn't know where all the stroke patients have gone.

107
00:14:54.870 --> 00:15:06.540
Participant: There was one stage where it was like we've got at West part there's a stroke Ward rehab board and there were very few of them who actually had a stroke, so it seemed to be the numbers of people with stroke.

108
00:15:07.560 --> 00:15:15.900
Participant: and got a significantly gone down so whether they weren't coming into a hospital or what happened to them, I don't know it'd be interesting, I mean if you.

109
00:15:17.160 --> 00:15:24.000
Participant: If you've got any theories on that at all really because yeah they certainly seem to disappear for a couple of months right like.

110
00:15:29.190 --> 00:15:43.290
Researcher 1: Well yeah I don't know, maybe there we have some participants actually told us me will be will maybe get scared because of them coffee situation and that's why maybe they're.

111
00:15:43.680 --> 00:15:52.500
Researcher 1: If they have symptoms, they would stay, maybe at home and not going to a hospital or calling ambulance unless they have something for the like.

112
00:15:53.310 --> 00:16:03.810
Researcher 1: Serious and I think it's sock is serious but at that time I didn't know before it was a shocking time for everybody can they.

113
00:16:04.410 --> 00:16:17.790
Researcher 1: Think when they think to to visit the hospital or in a filibuster doctors would be visiting them at home so yeah that's maybe afflicted them decision so that's why maybe in some.

114
00:16:19.350 --> 00:16:26.700
Researcher 1: places the the, the number of people had stroke at that time, a drop down pretty much.

115
00:16:27.780 --> 00:16:30.300
Participant: yeah and it makes sense.

116
00:16:30.690 --> 00:16:34.230
Researcher 1: yeah it was really a common like.

117
00:16:36.090 --> 00:16:47.040
Researcher 1: thing that we noticed from different people our participants actually not just from one local context we actually have interviewed people from different.

118
00:16:48.420 --> 00:16:53.190
Researcher 1: out in England and they alluded to the same thing.

119
00:16:53.670 --> 00:16:56.190
Participant: Oparticipant yeah very interesting yeah.

120
00:16:56.370 --> 00:17:11.280
Researcher: yeah Oparticipant, so my next question is sort of on self management program so were there any efforts to introduce self management programs to the patient's body our recovery and sort of were there any specific efforts taken to improve.

121

00:17:11.700 --> 00:17:12.180
Participant: let's see.

122
00:17:13.170 --> 00:17:18.720
Participant: let's continue because we we work in the rehabilitation hospital so that's part of the the natural rehabilitation any.

123
00:17:18.900 --> 00:17:33.240
Participant: as well, so it's always encouraging the patients to be as independent as possible, so they would have been put in place the best as they can, and probably just with the more limits of having to use like the personal equipment, the protective in the ppa and stuff as well, maybe.

124
00:17:33.300 --> 00:17:34.830
Participant: Not a little bit more difficult to do.

125
00:17:34.830 --> 00:17:48.540
Participant: That but yeah that that carried on as much as as feasible really as well, I suppose, just a bit more awareness obviously of life and infection control and those sort of protocols have to be considered just that little bit more as well.

126
00:17:49.140 --> 00:17:58.380
Researcher: yeah Oparticipant, so we were just wondering whether there were any specific efforts taken to improve their mobility in terms of self management programs.

127
00:17:59.670 --> 00:18:00.360
Participant: and

128
00:18:02.100 --> 00:18:13.440
Participant: Not not not in our trust, not that was aware of you know we've we've got some brilliant therapist physios OTS and stuff who would have been able, people as much as possible really so but there wasn't anything and.

129
00:18:14.010 --> 00:18:19.590
Participant: Their normal duties really they just carried on providing as best as services they as they do normally.

130
00:18:20.370 --> 00:18:32.460

Researcher: yeah Oparticipant, so you did mention about like the self management programs that you sort of have been using in stroke care we just wanted to ask that the patients benefit from this and how did they benefit from it.

131
00:18:34.860 --> 00:18:44.550
Participant: Just increasing their their independence, really, as well as just being able to do it get more control at least have some control over some of their activities as well really and.

132
00:18:44.940 --> 00:18:52.500
Participant: And just that achievement that they're able to do something for themselves, rather than having to be nursed or rely on somebody else as well, so yeah.

133
00:18:54.030 --> 00:19:11.130
Researcher: Oparticipant well we're going to move on to our next section, which is the final section so it's sort of outlook and so i'm moving forward from this experience, so the first question is when the health services start to reopen, what do you consider as priorities for stroke services.

134
00:19:12.000 --> 00:19:20.340
Participant: I think a lot for their emotional well being I think there's a lot of people who've been really struggling who are isolated as well, so I think there's a lot of thought, where.

135
00:19:21.300 --> 00:19:31.140
Participant: people's moods have significantly dropped and also there they became D conditioned as well, really, and that social interaction as well, because I think a lot of that is.

136
00:19:31.740 --> 00:19:40.620
Participant: We work with a lot of people who were working before the stroke and then unfortunately they're not able to go back to work after the stroke so they've had the work taken away from them.

137
00:19:40.950 --> 00:19:46.200
Participant: they've had all the interactions and related to work taken away from them so.

138
00:19:47.130 --> 00:19:56.250
Participant: it's trying to build off that confidence to be able to go out and engage with others and do activities but feel safe as well, because a lot of people have been very.

139
00:19:56.730 --> 00:20:03.780

Participant: very worried because they've got a health condition to then go out and do social activities and there's not be many of them.

140
00:20:04.440 --> 00:20:14.400
Participant: We used to do, like our groups yoga groups women's groups, Jim groups and stuff and a lot of those got put on hold relay so what they've only really in the last couple of months.

141
00:20:14.700 --> 00:20:26.820
Participant: To to open up again as well, so he's trying to encourage people to try and get back to doing those things that helped as much as they're coming in and having like the outpatient physio ot and speech but but that's essentially.

142
00:20:28.650 --> 00:20:29.670
Researcher: yeah absolutely.

143
00:20:29.700 --> 00:20:32.310
Participant: yeah yeah oparticipant.

144
00:20:32.910 --> 00:20:45.090
Researcher: So sort of we if we were to have a minute let's hope it will never happen, but if we were to have a wave or another pandemic what can be healthcare system do to ensure optimal services in delivering care.

145
00:20:48.000 --> 00:20:49.080
Participant: yeah and.

146
00:20:50.280 --> 00:20:57.600
Participant: Personally, I feel like they need to carry on doing that the face to face stuff I don't think for a lot of patients the virtual stuff works, I think people.

147
00:20:57.810 --> 00:21:05.100
Participant: need to actually physically see people and receive actual physical treatment, rather than doing it virtually over the telephone as well, so I think.

148
00:21:05.460 --> 00:21:18.510
Participant: You know it's making sure people, which we did have you know, like the correct ppa and stuff like that, but to enable people to actually have the home visits have the Community support have the regular sessions within the hospital as well and.

149
00:21:18.930 --> 00:21:23.700
Participant: Just enable them to be seen as as much as possible complying with obviously all the.

150
00:21:24.240 --> 00:21:28.230
Participant: ppa infection prevention regulations oparticipant.

151
00:21:28.560 --> 00:21:30.450
Researcher: yeah I do agree as well.

152
00:21:31.620 --> 00:21:42.960
Researcher: As face to face is definitely important even even I, because I, you also go on placements as well, so definitely face to face is so much different compared to telephone calls yeah.

153
00:21:42.990 --> 00:21:52.350
Participant: yeah you learn so much more about somebody you get a lot better idea of the people's needs when you're working with them face to face it's it's just not the same virtually at all.

154
00:21:52.920 --> 00:22:08.250
Researcher: yeah absolutely So what can be healthcare system do to ensure optimal services for people in isolation, so this may include like people who are positive for covert or anything because of wonder ability.

155
00:22:09.000 --> 00:22:19.830
Participant: yeah I mean, maybe even if it is even that we just said it's better face to face, but even if they're in isolation, even like like TV services regarding there will be in that got somebody to actually talk to.

156
00:22:20.520 --> 00:22:29.790
Participant: or even not advise them how to do some of the exercises and some of their rehabilitation at home, I think, all that would just help, just like that reassurance as well, and just.

157
00:22:30.570 --> 00:22:36.150
Participant: The somebody out there who's listening and understand their needs, as well, really so yeah I think that needs to.

158
00:22:36.480 --> 00:22:45.300
Participant: to kick in a lot more, we did use the social prescribing and service quite a bit as well we do like that sort of contacts and stuff as well, so.

159
00:22:46.020 --> 00:22:52.230
Participant: I think services like that would have to kick in a lot more as well just to to help with those people who will, who are isolated.

160
00:22:52.680 --> 00:23:05.940
Participant: And because I think some people have ended up having quite long term needs, where if they'd had the input earlier on, maybe their emotions and feelings might not have escalated like with their anxiety and depression as well.

161
00:23:06.780 --> 00:23:12.090
Researcher: yeah you mentioned about social prescribing services can you talk a little bit more about that and.

162
00:23:12.120 --> 00:23:17.430
Participant: yeah yeah can I speak of the one in a more advanced and so they are on for people.

163
00:23:18.210 --> 00:23:27.270
Participant: Who basically you telephone somebody off and you find out what sort of groups, they They need assistance with or whether they need assistance with their.

164
00:23:27.600 --> 00:23:35.910
Participant: Their finances or any sort of advice in any other way as well, and they have like a link worker who works with the person and.

165
00:23:36.750 --> 00:23:43.980
Participant: they've linked some of our people in we like different groups, they like walking groups or reading groups as well, and just as.

166
00:23:44.580 --> 00:23:52.770
Participant: just trying to find out what the person is interested in and then like looking around for the services in the local area and trying to link them in with those as well.

167
00:23:54.090 --> 00:23:54.390
Participant: yeah.

168
00:23:55.170 --> 00:23:56.610
Researcher: yeah sorry go on.

169
00:23:57.600 --> 00:24:06.210
Researcher 1: I just want to say, this is a very, very good idea to connect be able to work in groups and to get social support from.

170
00:24:07.740 --> 00:24:11.190
Researcher 1: People who are related to that surfaces.

171
00:24:11.610 --> 00:24:23.670
Participant: yeah yeah I think what people have had a stroke just sometimes now in somebody else's in similar circumstances they're not alone, and I think that that's a massive part of them as a recovery really yeah oparticipant.

172
00:24:23.940 --> 00:24:24.990
Researcher: This is.

173
00:24:25.530 --> 00:24:26.250
Researcher 1: Also, a hill.

174
00:24:27.810 --> 00:24:38.610
Researcher 1: liberation of Beijing for self management so providing a social support to this is a very important element of.

175
00:24:39.810 --> 00:24:46.530
Researcher 1: Making people confidence in in their self management skills and abilities yeah.

176
00:24:46.620 --> 00:24:52.560
Participant: Definitely definitely yeah and they're less rely, and then on the on the Community services as well isn't it really it's.

177
00:24:53.340 --> 00:25:12.120
Researcher 1: Especially, especially where there is a long list of waiting or maybe that the surfaces are cut it down like what happened during the pandemic, so this actually social support will help to connect people with groups or other maybe social support.

178
00:25:13.980 --> 00:25:15.360
Participant: Definitely well needed yeah.

179
00:25:15.780 --> 00:25:17.490
Researcher 1: yeah yeah oparticipant.

180
00:25:17.910 --> 00:25:24.120
Researcher: Oh well, thank you for answering all the questions 0participant, I really appreciate it, I might is there anything you would like to add.

181
00:25:24.660 --> 00:25:37.860
Researcher 1: I would first ask cave if you want to add anything, or you feel we missed anything during the interview about your experience with soccer have letitia during the pandemic.

182
00:25:38.340 --> 00:25:42.600
Researcher 1: So i'm going to add anything that you feel we missed that during.

183
00:25:42.690 --> 00:25:44.220
Researcher 1: The interview yeah.

184
00:25:44.490 --> 00:25:53.460
Participant: I suppose because well because it's my part, so my services vocational rehab, but it was that return to work part as well that you know people will a lot of people will work in.

185
00:25:53.820 --> 00:26:03.210
Participant: And then they had the stroke and then night they had all the concerns about returning to work and their employers and being furloughed as well, really, so I am.

186
00:26:03.690 --> 00:26:14.580
Participant: A lot of people missed out on the significant support we tried to do as much as we could, but a lot of people missed out on that and I think people you know ended up getting quite distressed and you know in financial.

187
00:26:16.800 --> 00:26:25.320
Participant: Difficulties as well, particularly like trying to manage the the benefits sister when it's all via telephone and virtual as well really so that was.

188
00:26:25.680 --> 00:26:33.270
Participant: That was a massive part and i'd say a lot of my role has been trying to pick up those pieces, since then, and just trying to give people the support as well, but.

189
00:26:34.320 --> 00:26:39.180
Participant: I think it is asking that work question and making sure that they get the support and.

190
00:26:39.930 --> 00:26:47.760
Participant: You know that link with letting employees know liaising with employers about the stroke and whether it's going to be feasible some big to come back to work as well.

191
00:26:48.030 --> 00:26:57.660
Participant: that's still should have carried on we try to as much as we could we didn't do it in the in the numbers that you know it should have been done that, as well yeah yeah yeah.

192
00:26:57.930 --> 00:27:16.650
Researcher 1: All right, thank you very much, I will just ask also question you have mentioned a good example of self management, I think program or something, you said you know, a guru who used to rich program, I think, or something you could just tell us a little bit about that.

193
00:27:17.490 --> 00:27:21.480
Participant: yeah so it's run by the occupational therapy outpatient service.

194
00:27:23.130 --> 00:27:24.180
Researcher 1: corps for Hampton.

195
00:27:24.420 --> 00:27:25.500
Participant: yeah West park.

196
00:27:25.620 --> 00:27:27.000
Researcher 1: Rehabilitation yeah yeah.

197
00:27:27.030 --> 00:27:27.300
Participant: yeah.

198
00:27:27.360 --> 00:27:32.940
Participant: So it's very happy yeah yeah so it's it's run by the the OTS and the end it's a short.

199
00:27:33.210 --> 00:27:45.510

Participant: 30 minute 45 minute sessions just basically going through an LM stretches as well, basically, looking at like tone management managing the stretchy is trying to facilitate as much movement as possible, really.

200
00:27:45.930 --> 00:27:54.720
Participant: yeah so yeah it's usually I think people engage in it for six to eight weeks, but they can continue with it and be referred back into it as well.

201
00:27:54.840 --> 00:28:02.070
Researcher 1: And I how How was it was it delivered during the abandoned was it like online delivery.

202
00:28:02.460 --> 00:28:03.900
Participant: yeah yeah it's it seems.

203
00:28:04.110 --> 00:28:05.430
Researcher 1: yeah yeah yeah.

204
00:28:05.520 --> 00:28:12.240
Participant: yeah so it's all done via teams as well and, in fact, they also what we've just introduced is against group as well.

205
00:28:12.570 --> 00:28:26.280
Participant: yeah so it's a volunteer it was a guy who was working with the ladder brain injury he works as a volunteer and they do like a games group and do like trivial pursuit play your cards right and stuff like that, as well or online and.

206
00:28:27.360 --> 00:28:28.830
Participant: People like yeah that that's.

207
00:28:30.270 --> 00:28:34.980
Researcher 1: Tough people like this yeah yeah it was fantastic and for a lot of people that was.

208
00:28:35.100 --> 00:28:46.860
Participant: Perhaps, their only contact with other people as well, so yeah that that worked really well as well, so things like that, just like like we mentioned earlier that they're not on their own there's other people out there as well, so.

209

00:28:47.190 --> 00:28:49.470
Participant: yeah yeah yeah so they need you.

210
00:28:50.340 --> 00:28:52.770
Researcher 1: yeah john do you want to add anything.

211
00:28:53.610 --> 00:28:56.490
Researcher: I know i'm really happy with it yeah.

212
00:28:56.550 --> 00:28:57.360
Researcher: Oparticipant, thank you.

213
00:28:57.990 --> 00:29:19.950
Researcher 1: Oparticipant, thank you very much again for your time today with us and we will appreciate your participation in our study and we have a small gift is just some a gift voucher that will be sending to you think you working where Sammy.

214
00:29:20.280 --> 00:29:22.500
Researcher 1: yeah she was working right there.

215
00:29:22.500 --> 00:29:25.830
Participant: yeah you're working receiver right yeah yeah.

216
00:29:26.580 --> 00:29:31.830
Researcher 1: Because I yeah i've been working with Sheba so i'll be sending your future with Sheba.

217
00:29:33.180 --> 00:29:37.230
Participant: Via Sammy so uh i'll have to check him and make sure I get it then we're like all right.

218
00:29:39.270 --> 00:29:40.050
Researcher 1: All right, so.

219
00:29:40.320 --> 00:29:41.400
Researcher 1: yeah hopefully.

220
00:29:42.510 --> 00:29:50.460

Researcher 1: anytime from now till the end of this week, you will be receiving your future it's already on there I just wanna.

221
00:29:51.660 --> 00:29:53.820
Researcher 1: meet Sheba on campus maybe.

222
00:29:54.900 --> 00:30:01.500
Researcher 1: Wednesday or Thursday and then i'll give it to her so it'd be able to if we are you working on Friday.

223
00:30:01.950 --> 00:30:03.810
Participant: yeah yeah amen yeah yeah does not.

224
00:30:03.960 --> 00:30:05.370
Participant: Work whenever yeah yeah.

225
00:30:05.400 --> 00:30:05.670
Researcher 1: yeah.

226
00:30:05.940 --> 00:30:08.100
Participant: I know with me so i'll track him down yeah.

227
00:30:08.190 --> 00:30:09.090
Researcher 1: Oparticipant, all right.

228
00:30:10.110 --> 00:30:11.280
Researcher 1: Thank you very much K.

229
00:30:12.660 --> 00:30:12.870
Researcher 1: yeah.

230
00:30:13.800 --> 00:30:20.910
Participant: yeah what were you hoping for the be really interesting to just see the research and stuff as well as is it something you hope to publish or is it.

231
00:30:21.240 --> 00:30:23.040
Researcher 1: yeah yeah yeah yeah yeah.

232
00:30:23.220 --> 00:30:39.030
Researcher 1: I will actually we need to publish the study again so we're still in the stage of collecting our data, and we are collecting data from patients and fear August so.

233
00:30:39.510 --> 00:30:44.370
Researcher 1: i've done about it, I will be writing our baby and, hopefully, you will get down.

234
00:30:45.330 --> 00:30:48.600
Researcher 1: The result when we get finished with that yeah.

235
00:30:48.660 --> 00:30:50.580
Participant: that'd be really interesting to see it yeah yeah.

236
00:30:50.670 --> 00:30:54.750
Researcher 1: So right, thank you very much for asking, thank you for your time again.

237
00:30:55.380 --> 00:30:57.120
Participant: Oparticipant nice to have met you both Thank you.

238
00:30:57.150 --> 00:30:58.260
Researcher 1: yeah Thank you.

239
00:30:59.700 --> 00:31:00.030
Participant: bye.

240
00:31:00.120 --> 00:31:01.560
Participant: bye bye bye.

Transcript: Participant

AS: OK, so now is recording.

KP: My name is Participant P. and i'm happy to be interviewed today for the research.

AS: All right, and you agree to record right?

KP: i'm happy for the recording as well yeah.

AS: All right, thank you very much for your time, and thank you again for taking part in our study about your experience with stroke rehabilitation during the covid period.

AS: Just reminding you, there is no true or wrong answer. Just we are interested in what your thoughts or opinions or experiences that you are willing to share with us today. So feel free to express whatever Ideas you want to share with us. And also remind you, we won't use your name in any of our paper or poster that will come out at the end of this study. So feel free to share whatever information you want. And it is your right to stop at any time, stop the recording or stop the interview. And, if you need to take a break just let me know. We won't take that much of time, probably 45 minutes, whatever less or more little bit. And you have any question before we start?

KP: No that's absolutely fine.

AS: Alright, so now let us start, Participant, at your local practice. And if you can please tell us about stroke care and rehabilitation services, since the first wave of the pandemic?

KP: That's fine. So i'm quite unique, and in the first wave I was working as a band six occupational therapists in impatient stroke rehab. And so, at that time, we very much didn't know what it was going to look like really. And, a lot of people had rumbles about being redeployed yeah. And there was a huge drive to get people out of the Inpatient unit as soon as possible. Within about a month or so, that being you know, we need to push people out, I was redeployed to the Community stroke team, which was for the majority of the pandemic, where I worked. So it was an early supporter discharge team, where I would be getting people out of hospital, freeing up beds, because if they were in the hospital, they were deemed to be at risk of having covid. So it's flush them out, get them out as soon as you can. I worked with that team before, so what we're used to do, just so you know the structures, if someone could step around with assistance of one person, they can be discharged with our team, that is supported discharged. Now, because they redeployed so many different people from the Inpatient unit to the other end of the pathway, which was the Community, we then could develop capacity. So rather than just taking people as a step round with one person, we were taking double, double up people, so people that have more dense weakness, people who have more complex needs to get them home quicker, and get their rehab done at home, rather than in the Inpatient setting. And so, for me, there were benefits of that for some patients and certainly the ones who had psychological impact after the stroke. They were happier at home, it was more familiar, their family members could be there for them. And we saw some people progress really well. For other people, we didn't have the equipment in the Community for rehab that we would have had an Inpatient unit, so we will have to be quite creative with resources, and I think some people were left with more of a disability than they might have done if they were inpatient for longer. Um, ask me if there is anything you want to ask me go on.

AS: If you just give us a little bit of what have been at what are the services that have been delivered even either in the Inpatient setting or for the Community, and also what modified systems were in place at your workplace for people after stroke?

KP: So in terms of the service we provided, it was going in and helping people to get washed and dressed, and helping them to make their meals, and doing their physical therapy, their upper limb therapy, their cognitive assessments, the cognitive rehabilitation. And again, prior to that, they would probably have more intense input, but actually the drivers to get people out of hospital

basically. And in terms of the operational changes, we worked longer hours. So in the past, it was the rehab assistants that would do late shifts into the evening, so I think it was one till nine. And now because we had to see people who are assistance of two, the qualified staff had to do the same. We were working probably every other weekend, very little downtime. We were also encouraged to work from home, before that we would always be in the office together; you do a visit and you come back to the office, and it was founded upon if you went to work from home, that concept was quite alien to us initially. And I know even now that the team is still doing that.

AS: Yeah all right. So Participant, thank you for this. Let us go to talk about the impact and needs of patients during that time. So what do you think were the problems, or for people who have had stroke during the pandemic, in accessing services and having proper rehabilitation?

KP: So I think we all noticed in the early six months of you know Covid, we were very quiet in the acute services. Nobody was presenting themselves to A&E. And then now in retrospect, people have come through the system and had their second stroke, so the first one maybe was a bit more mild and that's just cracked on with it. But now they've realised if they've had the treatment back then, so I think people missed the treatment they should have had and, which is a real shame. But if they didn't present themselves because they're worried about Covid, they can't be helped, I think, other people didn't have the long rehab they should have had, or they chose to go home instead. So I think i've seen a lot more upper limb issues, especially with tone. So after the stroke it's, you know, can be quite tight with stretching and botox or splinting that can be managed, but again because there wasn't that professional intervention early on, it's been missed. When people have been discharged too early, it was good in some respects, but there have been people that I would say that they have been left with longer disabilities and longer term issues.

AS: Yeah alright. So what do you think also could be the impact of the pandemic or rehabilitation services for people after stroke?

KS: I mean, I think we can reflect on what we did in that service in terms of assistance of two people, because we can take people out of hospital earlier. I think for me there's been more of a push about this “home first”. A couple of people were driving it let's go ahead. I feel like there's more pressure now in the Inpatient unit, which is where I am now, because they have learnt from Covid that when all the resources are pulled together we can get people out of hospital quicker. And lots of money has been pumped into the community to get people home, and I think there's been a huge drive that actually: home is the best place for people versus hospital.

AS: And Participant, if you can reflect a little bit on the impact in terms of mobility and the recovery in long term?

KP: So I think if people haven't been in the Inpatient setting for as long as they would have been, mobility has been worse with disability and longer term. The same with the arm really, and with the leg, i've seen quite a lot of tight legs. But had they had that early input, they probably wouldn't have had the spastic contractures at this point. And also the equipment to practice mobility just wasn't there.

AS: And how would you see that on the long term for this people?

KS: I think if they've missed out their six weeks or so of rehab, and if they've had their six weeks of Community therapy, that in total, would have been twelve weeks. But they only had six weeks. So they haven't had enough therapy to, you know, in the long term that will be a huge disadvantage to them. I think it shows that straight pathways are really key looking at that acute rehab Community phase, and each element has had its own value, but because that was kicked out, it was yeah acute out, some rehab straight out, they have missed that intense therapy or that longevity. Does that make sense?

AS: yeah yeah, makes sense. Participant, I want you to think, from a therapist perspective, since you have been working with people who have had stroke, please can you tell me about patients' need during this pandemic in terms of the rehabilitation?

KP: Yeah sure. So they had cognitive impairments that I that I saw. And there's always usually some form of a physical disability, upper limb problem, or mobility or balance problem. One of the things I would say, was particularly prevalent was mood. Mood was a big one, because if they were in the Inpatient setting the families couldn't visit, notionally that took its toll. I think we really undervalue family input for someone's rehab recovery. And if they were at home, the mood was still sometimes an issue, and because it was such a strain on the relative so early on. So in the past, you would have had six weeks or so with the inpatient and family come visit, and they adjust to this is the new way of life. They would have an adjustment period, they didn't get that. So it was straight home, this is the reality of what things are going to be like. It's a life changing event when you have a stroke, and I think, they didn't have that processing time. In stroke sense we call it like a grieving process, where you're grieving for the person that you were, that you're this new person from the stroke and there's that adjustment period between it. Rehab or Inpatient settings offer a platform for someone to transition from one to the other, but because of Covid and then pushing everyone out, they didn't have that. So there's more strain on families, and mood I would say is the biggest one I was noticing.

AS: Yeah could you think of any other maybe needs for patients at that time?

KP: Do you mean like need things that needed to be assessed, or do you mean…

AS: No, no, what were their needs for rehabilitation during that time?

KP: i'm not sure I get what you mean by that.

AS: Like as a therapist, we want to see what do you think the patient's needs during the pandemic time was in terms of their rehabilitation, either their needs were met or not met at that time.

KP: I think they were met at a basic level in terms of cognition, physically emotionally. But I think for complex patients, where it was multifaceted and there was many layers to that person and they had cognitive problems, they had mood problems, they had physical, you know your complex patients. I would say, there were times where we could not meet that need. And like psychology is a huge need that is not met, definitely isn't there.

AS: Right yeah, so Participant let us move to another aspect about the management strategies that have been maybe implemented during that time. if you could please tell us what were the strategies used to manage access to the services during the pandemic and look down?

KP: So we had some people in reaching into the acute settings and rehab settings that worked in the Community team. So they could go in, alongside the therapists, that were trying to get everyone out of hospital, and almost do like the triage and the initial assessment first. Then they would phone our team and say this person is coming out, they need four calls a day, this is who they live with, this is the set-up. And then they would be the link person to liaise back and forth between the Community team, and they would try and push them out, but they would know what our capacity was. So it was that, having a link person at all stages of the pathway to move the flow basically.

We did a lot of video calls as well. Sometimes we worked with the person face to face, it would be like video call like we have now, or telephone calls. We usually did an initial triage on the phone, which would never have done before. You look at the priorities and the needs. The patients that were in the nursing homes, we knew they were cared for, but we couldn't see them, so that was again a phone call to the nursing home to discuss if we could offer any advice that we couldn't see those patients.

AS: yeah, can you think of any other maybe strategies that have been in place? Like did you provide your patient with maybe some resources for their maybe treatment?

KP: yeah so we had like packs that we sent out. We posted a lot of things at people. If we phone them we triage them, and it was literally just, any driving advice or send them DVLA guidelines, or "I've got a few issues with my upper limb, but it's nothing major, I don't want you to come out, but I do want to do something myself", we had little packs that we sent out.

AS: yeah and how about maybe following up with those patients?

KP: So they were all supposed to have a six month review, that we put onto a waiting list. The ones that could have it on the phone would have it as a telephone six months follow-up. Again, before, we would go out and see that person face to face, or we would call them to clinic.

AS: yeah and Participant, if you think about all of these strategies that have been used, if you can think any of them was an example of maybe good practice used at that time?

KP: I think, to be honest, a lot of the virtual stuff was actually really worthwhile, because some patients don't want you to go to their house, regardless of Covid. So it gave them more choice about how they would like to be treated. You know now as things are, going a little bit more normal, people are being offered: "do you want to be seen in person?", "do you want it virtual?", "do you want a phone call?', and it's empowering the patients to choose what they want, so I think that's really good. I think with a service like early supported discharge, and if you're working with somebody for all of their rehab goals as a qualified therapists and you're only seeing them between the hours of eight and four, you see a very different picture than when you go on the late shift, which we were doing during covid. So that rehab persistence historically would say "so and so last night I really struggled getting up stairs, I struggle with this, I struggle with that." But the qualified would never have seen that for themselves, but actually we were seeing the challenges for the rehab assistants, and then we could put things in place for the patients quicker before their evening plan, which meant they were safer in the evening.

AS: Participant, was there any efforts to introduce self-management for patients at that time?

KP: Was there any what sorry.

AS: any efforts to introduce self-management program?

KP: Yes, yeah there was a big drive on self management, and lots of things getting sent to the post. And also involving family members as well.

AS: Any good examples of self management?

KP: There is a program called constraint induced movement therapy that involves a lot of self-management with the patient having to do with shaping activities, and so they would be doing that and a family member may be supporting that. And it was also the grasp program, for the upper limb. We also pointed people to stroke association and those sorts of resources - the DVLA guidelines to driving, so it was kind of signposting where people needed to go to look.

AS: Oparticipant, was there anything to teach your patient how to do their therapeutic exercise, or maybe how to provide maybe a feedback or something on how they are doing?

KP: I don't remember anything about feedback on how they are doing. It would usually have been that the physio who had triage that patient would send a pack to that person. Rather than just sending out, you know, lots of different bits of information. Specifically, what had been raised within the initial assessment - was it an upper limb problem, was it a cognitive problem, was it actually that they just wanted some advice about mood. Is printing off bespoke to that person, we didn't just have packs (PAC?), it was what does that person need, what needs to be included in the PAC for that person.

AS: Yeah, were they involved in like setting goals for their rehabilitation, or maybe developing any maybe therapeutic plan for their exercise or something?

KP: We have a goal setting meeting on a Tuesday, and each therapist does an interdisciplinary assessment when you first go, and you set the goals with the patient then about what it is they want to achieve, so they were definitely involved. And again, as we go, you know, if it was patients we're actually seeing face to face, we talk about "right you achieved your goal if you are able to wash and dress your top half", "what's your next goal", "do you want to start getting into the shower". Despite all odds, they were definitely involved with the goals.

AS: Was there anything specific for self management to improve mobility?

KP: it's a difficult one, I know the physios did a lot of work around giving out leaflets, but I don't know whether there was any website that they suggested. I don't think they created any videos or anything like that.

AS: All right, all right, and did you think patients get benefit from this programs from self management?

KP: yeah 100%. I think there's a limit to who you can give it to you. So if someone's got a cognitive impairment, it might not always be appropriate, but if they've got a relative that can help and support implementation of self-stuff that then that's great. We would always empower patients to own their illness, and if they're more motivated to do it themselves, we know they're going to keep progressing. So I don't think that was anything unfamiliar to therapy, I think that's something we would have done before Covid anyway, and it is something that we will continue to do.

AS: Alright alright, thank you very much for this Participant. The last part of this interview is about when the health care services go back to normal, and hopefully we are almost going back to normal. I don't know about your workplace, but let us hope that. So when the services go back and re-open again to normal, what do you consider as priorities for stroke rehabilitation?

KP: it's a good question. I think it's trying to make a seamless service across the pathway, because what Covid has taught us is that it can be very disjointed. What was good is having that link person. So for the patient's journey, it's acknowledging that the stroke from the moment they are picked from the ambulance to when they're discharged from the Community team, it should be a seamless service. And I think each area needs to look at their pathway a lot more, and also their skill set so if, you know, there should be more sharing of knowledge from each area, so acute rehab and community. We should all be sharing knowledge and expertise to help and improve services. So, you know, in the acute setting, if they are particularly good at the upper limb, could they do a virtual training course for someone in rehab and vice versa, that they're specialists in. And we're all specialist straight, but there are some areas of interest that other people have more than others, and so I think that that's a big push. I think the other big priority is getting family involvement, because they've been such a big amount of time where families weren't involved because they weren't allowed to be.

AS: are you talking here about inpatient or outpatient?

KP: I think it's important at all parts of the journey. Every single stage, family involvement is really key. So, you know, communicating right away from the beginning to the end, “what we're doing”, “how we're getting on”, obviously with the patient's consent to do that, but if that's who they've chosen as a next of kin, involving them and helping them to promote. I know in the Inpatient setting now, we have, you know, we're aiming for 45 minutes per person as per S-NAP. If we are looking at neuroplasticity, that repetition and repetition for improvement, if the families can be involved, then we're onto a winner.

AS: I think also self management programs can help with that. Like you can teach them how to do, a lot of training outside of their formal therapy sessions.

KP: Definitely yeah. And I think it's looking at the capacity to be flexible with services, so we know that people in the Community can come out quicker if they choose to. There are people that it benefited to come out quicker. And if the Community services could be more flexible to have assistance of two occasionally if it was appropriate for that person because of their mood, because they didn't want to be in hospital, you know.

AS: I also think motivation is a good element on that.

KP: People sometimes don't want to be in the inpatients setting, and it's not until they get home they think “oh actually, I need to do some therapy now, but if the Community team could be a little bit more flexible, or we could work across from Community to inpatient and vice versa, almost lending out staff if to meet the needs, you know the flexibility would be so much better. Ee'd be looking at tailoring services for the patients.

AS: Is there any priorities that you would consider, if things go back to normal?

KP: I think those are the main things.

AS: Alright. Participant, let us think if we have, and probably won't have any other waves in future. But if it comes back again, what can the healthcare system do to ensure optimal services for stroke rehabilitation?

KP: I think now we know what we know about Covid that we didn't know before. We have the most appropriate PPE. It would be a case of keeping things as normal as possible for the patients, because this early in March when it happened, I had no masks for my face, I had to go out and just get on with it; we didn't have anything. So actually, now we have the PPE, there is no reason for us not to continue. We've got through it now, if it comes back we know what we're dealing with. We know how to prevent the spread of infection, we just need to be continuing the rehab as we work because I don't think cutting it short did people any favors in the long run. And we also have the vaccine which we didn't have before.

AS: yYeah. How about people who have to isolate?

KP: Is this inpatient, or we talking Community or both?

AS: Let's just talk about inpatient?

KP: Yeah, if they are isolating as an inpatient, we would still be expected to see them for therapy. We would just have to wear more intense PPE, so a different mask than we would be wearing generally. They would have no different treatment than everybody else.

AS: Yeah. How about resources Participant? Can you maybe talk about the resources that we need to ensure optimal delivery of optimal services for stroke patients?

KP: Psychology is the one area that I would say we do not have enough off, and underestimating the impact of stroke “nevermind covid”, and the you know the loss of what's happened, it's no psychological support. There is some, but not enough, so I think my key priority if I was able to make, that would be to get psychology input.

AS: Yeah all right, thank you very much Participant for your time today. I’m done with my question, I’m just giving you time if you feel that anything you have missed or I have missed in my question, then please share whatever information you think are relevant to our scope of study. Otherwise I’m done with my questions.

KP: Think it's probably the lack of support of the staff.

AS: And what kind of support?

KP: You know what, it was scary early on, it was really scary because we were being asked by our patients about things. And we were under informed, things were changed at the drop of a hat. And it was nobody's fault, but it was absolute chaos. So to then go out and put on a uniform and to be expected to be professional and act like everything was going to be oparticipant was really hard. But had we been better informed, or now we know what we're doing, to have some training on Covid and what was to be expected, we could have supported patients in understanding what that meant for them.

AS: Well, hopefully we won't see that again, you know when Covid started, It was, you know, everything was shaking up and everyone doesn't know what to do at that time. But I think now we are, as you said, we are prepared maybe to have it again. Hopefully we won't but if anything happen again, then I think we are motivated to deal with that.

KP: We've also lost a lot of very good nurses, which is really, really sad as well, because they found the strain so much. We are frontline staff as therapists that they will really in the midst of it. And I think I've noticed a short staffing of nurses, more than ever, which is a real shame as well.

AS: yeah yeah I agreed with that you're right. Is there anything?

KP: that's probably it really.

AS: Well, thank you very much for your time again. I will stop recording now.

GMT20211112-130307_Recording FEBRUARY 2022 JW - DEPTH - 33 MINS

[FEMALE RESPONDENT]
[Other comments:]

All right, so can you do it now, please?

Yes, my name is participant and I am happy with this interview being recorded.

Thank you very much again, Participant, for your time and participation in this study, so we'll start now our questions. Do you have any questions before we start?

No, none. No.

All right, so let us start. If you can, please tell us about your experience with stroke care and rehabilitation delivery during the first wave of the pandemic; what it was looking like when the first wave came and how it affected your daily practice in stroke care.

Okay, so, during the first wave, I wasn't actually in post back until - I came back into post in June. I was on maternity leave for those first three months and coming back into a role that was split across acute and community stroke rehab. The biggest change really for us during that was the fact that we went to all virtual or telephone patient contact for our community caseload. Rather than us going to a patient's property, doing a face-to-face assessment, it was all conducted via the Visionable programme. We would send patients some information via email if they had it, or through the post, and then everything else was done via the web-based format for the initial…

You said you're working in acute and community, right? Which one?

Yes.

You're working in both?

Yes, my post is flexible. Predominately, in the community, but I also do work on the acute wards as well.

If you can tell us what services did the NHS or the other community services provide to the people after stroke during that time. You said all the, maybe, resources or all the services moved to online and telephone-based…

Pretty much.

Yes, so if you can tell us about it.

Yes, so would have normally done our face-to-face post-acute-stroke rehab with patients face-to-face. Obviously, that changed. The bulk were assessments; treatment planning; lots of gesturing down videos to educate patients on exercise programmes and things like that. There were the odd patients where if we had full PPE, there were no symptoms, patients were COVID-symptom free, as were we, that we would go out and do some face-to-face delivery, but certainly in the very first - that was very few and far between. The bulk of our time, we were redeployed back into the acute wards to continue with the inpatient work to facilitate the discharges out of the acute sector as quickly as we could. Whereas normally we would see people - once they've finished their inpatient rehab, we would then see them for

six weeks post-acute whilst they're home, settle them back in at home, reintegrate with community. Between that and then the isolation for families, we weren't having as many family involvement with exercise plans. We were relying on a lot of patients to be quite self-sufficient, which is a big change from what we would normally do. We normally actively encourage family-and-friend participation in rehab.

Were there any other maybe modified systems that were in place at your workplace during that time?

On the wards, obviously it was changes to PPE/how long we were seeing patients for. We weren't doing as much inpatient rehab, but obviously a certain amount is required in order to get people home. Then, as I say, the biggest thing for us is not doing the face-to-face out in the community. Not being able to do that, and not able to take patients to shops, to banks because of all the services being closed through lockdown. It was a big challenge for us I think as clinicians because we're so used to seeing people face-to-face. To then go to a full virtual or telephone system made life quite challenging.

Yes, it's quite, I think, a big shift in the daily practice and dealing with patients.

Yes, absolutely.

Yes, it wasn't easy, I think. Participant, let us move to a patient-like aspect. What do you think were the problems for people who have had a stroke during the pandemic in accessing the services for rehabilitation?

Yes, absolutely there were quite a lot because if you think about the way that stroke affects certain patients after their stroke in terms of cognitive and communication problems, to then say that 'We are treating you via a phone or the internet,' that's quite a complex cognitive - and heavily involved in verbal language, that made access for those patients particularly difficult. For those who English isn't their first language or who don't speak any English at all, that was also problematic because we weren't able to get interpreting services involved. Those that had the ways and means to use our service through communication technology, those were the problems we had.

Also, in Sandwell, we have quite a low socioeconomic population anyway, so we do have certain patients who don't have these facilities. We have a lot of elderly patients who don't have the

internet, who don't use smartphones, who don't have laptops/tablets, so, again, that proved to be quite problematic. For those patients, they either weren't seen by us if there was a risk to them if they were self-isolating or if they were shielding. We wouldn't necessarily see them, or we would only see them for a very short period of time at a distance infrequently, so obviously their outcomes are not going to be as good. From a staff point of view, it wasn't as satisfying as it would have been if you take the pandemic out of the equation.

Well, Participant, how do you think this used to impact their physical - and maybe a different aspect, but including physical rehabilitation after stroke, like the situation of having the challenge of communication and not being seen by a therapist or a clinician?

Yes.

What do you think is the impact of this situation on their rehabilitation?

Certainly, from a physical point of view when we're doing mobility rehab, us, the physiotherapists, etc., if we - there are some patients who you need to be hands-on with to enable their onward progression of that mobility, and certainly that will have been missed. There will be patients out there now who had the potential to be more mobile than they probably are now, and that will have been limited by the fact that ourselves and our longer-term rehabilitation services, we weren't running. This sounds very candid. If we could watch people walk down a webcam and say, 'Pick your foot up a bit more. Move your weight this way, that way,' but that again relied to a certain extent of having another person in the house. For those who lived alone and whose family - because originally there weren't bubbles and things like that; you were just - if you lived on your own, you were on your own. That then made treating them even more challenging. If you had somebody who lived with somebody else, they probably had a better physical outcome in terms of mobility than those who lived alone because we would have to recommend that they stay relatively in one place to maintain safety.

What do you think is the impact on a long-term scale regarding their mobility, not having proper rehabilitation?

In terms of those that are less mobile, the onward impact for - because we have patients of all ages. You've got your working-age people who may not then get back to work. That's going to have a massive

impact on them financially and from a quality-of-life perspective. I think some of the feedback we had was not - 'The whole world hasn't stopped just because COVID is here. People are still getting ill with other things,' and there was quite a frustration for patients. You would imagine, looking at the other clinical research for the treatments that we do, if we just stopped doing them, which essentially we did for a period of time, clinical outcomes would be worse. People would be more dependent, more reliant on carers, more reliant on adaptive equipment to enable them to live independently in their own properties.

If you think from a therapist's perspective, what would you consider as a patient's need for rehabilitation during the pandemic time? Like what were the needs for rehabilitation during the pandemic time?

From an acute point of view, it was, get them up, get them on their feet, assess them. Those who required inpatient rehab ultimately did still get that to a certain level. Those that were fast-tracked through and out were the ones that - or the ones who were more walking wounded, if you'll pardon the expression, it's not the greatest, were the ones I think who struggled more, so from… Sorry, what was your question again? I got distracted.

No, no, as a therapist, we want to see from your perspective, what do you think are the needs of rehabilitation on patients during the pandemic time? What they needed most during that time for their rehabilitation.

Yes, so contact; contact with other humans. It's very, very hard to therapy somebody in this sort of situation, and then, when you're taking into that the account of the social isolation, the shielding, the loneliness, the separation from families, I think that's what really caused our patients the greatest hardship.

Participant, Participant…

Again, from my point of view, it was…

Go ahead. Go ahead. Sorry.

Yes, I was just thinking from my point of view, that was very hard to manage.

Participant, I think you are not hearing me at the participante time when I talk. You get my response late, so I disrupt you sometimes. Sorry for that.

Oh, okay, no problem.

Yes, but just go ahead, please.

Yes, I think it's more - it was that social side, both from our point of view… The building of a therapeutic relationship is pivotal to help our patients trust us with what we're getting them to do, and when you can't build that, that makes that contract between you and your patient quite hard to get buy-in into. Also, if you're trying to get people to do it self-directed, again, people's motivation isn't there as much. Then there was a lot of, 'Why can't you just come and see me?' That, as a therapist, was quite hard to take at times because you would know that you would want to go and do this, you can see people suffering, but we weren't allowed. Particularly, in the first wave, we weren't allowed to go and go to their houses. There was a lot of staff sickness.

I think from the patients' point of view also, for people who were discharged home, some of them, I think they are not happy for a therapist to come to their homes at that time because everyone was worried about the pandemic and people getting to their homes.

Yes, absolutely, and we still get that to a certain extent now. Some patients, you will contact and you'll say, 'Actually, I've worked on the ward for the weekend,' and they'll say, 'Oh, I don't want you anywhere near me now because I don't want you coming into my home.' Yes, some patients just weren't bothered and just wanted us to come and see them and were frustrated that we couldn't. Then, as you say, you had the flip side of that, which was the ones who you really thought, I could probably just about justify why I'm seeing you, didn't want you, so, yes, it was quite…

Participant, do you think patient motivation can play a role in that now after people…? Let us say, people are coming back to normal life as much as they can now, but I think patient motivation will play a big role in self-directed and self-management therapy if you want to prescribe any of them.

Yes, absolutely, it does. It does whether there's a pandemic going on or not, but the impact of the pandemic was patients weren't having that member of the family going, 'Come on, mum, just try. Just try this. Just do this,' or 'I'll do it with you.' It wasn't there, so then that… Obviously, all the news was very negative. There were queues everywhere. Everything - people were very isolated. I think then to get people to see the benefit of practising their upper limb, moving their hand, and this sort of stuff, they just didn't… That was hard to engage, but then you knew from your therapist's point of view if you didn't try to get them to do it, you knew that things would either go backwards or they wouldn't achieve their full potential.

Well, Participant, if we go back to the patients' needs during the pandemic time, their needs for rehabilitation, and you as a therapist who have been working, as you said, in acute and also as a community therapist. If you can specify the need for rehabilitation during acute and also during community because you have stated them in general. Do you see them going at the participante extent for people who are in acute or people who are in community, or the needs are different for them?

Yes, they absolutely are. Generally, if a patient can be managed in their own home with therapists coming in and out, yes, regardless of the pandemic, we would try to facilitate that, once they are medically stable, as early as we feasibly could. I think in the pandemic… We always say as occupational therapists, 'We can get a patient out of a hospital whenever we need to.' It just means that some of them are more dependent on equipment/hoisting. They're not mobile; they can't walk; they need someone to move them from A to B. I think those that we could do that with, we did, and that will have been at the expense of their rehab. In terms of specifically those that we could not get out of this hospital, we had to keep them here.

We rehabbed them very similarly to that that we would have done because they were here; they were in our bed base. Yes, we were more socially distanced, but if we still needed to physically support a patient to do something, then we would. You would breach your social distancing to allow them to do that and to treat them, whereas, in community, there was a very big shift. We weren't hands-on. We weren't seeing them. We were doing the best we could via a screen, via a phone call, whereas actually, in acute, we were still providing that face-to-face, that contact, that hands-on care. Albeit we were seeing - there was the COVID risk, we were still able to deliver that service.

Well, Participant, thank you for this, and let us move to another… We have talked about patient need and the situation for you as a therapist during that time, so now I want you to talk about the strategies that have been used to manage the situation during the pandemic. Strategies to deliver the rehabilitation or maybe strategies to help patients to access the services for rehabilitation. If you can tell us about those strategies, please.

From a community perspective, one of the strategies we used was at the point of triage, so when we get the referral in, we were doing a more in-depth triage, so that then we could send education material/ assessment material to the patient at that point, ready for their virtual visit with the assessing therapist. Some of the cognitive assessments that we do, we would send the part that we need the patient to fill in. We would send that to them. Then, when we did the virtual visit, we would complete the bit that they've had posted to them with the bit over the phone. That strategy, whilst it worked, it will have skewed some of the results because patients will have been able to look at what the materials were in advance of the assessment, but as a strategy, it was the best-placed alternative we could think of.

It was somewhat successful. It did help. It gave us a starting point. I think, again, posting materials tended to be the biggest strategy. We did have a couple of patients who we lent - we only have a couple, but we did lend an iPad to a patient to enable access. We didn't have enough of them to go around, we had one, and it was a case of is there anybody in somebody's family, who has this accessible technology, they could borrow it from within the family, or friends? Certainly, I can think of one gentleman who had - I think he'd lost a smartphone or something. He had internet access, but he didn't have the actual piece of hardware to use it, so we did lend him that for a period of time so we could work with him. Again, that was just luck of the draw. On that day, we had something available, and we were able to provide that.

What have you been providing through those devices? Were they only for meeting them online and having maybe online sessions or rehab or whatever, or you maybe sent them some resources for - like exercise videos or something?

Yes, so we send exercise sheets, information sheets. We've done that electronically for a while anyway trying to save the trees, and we still do that now because actually, it works quite well, so we had been doing that as well, that sending of information. We used YouTube a lot more. Videos that were out of our sources, that were ones that we'd found, that we were like, oh - some of the bathing equipment, for example, that we have access to. The equipment-loan stores would fit something and then we would

send them a video to show them how to use it, and then we would watch them, obviously dressed, trying to access their bathtub, so we were trying to use those videos as well. Some of them were better than others, as you can imagine, but those do work, and some of the videos we still use. Although now if I'm tending to show somebody a video, it tends to be I'm normally there with them and I'm just showing it them for - so they can see in action in advance of something else being delivered, for example.

Can you think of anything that you felt, at that time when you started using this strategy, it was an example for good practice? Was there anything you would consider as good practice?

I think certainly some of the upper-limb-type exercises where you've got a patient seated who you would have gone out to facilitate, but you would have been more of a 'Keep going, keep going, keep going. Do this,' change and demonstrate rather than needing hands-on, I think that was good practice. The area that we cover is geographically quite large, so we can lose a lot of our day travelling from A to B. What we noticed in the pandemic was we were actually able to… For those patient groups, we were able to see more because we could literally hang up from one meeting and go to the next one with a 30-second lag time, whereas ordinarily, you'd have at least a 20 to 30-minute drive from patient to patient. That was good practice, and the NHS wants us to go to more telehealth medicine. Some of our triage. We did our triage virtually as well, so rather than just a telephone call, we were doing that face-to-face, and I think that has some scope into the future. It just is very reliant on your patient population having the technology and the facilities to access that.

Working on that and introducing the telehealth thing and all the strategies that you have used, was there any effort to introduce self-management programmes for patients after stroke?

We already do promote very much a self-management type approach and strategy because if the patients are waiting from week to week to week to see us, there's a lot of days in between, just because of the way our service is configured, where they wouldn't have rehab. I think as a team, we've switched the way in which we introduce our service, so we're very much more of, 'We come. We guide. You do.' I think before, we'd have gone more, 'Actually, we will come and we will do with you,' whereas now I think we do more, 'We'll guide you, but the effort, the emphasis is very much on you because we may have short-notice cancellations, because we may not be able to come and see you as regularly.' If we get symptoms, we still have to self-isolate pending your [sic] PCR, so that would have an impact. Whereas now, by encouraging that self-management, patients, whilst they miss out maybe on the clinician time, I

don't think they miss out as much on the exercise planning. They know the expectation is somewhat upon them to do their own thing.

Was there anything of these self-management programmes targeting immobility in particular?

There were some. Probably, that would be more what the physiotherapists would have concentrated on more so than ourselves. Yes, we would certainly be saying to those that just - that needed practice, that didn't need the hands-on element, 'You need to be doing so many laps of your living room. You need to be going into your kitchen every hour.' Yes, we were still trying to set targets of how often to get up and how often to do things, but the ins and outs of the specific mobility rehab would probably be best - yes, I'd chat to one of the physios about that more so.

From what you have seen, did the patients benefit from that? Did the patients benefit from the plans?

Yes, absolutely. Yes.

You think they are benefitting from them?

Yes, I think they benefitted from it in so much as we didn't have an alternative. Whilst it was not what we wanted to deliver, we would never have… You don't set out when you qualify to only see people down a phone. I think they benefitted from it in as much as they possibly could. I do think some of the outcomes will have been limited.

Well, yes, and I think they are better than having nothing to do, or nothing to provide for the patients at least.

Yes, absolutely.

Participant, let us move to the last part of this interview. It's about actually when the health services start to go back to normal as the time prior to the COVID era. What would you see as priorities for rehabilitation after stroke when the services go back to normal? What are the priorities for stroke rehabilitation?

I think it's just giving patients the access again. It's reinstating that face-to-face. Most of ours, actually we have our service. We now provide the participante level of access to service that we did pre-pandemic, so our initial assessments are now done face-to-face unless a patient requests that it isn't. The participante with follow-ups. They're all done face-to-face unless either we don't need to, but that would be - that's not because of a pandemic reason, or if a patient is requesting. I do have a patient currently now who still doesn't really want us to visit. She's much happier for us to converse through WhatsApp calls rather than a face-to-face, and we respect that. I think reinstating patients and getting them back to being able to interact with their community as things reopen, that reintegration. I think those are the things where we will be able to facilitate more as things return to normal.

Participant, let us think, if we have another wave of the pandemic, hopefully, we won't, but let us think that way, we need to consider that, what can the healthcare system do to ensure optimal services in the future for delivering of stroke rehabilitation?

Obviously, it's making sure that we have - trying to give patients the access if we can, but it's how that would be, on the ground, delivered, whether we have a bank of iPads and whatever that we can literally post through somebody's door and say, 'Here we go. This is what we've got. This is what you can use.' I think if there is another wave that hits us and we end up similar to how we were before, I would envisage that we will just be PPE-d up to the eyeballs and we will carry on. Especially with the vaccines and the fact that that will become mandatory for NHS staff. I think all of those things are the things… We need to work out how to live with it, don't we, rather than going into hiding almost, but it is a challenge. I do anticipate we will just be PPE-d up and essentially just carry on.

How about the plan for reaching people who are in isolation?

From our point of view from delivering ours, it is we are still sending those patients - if they're isolating because they are symptomatic or somebody in their house is symptomatic, we wear our FFP3 masks, the PPE, and we see them at the end of the day. We try to reduce that risk of going from a patient who is in that environment to somebody who is at risk. We see them at the end of the day, get changed, go home, shower. Those sorts of infection-control strategies are probably the ones that are mostly in place to help try to mitigate that risk.

Participant, I'm done with my questions. Thank you very much for your time. I just want to give you the mic if you want to add anything or share any information about your experiences with stroke rehabilitation during the pandemic time. If you feel we have missed any points or you want to add anything, please add that now, or you can reach me later. If you remember anything later, you just give me an email with whatever information you want to add. All right?

Yes. No, I think we've covered it, yes.

Yes, go ahead.

It's just been hard. It's been hard work, it's been very demoralising, and it's been very tiring, I think is probably the safe way, for us and for our patients because we want to do the best we can and we haven't been able to, but things are improving, so fingers crossed.

It is. It is. Yes, yes, let us hope that way. Thank you very much again, Participant, for your time. Participant, we have a small gift. We have a shopping voucher as an appreciation for your time today. If you can send me your address, please, so I can post it to you, please.

Yes. Oh, thank you.

[Thank and close]

[END OF TRANSCRIPT]

GMT20211116-173218_Recording FEBRUARY 2022 SR - DEPTH - 39 MINS

[FEMALE RESPONDENT]
[Other comments:]

My name is Participantand I agree to the recording of this interview.

All right, thank you very much, Participant, for joining us today. Thank you for your time.

[Moderator explains the research]

So Participant, please tell us about stroke care and rehabilitation services since the first wave of the pandemic, and how that affected daily practice of stroke rehabilitation.

Yes, so thinking back to March 2020, I was working on a hyperacute stroke ward, and we saw the numbers of patients drop down very significantly. So we had 18 beds on the ward, and I think it went down to about five patients, and we had so many empty beds. Then the level of COVID patients started to rise and so there was a number of staff that were then redeployed into COVID, HDU and ITU services, of which I was one of them. Just because I'd worked in the Trust for ten years, and so I had the skills to go and work within the HDU team, even though I'm the team lead of the team on the stroke unit, but it's just about who had the right skills for doing those things. So myself and a colleague went over to that team and then left a few of my colleagues on the unit.

Participant, was the reduction in patients because of COVID, or because people may be worried to come to the unit?

I think it was a combination. My perception was that people were a bit worried about coming into hospital, so if they had minor strokes, they were perhaps staying at home, and then those people were being seen through more TIA clinics and then being referred into ESD, without the need to come into hospital. I think, probably, some people, I guess they weren't being checked on as regularly because people couldn't go in and see people at home, so perhaps strokes were going unnoticed, and so those people didn't come into hospital. Yes, it's interesting because that was only quite a temporary reduction from March. I think, within a month or two, it was back to very similar levels, and has been ever since really, so I think that's been the challenge, as well as moving wards a couple of times. We shared a ward with - we were on one half of the ward with the medical ward, and that ward was going to be changed into a COVID ward, and because patients are quite vulnerable, we were moved to a different ward. Then as the numbers just kept going up, in terms of COVID patients, we moved wards again, and just access to resources, like a gym for doing rehab, was taken away from us quite early on really.

A mixture of not being allowed to use it because of infection control and then not actually having access to a space because every space there was, was changed into a patient space because of needing them for pure numbers of patients really.

During that time, Participant, if you can tell us, what sort of rehabilitation services were delivered to a stroke patient at that time?

Yes, I think there was quite a focus on discharging patients from hospital as soon as possible, which was always our approach, but was maximised because of, I guess, people were worried about older patients, vulnerable patients catching COVID, and so if they were safe to be transferred into the community, they were. Then ESD teams took a bit of time to get up to scratch with what PPE they needed to wear, and whether they could go into people's houses, whether patients were happy for people to come into their houses - some patients didn't want that - and adapting services. So yes, I worked in an ESD team in the past, and it was always just going round in a car, seeing patients, with very little technology used, and I think, quite quickly, things changed to doing virtual - lots more phone calls, lots more virtual work, which I think was needed anyway, but it took a little bit of time to catch up on those things, and I think… You see some people coming back into hospital, like, another stroke, that had a stroke during that initial time, and they just described the fact that they didn't really get very much support when they were discharged from hospital because… I think people feel that anyway.

Yes, there's research with the Stroke Association that just says that people do feel a bit abandoned when they're discharged from hospital, and I think that's probably exacerbated.

Yes, well, this actually was reported by some patients, even before COVID, but I think it got worse during the COVID time. Participant, what else can you tell us about modified systems that were in place at your workplace?

Yes, I think we were probably quite lucky. I think, some hospitals, it was like, no rehab just at all. We carried on with doing rehab that was going to get patients out of hospital, and then people that required ongoing inpatient rehab would still receive it, but I think if other areas, they didn't have the staff in order to provide that, we were then - because I moved - it was only for a couple of months. Obviously, been there ever since, but we had staff move into the team from, like, neuro outpatients that didn't - when they had all their appointments cancelled, they moved staff into our team, and so that was bolstered up. I know some people described that they were actually able to do more because there were less patients to start with. They did more rehab than they had done before, but I think that quite quickly levelled out again to how things were before. I think, things like not being able to access… Not so much from a physio point of view, from OT. Access to kitchen for assessments, access like the gym, access the stairs, even, and people say, 'Just take a step to the bedside and assess whether the person can do it.'

Of course, with stroke, there's so many other factors that affect that, in terms of visual perceptual, the ability to hold a banister rail, that sometimes, those things - so you're making judgements in less than ideal conditions, but, I suppose - and our ESD team did start to then go into see patients.

When did they start going into patients?

Yes, I think they probably started quite quickly, but they had to go by, like, a risk assessment process, so lots of people, I think, set up these risk assessments. So you think, is the person at risk of deteriorating? Are they unsafe? Are there things that would mean that they warrant an in-person visit? So they would use criteria to work out whether to see the person, in person, or not. We had quite a few patients catch COVID and be on COVID wards with a stroke, which I think was even harder, probably, because they - a lot of them were completely well patients. They happened to have it, but were completely asymptomatic, but they were transferred to a COVID ward where you had non-specialist staff. Nursing, medical and - and we'd go to see them, but it was difficult because of environment and things.

Social distancing and everything.

Yes, well, that and PPE, and… It was very much that you just wear level one PPE when you're seeing stroke patients, and when you're - you're in very close contact with the person when you're doing, like, sitting balance work.

Everyone was scared at that time. It was an uncertain time for everybody.

Yes, I think so, but also, yes, they would say, 'Oh, you could put a mask on the patient if they're coughing and things.' You could do that, and I think that hasn't necessarily changed that much. I know Public Health England have changed their guidance, in terms of thinking about the length of duration, how close a contact you're going to be with the person and do a bit of an assessment about what PPE to use, but individual - our hospital Trust has not changed their policy at all, with regards to that. I probably should be upfront and tell - it is an area of specific interest to me, this, because I did my master's dissertation on this specific topic, actually, that I've just finished.

Well, congratulations for that.

Thank you. So it was a similar exploratory study, but it was a survey, instead of interviews, so my experiences and, obviously, the experiences I know about lots of different people - just to make sure that those are distinguished, but yes, visitors is another big issue.

Yes, this is actually my next question. I want to ask you from a patient point of view, what do you think were the problems for people who have had strokes during the COVID time? I mean patients' problems.

Yes, so starting from when they're admitted to hospital, I think the actual medical treatment was as is, so people received thrombolysis, they received a thrombectomy if they needed to. They came to the ward, but lots of patients, as a result of stroke, have communication problems, and they're older patients that have hearing problems or visual problems, and so wearing masks with staff is really difficult for them to understand, hear. Also, just in terms of recognition of people. We find that people - it's a lot harder to recognise someone when they're - more than half their face is covered with a mask. So you go to people on a daily basis and they don't remember it's you. They think it's someone completely different, so it's quite hard to develop that therapeutic relationship with a patient when they think you're someone completely different, it's not the same person. Yes, they're scared about getting COVID in hospital as well. They want to get home. Yes, they're worried about having had a stroke, they're worried about COVID as well, and then getting the treatment that they need really. They're not seeing loved ones. Now it's fine. We have hour visiting slots.

You can have one visitor for one hour, but when there were no visitors at all, that was very difficult for people that relied on their family a lot. We had a very good ward sister, and she would allow us, in really critical circumstances, to bring relatives into the hospital when it was really needed, but the majority of the time, visitors, the person's family, they don't really understand. You say, 'Oh, they've had a stroke,' but stroke is such a big scale of things. You can say, 'Well, they're ready to come home now.' They're fine, they have no symptoms. Their thrombolysis has worked and they're back to normal, and relatives can't believe it. They're thinking, oh, this is too soon, or you're trying to impress upon them how unwell somebody is after a stroke, and they're not getting it because they can't come in and see it with their own eyes, to see how their relative is. The confidence of looking after that person at home is difficult to gauge whether they're going to be able to do it, and then, sometimes, you discharge people with the best will in the world, hoping it will work, but families don't necessarily know exactly how hard it's going to be to look after…

Yes, it's not easy at all.

A lot of the time, people didn't want formal care, so any kind of reablement carers or things, they - the families were taking that aspect on because they didn't want additional people into their houses during the COVID pandemic, so… I know COVID clearly hasn't gone away, but it's felt very how things were before, aside from we still don't have a rehab space, but we've got… We've had increasing numbers of patients admitted on a monthly basis. Before, there was that very slight dip, but it's just going up and up really, in terms of admissions per month, so it kind of feels fairly normal-ish, apart from the fact we still wear masks, and we have no space for doing bed-based rehab with a plinth and with parallel bars and those kinds of things, which is obviously - I think, does impact - we do our best, but it impacts the patient, and they're not necessarily receiving the best quality of rehab as they could be.

From what you have seen, what is the impact of not getting a broad rehabilitation on stroke survivors, especially on a long-term scale?

Possibly not recovering to the extent that they might have done, because that initial phase is really important, and if they're not able to work on - use a tilt table, work on sitting balance and do those tasks. Just even spending time away from their bedside, which is really beneficial. It's difficult to really measure it, in terms of whether people are going home - you wouldn't be able to look before and afterwards, because there's so much variation, in terms of levels of patients, but yes, maybe you're sending people home a little bit more impaired than you might have done beforehand, and relying on services which - in the area that I work, you have ESD for six weeks and then, after that, it's generic community therapy or outpatients, but you have to be able to travel to an outpatient appointment, so there's no…

They have a long list in some cases.

Well, absolutely. Their services just stopped completely during the pandemic, outpatient services. Although ESD was able to carry on, neuro outpatient services just stopped completely. They're up and running now. I don't work in that area, so I don't really know, but I think now they've got to the stage where, if they need to see people face-to-face, they can, whereas I think, initially, it was just telephone appointments and telling people what they needed to be doing. So I suspect that inpatient experience isn't now that different, but I wonder, yes, how - the people that had strokes during the pandemic, how -

unless someone is actually quite good at advocating for that person, or they go and they say, 'I need more therapy,' they've missed out, and there's not really much way to find out who those people are, and how they access more therapy, and actually, is it too late now?

Yes. Well, if you would speak about, also, mobility. The impact on mobility. You are an OT, right?

Physio.

Oh, physio, sorry. So if you can talk about mobility, because we have a focus on the impact of COVID - rehabilitation during COVID, on mobility in particular. How do you see the impact on mobility recovery for those patients?

It's tricky to tell, because either patients are quite good, and they're mobile from very early on, and if they're not mobile, then they tend to go to inpatient stroke services, which is offered in a different hospital. So the average length of stay of our unit is about six days, so either someone is pretty good and they're mobile, and they can go home, or they're not really - or they make very quick improvements and are able to go home.

Let us talk about people who you think need some sort of support to make a good recovery with their mobility. How that will impact them, especially on a long-term scale.

We saw a lot of people coming into hospital, having become very deconditioned over the lockdown period because people stopped going out because they were scared. They stopped going for walks, they stopped really doing much at all, and so quite a few people had been deteriorating before even they perhaps had a stroke, and then it's even worse after that. Yes, I suppose, risks of more people needing care in the community, more people needing to go into a care home because they're not mobile, because - yes, a lot of the time, there is a lot more to it than mobility, but mobility is used as the major factor about, can this person go home or not? Clearly, there's other things that need to be taken into consideration, but often the physical aspects are the ones that are taken into account.

Yes, well, thank you, Participant. Participant, from a therapist perspective, what would you think were the needs of stroke survivors for rehabilitation during the pandemic time?

Their needs were the same as before, but it's just what… Yes, whether they were able to get access to the services to meet those needs. So yes, regular intensive therapy from all the disciplines that are required. I think we're quite lucky, in terms of staffing on our ward, and we're often able to meet the SSNAP targets of delivering 45 minutes of therapy, but yes, some other teams, perhaps, were not before, and then during - they're still not, in the pandemic, able to do that, so… Then at the stage where they're either ready to go home, or they're ready to go to inpatient rehab, that they receive the length of time that's required to meet their goals and, ideally, return them back to as good as they can be. So whatever their needs are, based on the assessment, and then the goals that are set.

Is there anything in particular you felt, during the pandemic, the patient needed in particular for their rehabilitation, or do you think there wasn't something specific for that time? They only need what they need on a regular basis.

I can't think of anything specific. I guess it's aspects like, who is going to do their shopping, who is going to do other things that were made more difficult in lockdowns? In terms of the rehab, we know what people need. They have a certain type of stroke, you would know what the guidelines suggest they should receive, and that was the case before, and it's the case now.

Yes, all right. Thank you. Let us move to another aspect about the management strategies that have been used during that time to ensure optimum service delivery, or maybe just to deal with the situation. You have a sudden cut of the services and maybe you weren't allowed to see patients, or maybe to deal with them as you did before. So at that time, what were the strategies that had been used for service delivery during the pandemic?

I suppose, yes, the redeployment from other teams to make sure that there's enough staff on the ward.

Any alternative ways to maybe deliver the rehabilitation programmes?

In an inpatient setting?

Yes.

I'm convinced that, in the community, and in inpatient settings, more rehab settings, we never really delivered any group therapy before the pandemic, so that's not necessarily changed. Working with patients' relatives. Each ward was bought a tablet, which mounted on to a table, and you could set up Zoom meetings with families, so the patients could keep in touch with their families and have virtual MDT meetings with families.

Was there any effort for maybe self-management programmes or interventions?

Yes, sometimes we probably sent patients home with some exercise programmes because they might not be seen by ESD as quickly as they are normally. Patients go home and they're reviewed the next day by ESD, so there's not much need to do that, and then self-management is more taken on - but I suspect ESD will do a lot more. They'll probably set some exercises or some therapy for them to do, and they might review that via a phone call.

Is there any example of good practice on self-management during that time?

Not really, within a - within an inpatient setting, as soon as someone…

Maybe not in the acute setting, but when you prepare patients to move, or to discharge to the community, or to their homes, do you prepare them or introduce any self-management programmes?

I guess, occasionally, some exercise programmes to do, but often things change quite a bit when someone goes home. Even their goals change quite a bit. Yes, you set some goals with someone, and then they go home, and they realise, actually, what they do want to be able to achieve, because they're back in their own environment. So the ESD team will take on a lot of that self-management aspect of things, so we probably don't do a great deal of that necessarily, no.

Yes. During inpatient time, was there any self-management directed for mobility outcomes, or to improve mobility for your patients?

If someone could probably - if they could walk, they probably would have gone home.

At that time, yes.

Beds are, and continue to be, extremely tight. Like, this morning, we had 86 patients waiting in A&E for a bed. There's not a single bed in the hospital, so you cannot afford to keep someone in hospital that can walk safely on their own really, so very little of that.

You are not the first one who is saying this. I have heard this many times from people who are working in acute units. They say, 'Our only priority at that time is just to get people safe, and discharge safely to their homes,' and to save some beds for other people who are coming.

That's why, yes, ESD teams need to be better resourced if we're going to do that, because they need more staff, because we're sending people home needing more therapy.

Yes, all right, so let us go to the last part of the interview. It's about, actually, when the health services go back to normal, and hopefully, we'll see that. I think we are kind of going back to normal. So what would be your priorities for people who have had a stroke, as a therapist?

It's difficult. The phrase 'back to normal', I don't think there is a normal. I think it's a new normal. I don't think it's ever going to go back to the way things were. There won't be a point where it's like, today, everything is fine, so I think that's a bit tricky really, but…

I mean we now get rid of the lockdowns and some of the social distancing measures. So hopefully, life will be going back to normal, or semi-normal, or new normal, as you said, and you are allowed to practise whatever you were practising before the COVID time. So what would be your priorities for stroke rehabilitation?

Well, yes, having the resources, so that's from equipment and a gym area on the ward to use, as well as staffing resources, so having sufficient staff. We've been very lucky because we've actually had more funding during the COVID pandemic because we've increased our thrombectomy service to 24/7, whereas it was just Monday to Friday, 8:00 'til 4:00. So there's more money that comes with that, and so we've been able to employ more staff. So we now work on the weekends, as well as the week, so we're lucky that, actually, for us, we've actually made a really important step forward during a really difficult period of time, but that's not the same for everyone. So yes, more resource is probably what is needed,

as well as, I think - training, I think, has probably fallen by the wayside, in terms of staff, so staff being able to deliver high quality rehab because, yes, what comes with - yes, increasing resource and staffing, people have to actually know what they're doing, and I think staffing is difficult across the country really. It's quite difficult to fill positions, particularly OT positions.

Very, very difficult to fill them with suitable people, so it's not almost as easy as saying you just need more money, because money isn't the… Sufficiently qualified staff to do the job. You can throw as much money at the problem as you want, but we can't often get more than one applicant for a job that's a permanent Band 6 specialist job because, yes, there just aren't the applicants really. So yes, the resource really, as well, because yes - I don't know what patients would want. I suspect they don't want to stay in hospital longer than it's needed, but they want to feel supported and that they have probably a longer period of rehab when they're discharged from hospital. That's probably the area that needs the most significant work because the patient is unwell, they've come into hospital, they're there. The therapists are on the ward as well. They're going to get seen, whereas I think it falls by the wayside a little bit when the person goes home from hospital, so probably that side of things is…

All right. Let us think, Participant, if we have another wave, and hopefully we won't, just let us think that way. If we have another wave of COVID or any other pandemic, what would be a good plan for the healthcare system to apply to ensure optimum delivery of the services? Here, I mean stroke rehabilitation services. What would be a good plan?

Well, I think, certainly, after the first wave with the other peaks, so after the wave in March 2020, there was a pretty significant wave in January 2021, and a lot of things - we learnt a lot of things from the first wave, and then put them into place in the second wave. It's about retaining staff in the areas that are needed, but you have to think about the priorities of the Trust as well. So as much as they might say, 'Well, you have to keep specialist staff in specialist areas,' you have to think about the priorities, which are ITU and HDU. In terms of physio priorities, respiratory comes first, and so however much - I was redeployed a second time in January 2021, and that was delayed and delayed and delayed until the last possible moment where there was nobody else they could take across. So they did learn things from the first wave, and tried to delay and keep people within their teams so that that was better, but I think it's tricky to know, and I think having a bit of a risk assessment or a way of - a decision-making tool, almost. So should this patient stay in hospital?

Is the risk of them catching COVID so sufficient that, actually, they would be better off receiving less good rehab but they'd be safer at home, or are they a younger patient where, actually, their

risk of COVID is not particularly high, and they have really high needs from an inpatient rehab setting point of view, in which case, they should probably stay in hospital. We were quite lucky. I heard, in other hospitals, lots of stroke wards were hit with COVID, and a whole ward caught it and lots of patients died. Probably, they used strategies to try and stop that from happening, but we did hear it about lots of units locally to us, because we were on ambulance divert for those hospitals where they'd had to shut the whole ward because of COVID outbreaks. So that becomes difficult when that does happen, but yes, I think, probably, maintaining the ESD and the community services on discharge, and how that happens, is probably the most important thing, but everyone is different in terms - if they can engage with Zoom, or if they can engage with virtual things - sometimes, you just need to go, and you need to be there in person.

Yes. How about people who are in isolation or who need to be isolated? What would be a plan for…

Because they're vulnerable, or because they have COVID?

No, because they have COVID.

So if they're unwell from COVID from a respiratory point of view, and that outweighs their stroke needs, then they would probably go to a respiratory ward to have specialist care for COVID. If their stroke needs outweigh their COVID needs, then they would be in a side room on the stroke ward and they wouldn't be moved now, which is different to the process that happened in the first wave, where every single patient, if they had COVID, they just got shipped off to another ward. So they can still receive the specialist care, but they often have to wait out their isolation period in hospital, before they can move on to a rehab hospital because, yes, the inpatient/stroke rehab units will not - yes, they won't take someone that's COVID positive and needs to isolate, so that person would have to wait out their 14 days in hospital, whilst they're still getting rehab and being seen. Right at the start when there was - everyone said, 'Avoid at all costs going and seeing a patient that has COVID.' That's not the case now.

It's just, put on the appropriate PPE and do what's needed, within the confines of, you have to keep the person within their side room, so you can only use the resources that you've got in that room for seeing them. That's what we're working with the majority of the time anyway now, without having a gym to take patients to, so that's sort of as per normal.

Yes. All right, thank you, Participant, very much for your time. I'm done with my questions. I'm just giving you the opportunity, if we have missed anything or you feel you want to share with us about your experience with stroke rehabilitation during the pandemic, please add it now.

No, I don't think so. It's probably just about whether or not we're going to see more patients coming in with stroke because people have become overweight, unfit, they're not exercising. Older people have been isolating themselves and they're probably increasing their stroke risk factors over time, so whether that is going to continue to increase the number of patients that we see, which is going to continue to put pressure on the resources that are already quite stretched anyway, I guess we'll have to wait and see whether that does happen. I think, now the service is fairly - the inpatient service is fairly similar. Without working in ESD, I wouldn't be able to tell you what that's been like, but I suspect it's not been a particularly easy time for stroke patients to have a stroke during the pandemic, particularly in the periods of the lockdowns. I think those kinds of periods are probably the hardest, and then the rest of the time has felt fairly… Well, either it's gone on for so long, it feels like normal, and you forget how things were before March 2020, but yes.

All right, well, thank you very much, Participant, for your time again.

You're very welcome.

[Moderator gives details of gift voucher, thanks and close]

[END OF TRANSCRIPT]
