# Supplementary material for: Exploring the experiences of people who had a stroke and therapists who managed people with stroke during the COVID-19 pandemic: An exploratory qualitative study
Source: PLoS One. 2023 Feb 28;18(2):e0282325. doi: 10.1371/journal.pone.0282325 (PMC9974115; doi:10.1371/journal.pone.0282325)
Supplement: S2 File — (DOCX) [file pone.0282325.s002.docx]

**Appendix A:** Topics covered in the therapists’ interviews.

**Experience of delivering services**:

1. Can you please tell me about stroke care and rehabilitation delivery services since the first wave of the pandemic?
2. What services did the NHS or other community services provide to the people after stroke?
3. What modified systems were in place at your workplace for people with stroke?

**Impact and needs of patients:**

1. What do you think were the problems for people who had a stroke during the pandemic in accessing services?
2. What do you think is the impact of the pandemic on rehabilitation services for people with stroke?
3. How do you think this might affect them in terms of their mobility and recovery in the long-term?
4. From a therapist’s perspective, please can you tell me about the patients’ needs during this pandemic in terms of rehabilitation?

**Management strategies:**

1. What were the strategies used to manage access to services?
2. What were the strategies that have been used to deliver rehabilitation for patients during the pandemic?
3. Any examples of good practice used?
4. Was there any efforts to introduce self-management programmes to the patients for their recovery? Were any specific efforts taken to improve their mobility? Did the patients benefit and how?

**Outlook:**

1. When the health services start to reopen what do you consider as priorities for stroke services?
2. What can the healthcare system do to ensure optimal services in the future waves of the pandemic in delivering care and rehabilitation?
3. What can the healthcare system do to ensure optimal services for people in isolation?

**Appendix B:** Topics covered in the stroke survivors’ interviews.

**Experience of stroke:**

1. Please tell me about your experience of having a stroke
2. Please tell me how the stroke has affected you and your daily activities.
3. What do you think were the main challenges that you experienced as a result of the pandemic in accessing services?
4. How did the stroke affect you in terms of your mobility?

**Care received:**

1. Tell me about the care provided by the NHS or other community services after the stroke.
2. What do you think was the impact of the pandemic on your care and rehabilitation services?
3. Tell me about the things or people that you feel were helpful to manage your situation?

**Needs and goals:**

1. Can you tell me about your needs during this period in term of healthcare and rehabilitation services? Which of your needs were not fulfilled?
2. What is your main goal for rehabilitation?

**Management:**

1. Did the healthcare professionals teach you any strategies (to self-manage) that you could use or build on, for your recovery at home after stroke?
2. Apart from the self-management strategies that professionals taught you, please tell me what else have you been doing to manage your situation since the onset of your stroke? Were any specific efforts taken to improve your mobility?
3. More specifically, what have you been doing either on your own (self-management) or with the help of others to achieve your rehabilitation goal?
4. Were the self-management strategies useful for you and in what way?

**Future care:**

1. When the health services start to reopen what do you think are your priorities for your rehabilitation?
2. How can we ensure services are tailored to people affected by stroke in the future waves of the pandemic or when you have to isolate?

**Appendix C**: Priorities for care identified by participants

| Priority (Frequency) | | Supporting quote |
| --- | --- | --- |
|  | Inpatient setting   - Allow family visit and contact time (5) | *“The first one is find a way for family to come, I would say that is definitely a priority.” (P5)* |
|  | - More interactions between patients and healthcare professionals (1) | *“The ward sister hasn’t got time to talk, I never had a conversation with her ever. I would have liked to have known, how am I doing, am I doing well, will I get over this, will I ever walk again, would I be able to look after myself again.” (P6) “In reality on the ward, you know, the precautions are there, you're wearing a mask, we didn't really interact as patients that much.” (P3)* |
|  | More conversations or information regarding mental health with patients (1) | *“I think they should talk to you more, tell you about what's happening, what the prospects are, what you can do to help your mental health, give you more encouragement.” (P6)* |
|  | Allow more time for staff to develop continuous professional development (1) | *“… the extra studying, the time for this study, time for people's well-being, helping people succeed. So put in time for the well-being stuff.” (P5)* |
|  | Improve information technology in NHS (2) | *“I had got an iPad but they got no Wi-Fi. Wi-Fi wasn't working so I couldn't use my iPad or watch films. There was no TV in the ward at all. Nothing to pass the time away. I got nothing to do.” (P6)* |
|  | *Inpatient and Community Settings* |  |
|  | Provide more therapy sessions (3) | *“I hope there would be more OT [Occupation Therapy], I hope there will be more physio. I really like to be in a gym, I'd like to be able to use equipment to do things.” (P5)* |
|  | *Community Setting* |  |
|  | Receive therapies and treatments in a timely manner (1) | *“I would wish everyone got immediately the treatment I got and immediately all the services.” (P1)* |
|  | More follow-up community support, e.g. daily check-in services on phone (3) | *“I think even if just, calling you, check that you are okay, because he [stroke charity representative] might not always ring, and, you know, reach out just to check that you're okay.” (P2) “… the biggest need was to have support and to have somebody who work with you on a daily basis.” (P4)* |
|  | More support from independent stroke organisations (2) | *“I definitely think more support from organisations like Stroke Association. And I know it's difficult, but during the pandemic, just a phone call, I found really, really difficult to deal with.” (P4)* |
|  | More support to family and caregivers (2) | *“They [the family] had no idea what was going on for me, and they were certainly not prepared for me.” (P5) “My sister would have said support from like the council or social services.” (P4)* |
|  | Resume face-to-face general practitioner (GP) consultations (2) | *“That is not only me but this is a national situation, is I don't have access to GP.” (P1) “ If it wasn't a pandemic, they would have been able to see me and… I think that might be the stroke may be may not even have happened.” (P2)* |
| - Provide education regarding stroke to the general public (1) | | *“There needs to be more education in the public… I feel like I missed signs as well. And as well as the hospital missing signs.” (P2)* |

Appendix D: COREQ Checklist

**Manuscript:** Exploring the experiences of people who had a stroke and therapists who managed people with stroke during the Covid-19 pandemic: An exploratory qualitative study.

**Consolidated criteria for reporting qualitative studies (COREQ): 32-item checklist**

Developed from:

Tong A, Sainsbury P, Craig J. Consolidated criteria for reporting qualitative research (COREQ): a 32-item checklist for interviews and focus groups. *International Journal for Quality in Health Care*. 2007. Volume 19, Number 6: pp. 349 – 357

| **No. Item** | **Guide questions/description** | **Reported on Page #** |
| --- | --- | --- |
| **Domain 1: Research team and reﬂexivity** |  |  |
| *Personal Characteristics* |  |  |
| 1. Inter viewer/facilitator | Which author/s conducted the interview or focus group? | Page 6 |
| 2. Credentials | What were the researcher’s credentials? E.g. PhD, MD | Page 6 |
| 3. Occupation | What was their occupation at the time of the study? | Page 1 and 6 |
| 4. Gender | Was the researcher male or female? | Page 1 |
| 5. Experience and training | What experience or training did the researcher have? | Page 1 |
| *Relationship with participants* |  |  |
| 6. Relationship established | Was a relationship established prior to study commencement? | Page 6  . |
| 7. Participant knowledge of the interviewer | What did the participants know about the researcher? e.g. personal goals, reasons for doing the research | Page 6 |
| 8. Interviewer characteristics | What characteristics were reported about the interviewer/facilitator? e.g. Bias, assumptions, reasons and interests in the research topic | Page 6 |

| **Domain 2: study design** |  |  |
| --- | --- | --- |
| *Theoretical framework* |  |  |
| 9. Methodological orientation and Theory | What methodological orientation was stated to underpin the study? e.g. grounded theory, discourse analysis, ethnography, phenomenology, content analysis | Page 1,4 and 6 |
| *Participant selection* |  |  |
| 10. Sampling | How were participants selected? e.g. purposive, convenience, consecutive, snowball | Page 5 |
| 11. Method of approach | How were participants approached? e.g. face-to-face, telephone, mail, email | Page 5 |
| 12. Sample size | How many participants were in the study? | Page 1 and 7 |
| 13. Non-participation | How many people refused to participate or dropped out? Reasons? | Page 7 |
| *Setting* |  |  |
| 14. Setting of data collection | Where was the data collected? e.g. home, clinic, workplace | Page 6  . |
| 15. Presence of non-participants | Was anyone else present besides the participants and researchers? | Page 5 |
| 16. Description of sample | What are the important characteristics of the sample? e.g. demographic data, date | Page 5 and 6 |
| *Data collection* |  |  |
| 17. Interview guide | Were questions, prompts, guides provided by the authors? Was it pilot tested? | Page 5 and 6 (copy of the guides attached as appendices A and B) |
| 18. Repeat interviews | Were repeat interviews carried out? If yes, how many? | No Page 5 |
| 19. Audio/visual recording | Did the research use audio or visual recording to collect the data? | Page 6 |
| 20. Field notes | Were ﬁeld notes made during and/or after the inter view or focus group? | Page 6 |
| 21. Duration | What was the duration of the interviews or focus group? | Page 6 |
| 22. Data saturation | Was data saturation discussed? | Page 6 |
| 23. Transcripts returned | Were transcripts returned to participants for comment and/or correction? | Page 7 |
| **Domain 3: analysis and ﬁndings** |  |  |
| *Data analysis* |  |  |
| 24. Number of data coders | How many data coders coded the data? | Page 7 |
| 25. Description of the coding tree | Did authors provide a description of the coding tree? | Page 7 |
| 26. Derivation of themes | Were themes identiﬁed in advance or derived from the data? | Page 7 |
| 27. Software | What software, if applicable, was used to manage the data? | NA |
| 28. Participant checking | Did participants provide feedback on the ﬁndings? | Page 7 |
| *Reporting* |  |  |
| 29. Quotations presented | Were participant quotations presented to illustrate the themes/ﬁndings? Was each quotation identiﬁed? e.g. participant number | Page 9 to 21 |
| 30. Data and ﬁndings consistent | Was there consistency between the data presented and the ﬁndings? | Page 9 to 21 |
| 31. Clarity of major themes | Were major themes clearly presented in the ﬁndings? | Page 9 to 21 |
| 32. Clarity of minor themes | Is there a description of diverse cases or discussion of minor themes? | Page 9 to 25 |
