# Supplementary material for: Exploring the experiences of people who had a stroke and therapists who managed people with stroke during the COVID-19 pandemic: An exploratory qualitative study
Source: PLoS One. 2023 Feb 28;18(2):e0282325. doi: 10.1371/journal.pone.0282325 (PMC9974115; doi:10.1371/journal.pone.0282325)
Supplement: Attachment — Submitted filename: Response to Reviewers.docx [file pone.0282325.s003.docx]

**Comment to the reviewer:**

Thank you very much for your thoughtful comments on the submitted manuscript. Here is the response to the comments in details:

| **Reviewer’s Comments** | **Response** |
| --- | --- |
| 1. The description of the different stages of recovery acute, subacute and community are specified in the abstract and in the results. Make a more explicit link to this point in the sampling and eligibility criteria in the methods. | Thank you for the helpful comment. To improve the link, we have added justification for the inclusion criteria and sampling strategy based on recovery stages to the methods section on page 5. |
| 2. For Table 2 add a key to interpret the scores of the BI, SSQOL, MMSE and GDS? | A key has been added to table 2 with the indicative information for BI, SSQOL, MMSE and GDS to explain the range of scores along with supportive evidence where available. |
| 3. Results - for most of the themes there is a balance between stroke survivor and therapist quotes. For some of the themes eg disruption of services, could the authors include a therapists quote? likewise this is the case for communication issues, a therapist quote could be included here as well? | Thank you for this comment. We have added some quotes from therapists to establish the balance between patient and therapist quotes for the suggested points (for disruption of services on page 10 and for communication on page 14). |
| 4. Typo, Discussion para 3, line 1 list should be visits? | Yes, this has been corrected. Thank you |
| 5. Recall of acute and subacute stages is this a potential limitation as well? | This potential limitation has been recognised and added to the limitations section on page 26. |
| 6. Clinical messages - consider merging the two points relating self management and add a clinical message regarding use of technology, reflecting on the experiences found in this work and future areas to consider in relation to the use of technology in stroke rehabilitation. | Changes have been made considering your suggestions. Thank you |
| 7. Include the completed COREQ checklist as an appendix? | The COREQ checklist has been added as appendix D |
